# Chiral Brønsted acid-controlled intermolecular asymmetric [2 + 2] photocycloadditions

**DOI:** 10.1038/s41467-021-25878-9

**Published:** 2021-09-30

**Authors:** Evan M. Sherbrook, Matthew J. Genzink, Bohyun Park, Ilia A. Guzei, Mu-Hyun Baik, Tehshik P. Yoon

**Affiliations:** 1 Department of Chemistry, University of Wisconsin–Madison, 1101 University Avenue, Madison, Wisconsin 53706 USA; 2 Department of Chemistry, Korea Advanced Institute of Science and Technology (KAIST), Daejeon, 34141 Republic of Korea; 3 Center for Catalytic Hydrocarbon Functionalizations, Institute for Basic Science (IBS), Daejeon, 34141 Republic of Korea

**Keywords:** Asymmetric catalysis, Photocatalysis, Synthetic chemistry methodology

## Abstract

Control over the stereochemistry of excited-state photoreactions remains a significant challenge in organic synthesis. Recently, it has become recognized that the photophysical properties of simple organic substrates can be altered upon coordination to Lewis acid catalysts, and that these changes can be exploited in the design of highly enantioselective catalytic photoreactions. Chromophore activation strategies, wherein simple organic substrates are activated towards photoexcitation upon binding to a Lewis acid catalyst, rank among the most successful asymmetric photoreactions. Herein, we show that chiral Brønsted acids can also catalyze asymmetric excited-state photoreactions by chromophore activation. This principle is demonstrated in the context of a highly enantio- and diastereoselective [2+2] photocycloaddition catalyzed by a chiral phosphoramide organocatalyst. Notably, the cyclobutane products arising from this method feature a *trans*-*cis* stereochemistry that is complementary to other enantioselective catalytic [2+2] photocycloadditions reported to date.

## Abstract (web summary)

Lewis acids have recently been shown to enable stereocontrol in photochemical cycloadditions, a difficult task due to the reactivity of excited-state compounds. Here the authors show that chiral Brønsted acids are competent chromophore activators in [2+2] cycloadditions, forming diastereomers disfavored in similar Lewis acid catalyzed photochemical reactions.

## Introduction

**Fig. 1:**
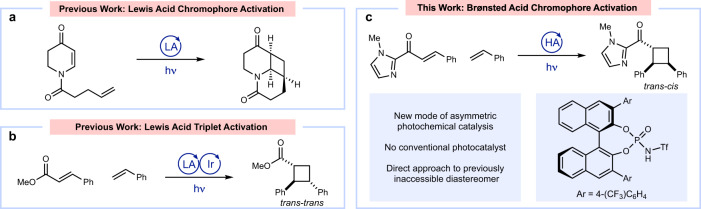
Asymmetric acid-catalyzed photoreactions. **a** Intramolecular [2 + 2] photocycloadditions via Lewis acid-catalyzed chromophore activation; LA = Lewis acid. **b** Intermolecular [2 + 2] photocycloadditions via Lewis acid-catalyzed triplet activation. **c** Intermolecular [2 + 2] photocycloadditions via Bronsted acid-catalyzed chromophore activation. HA = Brønsted acid.

A defining characteristic of modern synthetic chemistry is the capacity to conduct complexity-building organic reactions with high levels of stereocontrol. The strategies available to dictate the stereochemical outcome of photochemical reactions, however, remain significantly underdeveloped compared to other classes of organic reactions^1–8^. The difficulty of conducting asymmetric photochemical reactions has often been attributed to the very short lifetimes and high reactivities associated with electronically excited compounds; these features challenge the ability of catalysts to intercept and modulate the behavior of organic excited states. In the past few years, highly enantioselective photoreactions have increasingly exploited chiral Lewis acids, either as catalysts or as co-catalysts. The insight that the same privileged classes of chiral Lewis acid structures that have been so enabling in ground-state asymmetric synthesis can also be applied to enantioselective photochemistry has increased the pace of discovery in this area. There are two general mechanisms by which chiral Lewis acids can influence the enantioselectivity of excited-state photoreactions. First, Bach pioneered the chromophore activation strategy in which chiral Lewis acids are used to attenuate the singlet excited-state energy of α,β-unsaturated carbonyl compounds (Fig. 1a)^9,10^. As the absorption of the catalyst-bound substrate occurs more strongly at longer wavelengths than the free substrate, the activated substrate may be selectively excited by a judicious choice of irradiation wavelength. Second, our group has developed a mechanistically distinctive strategy that we have termed “triplet activation.” In this approach, chiral Lewis acid coordination lowers the triplet energy of α,β-unsaturated carbonyls, allowing for their selective activation by a triplet sensitizer over unbound substrate molecules (Fig. 1b)^11–13^. Although fundamentally distinct, both strategies use chiral Lewis acids to modify the excited-state properties of the substrate and preferentially activate the catalyst-bound complex, thereby minimizing racemic background reactivity.

In the first decade of the twenty-first century, secondary amine and Brønsted acid organocatalysis emerged as an alternative approach to chiral Lewis acid catalysis for a wide range of asymmetric ground-state transformations^14–17^. Remarkably, these catalysts could control many of the same classes of asymmetric transformations as better-studied chiral Lewis acids, often enabling the use of functionalized substrates that would not be compatible with strongly Lewis acidic catalyst structures. One might reasonably wonder if organocatalysts might prove equally versatile in asymmetric photochemistry. Very recently, Takagi and Tabuchi^18^ demonstrated that chiral phosphoric acids are effective stoichiometric templates for asymmetric [2 + 2] photocycloaddition reactions of quinolones, although this interaction had little effect on the photophysical properties of the substrate. Pioneering studies by Melchiorre and colleagues^19,20^ have demonstrated that secondary amine organocatalysts can activate prochiral enals by chromophore activation: the iminium intermediates absorb at longer wavelengths than the free enal substrates and the excited states have been utilized in a variety of useful organic transformations. Very recently, Bach and colleagues^21,22^ demonstrated the first example of triplet activation by secondary amine organocatalysis, demonstrating that the vinyl iminium intermediate undergoes energy transfer faster than the free parent enal and can be the key intermediate in an enantioselective [2 + 2] photocycloaddition. The ability of Brønsted acid catalysts to engage in chromophore and triplet activation mechanisms, however, has been underexplored. Although hydrogen bonding and Brønsted acid catalysts have been studied extensively as chiral sensitizers for asymmetric photoreactions^23–25^, their ability to alter the photophysical properties of organic substrates has not been significantly exploited. In an isolated example, Sivaguru and colleagues^26,27^ described an enantioselective intramolecular [2 + 2] cycloaddition using a chiral thiourea catalyst that could activate a coumarin substrate through both static and dynamic complex formation. Our group also recently reported the first example of triplet activation in a racemic [2 + 2] photocycloaddition reaction in which the addition of *p*-TsOH as a co-catalyst was demonstrated to increase the rate of triplet energy transfer from a Ru(II) photocatalyst to 2-acyl imidazole **1**, which subsequently underwent a [2 + 2] photocycloaddition^28^.

In this work, we report a highly enantio- and diastereoselective excited-state [2 + 2] cycloaddition catalyzed by a 1,1′-Bi-2-naphthol (BINOL)-derived phosphoramide Brønsted acid catalyst. A combination of spectroscopic and computational studies indicate that this catalyst operates via the principle of chromophore activation (Fig. 1c).

## Results

### Reaction development

**Fig. 2:**
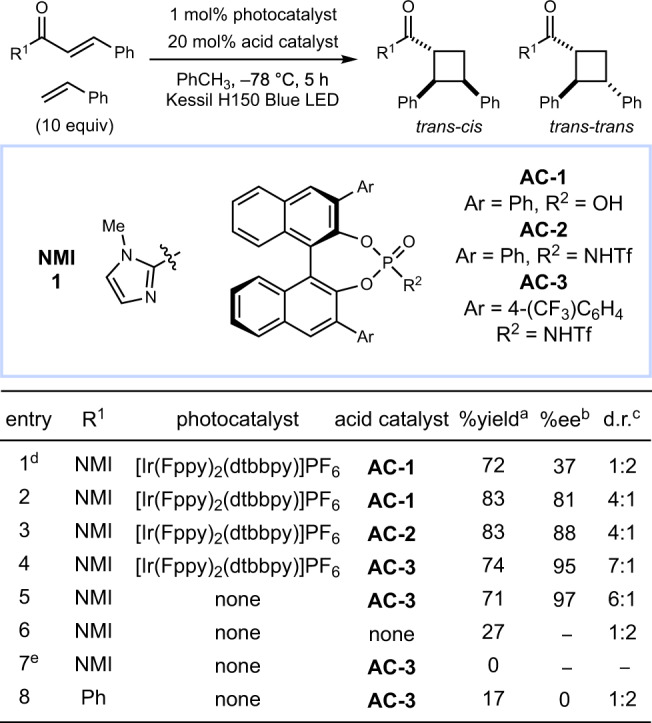
Optimization studies. ^a^Yields and diastereomer ratios determined by ^1^H NMR spectroscopy. ^b^Enantiomeric excess of the major diastereomer determined by chiral HPLC. ^c^Ratio of *trans*–*cis* isomer to *trans*–*trans* isomer. ^d^Conducted at room temperature. ^e^Conducted in the dark.

The discovery of this chiral organocatalytic photoreaction occurred serendipitously during our studies to further develop the *p*-TsOH/photocatalytic [2 + 2] cycloaddition method into an asymmetric reaction by the use of a chiral Brønsted acid. We began by screening BINOL-derived Brønsted acid co-catalysts, as they constitute a privileged catalyst class in thermal asymmetric transformations (Fig. 2)^29^. When **1** and styrene were irradiated in the presence of 1 mol% [Ir(Fppy)_2_(dtbbpy)]PF_6_ and 20 mol% acid catalyst **AC-1**, the cycloadduct was obtained in 37% ee in a 1:2 ratio of *trans*–*cis* to *trans*–*trans* diastereomers (entry 1). We observed a reversal in the diastereoselectivity at −78 °C and a concomitant increase in enantioselectivity (81% ee, entry 2). The more acidic *N*-triflyl phosphoramide **AC-2** led to a further increase in enantioselectivity (entry 3), consistent with a stronger substrate–catalyst interaction^30^. Finally, replacing the phenyl groups on the catalyst with 4-trifluoromethylphenyl groups (**AC-3**) led to the optimal catalyst, affording the cycloadduct in 7:1 d.r. and 95% ee (entry 4).

To our surprise, control experiments omitting the photocatalyst from the reaction gave nearly identical yield and selectivity (entry 5). This indicates that **AC-3** is not only responsible for the selectivity of the reaction but also activates the substrate towards photoexcitation. In the absence of both catalysts, the racemic product was still formed in low yield, indicative of a slow direct photoreaction that is outcompeted by the acid-catalyzed reaction (entry 6). Notably, in this control experiment, the *trans*–*trans* diastereomer is favored in a 2:1 ratio, indicating that the diastereoselectivity of the acid-catalyzed reaction (entry 5) is a product of catalyst control. Chalcone was also tested as a substrate and gave diminished yields of product with no ee, suggesting that a sufficiently basic functionality capable of interacting with the Brønsted acid catalyst is required (entry 8). The *trans*–*cis* diastereoselectivity of the major product is notable: direct excitation and triplet-sensitized reactions of acyclic enones with alkenes typically favor formation of the *trans*–*trans* isomer^31–34^. Indeed, the direct photoreaction conducted in the absence of the chiral Brønsted acid (entry 6) favors the *trans*–*trans* diastereomer and thus we conclude that the observed diastereoselectivity is imposed by the acid catalyst. This method constitutes the first enantioselective photocycloaddition that selectively accesses a simple monocyclic diarylcyclobutane with 1,2-*cis* diastereoselectivity.

### Reaction scope

**Fig. 3:**
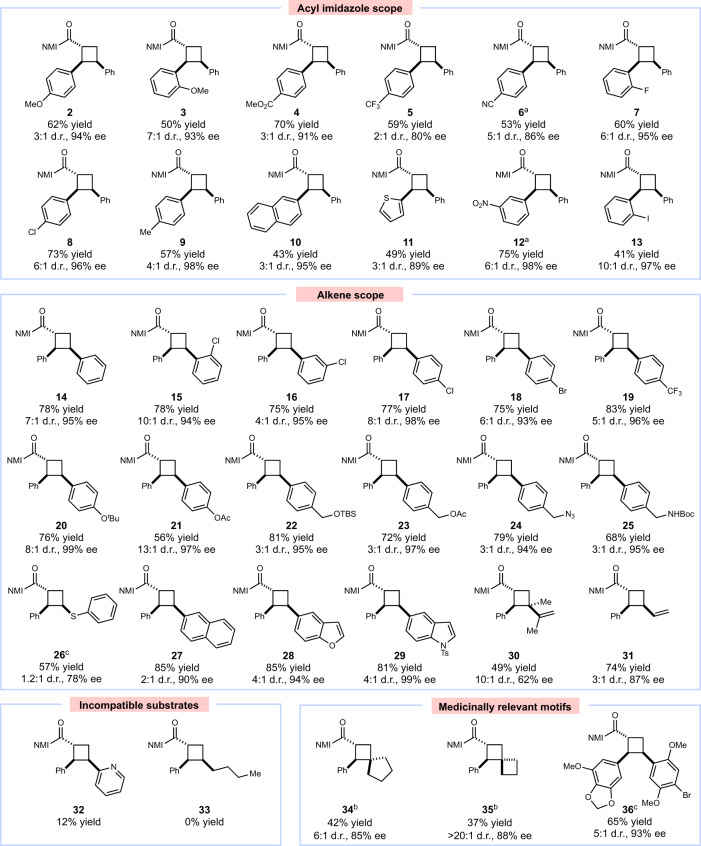
Scope studies. Reactions conducted using 1 equiv 2-acyl imidazole, 10 equiv alkene, 20 mol% **AC-3** in toluene, irradiating for 14 h with a Kessil H150 LED unless otherwise noted. Yields represent the isolated yield of both diastereomers. Enantiomeric excess of the major diastereomer determined by chiral HPLC. ^a^Conducted for 24 h. ^b^Conducted with 40 mol% acid catalyst **AC-3**. ^c^Conducted in CH_2_Cl_2_.

**Fig. 4:**
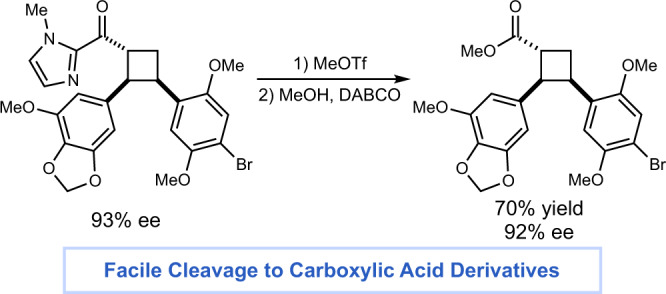
Auxiliary cleavage. The imidazolyl group of the cycloadduct is readily cleaved in good yield with retention of enantioselectivity.

We evaluated the scope of the reaction under optimized reaction conditions (Fig. 3). A variety of α,β-unsaturated carbonyl compounds with electronically varied β-aryl substituents (**2**–**9**) were accommodated, producing the corresponding cycloadducts in high yields and selectivities. Substrates with substituents at the ortho position (**3**, **7**) reacted slower than corresponding substrates with meta or para substitution but were formed in higher diastereoselectivity. A thienyl substrate (**11**) reacted efficiently. Finally, as this reaction does not require a conventional photocatalyst, substrates with oxidation or reduction potentials within the range of common transition metal photocatalysts are tolerated^35^, including nitroarenes (**12**) and aryl iodides (**13**). The alkene acceptor scope also includes substitution at each position of the arene acceptor (**15**–**17**) and an array of electron-withdrawing (**18**–**19**) and -donating (**20**–**21**) substituents. Notably, a *p*-alkoxy-substituted styrene (**20**) was tolerated under these conditions, whereas the same alkene undergoes rapid polymerization using our previous Lewis acid-catalyzed methods^11^. Various functional groups including protected alcohols (**22**–**23**), a photolabile azide (**24**), and a protected amine (**25**) were all tolerated. Heterocycles were also readily transformed including benzofuran- and indole-substituted alkenes (**28**–**29**). Substituted and unsubstituted dienes (**30**–**31**) were competent substrates. Any substrate that features alternate binding sites for the chiral catalyst, however, generally affords low reactivity (**32**). Intriguingly, two aliphatic exocyclic alkenes (**34**–**35**) gave moderate yields and good ee to afford medicinally interesting spirocyclic products^36^. Cycloadditions between two densely functionalized reaction partners also provided high enantioselectivity (**36**) and cleavage of the imidazolyl group to a methyl ester occurred with no significant loss of stereochemical fidelity (Fig. 4).

### Mechanistic studies

**Fig. 5:**
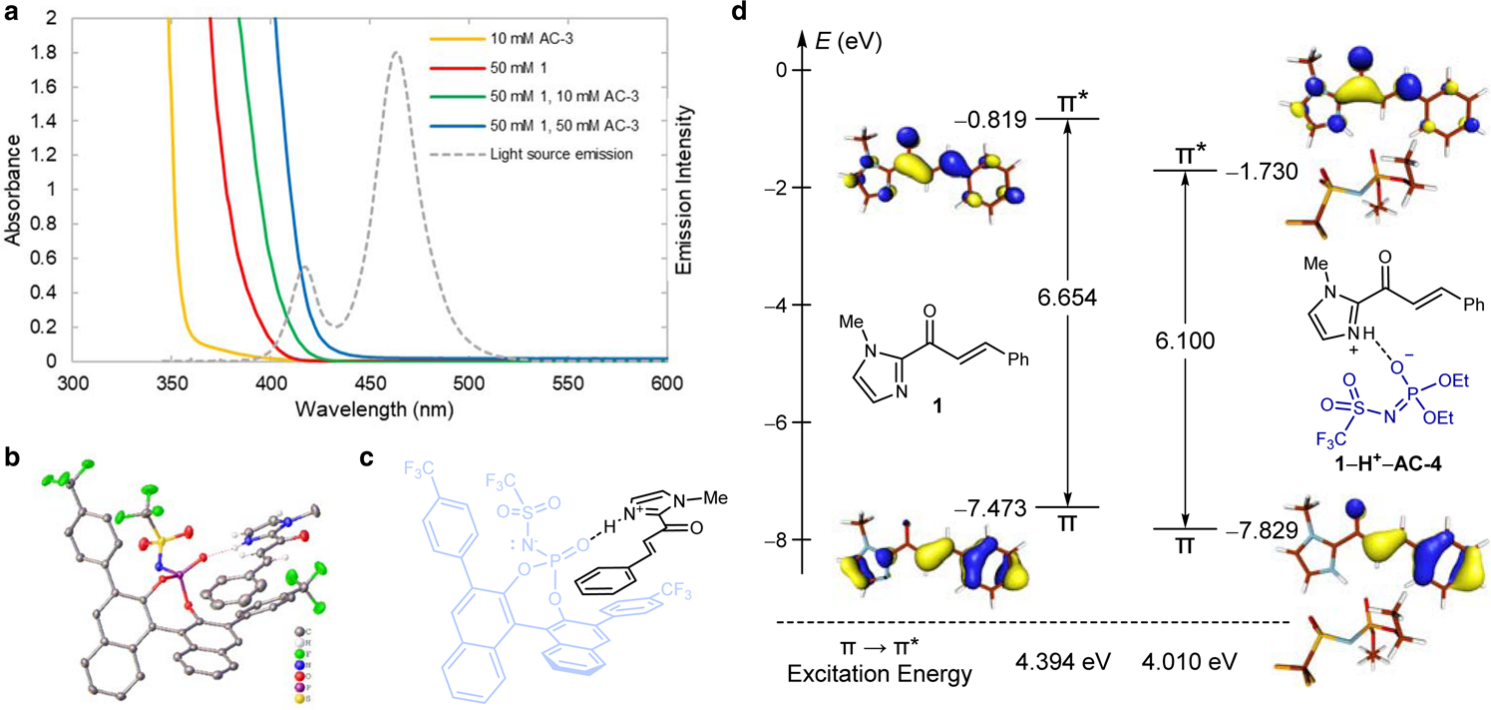
Mechanistic studies. **a** Ultraviolet-visible spectra of **1** and **AC-3** in CH_2_Cl_2_. **b** X-ray crystal structure of acid-bound substrate. **c** Binding depiction of acid-bound substrate. **d** HOMO(*π*) and LUMO(π*) of **1** and **1**–**H**^+^–**AC-4**.

An intriguing outcome of our optimization study was the unexpected observation that **AC-3** catalyzes this reaction without a separate triplet sensitizer. We therefore elected to investigate its role in greater detail. Neither **1** nor **AC-3** absorbs appreciably in the visible region (Fig. 5a), and as the emission of the light source does not overlap with the absorption of the acid catalyst, direct excitation of the acid catalyst is not feasible. We therefore conclude that the Brønsted acid is not a conventional triplet photosensitizer in this reaction. The substrate, on the other hand, absorbs weakly at wavelengths >400 nm, accounting for the slow background reaction in the absence of catalyst (Fig. 2, entry 6). Upon combining colorless solutions of the acid and substrate, the mixture became noticeably yellow, corresponding to a red shift in absorption that increased with added acid catalyst. The emission of the light source overlapped well with bound but poorly with unbound substrate, consistent with relatively minimal contribution from the racemic background reaction.

We next performed nuclear magnetic resonance (NMR) titration experiments with **1** and **AC-3** in toluene-*d*_8_ and calculated an association constant of *K*_a_ = 7.5 × 10^6^ (Supplementary Fig. 5). We also isolated the substrate-acid complex from diethyl ether and determined its structure using X-ray crystallography (Fig. 5b, c). Key features of the interaction included an apparent hydrogen bond between the protonated imidazolium on the substrate and the phosphoramide oxygen on the catalyst. This interaction differs significantly from the typical chelating interactions of acyl imidazoles with chiral Lewis acid catalysts, including the chiral-at-metal Rh catalysts Meggers and colleagues^37,38^ have utilized in analogous asymmetric [2 + 2] photocycloaddition reactions. Further, the orientation of the substrate alkene relative to the *π*-surface of the biaryl catalyst renders the top face of the enone more accessible for cycloaddition, which is consistent with the experimentally observed absolute configuration of the cycloadduct. Although both the substrate and acid catalyst are white solids, the isolated crystals were yellow, indicating that the complex absorbs visible light. Together, these results are suggestive that the crystal structure could be similar to the catalytically active complex in solution.

**Fig. 6:**
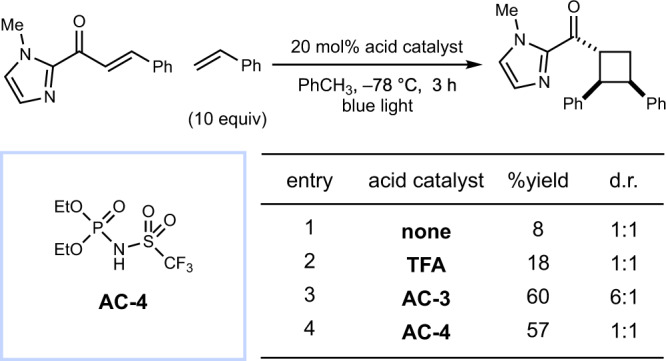
Effect of structurally diverse Brønsted acids on the cycloaddition. Yields and diastereomer ratios determined by ^1^H NMR spectroscopy.

Based on these data, we considered two possible modes of substrate activation within the acid–substrate complex. First, we considered a chromophore activation mechanism in which protonation of the substrate lowers the relative energy of its first singlet excited state, thus making direct excitation by visible light achievable. This hypothesis is consistent with the red-shifted absorption of enones in sulfuric acid solution^39,40^. Second, we considered that the chiral catalyst and enone substrate might form an electron donor–acceptor complex^41–45^. In this scenario, a new intermolecular transition between the highest-occupied molecular orbital (HOMO) of the electron-rich phosphoramide donor and the lowest-unoccupied molecular orbital (LUMO) of the electron-deficient imidazolium acceptor could be lower in energy than the intramolecular transitions on either individual species. To distinguish between these mechanisms, we examined the influence of various acid catalysts on the yield of the photocycloaddition (Fig. 6). If the electron donor–acceptor mechanism were operative, acid catalysts lacking the *π*-conjugation of **AC-3** would not form photochemically active complexes; however, a variety of acids increased the reaction rate, with similar apparent reaction rates using **AC-3** and **AC-4**. Therefore, the bathochromic shift in the absorption of the substrate is likely an intrinsic property of the protonated substrate and not the result of a specific donor–acceptor interaction.

To understand the origin of this effect, we conducted density functional theory (DFT) and time-dependent DFT (TDDFT) calculations (Fig. 5d). Unsurprisingly, the HOMO and LUMO of **1** were found to be fully delocalized across the molecule with a HOMO–LUMO gap of 6.65 eV. The *π*-conjugation in the HOMO can be divided into an imidazolium and a cinnamoyl part. After protonation (**1H**^**+**^**–AC-4**), the HOMO becomes significantly more localized and is dominated by the cinnamoyl part (see Supplementary Information for details). This demixing of the two *π*-components upon protonation weakens the electrostatic impact of the proton on the HOMO energy, resulting in a relatively moderate shift of −0.36 eV. In contrast, the LUMO maintains delocalization across both *π*-domains and the LUMO energy shifts by −0.91 eV upon protonation. Consequently, the HOMO–LUMO gap is reduced from 6.65 to 6.10 eV, enabling excitation by visible light. TDDFT/Tamm–Dancoff approximation calculation and natural transition orbital analysis revealed the excitation from HOMO to LUMO (*π* → π* transition) in **1** and **1H**^**+**^**–AC-4** to be most important, and the excitation energies of 4.40 and 4.01 eV, respectively, were in good agreement with experimental observations.

Given this combination of synthetic, spectroscopic, and computational evidence, we propose that the BINOL-derived acid acts as a dual-purpose catalyst, altering the absorption spectrum of the substrate and providing a stereodifferentiating environment for the cycloaddition. The red-shifted absorption of the bound substrate enables selective excitation of the catalyst-bound complex and minimal competition from direct racemic background photocycloaddition. The resulting excited-state substrate is preorganized relative to the chiral environment defined by the Brønsted acid catalyst and styrene approaches at the accessible face of the enone, yielding highly enantioenriched product.

We have developed a highly enantioselective [2 + 2] photocycloaddition catalyzed by a BINOL phosphoramide organocatalyst. We believe that these results are significant for a variety of reasons. First, the stereocontrol offered by this reaction is high and the access it provides to the *trans*–*cis* diastereomer of diarylcyclobutanes distinguishes it synthetically from previous reports of asymmetric [2 + 2] photocycloadditions. Thus, although highly enantioselective catalysis of excited-state reactions is a relatively new capability in synthetic chemistry, the level of comprehensive control these methods collectively provide are beginning to approach the sophistication available in more conventional asymmetric reactions. More importantly, this study demonstrates that chiral Brønsted acids can activate enones by altering the absorption properties of the substrate. The extension of the concept of chromophore activation to chiral Brønsted acid-catalyzed photoreactions adds a distinctive approach to the strategic toolbox available for the design of stereocontrolled organic photoreactions.

## Methods

### General

Photochemical reactions were carried out with a Kessil H150-Blue lamp. Diastereomer ratios for reactions were determined by ^1^H NMR analysis of unpurified reaction mixtures vs. a phenanthrene internal standard. ^1^H and ^13^C NMR data were obtained using a Bruker Avance-500 spectrometer with a DCH cryoprobe and are referenced to tetramethylsilane (0.0 p.p.m.) and CDCl_3_ (77.0 p.p.m.), respectively. ^19^F and ^31^P NMR data were obtained using Bruker Avance-400 spectrometer. NMR data are reported as follows: chemical shift, multiplicity (s = singlet, d = doublet, t = triplet, q = quartet, p = pentet, sext = sextet, sept = septet, m = multiplet), coupling constant(s) in Hz, integration. NMR spectra were obtained at 298 K unless otherwise noted. Fourier-transform infrared spectra were obtained using a Bruker Tensor 27 spectrometer and are reported in terms of frequency of absorption (cm^−1^). Melting points were obtained using a Stanford Research Systems DigiMelt MPA160 melting point apparatus and are uncorrected. Mass spectrometry was performed with a Thermo Q ExactiveTM Plus using electrospray ionization-time of flight. Enantiomeric excesses were determined by chiral high-performance liquid chromatography (HPLC) of isolated materials using a Waters e2695 separations module with 2998 PDA detector and Daicel CHIRALPAK® columns, and HPLC grade *i*-PrOH and hexanes. Traces were acquired using Empower 3® software. Optical rotations were measured using a Rudolf Research Autopol III polarimeter at room temperature in CH_2_Cl_2_. Ultraviolet-visible absorption spectra were acquired using a Varian Cary® 50 UV-visible spectrophotometer. For the preparation of catalysts and substrates, see Supplementary Methods. For mechanistic experiments, see Supplementary Figs. 1–6; for crystallographic data, see Supplementary Figs. 7–12; and for computational data, see Supplementary Figs. 13 and 14.

### General photoreaction procedure

An oven-dried Schlenk tube was charged with acyl imidazole (0.4 mmol, 1.0 equiv), styrene (4.0 mmol, 10.0 equiv.), **(*R*)-AC-3** (0.08 mmol, 0.2 equiv.), and 8 mL toluene. The Schlenk tube was sealed with a glass stopper and degassed via freeze–pump–thaw technique (3 × 5 min). This was then cooled to −78 °C and irradiated with a Kessil H150-Blue lamp for 14 h. The reaction mixture was then diluted 2–3× with CH_2_Cl_2_ before addition of 2 mL sat. aq. NaHCO_3_. The mixture was vigorously stirred for 1 min, the organic layer separated, and the aqueous layer extracted with CH_2_Cl_2_ (2 × 4 mL). The combined organics were dried over Na_2_SO_4_, concentrated, and analyzed by ^1^H NMR vs. internal standard (phenanthrene), to determine conversion and diastereomeric ratio. The crude mixture was then purified via flash column chromatography using Et_2_O/pentanes as the eluent, giving an isolated mixture of separable diastereomers. The major diastereomer of each mixture was characterized.

## Supplementary information

Supplementary Information

Description of Additional Supplementary Files

Supplementary Data 1

## Data Availability

All data generated in this study are provided in the Supplementary Information files. The X-ray crystallographic coordinates for compound **18** and for the **1**•**AC-3** salt have been deposited at the Cambridge Crystallographic Data Centre (CCDC) under deposition numbers CCDC-2022776 and CCDC-2022777, respectively. These data can be obtained free of charge from The Cambridge Crystallographic Data Centre via https://www.ccdc.cam.ac.uk/data_request/cif.

## References

[1] Rau H (1983). Asymmetric photochemistry in solution. Chem. Rev..

[2] Inoue Y (1992). Asymmetric photochemical reactions in solution. Chem. Rev..

[3] Inoue, Y. & Ramamurthy, V. *Chiral Photochemistry. Molecular and Supramolecular Photochemistry* Vol. 11 (Marcel Dekker, 2004).

[4] Brimioulle R, Lenhart D, Maturi MM, Bach T (2015). Enantioselective catalysis of photochemical reactions. Angew. Chem. Int. Ed..

[5] Zou Y-Q, Hörmann FM, Bach T (2018). Iminium and enamine catalysis in enantioselective photochemical reactions. Chem. Soc. Rev..

[6] Sherbrook, E. M. & Yoon, T. P. in *Specialist Periodical Reports: Photochemistry* (eds Albini, A. & Protti, S.) Vol. 46, 432−448 (Royal Society of Chemistry, 2019).

[7] Prentice C, Morrisson J, Smith AD, Zysman-Colman E (2020). Recent developments in enantioselective photocatalysis. Beilstein J. Org. Chem..

[8] Rigotti T, Alemán J (2020). Visible light photocatalysis – from racemic to asymmetric activation strategies. Chem. Commun..

[9] Brimioulle R, Bach T (2013). Enantioselective Lewis acid catalysis of intramolecular enone [2+2] photocycloaddition reactions. Science.

[10] Brenninger C, Jolliffe JD, Bach T (2018). Chromophore activation of α,β-unsaturated carbonyl compounds and its application to enantioselective photochemical reactions. Angew. Chem. Int. Ed..

[11] Blum TR, Miller ZD, Bates DM, Guzei IA, Yoon TP (2016). Enantioselective photochemistry through Lewis acid-catalyzed triplet energy transfer. Science.

[12] Miller ZD, Lee BJ, Yoon TP (2017). Enantioselective crossed photocycloadditions of styrenic olefins by Lewis acid catalyzed triplet sensitization. Angew. Chem. Int. Ed..

[13] Daub ME (2019). Enantioselective [2+2] cycloadditions of cinnamate esters: generalizing Lewis acid catalysis of triplet energy transfer. J. Am. Chem. Soc..

[14] Erkkilä A, Majander I, Pihko PM (2007). Iminium catalysis. Chem. Rev..

[15] Mukherjee S, Yang JW, Hoffmann S, List B (2007). Asymmetric enamine catalysis. Chem. Rev..

[16] Doyle AG, Jacobsen EN (2007). Small-molecule H-bond donors in asymmetric catalysis. Chem. Rev..

[17] Akiyama T (2007). Stronger Brønsted acids. Chem. Rev..

[18] Takagi R, Tabuchi C (2020). Enantioselective intramolecular [2+2] photocycloaddition using phosphoric acid as a chiral template. Org. Biomol. Chem..

[19] Silvi M, Verrier C, Rey YP, Buzzetti L, Melchiorre P (2017). Visible-light excitation of iminium ions enables the enantioselective catalytic β-alkylation of enals. Nat. Chem..

[20] Verrier C (2018). Direct stereoselective installation of alkyl fragments at the β-carbon of enals via excited iminium ion catalysis. ACS Catal..

[21] Hörmann FM, Chung TS, Rodriguez E, Jakob M, Bach T (2018). Evidence for triplet sensitization in the visible‐light‐induced [2+2] photocycloaddition of eniminium ions. Angew. Chem. Int. Ed..

[22] Hörmann FM (2020). Triplet energy transfer from ruthenium complexes to chiral eniminium ions: enantioselective synthesis of cyclobutanecarbaldehydes by [2+2] photocycloaddition. Angew. Chem. Int. Ed..

[23] Bibal B, Mongin C, Bassani DM (2014). Template effects and supramolecular control of photoreactions in solution. Chem. Soc. Rev..

[24] Ramamurthy V, Sivaguru J (2016). Supramolecular photochemistry as a potential synthetic tool: photocycloaddition. Chem. Rev..

[25] Burg F, Bach T (2019). Lactam hydrogen bonds as control elements in enantioselective transition-metal-catalyzed and photochemical reactions. J. Org. Chem..

[26] Vallavoju N, Selvakumar S, Jockusch S, Sibi MP, Sivaguru J (2014). Enantioselective organo-photocatalysis mediated by atropisomeric thiourea derivatives. Angew. Chem. Int. Ed..

[27] Vallavoju N (2016). Organophotocatalysis: insights into the mechanistic aspects of thiourea-mediated intermolecular [2+2] photocycloadditions. Angew. Chem. Int. Ed..

[28] Sherbrook EM, Jung H, Cho D, Baik M-H, Yoon TP (2020). Brønsted acid catalysis of photosensitized cycloadditions. Chem. Sci..

[29] Parmar D, Sugiono E, Raja S, Rueping M (2014). Complete field guide to asymmetric BINOL-phosphate derived Brønsted acid and metal catalysis: history and classification by mode of activation; Brønsted acidity, hydrogen bonding, ion pairing, and metal phosphates. Chem. Rev..

[30] Nakashima D, Yamamoto H (2006). Design of chiral *N*-triflyl phosphoramide as a strong chiral Bronsted acid and its application to asymmetric Diels–Alder reaction. J. Am. Chem. Soc..

[31] Du J, Skubi KL, Schultz DM, Yoon TP (2014). A dual-catalysis approach to enantioselective [2+2] photocycloadditions using visible light. Science.

[32] Lei T (2017). General and efficient intermolecular [2+2] photodimerization of chalcones and cinnamic acid derivatives in solution through visible-light catalysis. Angew. Chem. Int. Ed..

[33] Li R (2017). Photocatalytic regio- and stereo-selective [2+2] cycloaddition of styrene derivatives using a heterogeneous organic photocatalyst. ACS Catal..

[34] Fan X, Lei T, Chen B, Tung C-H, Wu L-Z (2019). Photocatalytic C–C bond activation of oxime ester for acyl radical generation and application. Org. Lett..

[35] Nguyen JD, D’Amato EM, Narayanam JM, Stephenson CR (2012). Engaging unactivated alkyl, alkenyl and aryl iodides in visible-light-mediated free radical reactions. Nat. Chem..

[36] Carreira EM, Fessard TC (2014). Four-membered ring-containing spirocycles: synthetic strategies and opportunities. Chem. Rev..

[37] Huang X (2017). Direct visible-light-excited asymmetric Lewis acid catalysis of intermolecular [2+2] photocycloadditions. J. Am. Chem. Soc..

[38] Jung H (2021). Understanding the mechanism of direct visible-light-acitivated [2+2] cycloadditions mediated by Rh and Ir photocatalysts: combined computational and spectroscopic studies. Chem. Sci..

[39] Zalewski RI, Dunn GE (1968). Protonation of conjugated carbonyl groups in sulfuric acid solutions. I. Adaptation of the amide acidity function H_A_ for protonation of the carbonyl group in non-Hammett bases. Can. J. Chem..

[40] Zalewski RI, Dunn GE (1969). Protonation of conjugated carbonyl groups in sulfuric acid solutions. II. Protonation and basicity of α,β-unsaturated alicyclic ketones. Can. J. Chem..

[41] Mulliken RS (1952). Molecular compounds and their spectra. J. Am. Chem. Soc..

[42] Rosokha SV, Kochi JK (2008). Fresh look at electron-transfer mechanisms via the donor-acceptor bindings in the critical encounter complex. Acc. Chem. Res..

[43] Lima CG, Lima TM, Duarte M, Jurberg ID, Paixão MW (2016). Organic synthesis enabled by light-irradiation of EDA complexes: theoretical background and synthetic applications. ACS Catal..

[44] Arceo E, Jurberg ID, Álvarez-Fernández A, Melchiorre P (2013). Photochemical activity of a key donor-acceptor complex can drive stereoselective catalytic α-alkylation of aldehydes. Nat. Chem..

[45] Bahamonde A, Melchiorre P (2016). Mechanism of the stereoselective α-alkylation of aldehydes driven by the photochemical activity of enamines. J. Am. Chem. Soc..

