# Supplementary material for: Chiral Brønsted acid-controlled intermolecular asymmetric [2 + 2] photocycloadditions
Source: Nat Commun. 2021 Sep 30;12:5735. doi: 10.1038/s41467-021-25878-9 (PMC8484615; doi:10.1038/s41467-021-25878-9)
Supplement: Supplementary file 1 — Supplementary Information [file 41467_2021_25878_MOESM1_ESM.pdf]

Supplementary Information

Chiral Brønsted Acid-Controlled Intermolecular Asymmetric [2+2] Photocycloadditions

Evan M. Sherbrook[1], Matthew J. Genzink[1], Bohyun Park[2,3],
Ilia A. Guzei[1], Mu-Hyun Baik*[2,3] and Tehshik P. Yoon*[1]

[1]Department of Chemistry, University of Wisconsin–Madison, 1101 University Avenue, Madison, Wisconsin 53706, United States

[2]Department of Chemistry, Korea Advanced Institute of Science and Technology (KAIST), Daejeon 34141, Republic of Korea

[3]Center for Catalytic Hydrocarbon Functionalizations, Institute for Basic Science (IBS), Daejeon 34141, Republic of Korea

*Corresponding Authors
Mu-Hyun Baik (mbaik2805@kaist.ac.kr)
Tehshik Yoon (tyoon@chem.wisc.edu)

Table of Contents

Supplementary Methods S1

1. **General Information** S1
2. **Catalyst Synthesis** S2
3. **Substrate Synthesis** S5
4. **Asymmetric [2+2] Photocycloaddition Reactions** S11
5. **Racemic [2+2] Photocycloaddition Reactions** S22
6. **Cleavage Reaction of Complex Cycloadduct** S28
7. **Mechanistic Experiments** S29
8. **Assignment of Diastereomers by 1D-NOE** S35
9. **X-Ray Crystallographic Data** S36
10. **Computational Data** S59
11. **NMR Data** S67
12. **HPLC Data** S105

Supplementary References S142

Supplementary Methods

1. General Information

Reagent Preparation: MeCN, THF, CH_2Cl_2, and toluene were purified by elution through alumina as described by Grubbs.[1] Most styrenes, all dienes, phenyl vinyl sulfide, methylenecyclopentane, and methylenecyclobutane were purchased from SigmaAldrich and distilled prior to use. Catalysts **AC-1** and **AC-2** were prepared by established methods for the synthesis of BINOL-derived phosphoric acids[2] and phosphoramidates.[3] Excluding those prepared below, all other starting materials, catalysts, or solvents were used as received from the supplier. Flash-column chromatography was performed with Silicycle 40-63 Å (230-40 mesh) silica. Photochemical reactions were carried out with an H150-blue Kessil Lamp ($\lambda_{em.}$ (max) = ~450 nm).

Product Characterization: Diastereomer ratios for reactions were determined by ^{1}H NMR analysis of unpurified reaction mixtures vs. a phenanthrene internal standard. ^{1}H and ^{13}C NMR data were obtained using a Bruker Avance-500 spectrometer with DCH cryoprobe and are referenced to tetramethylsilane (0.0 ppm) and $CDCl_3$ (77.0 ppm), respectively. This instrument and supporting facilities are funded by Paul J. Bender, Margaret M. Bender, and the University of Wisconsin. ^{1}H, ^{19}F, and ^{31}P NMR data were obtained using Bruker Avance-400 spectrometer. This instrument and supporting facilities are funded by the NSF (CHE-1048642) and the University of Wisconsin. NMR data are reported as follows: chemical shift, multiplicity (s = singlet, d = doublet, t = triplet, q = quartet, p = pentet, sext = sextet, sept = septet, m = multiplet), coupling constant(s) in Hz, integration. NMR spectra were obtained at 298 K unless otherwise noted. FT-IR spectra were obtained using a Bruker Tensor 27 spectrometer and are reported in terms of frequency of absorption (cm^{-1}). Melting points (mp) were obtained using a Stanford Research Systems DigiMelt MPA160 melting point apparatus and are uncorrected. Mass spectrometry was performed with a Thermo Q Exactive™ Plus using ESI-TOF (electrospray ionization-time of flight). This instrument and supporting facilities are funded by the NIH (1S10 OD020022-1) and the University of Wisconsin. Enantiomeric excesses were determined by chiral HPLC of isolated materials using a Waters e2695 separations module with 2998 PDA detector and Daicel CHIRALPAK® columns and HPLC grade *i*-PrOH and hexanes. Traces were acquired using Empower 3® software. Optical rotations were measured using a Rudolf Research Autopol III polarimeter at room temperature in CH_2Cl_2. UV-Vis absorption spectra were acquired using a Varian Cary® 50 UV-visible spectrophotometer with a spectrophotometer.

2. Catalyst Synthesis

(*R*)-(+)-2,2'-Bis(methoxymethoxy)-1,1'-binaphthalene: Reaction performed using a modification of a procedure previously reported by Taylor.[4] A 500 mL round-bottomed flask was charged with 200 mL dry THF, and 3.00 g (75.0 mmol, 2.5 equiv.) of 60% NaH in mineral oil was added. This mixture was cooled to 0 °C in an ice bath. A solution of 8.59 g (30.0 mmol, 1.0 equiv.) (*R*)-BINOL in 550 mL dry THF was added over approximately 20 min *via* cannula, controlling the resulting gas evolution. Then, 5.70 mL (75.0 mmol, 2.5 equiv.) freshly distilled chloromethyl methyl ether (MOMCl) was added portionwise at 0 °C. The reaction was warmed to room temperature and stirred overnight. The reaction was then slowly quenched with 20 mL sat. aq. NH_4Cl. This mixture was transferred to a separatory funnel with 100 mL CH_2Cl_2. After separating the organic layer, the aqueous layer was extracted with 3 x 100 mL CH_2Cl_2. The combined organics were washed with 20 mL brine, dried over Na_2SO_4, and concentrated. The resulting material was recrystallized from CH_2Cl_2/pentanes to give 9.06 g (24.0 mmol, 80% yield) of a crystalline white solid. Spectral data were consistent with previously reported values.[5] ^{1}H NMR (500 MHz, $CDCl_3$) δ 7.95 (d, *J* = 9.0 Hz, 2H), 7.85 (d, *J* = 8.3 Hz, 2H), 7.57 (d, *J* = 9.0 Hz, 2H), 7.34 (ddd, *J* = 8.0, 6.7, 1.2 Hz, 2H), 7.22 (ddd, *J* = 8.0, 6.7, 1.2 Hz, 2H), 7.15 (d, *J* = 8.3 Hz, 2H), 5.08 (d, *J* = 6.9 Hz, 2H), 4.97 (d, *J* = 6.9 Hz, 2H), 3.14 (s, 6H).

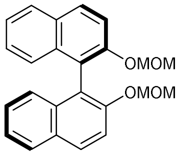

(±)-2,2'-Bis(methoxymethoxy)-1,1'-binaphthalene: Prepared and isolated through the above procedure for (*R*)-(+)-2,2'-Bis(methoxymethoxy)-1,1'-binaphthalene using 1.00 g (25.0 mmol) NaH in 100 mL THF, 2.86 g (10.0 mmol) (±)-BINOL in 25 mL THF, and 1.95 mL (25.0 mmol) MOMCl to give 2.84 g (7.6 mmol, 76% yield) of a white solid. Spectral data were consistent with those reported above.

(*R*)-(+)-3,3'-Dibromo-2,2'-bis(methoxymethoxy)-1,1'-binaphthalene: In a flame-dried 100 mL round-bottomed flask, 3.74 g (10.0 mmol, 1.0 equiv.) (*R*)-(+)-2,2'-bis(methoxymethoxy)-1,1'-binaphthalene was dissolved in 50 mL dry THF and cooled to –78 °C. Then, 12.5 mL (25.0 mmol, 2.5 equiv.) *n*-BuLi (2.0 M in hexanes) was added portionwise. This mixture was warmed to 0 °C for 1 h before returning to –78 °C and adding a solution of 1.28 mL (25.0 mmol, 2.5 equiv.) Br_2 in 10 mL pentanes. The reaction was then warmed to room temperature and stirred overnight. The remaining Br_2 was quenched by stirring with 30 mL sat. aq. Na_2SO_3 for 30 min. The resulting heterogeneous mixture was transferred to a separatory funnel with 30 mL H_2O. After separating the organic layer, the aqueous layer was extracted with 3 x 100 mL CH_2Cl_2. The combined organics were dried over Na_2SO_4, and concentrated. The resulting residue was dry-loaded onto silica and purified by flash column chromatography with a gradient of 2% → 5% → 10% EtOAc in hexanes to give 2.80 g (5.4 mmol, 54%) of a white solid. Spectroscopic data were consistent with previously reported values.[5] ^{1}H NMR (500 MHz, $CDCl_3$) δ 8.27 (s, 2H), 7.80 (d, *J* = 8.0 Hz, 2H), 7.44 (ddd, *J* = 8.0, 6.8, 1.2 Hz, 2H), 7.31 (ddd, *J* = 8.3, 6.8, 1.2 Hz, 2H) , 7.18 (d, *J* = 8.6 Hz, 2H), 4.83 (d, *J* = 5.9, 2H), 4.81(d, *J* = 5.9, 2H), 2.57 (s, 6H).

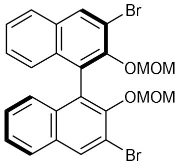

(±)-3,3'-Dibromo-2,2'-bis(methoxymethoxy)-1,1'-binaphthalene: Prepared and isolated through the above procedure for (*R*)-(+)-3,3'-Dibromo-2,2'-bis(methoxymethoxy)-1,1'-binaphthalene using 2.81 g (7.5 mmol) (±)-2,2'-bis(methoxymethoxy)-1,1'-binaphthalene in 50 mL THF, 9.2 mL (18.25 mmol) *n*-BuLi (~2.0 M in hexanes), and 0.93 mL (18.25 mmol) Br_2 in 10 mL pentanes to give 2.08 g (4.0 mmol, 53% yield) of a white solid. Spectral data were consistent with those reported above.

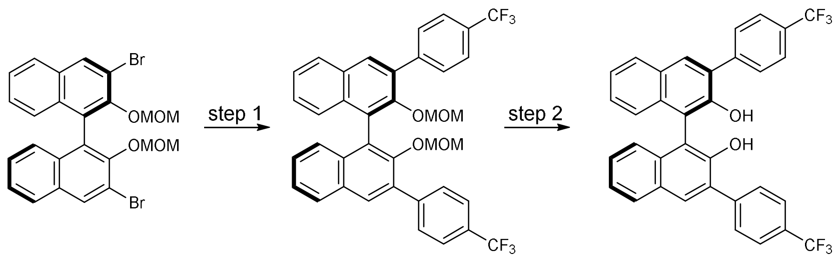

(*R*)-(+)-3,3'-Di-(4-trifluoromethyl)phenyl-[1,1'-binaphthalene]-2,2'-diol: *Step 1:* A 100 mL round-bottomed flask was charged with 2.80 g (5.35 mmol 1.0 equiv.) (*R*)-(+)-3,3'-dibromo-2,2'-bis(methoxymethoxy)-1,1'-binaphthalene, 3.56 g (18.73 mmol, 3.5 equiv.) 4-trifluoromethylphenylboronic acid, 15 mL 2 M aq. Na_2CO_3, and 30 mL 1,2-dimethoxyethane (DME) and fitted with a reflux condenser. The apparatus was purged with N_2 for 15 min before the addition of 340 mg (0.27 mmol, 0.05 equiv.) $Pd(PPh_3)_4$. The mixture was heated to 95 °C for 16 h. After cooling to room temperature, the mixture was filtered over a pad of Celite, the pad washed with EtOAc, and the resulting solution concentrated. This residue was partitioned between 100 mL EtOAc and 25 mL H_2O. After separating the organic layer, the aqueous layer was extracted with 3 x 20 mL EtOAc. The combined organics were dried over Na_2SO_4 and concentrated. *Step 2:* The resulting yellow solid, primarily composed of MOM-protected cross-coupling product, was taken up in 30 mL THF, and 0.8 mL conc. HCl was added. The mixture was heated to 65 °C for 3 h. This mixture was concentrated, and the residue partitioned between 100 mL EtOAc and 25 mL H_2O. After separating the organic layer, the aqueous was extracted with 3 x 50 mL EtOAc. The combined organics were dried over Na_2SO_4, then concentrated directly on silica for purification by flash column chromatography with 2 → 5% EtOAc in hexanes to give 2.67 g (4.65 mmol, 87% yield) of a white solid. Spectroscopic data were consistent with previously reported values.[6] ^{1}H NMR (500 MHz, $CDCl_3$) δ 8.06 (s, 2H), 7.96 (d, J = 8.0 Hz, 2H), 7.87 (d, J = 8.1 Hz, 4H), 7.74 (d, J = 8.2 Hz, 4H), 7.44 (ddd, J = 8.0, 6.8, 1.1 Hz, 2H), 7.37 (ddd, J = 8.2, 6.8, 1.2 Hz, 2H), 7.23 (d, J = 8.2 Hz, 2H), 5.32 (s, 2H).

(±)-3,3'-Di-(4-trifluoromethyl)phenyl-[1,1'-binaphthalene]-2,2'-diol: Prepared and isolated through the above procedure for **(*R*)-(+)-3,3'-di-(4-trifluoromethyl)phenyl-[1,1'-binaphthalene]-2,2'-diol.** *Step 1:* 2.08 g (4.0 mmol) (±)-3,3'-Dibromo-2,2'-bis(methoxymethoxy)-1,1'-binaphthalene, 2.65 (13.9 mmol) 4-trifluoromethylphenylboronic acid, 260 mg $Pd(PPh_3)_4$, 15 mL 2 M aq. Na_2CO_3, and 30 mL 1,2-DME were combined to give a yellow solid. *Step 2*: This yellow solid was combined with 20 mL THF and 0.6 mL conc. HCl to give 1.44 g (2.51 mmol, 63% yield) of a white solid. Spectral data were consistent with those reported above.

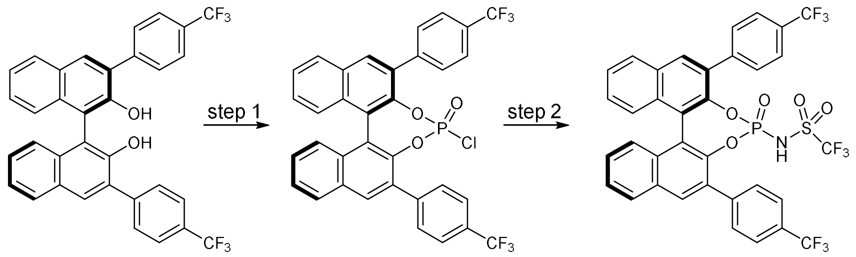

(*R*)-4-Trifluoromethylphenyl-BINOL ((trifluoromethyl)sulfonyl)phosphoramidate ((*R*)-AC-3): Prepared using a modification of a procedure previously reported by Yamamoto.[3] *Step 1:* A 100 mL flame-dried round-bottomed flask was charged with 2.48 g (4.3 mmol, 1.0 equiv.) (*R*)-(+)-3,3'-di-(4-trifluoromethyl)phenyl-[1,1'-binaphthalene]-2,2'-diol and 25 mL dry CH_2Cl_2, then cooled to 0 °C. To this solution was added 0.84 mL (9.0 mmol, 2.1 equiv.) freshly distilled $POCl_3$ and 1.90 mL (13.5 mmol, 3.1 equiv.) freshly distilled Et_3N. The reaction was warmed to room temperature and stirred under N_2 for 16 h. The resulting mixture was washed once with 10 mL H_2O. This aqueous layer was extracted with 2 x 10 mL

CH_2Cl_2. After drying over Na_2SO_4, the solution was concentrated to give a brown solid, which was passed through a short plug of silica with 5% EtOAc in pentanes to give a quantitative yield of white solid. This organic phosphoryl chloride and carried forward to the next step without additional purification. *Step 2:* A 100 mL flame-dried round-bottomed flask was charged with 2.78 g (4.25 mmol, 1.0 equiv) of the organic phosphoryl chloride synthesized above, 634 mg (4.25 mmol, 1.0 equiv.) trifluromethanesulfonamide, 1.04 g (8.50 mmol, 2.0 equiv.) dimethylaminopyridine (DMAP), and 50 mL dry CH_2Cl_2. This mixture was stirred under N_2 at room temperature for 24 h. The crude reaction mixture was washed directly with 20 mL 6 M HCl and dried over Na_2SO_4. The resulting solution was concentrated directly onto silica and purified by flash column chromatography with 10% → 50% → 70% EtOAc in pentanes. The resulting solid was dissolved in CH_2Cl_2, washed with 6 M HCl (3 x 50 mL), dried over Na_2SO_4, filtered, and concentrated to give 1.64 g (2.14 mmol, 50% yield) of a white crystalline solid. (dec. pt. = 250+ °C). $[\alpha]_D^{22}$ –234.0° (c0.970, CH_2Cl_2). ^{1}H NMR (500 MHz, $CDCl_3$) δ 8.13 (s, 1H), 8.05 (d, J = 8.0 Hz, 1H), 8.04 (s, 1H), 8.02 (d, J = 8.0 Hz, 1H), 7.76 (d, J = 8.1 Hz, 2H), 7.64–7.60 (m, 7H), 7.56 (ddd, J = 8.0, 6.3, 1.4 Hz, 1H), 7.48 (d, J = 8.3 Hz, 1H), 7.45–7.35 (m, 3H), 6.35 (br s, 1H). ^{13}C NMR (125 MHz, $CDCl_3$) δ 142.76, 142.66, 142.41, 142.33, 139.49, 139.20, 132.47, 132.45, 132.19, 132.18, 132.14, 132.02, 132.00, 131.95, 131.91, 131.82, 130.48, 130.22, 130.02, 129.96, 129.70, 128.85, 128.64, 127.49, 127.39, 127.33, 127.10, 127.02, 126.93, 126.85, 125.39, 125.36, 125.33, 125.30, 125.16, 124.96, 124.94, 124.91, 124.88, 124.85, 123.00, 122.78, 122.76, 122.68, 122.33, 121.95, 121.93, 120.84, 120.52, 119.78, 117.22, 114.67. More signals appear in this list than expected from carbons alone due to both C-F and C-P coupling. Due to signal overlap it was not possible to fully deconvolute the splitting patterns that give rise to these individual signals. I ^{19}F NMR (377 MHz, $CDCl_3$) δ –62.92, –63.37, –78.32. ^{31}P NMR (162 MHz, $CDCl_3$) δ –5.05. HRMS (ESI) calculated for $[C_{35}H_{23}F_9N_2O_5PS]^+$ ($M+NH_4^+$) requires *m/z* 785.0916, found 785.0918.

(±)-4-Trifluoromethylphenyl-BINOL ((trifluoromethyl)sulfonyl)phosphoramidate ((±)-AC-3): Prepared and isolated through the above procedure for ***(R)*-A1**. *Step 1:* 1.43 g (2.5 mmol) (±)-3,3'-Di-(4-trifluoromethyl)phenyl-[1,1'-binaphthalene]-2,2'-diol, 0.47 mL (5.0 mmol) $POCl_3$, and 1.03 mL (7.5 mmol) Et_3N were combined to give a white solid. *Step 2*: This white solid (1.47 g, 2.25 mmol) was combined with 335 mg (2.25 mmol) trifluoromethanesulfonamide, 550 mg (4.50 mmol) DMAP, and 25 mL CH_2Cl_2 to give 0.87 g (1.13 mmol, 50% yield. Spectral data were consistent with those reported above.

Diethyl ((trifluoromethyl)sulfonyl)phosphoramidate (AC-4): A flame-dried 250 mL round-bottomed flask was charged with 0.89 g (6.0 mmol, 1.0 equiv.) trifluoromethylsulfonamide and 1.48 g (12.0 mmol, 2.0 equiv.) 4-dimethylaminopyridine in 50 mL CH_2Cl_2. To this solution, 0.87 mL (6.0 mmol, 1.0 equiv.) diethyl chlorophosphate was added dropwise at room temperature and the reaction was stirred for 24 h. The reaction mixture was washed with 6 x 30 mL 6 M HCl then dried over Na_2SO_4, filtered, and concentrated. Spectral data were consistent with previously reported values.[7] ^{1}H NMR (500 MHz, $CDCl_3$) δ 7.30 (s, 1H), 4.28 (m, 4H), 1.41 (t, J = 7.1 Hz, 6H).

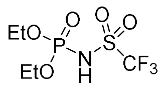

3. Substrate Synthesis

2-Acetyl-1-methylimidazole: A solution of 0.84 mL (10.5 mmol, 1.05 equiv.) 1-methylimidazole in 25 mL dry THF was prepared in a flame-dried 100 mL round-bottomed flask. After cooling to –78 °C, 5.5 mL (10.5 mmol, 1.05 equiv.) *n*-BuLi (1.91 M in hexanes) was added portionwise. The reaction was warmed to 0 °C for 30 min before returning to –78 °C. A solution of 1.15 mL (10.0 mmol, 1.0 equiv.) 4-acetylmorpholine in 25 mL THF was prepared in a flame-dried 50 mL pear-shaped flask and added to the solution of deprotonated 1-methylimidazole *via* cannula. The reaction was warmed to room temperature and stirred overnight. The resulting solution was stirred vigorously, then 2 mL glacial acetic acid added dropwise. This solution was transferred to separatory funnel with 100 mL EtOAc, then washed with 30 mL sat. aq. $NaHCO_3$ and 30 mL sat. aq. NaCl. Each wash solution was back-extracted with 30 mL additional EtOAc. The combined organics were dried over Na_2SO_4 and concentrated. The product was purified by flash column chromatography with 2:3 EtOAc/pentanes to give 700 mg (5.6 mmol, 56% yield) of a colorless oil. Spectral data were consistent with previously reported values.[8] ^{1}H NMR (400 MHz, $CDCl_3$) δ 7.14 (s, 1H), 7.03 (s, 1H), 4.00 (s, 3H), 2.66 (s, 3H).

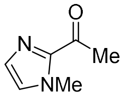

***N*-Methoxy-*N*-methylcinnamide:** A flame-dried 50 mL round-bottomed flask was charged with 8.64 g (52.0 mmol, 1.0 equiv.) cinnamoyl chloride and 5.34 g (54.7 mmol, 1.05 mmol) *N,O*-dimethylhydroxylamine hydrochloride in 150 mL dry CH_2Cl_2. The mixture was cooled to 0 °C, and 9.20 mL (114.4, 2.2 equiv.) pyridine was added slowly. The reaction was then warmed to room temperature and stirred overnight. Then 1 M HCl was added, the organic layer separated, and the aqueous layer extracted with 3 x 25 mL additional EtOAc. The combined organics were washed with 25 mL sat. aq. $NaHCO_3$ and 25 mL sat. aq. NaCl. The combined organics were dried over Na_2SO_4 and concentrated. The products were purified by flash column chromatography with 1:1 Et_2O/pentanes to give 8.14 g (42.6 mmol, 82% yield) of a viscous oil that solidified after extended drying *in vacuo*. Spectral data were consistent with previously reported values.[9] ^{1}H NMR (400 MHz, $CDCl_3$) δ 7.74 (d, J = 15.9 Hz, 1H), 7.59–7.56 (m, 2H), 7.41–7.36 (m, 3H), 7.04 (d, J = 15.9 Hz, 1H), 3.77(s, 3H), 3.32 (s, 3H).

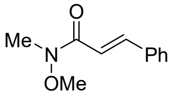

2-Cinnamoyl-1-methyl-1*H*-imidazole (1): A flame-dried 100 mL round-bottomed flask was charged with 0.71 mL (8.93 mmol, 1.05 equiv.) *N*-methylimidazole and 30 mL dry THF, then cooled to –78 °C. A volume of 3.6 mL (8.93 mmol, 1.05 equiv.) *n*-BuLi (2.50 M in hexanes) was added portionwise. The reaction was warmed to 0 °C for 30 min, then returned to –78 °C. A solution of 1.62 g (8.50 mmol, 1.0 equiv.) *N*-methoxy-*N*-methylcinnamide in 10 mL THF was prepared in a flame-dried pear-shaped flask and added to the solution of deprotonated 1-methylimidazole *via* cannula. The reaction was warmed to room temperature and stirred overnight. The resulting solution was stirred vigorously, then 2 mL glacial acetic acid was added dropwise. This solution was transferred to separatory funnel with 50 mL EtOAc and shaken with 20 mL water. The organic layer was separated and the aqueous extracted with 3 x 25 mL additional EtOAc. The combined organics were then washed with 30 mL sat. aq. $NaHCO_3$ and 30 mL sat. aq. NaCl, dried over Na_2SO_4, and concentrated. The products were purified by flash column chromatography with 1:1 Et_2O/pentanes. Yield: 1.32 g (6.2 mmol, 73% yield) of an off-white solid. Spectral data were consistent with previously reported values.[10] ^{1}H NMR (400 MHz, $CDCl_3$) δ 8.08 (d, J = 16.0 Hz, 1H), 7.83 (d, J = 16.0 Hz, 1H), 7.72–7.68 (m, 2H), 7.43–7.38 (m, 3H), 7.23 (s, 1H), 7.09 (s, 1H), 4.10 (s, 3H).

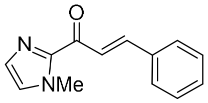

General Method for the Preparation of Substituted Cinnamoyl Methylimidazole Derivatives: A round-bottomed flask was charged with 2-acetyl-1-methylimidazole (1.0 equiv.), and a mixture of EtOH and H_2O. This solution was sparged briefly with N_2 (5 min). Aromatic aldehyde (1.0–1.1 equiv., freshly distilled if possible) was then added to the solution, followed by a catalytic quantity of KOH. The reaction was then stirred under N_2 for 12–16 h (overnight). *Work-up 1*: If the resulting solution was hetereogeneous, the product was filtered and washed with H_2O and cold EtOH to give the pure desired product. *Work-up 2*: If the resulting solution was homogeneous, the crude reaction mixture was diluted with CH_2Cl_2, transferred to separatory funnel, and shaken with H_2O. The organic layer was separated and the aqueous extracted with

three portions of CH_2Cl_2. The combined organics were dried over Na_2SO_4, concentrated, and purified by flash column chromatography.

(*E*)-3-(4-Methoxyphenyl)-1-(1-methyl-1*H*-imidazol-2-yl)prop-2-en-1-one: Prepared using the general method above using 372.7 mg (3.0 mmol, 1.0 equiv.) 2-acetyl-1-methylimidazole, 0.26 mL (3.0 mmol, 1.0 equiv.) 4-methoxybenzaldehyde, ~25 mg (½ pellet) KOH, 6 mL EtOH, and 3 mL H_2O. Work-up 2, then purified by flash column chromatography with 1:1 Et_2O/pentanes to give 382 mg (1.58 mmol, 53% yield) of an off-white solid. Spectral data were consistent with previously reported values.[11] ^{1}H NMR (400 MHz, $CDCl_3$) δ 7.96 (d, *J* = 15.9 Hz, 1H), 7.80 (d, *J* = 15.9 Hz, 1H), 7.66 (d, *J* = 8.7 Hz, 1H), 7.21 (s, 1H), 7.07 (s, 1H), 6.92 (d, *J* = 8.7 Hz, 1H), 4.10 (s, 3H), 3.85 (s, 3H).

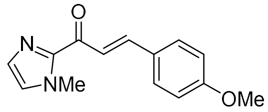

(*E*)-3-(2-Methoxyphenyl)-1-(1-methyl-1H-imidazol-2-yl)prop-2-en-1-one: Prepared using general method above using 499.6 mg (4.0 mmol, 1.0 equiv.) 2-acetyl-1-methylimidazole, 0.41 mL (4.4 mmol, 1.1 equiv.) 2-methoxybenzaldehyde, ~25 mg (½ pellet) KOH, 8 mL EtOH. Work-up 2, then purified by flash column chromatography with 1:1 Et_2O/pentanes to give 877 mg (3.6 mmol, 90% yield) of an off-white solid. Spectral data were consistent with previously reported values.[12] ^{1}H NMR (400 MHz, $CDCl_3$) δ 8.24 (d, *J* = 16.2 Hz, 1H), 8.09 (d, *J* = 16.2 Hz, 1H), 7.76 (dd, *J* = 7.6, 1.6 Hz, 1H), 7.37 (ddd, *J* = 8.9, 7.6, 1.6 Hz, 1H), 7.21 (s, 1H), 7.07 (s, 1H), 6.97 (t, *J* = 7.6 Hz, 1H), 6.93 (t, *J* = 8.9 Hz, 1H), 4.10 (s, 3H), 3.91 (s, 3H).

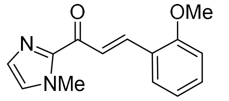

Methyl 4-[(1*E*)-3-(1-methyl-1*H*-imidazol-2-yl)-3-oxoprop-1-en-1-yl]benzoate (4a): Prepared using the general method above using 416.0 mg (3.3 mmol, 1.0 equiv.) 2-acetyl-1-methylimidazole, 594 mg (3.6 mmol, 1.1 equiv.) methyl 4-formylbenzoate, ~25 mg (1/2 pellet) KOH, 6 mL MeOH[13], and 3 mL H_2O. Work-up 1 to give 428 mg (1.58 mmol, 48% yield) of an off-white solid (mp = 138–140 °C). ^{1}H NMR (400 MHz, $CDCl_3$) δ 8.15 (d, *J* = 16.0 Hz, 1H), 8.06 (d, *J* = 8.3 Hz, 2H), 7.82 (d, *J* = 16.0 Hz, 1H), 7.75 (d, *J* = 8.3 Hz), 7.24 (s, 1H), 7.11 (s, 1H), 4.11 (s, 3H), 3.94 (s, 3H). ^{13}C NMR (125 MHz, $CDCl_3$) δ 180.10, 166.54, 143.94, 141.72, 139.19, 131.39, 130.06, 129.56, 128.51, 127.54, 125.02, 52.28, 36.40. HRMS (ESI) calculated for $[C_{15}H_{15}N_2O_3]^+$ ($M+H^+$) requires *m/z* 271.1077, found 271.1080.

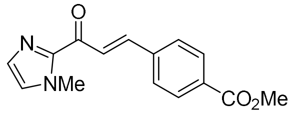

(*E*)-1-(1-Methyl-1*H*-imidazol-2-yl)-3-(4-trifluoromethylphenyl)prop-2-en-1-one: Prepared using the general method above using 328.0 mg (3.0 mmol, 1.0 equiv.) 2-acetyl-1-methylimidazole, 0.45 mL (3.3 mmol, 1.1 equiv.) 4-trifluoromethylbenzaldehyde, ~25 mg (1/2 pellet) KOH, 12 mL EtOH, and 6 mL H_2O. Work-up 2, then purified by flash column chromatography with 1:1 Et_2O/pentanes to give 442 mg (1.58 mmol, 53% yield) of an off-white solid. Spectral data were consistent with previously reported values.[12] ^{1}H NMR (400 MHz, $CDCl_3$) δ 8.14 (d, *J* = 16.0 Hz, 1H), 7.81 (d, *J* = 16.0 Hz, 1H), 7.79 (d, *J* = 8.3 Hz, 2H), 7.65 (d, *J* = 8.3 Hz, 2H), 7.24 (s, 1H), 7.11 (s, 1H), 4.11 (s, 3H).

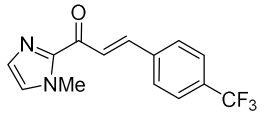

(*E*)-4-(3-(1-Methyl-1H-imidazol-2-yl)-3-oxoprop-1-en-1-yl)benzonitrile (6a): Prepared using the general method above using 418.0 mg (3.4 mmol, 1.0 equiv.) 2-acetyl-1-methylimidazole, 476.0 mg (3.6 mmol, 1.1 equiv.) 4-formylbenzonitrile, ~25 mg (1/2 pellet) KOH, 4 mL EtOH, and 2 mL H_2O. Work-up 1 to give 552.0 mg (2.31 mmol, 70% yield) of an off-white solid (mp = 228–232 °C). ^{1}H NMR (500 MHz, $CDCl_3$) δ 8.15 (d, J = 16.1 Hz, 1H), 7.79-7.76 (m, 3H), 7.69 (d, J = 8.3 Hz, 2H), 7.24 (s, 1H), 7.12 (s, 1H), 4.11 (s, 3H). ^{13}C NMR (125 MHz, $CDCl_3$) δ 179.72, 143.81, 140.53, 139.30, 132.59, 129.71, 128.92, 127.72, 126.14, 118.48, 113.35, 36.38. HRMS (ESI) calculated for $[C_{14}H_{11}N_3O]^+$ ($M+H^+$) requires *m/z* 238.0975, found 238.097.

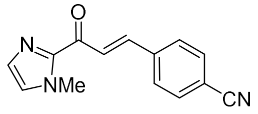

(*E*)-3-(2-Fluorophenyl)-1-(1-methyl-1H-imidazol-2-yl)prop-2-en-1-one (7a): Prepared using the general method above using 258.0 mg (2.1 mmol, 1.0 equiv.) 2-acetyl-1-methylimidazole, 0.23 mL (2.2 mmol, 1.1 equiv.) 2-fluorobenzaldehyde, ~25 mg (1/2 pellet) KOH, 8 mL EtOH, and 4 mL H_2O. Work-up 1 to give 270 mg (1.17 mmol, 56% yield) of a white solid (mp = 136–138 °C). ^1H NMR (400 MHz, $CDCl_3$) δ 8.13 (d, *J* = 16.2 Hz, 1H), 8.02 (d, *J* = 16.2 Hz, 1H), 7.77 (td, *J* = 7.6, 1.7 Hz, 1H), 7.39-7.35 (m, 1H), 7.23 (d, *J* = 0.9 Hz, 1H), 7.17 (t, *J* = 7.6 Hz, 1H), 7.13-7.09 (m, 2H), 4.10 (s, 3H). ^{13}C NMR (125 MHz, $CDCl_3$) δ 180.36, 161.73, 144.02, 135.45, 131.85, 129.46, 128.91, 127.35, 124.84, 124.35, 123.10, 116.14, 36.37. ^{19}F NMR (377 MHz, $CDCl_3$) δ –114.39. HRMS (ESI) calculated for $[C_{13}H_{11}FN_2O]^+$ ($M+H^+$) requires *m/z* 231.0928, found 231.0927.

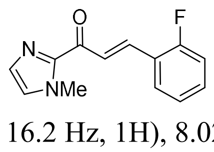

(*E*)-3-(4-Chlorophenyl)-1-(1-methyl-1H-imidazol-2-yl)prop-2-en-1-one: Prepared using the general method above using 248.2 mg (2.0 mmol, 1.0 equiv.) 2-acetyl-1-methylimidazole, 315.0 mg (2.2 mmol, 1.1 equiv.) 4-chlorobenzaldehyde, ~25 mg (1/2 pellet) KOH, 8 mL EtOH, and 4 mL H_2O. Work-up 1 to give 340.1 mg (1.38 mmol, 69% yield) of a white solid. Spectral data were consistent with previously reported values.[11] ^1H NMR (500 MHz, $CDCl_3$) δ 8.04 (d, *J* = 16.0 Hz, 1H), 7.76 (d, *J* = 16.0 Hz, 1H), 7.62 (d, *J* = 8.5 Hz, 2H), 7.37 (d, *J* = 8.5 Hz, 2H), 7.22 (s, 1H), 7.09 (s, 1H), 4.10 (s, 3H).

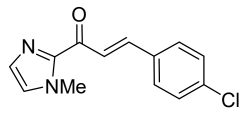

(*E*)-1-(1-Methyl-1H-imidazol-2-yl)-3-(p-tolyl)prop-2-en-1-one: Prepared using the general method above using 248.0 mg (2.0 mmol, 1.0 equiv.) 2-acetyl-1-methylimidazole, 0.26 mL (2.2 mmol, 1.1 equiv.) *p*-tolualdehyde, ~25 mg (1/2 pellet) KOH, 8 mL EtOH, and 4 mL H_2O. Work-up 2, then purified by flash column chromatography with 3:7 EtOAc/hexanes to give 323.3 mg (1.43 mmol, 71% yield) of a white solid. Spectral data were consistent with previously reported values.[11] ^1H NMR (500 MHz, $CDCl_3$) δ 8.03 (d, *J* = 16.0, 1H), 7.81 (d, *J* = 16.0 Hz, 1H), 7.60 (d, *J* = 8.1 Hz, 2H), 7.22 (m, 2H), 7.20 (s, 1H), 7.07 (s, 1H), 4.10 (s, 3H), 2.38 (s, 3H).

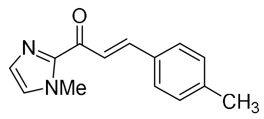

(*E*)-1-(1-Methyl-1H-imidazol-2-yl)-3-(naphthalen-2-yl)prop-2-en-1-one (10a): Prepared using the general method above using 370 mg (3.0 mmol, 1.0 equiv.) 2-acetyl-1-methylimidazole, 0.52 mg (3.3 mmol, 1.1 equiv.) 2-napthaldehyde, ~25 mg (1/2 pellet) KOH, 6 mL EtOH, and 3 mL H_2O. Work-up 1 to give 610 mg (2.32 mmol, 78% yield) of an off-white solid (mp = 153–155 °C). ^1H NMR (500 MHz, $CDCl_3$) δ 8.20 (d, *J* = 15.9 Hz, 1H), 8.09 (s, 1H), 8.00 (d, *J* = 16.0 Hz, 1H), 7.89-7.83 (m, 4H), 7.53-7.51 (m, 2H), 7.25 (s, 1H), 7.10 (s, 1H), 4.13 (s, 3H). ^{13}C NMR (125 MHz, $CDCl_3$) δ 180.47, 144.12, 143.47, 134.40, 133.35, 132.50, 130.58, 129.36, 128.66, 128.58, 127.78, 127.29, 127.27, 126.63, 124.31, 122.97, 36.41. HRMS (ESI) calculated for $[C_{17}H_{14}N_2O]^+$ ($M+H^+$) requires *m/z* 263.1179, found 263.1177.

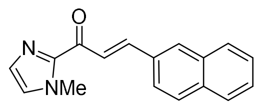

(*E*)-1-(1-Methyl-1H-imidazol-2-yl)-3-(thiophen-2-yl)prop-2-en-1-one: Prepared using the general method above using 250.0 mg (2.0 mmol, 1.0 equiv.) 2-acetyl-1-methylimidazole, 0.21 mL (2.2 mmol, 1.1 equiv.) 2-thiophenecarboxaldehyde, ~25 mg (1/2 pellet) KOH, 8 mL EtOH, and 4 mL H_2O. Work-up 2, then purified by flash column chromatography with 4:6 EtOAc/hexanes to give 331.0 mg (1.52 mmol, 75% yield) of an off-white solid. Spectral data were consistent with previously reported values.[10] ^1H NMR (400 MHz, $CDCl_3$) δ 7.95 (d, *J* = 15.7 Hz, 1H), 7.84 (d, *J* = 15.7 Hz, 1H), 7.42 (d, *J* = 5.0 Hz, 1H), 7.38 (d, *J* = 3.5 Hz, 1H), 7.22 (s, 1H), 7.08 (s, 1H), 7.08 (dd, *J* = 5.0, 3.5 Hz, 1H), 4.09 (s, 3H).

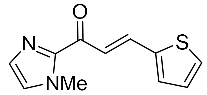

(*E*)-1-(1-Methyl-1H-imidazol-2-yl)-3-(3-nitrophenyl)prop-2-en-1-one (12a): Prepared using the general method above using 248.7 mg (2.0 mmol, 1.0 equiv.) 2-acetyl-1-methylimidazole, 331.4 mg (2.2 mmol, 1.1 equiv.) 3-nitrobenzaldehyde, ~25 mg (1/2 pellet) KOH, 8 mL EtOH, and 4 mL H_2O. Work-up 1 to give 420.3 mg (1.63 mmol, 82% yield) of a white solid. (mp = 161–162 °C). ^1H NMR (500 MHz, $CDCl_3$) δ 8.53 (t, *J* = 2.1 Hz, 1H), 8.25-8.23 (m, 1H), 8.18 (d, *J* = 16.0 Hz, 1H), 7.98 (d, *J* = 7.9 Hz, 1H), 7.83 (d, *J* = 16.0 Hz, 1H),

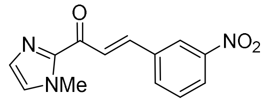

7.59 (t, J = 7.9 Hz, 1H), 7.25 (s, 1H), 7.12 (s, 1H), 4.11 (s, 3H). ^{13}C NMR (125 MHz, $CDCl_3$) δ 179.71, 148.76, 143.78, 140.16, 136.77, 134.10, 129.86, 129.71, 127.72, 125.70, 124.57, 123.01, 36.39. HRMS (ESI) calculated for $[C_{13}H_{11}N_3O_3]^+$ ($M+H^+$) requires *m/z* 258.0873, found 258.0872.

(*E*)-3-(2-Iodophenyl)-1-(1-methyl-1H-imidazol-2-yl)prop-2-en-1-one: Prepared using the general method above using 247.5 mg (2.0 mmol, 1.0 equiv.) 2-acetyl-1-methylimidazole, 515.0 mL (2.2 mmol, 1.1 equiv.) 2-iodobenzaldehyde, ~25 mg (1/2 pellet) KOH, 8 mL EtOH, and 4 mL H_2O. Work-up 1 to give 508 mg (1.50 mmol, 75% yield) of an off-white solid. Spectral data were consistent with previously reported values.[14] ^{1}H NMR (500 MHz, $CDCl_3$) δ 8.05 (d, J = 15.8 Hz, 1H), 7.97 (d, J = 15.8 Hz, 1H), 7.91 (dd, J = 8.0, 1.2 Hz, 1H), 7.82 (dd, J = 7.9, 1.6 Hz, 1H), 7.37 (t, J = 8.0 Hz, 1H), 7.22 (d, J = 0.9 Hz, 1H), 7.10 (s, 1H), 7.06 (td, J = 7.7, 1.6 Hz, 1H), 4.11 (s, 3H).

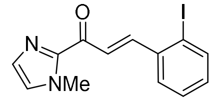

(*E*)-3-(7-Methoxy-2*H*-1,3-benzodioxol-5-yl)-1-(1-methyl-1*H*-imidazol-2-yl)prop-2-en-1-one (36a): Prepared using the general method above using 250.0.0 mg (2.0 mmol, 1.0 equiv.) 2-acetyl-1-methylimidazole, 360 mg (2.0 mmol, 1.0 equiv.) 5-methoxypiperonal, ~25 mg (1/2 pellet) KOH, 9 mL EtOH, and 1 mL H_2O. Work-up 1 to give 480 mg (1.68 mmol, 84% yield) of a yellow solid (mp = 210–212 °C). ^{1}H NMR (400 MHz, $CDCl_3$) δ 7.91 (d, J = 15.8 Hz, 1H), 7.71 (d, J = 15.8 Hz, 1H), 7.21 (d, J = 0.8 Hz, 1H), 7.08 (d, J = 0.8 Hz, 1H), 6.92 (d, J = 1.4 Hz, 1H), 6.88 (d, J = 1.4 Hz, 1H), 6.03 (s, 2H), 4.10 (s, 3H), 3.95 (s, 3H). ^{13}C NMR (125 MHz, $CDCl_3$) δ 180.37, 149.29, 144.10, 143.77, 143.41, 137.70, 129.83, 129.25, 127.22, 121.30, 109.31, 102.46, 102.05, 56.67, 36.41. HRMS (ESI) calculated for $[C_{15}H_{15}N_2O_4]^+$ ($M+H^+$) requires *m/z* 287.1026, found 287.1027.

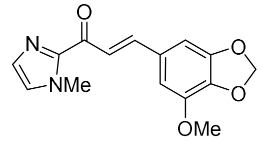

1-(Acetoxymethyl)-4-vinylbenzene: To a solution of pyridine (0.5 mL) and acetic anhydride (0.5 mL) in a flame-dried 50 mL round-bottomed flask was added 1.07 g (8.0 mmol, 1.0 equiv) (4-vinylphenyl)methanol and 98 mg (0.8 mmol, 0.1 equiv.) 4-dimethylaminopyridine. The reaction was stirred at room temperature for 20 h, then diluted with water (30 mL) and extracted with EtOAc (3 x 30 mL). The combined organic layers were washed with 1M HCl (30 mL) and sat. aq. $NaHCO_3$ (30 mL). The organic layers were dried over Na_2SO_4, filtered, and concentrated. The residue was purified by flash column chromatography with a gradient of 0% Et_2O in pentanes → 10% Et_2O in pentanes to give 1.35 g (7.7 mmol, 96%) of a colorless oil. Spectroscopic data were consistent with that previously reported.[15] ^{1}H NMR (500 MHz, $CDCl_3$) δ 7.41 (d, J = 8.3 Hz, 2H), 7.32 (d, J = 8.3 Hz, 2H), 6.72 (dd, J = 17.8, 11.0 Hz, 1H), 5.77 (dd, J = 17.8, 0.8 Hz, 1H), 5.27 (dd, J = 11.0, 0.8 Hz 1H), 5.10 (s, 2H), 2.11 (s, 3H).

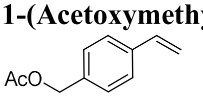

***tert*-Butyldimethyl(4-vinylbenzyloxy) silane:** A vial was charged with CH_2Cl_2 (6 mL) and 0.39 g (2.87 mmol, 1.0 equiv.) (4-vinylphenyl)methanol followed by 0.25 g (3.73 mmol, 1.3 equiv.) imidazole. The reaction was stirred until everything dissolved then 0.52 g (3.44 mmol, 1.2 equiv.) *tert*-butyldimethylsilyl chloride was added and the reaction was stirred at room temperature for 6 h. The reaction was diluted in sat. aq. NH_4Cl and extracted with CH_2Cl_2 (3 x 10 mL). The organic layers were combined, dried over Na_2SO_4, filtered, and concentrated. The residue was purified with flash column chromatography eluting with a gradient of 0% Et_2O in pentanes → 2:1 pentanes:Et_2O. Spectral data were consistent with previously reported values.[16] ^{1}H NMR (500 MHz, $CDCl_3$) δ 7.38 (AA'BB', J = 8.1 Hz, 2H), 7.28 (AA'BB', J = 8.1 Hz, 2H), 6.71 (dd, J = 17.6, 10.9 Hz, 1H), 5.72 (d, J = 17.6 Hz, 1H), 5.21 (d, J = 10.9 Hz, 1H), 4.73 (s, 2H), 0.94 (s, 9H), 0.10 (s, 6H).

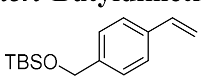

1-(Azidomethyl)-4-vinylbenzene: A flame-dried 250 mL round-bottomed flask was charged with 0.65 g (10.0 mmol, 2 equiv.) sodium azide and 0.76 g (5.0 mmol, 1.0 equiv.) 4-vinylbenzyl chloride dissolved in 50 mL DMF. The mixture was stirred for 24 h at room temperature. The reaction was diluted with water (100 mL) and extracted with Et_2O (3 x 50 mL). The organic layers were combined and washed with water (2 x 50 mL) and brine (50 mL). The organic layer

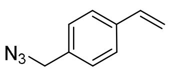

was dried over Na_2SO_4, filtered, and concentrated. The residue was purified by flash column chromatography eluting from 1:9 EtOAc:hexanes to give 0.75 g (4.7 mmol, 93%) of a light yellow oil. Spectral data were consistent with previously reported values.[17] ^{1}H NMR (500 MHz, $CDCl_3$) δ 7.43 (AA'BB', *J* = 8.1 Hz, 2H), 7.28 (AA'BB', *J* = 8.1 Hz, 2H), 6.72 (dd, J = 17.7, 10.9 Hz, 1H), 5.77 (dd, *J* = 17.7, 0.7 Hz, 1H), 5.28 (dd, *J* = 10.9, 0.7 Hz, 1H), 4.33 (s, 2H).

***tert*-Butyl 4-vinylbenzylcarbamate:** A 25 mL round-bottomed flask was charged with 0.78 g (4.9 mmol, 1.0 equiv.) 1-azidomethyl-4-vinyl-benzene, 2.52 g (9.8 mmol, 2.0 equiv.) triphenylphosphine, and 10:1 THF:H_2O (11 mL). The reaction was stirred at room temperature for 24 h, then dissolved in CH_2Cl_2 (20 mL) and extracted with 1 N HCl (2 x 20 mL). The acidic aqueous phases were combined, washed with EtOAc (3 x 20 mL) and adjusted to pH 10 by addition of 3 N NaOH. The alkaline solution was then extracted with CH_2Cl_2 (3 x 50 mL), and the combined organics were dried over Na_2SO_4 and concentrated in vacuo to give the desired amine, which was carried on without further purification. A 250 mL round bottom flask was charged with the crude amine as a solution in CH_2Cl_2 (100 mL), 1.55 g (7.3 mmol, 1.5 equiv.) di-*tert*-butyl dicarbonate, 0.82 mL (5.9 mmol, 1.2 equiv.) NEt_3, and 7 mL MeOH. The resulting solution was then stirred at room temperature overnight under an atmosphere of N_2. The reaction was concentrated, and the residue was purified by flash column chromatography eluting from 20% EtOAc in hexanes to give 0.74 g (3.2 mmol, 65% over two steps) of a colorless oil. Spectral data were consistent with previously reported values.[18] ^{1}H NMR (500 MHz, $CDCl_3$) δ 7.38 (d, *J* = 9.0 Hz, 2 H), 7.26 (d, *J* = 9.0 Hz, 2 H), 6.72 (dd, *J* = 10.9 Hz, 17.6 Hz 1H), 5.75 (dd, *J* = 0.9 Hz, 17.6 Hz, 1 H), 5.25 (dd, *J* = 0.9 Hz, 10.9 Hz, 1 H), 4.31 (d, *J* = 5.0 Hz, 2 H), 1.48 (s, 9 H).

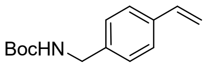

5-Vinyl-1-benzofuran: A flame-dried 25 mL round-bottomed flask was charged with 4.15 g (11.5 mmol, 1.4 equiv.) methyltriphenylphosphonium bromide in 8 mL dry THF and treated with 1.38 g (12.3 mmol, 1.5 equiv.) potassium *tert*-butoxide. The mixture was stirred for 30 min at room temperature before a solution of 1.20 g (8.2 mmol, 1.0 equiv.) 1-benzofuran-5-carbaldehyde in 9 mL THF was added dropwise. The reaction was stirred for 14 h, then diluted with water (50 mL) and extracted with EtOAc (3 x 10 mL). The combined organic layers were dried over Na_2SO_4, filtered, and concentrated. The crude product was purified by flash column chromatography eluting with hexanes to give 0.89 g (6.2 mmol, 75%) of a colorless oil. Spectral data were consistent with previously reported values.[19] ^{1}H NMR (500 MHz, $CDCl_3$) δ 7.62-7.61 (m, 2H), 7.46-7.38 (m, 2H), 6.82 (dd, *J* = 17.6, 10.8 Hz, 1H), 6.75 (dd, *J* = 2.0, 1.2 Hz, 1H), 5.73 (d, *J* = 17.6 Hz, 1H), 5.22 (d, *J* = 10.8 Hz, 1H).

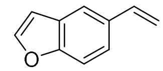

1-Toluenesulfonyl-5-vinyl-1H-indole: A solution of 0.96 g (6.5 mmol, 1.1 equiv.) potassium vinyltrifluoroborate, 23 mg (0.13 mmol, 0.02 equiv.) $PdCl_2$, 103 mg (0.39 mmol, 0.06 equiv.) triphenylphosphine, 6.3 g (19.5 mmol, 3.0 equiv.), Cs_2CO_3, and 2.27 g (6.5 mmol, 1.0 equiv.) 5-bromo-1-tosyl-1H-indole in THF/H_2O (9:1) (13 mL) was stirred at 85 °C for 22 h under a N_2 atmosphere in a sealed tube. After cooling to room temperature, the reaction was diluted with H_2O (20 mL) and extracted with CH_2Cl_2 (3 x 50 mL). The combined organic layers were dried over Na_2SO_4, filtered, and concentrated. The crude product was purified by flash column chromatography eluting from 10% EtOAc in hexanes to give 1.17 g (3.9 mmol, 61%) of a colorless oil. Spectral data were consistent with previously reported values.[20] ^{1}H NMR (500 MHz, $CDCl_3$) δ 7.93 (d, J = 8.7 Hz, 1H), 7.74 (d, J = 8.3 Hz, 1H), 7.54-7.52 (m, 2H), 7.40 (dd, J = 8.7, 1.8 Hz, 1H), 7.21 (d, J = 8.1 Hz, 1H), 6.76 (dd, J = 17.6, 10.9 Hz, 1H), 6.63 (d, J = 3.7 Hz, 1H), 5.71 (d, J = 17.5 Hz, 1H), 5.21 (d, J = 10.9 Hz, 1H), 2.33 (s, 3H)

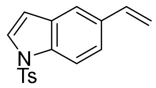

1-Bromo-2,5-dimethoxy-4-vinylbenzene: A flame-dried 100 mL round-bottomed flask was charged with 1.57 g (4.4 mmol, 1.1 equiv.) methyltriphenylphosphonium bromide in 40 mL dry THF. The vessel was cooled to 0 °C, and 500 mg of potassium *tert*-butoxide was added. The mixture was stirred for 30 min before cooling to –78 °C, at which point 0.98 g of 4-bromo-2,5-dimethoxybenzaldehyde in 5 mL THF was added portionwise. The mixture was warmed to room

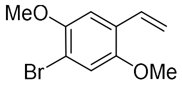

temperature and stirred for 18 h. The reaction mixture was then diluted with 50 mL 1:1 Et_2O/pentanes and filtered over a plug of Celite. The eluent was concentrated directly onto silica, then purified by flash column chromatography with 10% Et_2O in pentanes to give 710 mg (2.90 mmol, 66% yield) of a white solid. Spectral data were consistent with previously reported values.[21] ^1H NMR (500 MHz, $CDCl_3$) δ 7.90 (d, J = 15.9 Hz, 1H), 7.71 (d, J = 15.9 Hz, 1H), 7.26 (s, 1H), 7.08 (s, 1H), 6.92 (d, J = 1.3 Hz, 1H), 6.88 (d, J = 1.3 Hz, 1H), 6.03 (s, 2H), 4.10 (s, 3H), 3.95 (s, 3H).

4. Asymmetric [2+2] Photocycloaddition Reactions

General Procedure for Isolation-Scale Asymmetric [2+2] Cycloadditions: An oven-dried Schlenk tube was charged with the 1-methylimidazoyl enone (0.4 mmol, 1.0 equiv), styrene (4.0 mmol, 10.0 equiv.), ***(R)*-AC-3** (0.08 mmol, 0.2 equiv.), and 8 mL toluene. The Schlenk was sealed with a glass stopper and degassed *via* freeze-pump-thaw technique (3 x 5 min). This was then cooled to –78 °C and irradiated with a Kessil Lamp (H150) for 14 h. The reaction mixture was then diluted 2–3x with CH_2Cl_2 before addition of 2 mL sat. aq. $NaHCO_3$. The mixture was vigorously stirred for 1 min, the organic layer separated, and the aqueous layer extracted with CH_2Cl_2 (2 x 4 mL). The combined organics were dried over Na_2SO_4, concentrated, and analyzed by ^{1}H NMR vs. internal standard (phenanthrene) to determine conversion and diastereomeric ratio. The crude mixture was then purified *via* flash column chromatography using Et_2O/pentanes as the eluent, giving an isolated mixture of separable diastereomers. The major diastereomer of each mixture was characterized.

2-(4-Methoxyphenyl)-3-phenylcyclobutyl)(1-methyl-1H-imidazol-2-yl)methanone (2): Prepared

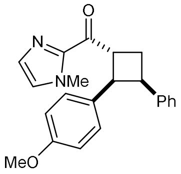

according to the general procedure for isolation-scale asymmetric experiments using 95.7 mg (0.39 mmol) (*E*)-3-(4-methoxyphenyl)-1-(1-methyl-1H-imidazol-2-yl)prop-2-en-1-one, 0.44 mL (4.0 mmol) of styrene, 61.3 mg (0.08 mmol) ***(R)*-AC-3**, and 8 mL toluene. The resulting material was purified by flash column chromatography eluting with 1:1 Et_2O/pentanes to give 84.8 mg (0.24 mmol, 62% yield) of two diastereomers (3:1 d.r.). Spectroscopic data were consistent with those previously reported.[12] Major Diastereomer: Viscous semisolid. 94% ee [Daicel CHIRALPAK OD-H, 5% to 50% iPrOH, 18 minutes, 1 mL/min, t_1=9.87 min, t_2=13.37 min]. $[\alpha]_D^{22}$ –57.7° (c0.45, CH_2Cl_2). ^{1}H NMR (500 MHz, $CDCl_3$) δ 7.17 (s, 1H), 7.16–7.13 (m, 2H), 7.09–7.05 (m, 3H), 7.04 (s, 1H), 6.89 (d, *J* = 8.5 Hz, 2H), 6.59 (d, *J* = 8.5 Hz, 2H), 4.98 (q, *J* = 8.8 Hz, 1H), 4.39 (t, *J* = 9.2 Hz, 1H), 4.03 (s, 3H), 3.95 (td, *J* = 8.9, 4.8 Hz, 1H), 3.67 (s, 3H), 2.78–2.69 (m, 2H). HRMS (ESI) calculated for $[C_{22}H_{22}N_2O_2]^+$ ($M+H^+$) requires *m/z* 347.1754, found 347.1750.

2-(2-Methoxyphenyl)-3-phenylcyclobutyl)(1-methyl-1H-imidazol-2-yl)methanone (3): Prepared

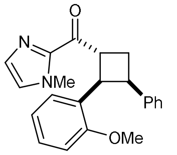

according to the general procedure for isolation-scale asymmetric experiments using 95.8 mg (0.40 mmol) (*E*)-3-(2-methoxyphenyl)-1-(1-methyl-1H-imidazol-2-yl)prop-2-en-1-one, 0.44 mL (4.0 mmol) of styrene, 61.1 mg (0.08 mmol) ***(R)*-AC-3**, and 8 mL toluene. The resulting material was purified by flash column chromatography using a gradient of 1:2 Et_2O/pentanes to 1:1 Et_2O/pentanes to give 68.2 mg (0.20 mmol, 50% yield) of two diastereomers (7:1 d.r.). Spectroscopic data were consistent with those previously reported.[12] Major Diastereomer: Viscous semisolid. 93% ee [Daicel CHIRALPAK OD-H, 5% to 50% iPrOH, 18 minutes, 1 mL/min, t_1=9.81 min, t_2=11.30 min]. $[\alpha]_D^{22}$ –115.4° (c1.71, CH_2Cl_2). ^{1}H NMR (500 MHz, $CDCl_3$) δ 7.20 (s, 1H), 7.17 (d, *J* = Hz, 1H), 7.12 (d, *J* = Hz, 2H), 7.08–7.05 (m, 2H), 7.05 (s, 1H), 7.00–6.95 (m, 2H), 6.69 (t, *J* = 7.5 Hz, 1H), 6.54 (d, *J* = 8.0 Hz, 1H), 5.20 (q, *J* = 9.4 Hz, 1H), 4.62 (t, *J* = 9.4 Hz, 1H), 4.01 (s, 3H), 3.99 (td, *J* = 9.4, 3.8 Hz, 1H), 3.65 (s, 3H), 2.81 (dt, 11.7, 9.0 Hz, 1H), 2.62 (ddd, *J* = 11.6, 9.5, 3.7 Hz, 1H). HRMS (ESI) calculated for $[C_{22}H_{22}N_2O_2]^+$ ($M+H^+$) requires *m/z* 347.1754, found 347.1751.

Methyl 4-(2-(1-methyl-1*H*-imidazole-2-carbonyl)-4-phenylcyclobutyl)benzoate (4): Prepared

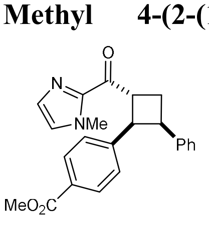

according to the general procedure for isolation-scale asymmetric experiments using 108.2 mg (0.40 mmol) methyl 4-[(1*E*)-3-(1-methyl-1*H*-imidazol-2-yl)-3-oxoprop-1-en-1-yl]benzoate, 0.46 mL (4.0 mmol) of styrene, 61.8 mg (0.08 mmol) ***(R)*-AC-3**, and 8 mL toluene. The resulting material was purified by flash column chromatography using a gradient of 1:1 Et_2O/pentanes to 3:1 Et_2O/pentanes to give 90.0 mg (0.24 mmol, 60% yield) of two diastereomers (2:1 d.r.). Major Diastereomer: Viscous semisolid. 91% ee [Daicel CHIRALPAK IC, 5% to 50% iPrOH, 18 minutes, 1 mL/min, t_1=12.44 min, t_2=14.70 min]. $[\alpha]_D^{22}$ –97.4° (c1.590, CH_2Cl_2). ^{1}H NMR (500 MHz, $CDCl_3$) δ 7.72 (d, *J* = 8.1 Hz, 2H), 7.19 (s, 1H), 7.13–7.03 (m, 8H), 5.07 (q, *J* = 9.0 Hz, 1H), 4.50 (t, *J* = 9.2 Hz, 1H), 4.04 (s, 3H), 4.03–4.00 (m, 1H), 3.82 (s, 3H), 2.80–

7.76 (m, 2H). ^{13}C NMR (125 MHz, $CDCl_3$) δ 192.59, 167.11, 145.27, 142.40, 140.29, 129.51, 129.03, 128.12, 128.04, 127.91, 127.59, 127.50, 126.11, 51.87, 45.63, 43.65, 42.04, 36.24, 28.64. HRMS (ESI) calculated for $[C_{23}H_{23}N_2O_3]^+$ ($M+H^+$) requires *m/z* 375.1703, found 375.1699.

(1-Methyl-1H-imidazol-2-yl)(3-phenyl-2-(4-(trifluoromethyl)phenyl)cyclobutyl)methanone (5):

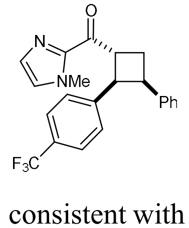

Prepared according to the general procedure for isolation-scale asymmetric experiments using 113.5 mg (0.40 mmol) (*E*)-1-(1-methyl-1*H*-imidazol-2-yl)-3-(4-trifluoromethylphenyl)prop-2-en-1-one, 0.46 mL (4.0 mmol) of styrene, 62.6 mg (0.08 mmol) **(*R*)-AC-3**, and 8 mL toluene. The resulting material was purified by flash column chromatography using a gradient of 1:3 to 1:1 Et_2O/pentanes to give 91.5 mg (0.24 mmol, 59% yield) of two diastereomers (2:1 d.r.). Spectroscopic data for both diastereomers were consistent with those previously reported.[12] Major Diastereomer: 80% ee [Daicel CHIRALPAK OD-H, 5% to 50% iPrOH, 18 minutes, 1 mL/min, t_1= 8.44 min, t_2= 11.42 min]. $[\alpha]_D^{22}$ –48.3° (c0.360, CH_2Cl_2). ^{1}H NMR (500 MHz, $CDCl_3$) δ 7.30 (d, *J* = 8.1 Hz, 2H), 7.19 (d, *J* = 0.7 Hz, 1H), 7.16–7.13 (m, 2H), 7.10–7.05 (m, 6H), 5.04 (q, *J* = 9.0 Hz, 1H), 4.49 (t, *J* = 9.3 Hz, 1H), 4.05 (s, 3H), 4.01 (td, *J* = 9.1, 5.0 Hz, 1H), 2.81–2.75 (m, 2H).

4-(2-(1-Methyl-1H-imidazole-2-carbonyl)-4-phenylcyclobutyl)benzonitrile (6): Prepared according to the general procedure for isolation-scale asymmetric experiments, irradiating for 24 h using 95.2 mg (0.40 mmol) (*E*)-4-(3-(1-methyl-1H-imidazol-2-yl)-3-oxoprop-1-en-1-yl)benzonitrile, 0.45 mL (4.0 mmol) of styrene, 60.5 mg (0.08 mmol) **(*R*)-AC-3**, and 8 mL toluene. The resulting material was purified by flash column chromatography using a gradient of 1:1 Et_2O/pentanes to 2:1 Et_2O/pentanes to give 72.0 mg (0.21 mmol, 53% yield) of three diastereomers (5:1 d.r.). Major Diastereomer: Viscous semisolid. 86% ee [Daicel CHIRALPAK OD-H, 5% to 50% iPrOH, 18 minutes, 1 mL/min, t_1=11.24 min, t_2=14.23 min]. $[\alpha]_D^{22}$ –166.3° (c0.54, CH_2Cl_2). ^{1}H NMR (500 MHz, $CDCl_3$) δ 7.34-7.32 (m, 2H), 7.19 (d, *J* = 0.9 Hz, 1H), 7.16-7.13 (m, 2H), 7.09-7.05 (m, 6H), 5.05 (q, *J* = 9.8 Hz, 1H), 4.48 (t, *J* = 9.4 Hz, 1H), 4.05 (s, 3H), 4.02 (td, *J* = 9.3, 4.5 Hz, 1H), 2.84-2.73 (m, 2H). ^{13}C NMR (125 MHz, $CDCl_3$) δ 192.24, 145.49, 142.35, 139.95, 131.51, 129.62, 128.63, 128.20, 128.07, 127.63, 126.36, 119.09, 109.56, 45.75, 43.53, 42.07, 36.22, 28.37. HRMS (ESI) calculated for $[C_{22}H_{19}N_3O]^+$ ($M+H^+$) requires *m/z* 342.1601, found 342.1598.

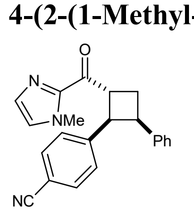

2-(2-Fluorophenyl)-3-phenylcyclobutyl)(1-methyl-1H-imidazol-2-yl)methanone (7): Prepared according to the general procedure for isolation-scale asymmetric experiments using 92.1 mg (0.40 mmol) (*E*)-3-(2-fluorophenyl)-1-(1-methyl-1H-imidazol-2-yl)prop-2-en-1-one, 0.44 mL (4.0 mmol) of styrene, 60.3 mg (0.08 mmol) **(*R*)-AC-3**, and 8 mL toluene. The resulting material was purified by flash column chromatography using a gradient of hexanes to 1:1 Et_2O/hexanes to give 80.7 mg (0.24 mmol, 60% yield) of two diastereomers (10:1 d.r.). Major Diastereomer: Viscous semisolid. 95% ee [Daicel CHIRALPAK AD-H, 5% to 50% iPrOH, 18 minutes, 1 mL/min, t_1=6.16 min, t_2=7.16 min]. $[\alpha]_D^{22}$ –90.3° (c0.33, CH_2Cl_2). ^{1}H NMR (500 MHz, $CDCl_3$) δ 7.20 (d, *J* = 1.0 Hz, 1H), 7.17-7.15 (m, 3H), 7.12-7.09 (m, 2H), 7.07 (s, 1H), 7.03-7.01 (m, 1H), 6.97-6.94 (m, 1H), 6.85-6.81 (m, 1H), 6.78-6.74 (m, 1H), 5.16 (q, *J* = 8.3 Hz, 1H), 4.66 (t, *J* = 9.5 Hz, 1H), 4.04 (s, 3H), 4.02 (td, *J* = 9.3, 3.9 Hz, 1H), 2.85-2.79 (m, 1H), 2.75-2.70 (s, 1H). ^{13}C NMR (125 MHz, $CDCl_3$) δ 192.64, 160.43, 142.62, 140.76, 129.47, 128.67, 127.90, 127.85, 127.54, 127.45, 126.95, 125.95, 123.35, 114.39, 42.11, 41.61, 40.18, 36.23, 28.70. ^{19}F NMR (377 MHz, $CDCl_3$) δ -115.91. HRMS (ESI) calculated for $[C21H_{19}FN_2O]^+$ ($M+H^+$) requires *m/z* 335.1554, found 335.1552.

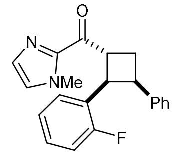

2-(4-Chlorophenyl)-3-phenylcyclobutyl)(1-methyl-1H-imidazol-2-yl)methanone (8): Prepared according to the general procedure for isolation-scale asymmetric experiments using 99.0 mg (0.40 mmol) (*E*)-3-(4-chlorophenyl)-1-(1-methyl-1H-imidazol-2-yl)prop-2-en-1-one, 0.44 mL (4.0 mmol) of styrene, 61.0 mg (0.08 mmol) **(*R*)-AC-3**, and 8 mL toluene. The resulting material was purified by flash column chromatography using a gradient of 1:2 Et_2O/pentanes to 1:1 Et_2O/pentanes to give 103.2 mg (0.29 mmol, 73% yield) of three diastereomers (6:1 d.r.). Major Diastereomer: Viscous semisolid. 96% ee [Daicel CHIRALPAK OD-H, 5% to 50% iPrOH, 18 minutes, 1 mL/min, t_1=8.88 min, t_2=12.15 min]. $[\alpha]_D^{22}$ –82.3° (c1.15, CH_2Cl_2). ^1H NMR (500 MHz, $CDCl_3$) δ 7.18-7.14 (m, 3H), 7.08-7.06 (m, 4H), 7.01 (d, *J* = 8.5 Hz, 2H), 6.91 (d, *J* = 8.5 Hz, 2H), 4.98 (q, *J* = 9.3 Hz, 1H), 4.40 (t, *J* = 9.3 Hz, 1H), 4.04 (s, 3H), 3.99-3.95 (m, 1H), 2.77-2.74 (m, 2H). ^{13}C NMR (125 MHz, $CDCl_3$) δ 192.72, 142.51, 140.38, 138.25, 131.58, 129.51, 129.30, 128.20, 128.05, 127.80, 127.45, 126.07, 45.27, 43.99, 41.90, 36.22, 28.38. HRMS (ESI) calculated for $[C_{21}H_{19}ClN_2O]^+$ ($M+H^+$) requires *m/z* 351.1259, found 351.1257.

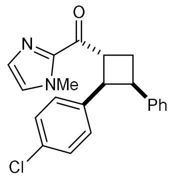

(1-Methyl-1H-imidazol-2-yl)(3-phenyl-2-(p-tolyl)cyclobutyl)methanone (9): Prepared according to the general procedure for isolation-scale asymmetric experiments using 89.3 mg (0.40 mmol) (*E*)-1-(1-methyl-1H-imidazol-2-yl)-3-(p-tolyl)prop-2-en-1-one, 0.45 mL (4.0 mmol) of styrene, 60.4 mg (0.08 mmol) **(*R*)-AC-3**, and 8 mL toluene. The resulting material was purified by flash column chromatography using a gradient of 1:2 Et_2O/pentanes to 1:1 Et_2O/pentanes to give 74.8 mg (0.23 mmol, 57% yield) of three diastereomers (4:1 d.r.). Major Diastereomer: Viscous semisolid. 98% ee [Daicel CHIRALPAK OD-H, 5% to 50% iPrOH, 18 minutes, 1 mL/min, t_1=8.34 min, t_2=10.88 min]. $[\alpha]_D^{22}$ –102.5° (c0.16, CH_2Cl_2). ^1H NMR (500 MHz, $CDCl_3$) δ 7.17 (d, *J* = 1.0 Hz, 1H), 7.14-7.12 (m, 2H), 7.10-7.04 (m, 4H), 6.88-6.84 (m, 4H), 5.01 (q, *J* = 8.7 Hz, 1H), 4.41 (t, *J* = 9.2 Hz, 1H), 4.03 (s, 3H), 3.96 (td, *J* = 8.9, 4.9 Hz, 1H), 2.76-2.72 (m, 2H), 2.16 (s, 3H). ^{13}C NMR (125 MHz, $CDCl_3$) δ 193.18, 142.68, 140.91, 136.59, 135.22, 129.40, 128.38, 128.29, 127.86, 127.86, 127.27, 125.77, 45.6l0, 44.08, 41.96, 36.22, 28.59, 20.95. HRMS (ESI) calculated for $[C_{22}H_{22}N_2O]^+$ ($M+H^+$) requires *m/z* 331.1805, found 331.1800.

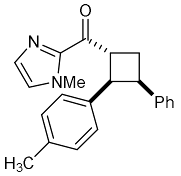

(1-Methyl-1H-imidazol-2-yl)(2-(naphthalen-2-yl)-3-phenylcyclobutyl)methanone (10): Prepared according to the general procedure for isolation-scale asymmetric experiments using 105.5 mg (0.40 mmol) (*E*)-1-(1-methyl-1H-imidazol-2-yl)-3-(naphthalen-2-yl)prop-2-en-1-one, 0.44 mL (4.0 mmol) of styrene, 61.3 mg (0.08 mmol) **(*R*)-AC-3**, and 8 mL toluene. The resulting material was purified by flash column chromatography using a gradient of 1:2 Et_2O/pentanes to 1:1 Et_2O/pentanes to give 63.6 mg (0.17 mmol, 43% yield) of two diastereomers (3:1 d.r.). Major Diastereomer: Viscous semisolid. 95% ee [Daicel CHIRALPAK OD-H, 5% to 50% iPrOH, 18 minutes, 1 mL/min, t_1=9.70 min, t_2=12.48 min]. $[\alpha]_D^{22}$ –104.6° (c0.43, CH_2Cl_2). ^1H NMR (500 MHz, $CDCl_3$) δ 7.64 (d, *J* = 9.4 Hz, 2H), 7.55 (s, 1H), 7.49 (d, *J* = 8.5 Hz, 1H), 7.36-7.30f (m, 2H), 7.21 (d, *J* = 1.0 Hz, 1H), 7.13-7.12 (m, 2H), 7.08-7.03 (m, 3H), 7.02 (dd, *J* = 8.5, 1.8 Hz, 1H), 6.99-6.96 (m, 1H), 5.17 (q, *J* = 8.9 Hz, 1H), 4.61 (t, *J* = 9.0 Hz, 1H), 4.10-4.04 (m, 4H), 2.83-2.80 (m, 2H). ^{13}C NMR (125 MHz, $CDCl_3$) δ 193.04, 142.64, 140.75, 137.44, 133.16, 131.90, 129.46, 128.24, 127.93, 127.66, 127.39, 127.38, 127.09, 126.72, 126.25, 125.91, 125.49, 125.07, 45.92, 44.04, 42.01, 36.24, 28.84. HRMS (ESI) calculated for $[C_{25}H_{22}N_2O]^+$ ($M+H^+$) requires *m/z* 367.1805, found 367.1803.

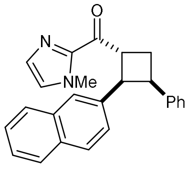

(1-Methyl-1H-imidazol-2-yl)(3-phenyl-2-(thiophen-2-yl)cyclobutyl)methanone (11): Prepared according to the general procedure for isolation-scale asymmetric experiments using 87.4 mg (0.40 mmol) (*E*)-1-(1-methyl-1H-imidazol-2-yl)-3-(thiophen-2-yl)prop-2-en-1-one, 0.45 mL (4.0 mmol) of styrene, 59.9 mg (0.08 mmol) **(*R*)-AC-3**, and 8 mL toluene. The resulting material was purified by flash column chromatography using a gradient of 15% to 50% EtOAc/hexanes to give 63.2 mg (0.20 mmol, 49% yield) of two diastereomers (3:1 d.r.). Spectroscopic data were consistent with those previously reported.[12] Major Diastereomer: Viscous

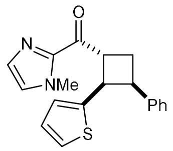

semisolid. 89% ee [Daicel CHIRALPAK OD-H, 5% to 50% iPrOH, 18 minutes, 1 mL/min, t_1=9.46 min, t_2=11.30 min]. $[\alpha]_D^{22}$ –42.0° (c0.10, CH_2Cl_2). ^{1}H NMR (500 MHz, $CDCl_3$) δ 7.22–7.17 (m, 5H), 7.14–7.11 (m, 1H), 7.06 (s, 1H), 6.93 (dd, *J* = 5.1, 1.1 Hz, 1H), 6.71 (dd, *J* = 4.9, 3.6 Hz, 1H), 6.64 (dt, *J* = 3.5, 1.1 Hz, 1H), 4.88 (q, *J* = 8.6 Hz, 1H), 4.62 (t, *J* = 8.8 Hz, 1H), 4.05 (s, 3H), 3.97 (td, *J* = 9.1, 5.1Hz, 1H), 2.86 (ddd, *J* = 11.8, 10.2, 5.0 Hz, 1H), 2.68 (dt, *J* = 12.0, 8.5 Hz, 1H). HRMS (ESI) calculated for $[C_{19}H_{18}N_2OS]^+$ ($M+H^+$) requires *m/z* 323.1213, found 323.1210.

(1-Methyl-1H-imidazol-2-yl)(2-(3-nitrophenyl)-3-phenylcyclobutyl)methanone (12): Prepared according to the general procedure for isolation-scale asymmetric experiments, irradiating for 24 hours using 102.8 mg (0.40 mmol) (*E*)-1-(1-methyl-1H-imidazol-2-yl)-3-(3-nitrophenyl)prop-2-en-1-one, 0.45 mL (4.0 mmol) of styrene, 61.0 mg (0.08 mmol) **(*R*)-AC-3**, and 8 mL toluene. The resulting material was purified by flash column chromatography using a gradient of 1:1 Et_2O/pentanes to 2:1 Et_2O/pentanes to give 108.7 mg (0.30 mmol, 75% yield) of two diastereomers (6:1 d.r.). Major Diastereomer: Viscous semisolid. 98% ee [Daicel CHIRALPAK OD-H, 5% to 50% iPrOH, 18 minutes, 1 mL/min, t_1=10.97 min, t_2=13.51 min]. $[\alpha]_D^{22}$ –101.8° (c0.32, CH_2Cl_2). ^{1}H NMR (500 MHz, $CDCl_3$) δ 7.91 (s, 1H), 7.86-7.84 (m, 1H), 7.28 (d, *J* = 7.8 Hz, 1H), 7.20-7.13 (m, 4H), 7.10-7.08 (m, 3H), 7.06-7.03 (m, 1H), 5.06 (q, *J* = 8.6 Hz, 1H), 4.52 (t, *J* = 9.4 Hz, 1H), 4.06-4.02 (m, 4H), 2.87-2.77 (m, 2H). ^{13}C NMR (125 MHz, $CDCl_3$) δ 192.13, 147.81, 142.31, 142.01, 139.82, 134.14, 129.67, 128.46, 128.26, 128.13, 127.59, 126.37, 122.95, 121.00, 45.34, 43.95, 41.95, 36.20, 28.13. HRMS (ESI) calculated for $[C_{21}H_{19}N_3O_3]^+$ ($M+H^+$) requires *m/z* 362.1499, found 362.1496.

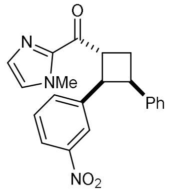

2-(2-Iodophenyl)-3-phenylcyclobutyl)(1-methyl-1H-imidazol-2-yl)methanone (13): Prepared according to the general procedure for isolation-scale asymmetric experiments using 135.6 mg (0.40 mmol) (*E*)-3-(2-iodophenyl)-1-(1-methyl-1H-imidazol-2-yl)prop-2-en-1-one, 0.45 mL (4.0 mmol) of styrene, 60.5 mg (0.08 mmol) **(*R*)-AC-3**, and 8 mL toluene. The resulting material was purified by flash column chromatography using a gradient of 1:2 Et_2O/pentanes to 1:1 Et_2O/pentanes to give 72.4 mg (0.16 mmol, 41% yield) of two diastereomers (10:1 d.r.). Major Diastereomer: Viscous semisolid. 97% ee [Daicel CHIRALPAK AD-H, 5% to 50% iPrOH, 18 minutes, 1 mL/min, t_1=5.69 min, t_2=6.18 min]. $[\alpha]_D^{22}$ –44.0° (c0.10, CH_2Cl_2). ^{1}H NMR (500 MHz, $CDCl_3$) δ 7.62 (dd, *J* = 7.9, 1.3 Hz, 1H), 7.24-7.22 (m, 2H), 7.19 (s, 1H), 7.17 (d, *J* = 7.8 Hz, 1H), 7.10 (t, *J* = 7.6 Hz, 2H), 7.05-6.99 (m, 3H), 6.68 (t, *J* = 7.5 Hz, 1H), 5.24 (q, *J* = 9.5 Hz, 1H), 4.55 (t, *J* = 9.6 Hz, 1H), 4.20, (td, *J* = 9.1, 3.0 Hz, 1H), 4.02 (s, 3H), 2.85-2.79 (m, 1H), 2.65-2.60 (m, 1H). ^{13}C NMR (125 MHz, $CDCl_3$) δ 192.28, 142.70, 141.63, 140.33, 138.85, 129.46, 128.68, 128.19, 127.80, 127.74, 127.51, 127.50, 125.95, 100.84, 50.67, 42.26, 41.10, 36.23, 27.68. HRMS (ESI) calculated for $[C21H_{19}IN_2O]^+$ ($M+H^+$) requires *m/z* 443.0615, found 443.0611.

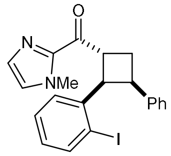

(2,3-Diphenylcyclobutyl)(1-methyl-1H-imidazol-2-yl)methanone (14): Prepared according to the general procedure for isolation-scale asymmetric experiments using 84.6 mg (0.40 mmol) 2-cinnamoyl-1-methylimidazole, 0.46 mL (4.0 mmol) styrene, 61.6 mg (0.08 mmol) **(*R*)-AC-3**, and 8 mL toluene. The resulting material was purified by flash column chromatography using a gradient of 1:2 to 1:1 Et_2O/pentanes to give 98.0 mg (0.31 mmol, 78% yield) of two diastereomers (7:1 d.r.). Spectroscopic data for the major diastereomer were consistent with those previously reported.[12] Major diastereomer: 95% ee [Daicel CHIRALPAK OD-H, 5% to 50% iPrOH, 18 minutes, 1 mL/min, t_1=8.97 min, t_2=11.84 min]. $[\alpha]_D^{22}$ –91.9° (c0.790, CH_2Cl_2). ^{1}H NMR (500 MHz, $CDCl_3$) δ 7.18 (d, *J* = 0.8 Hz, 1H), 7.14–7.11 (m, 2H), 7.09–7.04 (m, 6H), 6.99–6.98 (m, 3H), 5.05 (q, *J* = 9.0 Hz, 1H), 4.47 (t, *J* = 9.2 Hz, 1H), 4.04 (s, 3H), 3.99 (td, *J* = 8.7, 6.0 Hz, 1H), 2.78–2.74 (m, 2H).

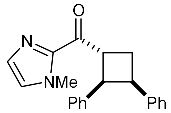

(3-(2-Chlorophenyl)-2-phenylcyclobutyl)(1-methyl-1H-imidazol-2-yl)methanone (**15**): Prepared according to the general procedure for isolation-scale asymmetric experiments using 85.1 mg (0.40 mmol) 2-cinnamoyl-1-methylimidazole, 0.51 mL (4.0 mmol) 2-chlorostyrene, 62.4 mg (0.08 mmol) **(*R*)-AC-3**, and 8 mL toluene The resulting material was purified by flash column chromatography using 1:1 Et_2O/pentanes to give 109.0 mg (0.31 mmol, 78% yield) of two diastereomers (10:1 d.r.). <u>Major Diastereomer:</u> 94% ee [Daicel CHIRALPAK AD, 5% to 50% iPrOH, 18 minutes, 1 mL/min, t_1=6.72 min, t_2=8.55 min]. White solid (mp = 94–97 °C). $[\alpha]_D^{22}$ –7.9° (c1.750, CH_2Cl_2). ^{1}H NMR (500 MHz, $CDCl_3$) δ 7.39 (dd, *J* = 8.0, 1.2 Hz, 1H), 7.16–6.98 (m, 10 H), 4.87 (dt, *J* = 9.7, 7.2 Hz, 1H), 4.51 (dd, *J* = 9.4, 7.2 Hz, 1H), 4.44 (td, *J* = 9.2, 6.2 Hz, 1H), 4.06 (s, 3H), 2.92 (ddd, *J* = 12.0, 10.1, 6.0 Hz, 1H), 2.74–2.68 (m, 1H). ^{13}C NMR (125 MHz, $CDCl_3$) δ 192.85, 142.45, 139.38, 138.15, 134.61, 129.40, 129.05, 128.15, 128.05, 127.52, 127.29, 127.23, 126.30, 126.03, 45.80, 43.87, 39.15, 36.26, 27.14. HRMS (ESI) calculated for $[C_{21}H_{20}ClN_2O]^+$ ($M+H^+$) requires *m/z* 351.1259, found 351.1258.

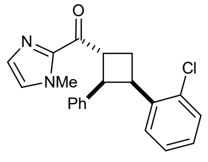

(3-(3-Chlorophenyl)-2-phenylcyclobutyl)(1-methyl-1H-imidazol-2-yl)methanone (**16**): Prepared according to the general procedure for isolation-scale asymmetric experiments using 83.0 mg (0.39 mmol) 2-cinnamoyl-1-methylimidazole, 0.52 mL (4.0 mmol) 3-chlorostyrene, 62.1 mg (0.08 mmol) **(*R*)-AC-3**, and 8 mL toluene. The resulting material was purified by flash column chromatography eluting from 10% acetone in pentane. to give 102.2 mg (0.29 mmol, 75% yield) of two diastereomers (4:1 d.r.). <u>Major diastereomer:</u> 95% ee [Daicel CHIRALPAK AD-H, 5% to 50% iPrOH, 28 minutes, 1 mL/min, t_1=8.46 min, t_2=12.35 min]. White solid (mp = 120–124 °C). $[\alpha]_D^{22}$ = -87.4° (c = 0.7, CH_2Cl_2). ^{1}H NMR (500 MHz, $CDCl_3$) δ 7.18 (s, 1H), 7.09-7.06 (m, 4H), 7.03-6.99 (m, 5H), 6.94-6.92 (m, 1H), 5.03 (q, *J* = 8.9 Hz, 1H), 4.46 (t, *J* = 8.9 Hz, 1H), 4.04 (s, 3H), 3.96 (td, *J* = 9.1, 4.8 Hz), 2.77-2.72 (m, 2H). ^{13}C NMR (125 MHz, $CDCl_3$) δ 192.79, 143.02, 142.50, 139.12, 133.71, 129.47, 129.04, 128.30, 127.88, 127.84, 127.44, 126.41, 126.07, 125.99, 45.77, 43.43, 41.70, 36.24, 28.30. HRMS (ESI) calculated for $[C_{21}H_{19}ClN_2O]^+$ ($M+H^+$) requires *m/z* 351.1259, found 351.1264.

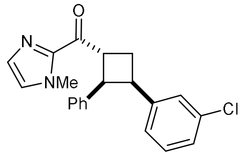

(3-(4-Chlorophenyl)-2-phenylcyclobutyl)(1-methyl-1H-imidazol-2-yl)methanone (**17**): Prepared according to the general procedure for isolation-scale asymmetric experiments using 85.5 mg (0.40 mmol) 2-cinnamoyl-1-methylimidazole, 0.48 mL (4.0 mmol) 4-chlorostyrene, 63.0 mg (0.08 mmol) **(*R*)-AC-3**, and 8 mL toluene. The resulting material was purified by flash column chromatography using 1:1 Et_2O/pentanes to give 109.5 mg (0.31 mmol, 77% yield) of two diastereomers (8:1 d.r.). <u>Major diastereomer:</u> 96% ee [Daicel CHIRALPAK AD, 5% to 50% iPrOH, 18 minutes, 1 mL/min, t_1=7.16 min, t_2=9.77 min]. White solid (mp = 121–124 °C). $[\alpha]_D^{22}$ –104.8° (c0.460, CH_2Cl_2). ^{1}H NMR (500 MHz, $CDCl_3$) δ 7.18 (s, 1H), 7.10–7.06 (m, 5H), 7.03–6.97 (m, 5H), 5.03 (q, *J* = 9.0 Hz, 1H), 4.46 (t, *J* = 9.3 Hz, 1H), 4.04 (s, 3H), 3.95 (td, *J* = 9.3, 4.6 Hz, 1H), 2.79–2.67 (m, 2H). ^{13}C NMR (125 MHz, $CDCl_3$) δ 192.81, 142.53, 139.40, 139.28, 131.53, 129.54, 129.48, 127.97, 127.86 (2C), 127.44, 126.03, 45.64, 43.50, 41.43, 36.25, 28.68. HRMS (ESI) calculated for $[C_{21}H_{20}ClN_2O]^+$ ($M+H^+$) requires *m/z* 351.1259, found 351.1261.

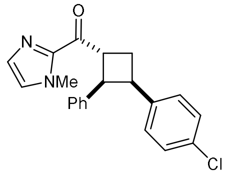

(3-(4-Bromophenyl)-2-phenylcyclobutyl)(1-methyl-1H-imidazol-2-yl)methanone (**18**): Prepared according to the general procedure for isolation-scale asymmetric experiments using 84.9 mg (0.40 mmol) 2-cinnamoyl-1-methylimidazole, 0.52 mL (4.0 mmol) 4-bromostyrene, 61.4 mg (0.08 mmol) **(*R*)-AC-3**, and 8 mL toluene. The resulting material was purified by flash column chromatography eluting from a gradient of 1:2 Et_2O/pentanes → 1:1 Et_2O/pentanes to give 118.5 mg (0.30 mmol, 75% yield) of two diastereomers (6:1 d.r.). <u>Major diastereomer:</u> 92% ee [Daicel CHIRALPAK OD-H, 5% to 30% iPrOH, 20 minutes, 1 mL/min, t_1=11.18 min, t_2=12.23 min]. White solid (mp = 131-135 °C). $[\alpha]_D^{22}$ = -84.5° (c = 1.2, CH_2Cl_2). ^{1}H NMR (500 MHz, $CDCl_3$) δ 7.23 (d, *J* = 8.4 Hz, 2H), 7.18 (s, 1H), 7.10-

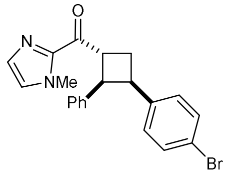

7.06 (m, 3H), 7.03-6.93 (m, 5H), 5.02 (q, J = 8.9 Hz, 1H), 4.46 (t, J = 9.3 Hz, 1H), 4.04 (s, 3H), 3.94 (td, J = 9.2, 4.5 Hz, 1H), 2.77-2.69 (m, 2H). ^{13}C NMR (125 MHz, $CDCl_3$) δ 192.78, 142.51, 139.94, 139.26, 130.91, 129.94, 129.48, 127.88, 127.85, 127.44, 126.04, 119.66, 45.56, 43.51, 41.48, 36.24, 28.65. HRMS (ESI) calculated for $[C_{21}H_{19}BrN_2O]^+$ (M+H$^+$) requires *m/z* 395.0754, found 395.0757.

(1-Methyl-1H-imidazol-2-yl)(2-phenyl-3-(4-(trifluoromethyl)phenyl)cyclobutyl)methanone (19):

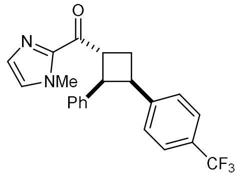

Prepared according to the general procedure for isolation-scale asymmetric experiments using 84.3 mg (0.40 mmol) 2-cinnamoyl-1-methylimidazole, 0.59 mL (4.0 mmol) 4-(trifluoromethyl)styrene, 63.0 mg (0.08 mmol) **(*R*)-AC-3**, and 8 mL toluene. The resulting material was purified by flash column chromatography eluting from a gradient of 1:2 Et_2O/pentanes to 1:1 Et_2O/pentanes to give 126.6 mg (0.33 mmol, 83% yield) of two diastereomers (5:1 d.r.). Spectroscopic data were consistent with those previously reported.[12] Major diastereomer: 96% ee [Daicel CHIRALPAK OD-H, 5% to 50% iPrOH, 13 minutes, 1 mL/min, t_1=8.17 min, t_2=9.45 min]. Viscous oil/semisolid. $[\alpha]_D^{22}$ = -74.4° (c = 2.0, CH_2Cl_2). ^{1}H NMR (500 MHz, $CDCl_3$) δ 7.36 (d, J = 8.1 Hz, 2H), 7.19-7.16 (m, 3H), 7.08-7.05 (m, 3H), 6.98-6.96 (m, 3H), 5.06 (q, J = 8.9 Hz, 1H), 4.51 (t, J = 9.4 Hz, 1H), 4.07-4.02 (m, 4H), 2.82-2.73 (m, 2H). ^{13}C NMR (125 MHz, $CDCl_3$) δ 192.64, 145.09, 142.49, 139.03, 129.52, 128.47, 128.15, 127.90, 127.80, 127.49, 126.15, 124.76, 123.18, 45.71, 43.50, 41.85, 36.24, 28.47. HRMS (ESI) calculated for $[C_{22}H_{19}F_3N_2O]^+$ (M+H$^+$) requires *m/z* 385.1522, found 385.1526.

(3-(4-*tert*-Butoxyphenyl)-2-phenylcyclobutyl)(1-methyl-1H-imidazol-2-yl)methanone (**20**): Prepared

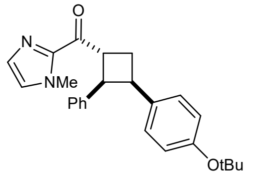

according to the general procedure for isolation-scale asymmetric experiments using 85.2 mg (0.40 mmol) 2-cinnamoyl-1-methylimidazole, 0.75 mL (4.0 mmol) 4-*tert*-butoxystyrene, 63.3 mg (0.08 mmol) **(*R*)-AC-3**, and 8 mL toluene. The resulting material was purified by flash column chromatography using a 1:1 Et_2O/pentanes to give 117.9 mg (0.30 mmol, 76% yield) of two diastereomers (8:1 d.r.). Major diastereomer: 83% ee [Daicel CHIRALPAK AD-H, 5% to 30% iPrOH, 20 minutes, 1 mL/min, t_1=7.90 min, t_2=8.56 min]. Viscous semisolid. $[\alpha]_D^{22}$ –69.0° (c0.620, CH_2Cl_2). ^{1}H NMR (500 MHz, $CDCl_3$) δ 7.17 (s, 1H), 7.04 (s, 1H), 7.03–7.00 (m, 2H), 6.97–6.93 (m, 5H), 6.75 (d, J = 8.4 Hz, 1H), 5.03 (q, J = 9.0 Hz, 1H), 4.41 (t, J = 9.3 Hz, 1H), 4.03 (s, 3H), 3.94 (dt, J = 9.3, 6.6 Hz, 1H), 2.76–2.73 (m, 2H), 1.23 (s, 9H). ^{13}C NMR (125 MHz, $CDCl_3$) δ 193.09, 153.13, 142.63, 139.54, 135.68, 129.40, 128.51, 128.04, 127.55, 127.33, 125.75, 123.90, 45.97, 43.53, 41.55, 36.23, 28.72 (2C), 28.25. HRMS (ESI) calculated for $[C_{25}H_{29}N_2O_2]^+$ (M+H$^+$) requires *m/z* 389.2224, found 329.2225.

(3-(4-Acetoxyphenyl)-2-phenylcyclobutyl)(1-methyl-1H-imidazol-2-yl)methanone (**21**): Prepared

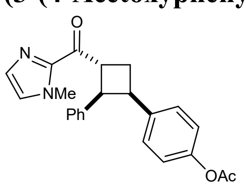

according to the general procedure for isolation-scale asymmetric experiments 84.9 mg (0.40 mmol) 2-cinnamoyl-1-methylimidazole, 0.61 mL (4.0 mmol) 4-acetoxystyrene, 62.7 mg (0.08 mmol) **(*R*)-AC-3**, and 8 mL toluene, The resulting material was purified by flash column chromatography using 1:1 Et_2O/pentanes to give 84.4 mg (0.23 mmol, 56% yield) of two diastereomers (13:1 d.r.). Major diastereomer: 97% ee [Daicel CHIRALPAK AD, 5% to 50% iPrOH, 18 minutes, 1 mL/min, t_1=10.79 min, t_2=12.47 min]. White solid (mp = 81–83 °C). $[\alpha]_D^{22}$ –90.0° (c1.180, CH_2Cl_2). ^{1}H NMR (500 MHz, $CDCl_3$) δ 7.17 (s, 1H), 7.08–7.04 (m, 5H), 7.01–6.98 (m, 3H), 6.85 (d, J = 8.6 Hz, 2H), 5.03 (q, J = 8.9 Hz, 1H), 4.45 (t, J = 9.2 Hz, 1H), 4.02 (s, 3H), 3.98 (td, J = 8.8, 5.5 Hz, 1H), 2.77–2.71 (m, 2H), 2.22 (s, 3H). ^{13}C NMR (125 MHz, $CDCl_3$) δ 192.90, 169.42, 148.69, 142.54, 139.39, 138.39, 129.44, 129.12, 127.93, 127.78, 127.39, 125.94, 120.85, 45.73, 43.58, 41.47, 36.22, 28.72, 21.11. HRMS (ESI) calculated for $[C_{23}H_{23}N_2O_3]^+$ (M+H$^+$) requires *m/z* 375.1703 found 375.1702.

3-(4-(((*tert*-Butyldimethylsilyl)oxy)methyl)phenyl)-2-phenylcyclobutyl)(1-methyl-1H-imidazol-2-yl)methanone (22): Prepared according to the general procedure for isolation-scale asymmetric experiments using 81.8 mg (0.39 mmol) 2-cinnamoyl-1-methylimidazole, 1.00 g (4.0 mmol) *O*-(*tert*-butyl)dimethylsilyl(4-vinylphenyl)methanol, 60.9 mg (0.08 mmol) **(*R*)-AC-3**, and 8 mL toluene. The resulting material was purified by flash column chromatography eluting with a gradient of 1:2 Et_2O/pentanes to 1:1 Et_2O/pentanes to give 144.3 mg (0.31 mmol, 81% yield) of three diastereomers (10:2:1 d.r.). <u>Major diastereomer:</u> 95% ee [Daicel CHIRALPAK OD-H, 5% to 50% iPrOH, 20 minutes, 1 mL/min, t_1=6.99 min, t_2=11.26 min]. Viscous oil/semisolid. $[\alpha]_D^{22}$ = -31.7° (c = 0.3, CH_2Cl_2). ^{1}H NMR (500 MHz, $CDCl_3$) δ 7.18 (s, 1H), 7.09-7.03 (m, 7H), 7.00-6.96 (m, 3H), 5.05 (q, *J* = 9.5 Hz, 1H), 4.62 (s, 2H) 4.45 (t, *J* = 9.3 Hz, 1H), 4.04 (s, 3H), 3.97 (dt, *J* = 9.5, 6.6 Hz, 1H), 2.74 (dd, *J* = 9.0, 6.7 Hz, 2H), 0.90 (s, 9H), 0.02 (d, *J* = 1.8 Hz, 6H). ^{13}C NMR (125 MHz, $CDCl_3$) δ 193.14, 142.62, 139.69, 139.43, 138.90, 129.43, 128.09, 127.93, 127.69, 127.33, 125.76, 125.76, 64.87, 45.72, 43.84, 41.73, 36.25, 28.85, 25.95, 18.39, -5.17. HRMS (ESI) calculated for $[C_{28}H_{36}SiN_2O_2]^+$ (M+H$^+$) - requires *m/z* 461.2619, found 461.2626.

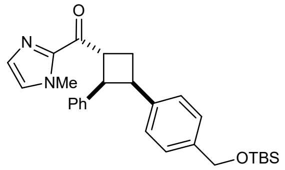

(3-(4-(Acetoxymethyl)phenyl)-2-phenylcyclobutyl)(1-methyl-1H-imidazol-2-yl)methanone (23): Prepared according to the general procedure for isolation-scale asymmetric experiments using 83.9 mg (0.40 mmol) 2-cinnamoyl-1-methylimidazole, 0.71 g (4.0 mmol) 4-acetoxymethylstyrene, 61.4 mg (0.08 mmol) **(*R*)-AC-3**, and 8 mL toluene. The resulting material was purified by flash column chromatography eluting with a gradient of hexanes to 30% EtOAc in hexanes to give 110.0 mg (0.28 mmol, 72% yield) of three diastereomers (10:2:1 d.r.). <u>Major diastereomer:</u> 97% ee [Daicel CHIRALPAK OD-H, 5% to 50% iPrOH, 28 minutes, 1 mL/min, t_1=13.45 min, t_2=23.64 min]. Viscous oil/semisolid. $[\alpha]_D^{22}$ = -51.0° (c = 0.2, CH_2Cl_2). ^{1}H NMR (500 MHz, $CDCl_3$) δ 7.18 (d, *J* = 0.9 Hz, 1H), 7.12-7.05 (m, 7H), 7.02-6.98 (m, 3H), 5.04 (q, *J* = 9.6 Hz, 1H), 4.98 (s, 2H) 4.47 (t, *J* = 9.6 Hz, 1H), 4.04 (s, 3H), 4.00 (td, *J* = 8.7, 6.1 Hz, 1H), 2.77-2.73 (m, 2H), 2.06 (s, 3H). ^{13}C NMR (125 MHz, $CDCl_3$) δ 192.98, 170.89, 142.57, 141.08, 139.56, 133.32, 129.45, 128.43, 127.91, 127.89, 127.75, 125.87, 66.13, 45.70, 43.73, 41.72, 36.25, 28.79, 21.05. HRMS (ESI) calculated for $[C_{24}H_{24}N_2O_3]^+$ (M+H$^+$) requires *m/z* 389.1860, found 339.1867.

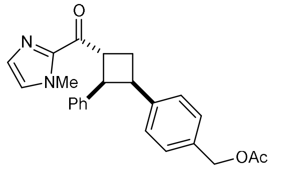

(3-(4-(Azidomethyl)phenyl)-2-phenylcyclobutyl)(1-methyl-1H-imidazol-2-yl)methanone (24): Prepared according to the general procedure for isolation-scale asymmetric experiments using 82.7 mg (0.39 mmol) 2-cinnamoyl-1-methylimidazole, 0.64 g (4.0 mmol) 1-(azidomethyl)-4-vinylbenzene, 61.2 mg (0.08 mmol) **(*R*)-AC-3**, and 8 mL toluene. The resulting material was purified by flash column chromatography eluting from a gradient of 1:2 Et_2O/pentanes → 1:1 Et_2O/pentanes to give 114.6 mg (0.31 mmol, 79% yield) of three diastereomers (10:2:1 d.r.). <u>Major diastereomer:</u> 94% ee [Daicel CHIRALPAK AD, 5% to 50% iPrOH, 18 minutes, 1 mL/min, t_1=8.51 min, t_2=10.99 min]. Viscous oil/semisolid. $[\alpha]_D^{22}$ = -88.0° (c = 0.5, CH_2Cl_2). ^{1}H NMR (500 MHz, $CDCl_3$) δ 7.18 (d, *J* = 0.9 Hz, 1H), 7.11-7.04 (m, 7H), 7.00-6.96 (m, 3H), 5.05 (q, *J* = 9.1 Hz, 1H), 4.47 (t, *J* = 9.3 Hz, 1H), 4.19 (s, 2H), 4.04 (s, 3H), 4.00 (dt, *J* = 9.7, 6.8 Hz, 1H), 2.76 (dd, *J* = 8.9, 6.6 Hz, 2H). ^{13}C NMR (125 MHz, $CDCl_3$) δ 192.92, 142.57, 141.11, 139.40, 132.57, 129.46, 128.69, 127.95, 127.90, 127.76, 127.39, 125.92, 54.46, 45.78, 43.68, 41.76, 36.24, 28.51. HRMS (ESI) calculated for $[C_{22}H_{21}N_5O]^+$ (M+H$^+$) requires *m/z* 372.1819, found 372.1826.

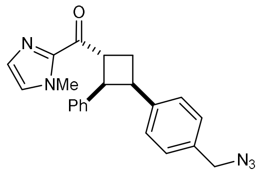

***tert*-Butyl(4-(3-(1-methyl-1H-imidazole-2-carbonyl)-2-phenylcyclobutyl)benzyl) carbamate (25):**

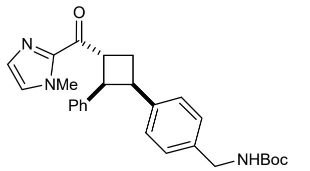

Prepared according to the general procedure for isolation-scale asymmetric experiments using 83.5 mg (0.39 mmol) 2-cinnamoyl-1-methylimidazole, 0.93 g (4.0 mmol) *tert*-butyl 4-vinylbenzylcarbamate, 61.7 mg (0.08 mmol) **(*R*)-AC-3**, and 8 mL toluene. The resulting material was purified by flash column chromatography eluting from a gradient of 30% EtOAc in hexanes → 50% EtOAc in hexanes to give 118.4 mg (0.27 mmol, 68% yield) of three diastereomers (10:2.5:1 d.r.). Major diastereomer: 95% ee [Daicel CHIRALPAK OD-H, 5% to 50% iPrOH, 28 minutes, 1 mL/min, t_1=17.23 min, t_2=22.76 min]. Viscous oil/semisolid. $[\alpha]_D^{22}$ = -66.5° (c = 0.7, CH_2Cl_2). ^1H NMR (500 MHz, $CDCl_3$) δ 7.18 (s, 1H), 7.06-7.03 (m, 7H), 6.99-6.98 (m, 3H), 5.04 (q, *J* = 8.9 Hz, 1H), 4.69 (s, 1H), 4.46 (t, *J* = 9.3 Hz, 1H), 4.18 (d, *J* = 5.1 Hz, 2H), 4.04 (s, 3H), 3.97 (td, *J* = 9.0, 5.1 Hz, 1H), 2.76-2.72 (m, 2H), 1.45 (s, 9H). ^{13}C NMR (125 MHz, $CDCl_3$) δ 193.03, 155.82, 142.57, 140.05, 139.63, 136.21, 129.43, 128.47, 127.89, 127.73, 127.37, 127.08, 125.83, 79.36, 45.66, 44.38, 43.74, 41.64, 36.25, 28.86, 28.41. HRMS (ESI) calculated for $[C_{27}H_{31}N_3O_3]^+$ ($M+H^+$) requires *m/z* 446.2438, found 446.2436.

(1-Methyl-1H-imidazol-2-yl)(2-phenyl-3-(phenylthio)cyclobutyl)methanone (**26**): Prepared according

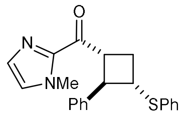

to the general procedure for isolation-scale asymmetric experiments using 85.2 mg (0.41 mmol) 2-cinnamoyl-1-methylimidazole, 0.53 mL (4.0 mmol) of phenyl vinyl sulfide, 121.8 mg (0.16 mmol) **(*R*)-AC-3**, and 8 mL toluene. The resulting material was purified by flash column chromatography using 1:1 Et_2O/pentanes to give 79.9 mg (0.23 mmol, 57% yield) of two diastereomers (1.2:1 d.r.). Spectroscopic data for both diastereomers were consistent with those previously reported.[12] Major Diastereomer: 78% ee [Daicel CHIRALPAK AD, 5% to 50% iPrOH, 18 minutes, 1 mL/min, t_1= 8.44 min, t_2= 12.46 min]. $[\alpha]_D^{22}$ –4.3° (c0.470, CH_2Cl_2). ^1H NMR (500 MHz, $CDCl_3$) δ 7.34 (d, *J* = 7.7 Hz, 2H), 7.30–7.28 (m, 4H), 7.25–7.18 (d, 4H), 7.12 (s, 1H), 7.02 (s, 1H), 4.48 (q, *J* = 9.1 Hz, 1H), 3.99 (s, 3H), 3.93 (t, *J* = 9.5 Hz, 1H), 3.85 (td, *J* = 9.4, 7.3 Hz, 1H), 2.93 (dt, *J* = 10.8, 8.2 Hz, 1H), 2.22 (q, *J* = 10.1 Hz, 1H). Minor Diastereomer: 75% ee [Daicel CHIRALPAK AD, 5% to 50% iPrOH, 18 minutes, 1 mL/min, t_1= 9.06 min, t_2= 10.22 min]. $[\alpha]_D^{22}$ –173.0° (c0.770, CH_2Cl_2). ^1H NMR (500 MHz, $CDCl_3$) δ 7.34 (d, *J* = Hz, 2H), 7.29 (t, *J* = 7.4 Hz, 1H), 7.24–7.15 (m, 4H), 7.12–7.07 (m, 3H), 7.04 (s, 1H), 5.14 (q, *J* = 8.9 Hz, 1H), 4.47 (t, *J* = 8.7 Hz, 1H), 4.35 (td, *J* = 7.8, 3.3 Hz, 1H), 4.01 (s, 3H), 2.76 (dt, *J* = 11.8 7.9 Hz, 1H), 2.47 (ddd, *J* = 12.1, 9.5, 3.0 Hz, 1H).

(1-Methyl-1H-imidazol-2-yl)(3-(naphthalen-2-yl)-2-phenylcyclobutyl)methanone (**27**): Prepared

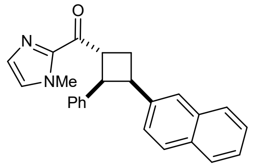

according to the general procedure for isolation-scale asymmetric experiments using 82.1 mg (0.39 mmol) 2-cinnamoyl-1-methylimidazole, 0.61 g (4.0 mmol) 2-vinylnapthalene, 60.3 mg (0.08 mmol) **(*R*)-AC-3**, and 8 mL toluene. The resulting material was purified by flash column chromatography eluting from a gradient of 1:2 Et_2O/pentanes to 1:1 Et_2O/pentanes to give 120.0 mg (0.33 mmol, 85% yield) of three diastereomers (5:1.5:1 d.r.). Major diastereomer: 90% ee [Daicel CHIRALPAK AD, 5% to 50% iPrOH, 13 minutes, 1 mL/min, t_1=7.61 min, t_2=9.76 min]. Viscous oil/semisolid. $[\alpha]_D^{22}$ = -97.7° (c = 0.4, CH_2Cl_2). ^1H NMR (500 MHz, $CDCl_3$) δ 7.75 (d, *J* = 6.9 Hz, 1H), 7.68 (d, *J* = 8.1 Hz, 1H), 7.65 (s, 1H), 7.52 (d, *J* = 8.5 Hz, 1H), 7.42-7.34 (m, 2H), 7.20 (d, *J* = 0.9 Hz, 1H), 7.07-7.05 (m, 2H), 7.03-6.98 (m, 4H), 6.94-6.91 (m, 1H), 5.12 (q, *J* = 9.5 Hz, 1H), 4.55 (t, *J* = 9.2 Hz, 1H), 4.16 (td, *J* = 9.1, 4.6 Hz, 1H), 4.05 (s, 3H), 2.90-2.82 (m, 2H). ^{13}C NMR (125 MHz, $CDCl_3$) δ 193.10, 142.61, 139.61, 138.56, 133.25, 131.90, 129.44, 127.94, 127.73, 127.67, 127.45, 127.39, 127.35, 127.25, 126.07, 125.86, 125.61, 125.11, 45.75, 43.95, 42.05, 36.27, 28.61.HRMS (ESI) calculated for $[C_{25}H_{22}N_2O]^+$ ($M+H^+$) requires *m/z* 367.1805, found 367.1808.

(3-(Benzofuran-5-yl)-2-phenylcyclobutyl)(1-methyl-1H-imidazol-2-yl)methanone (**28**): Prepared according to the general procedure for isolation-scale asymmetric experiments using 84.9 mg (0.40 mmol) 2-cinnamoyl-1-methylimidazole, 570.0 mg (4.0 mmol) 5-vinyl-1-benzofuran 61.5 mg (0.08 mmol) ***(R)*-AC-3**, and 8 mL toluene. The resulting material was purified by flash column chromatography using 1:1 Et_2O/pentanes to give 121.2 mg (0.34 mmol, 85% yield) of three diastereomers (4:1 d.r.). Major diastereomer: 94% ee [Daicel CHIRALPAK OD-H, 5% to 50% iPrOH, 18 minutes, 1 mL/min, t_1=9.081 min, t_2=10.288 min]. Viscous oil/semisolid. $[\alpha]_D^{22}$ = -28.0° (c = 0.7, CH_2Cl_2). ^{1}H NMR (500 MHz, $CDCl_3$) δ 7.51 (d, *J* = 2.2 Hz, 1H), 7.41 (s, 1H), 7.22-7.19 (m, 2H), 7.06 (s, 1H), 7.02-6.98 (m, 4H), 6.95-6.92 (m, 2H), 6.65 (d, *J* = 1.2 Hz, 1H), 5.09 (q, *J* = 9.2 Hz, 1H), 4.48 (t, *J* = 9.2 Hz, 1H), 4.10 (q, *J* = 7.6 Hz, 1H), 4.05 (s, 3H), 2.82-2.79 (m, 2H). ^{13}C NMR (125 MHz, $CDCl_3$) δ 193.14, 153.48, 144.75, 142.63, 139.69, 135.30, 129.42, 127.94, 127.68, 127.37, 127.09, 125.78, 125.21, 120.07, 110.57, 106.56, 45.88, 43.80, 41.91, 36.28, 29.18. HRMS (ESI) calculated for $[C_{23}H_{20}N_2O_2]^+$ ($M+H^+$) requires *m/z* 357.1598, found 357.1590.

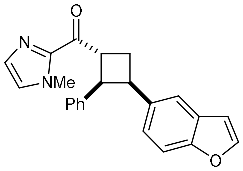

(1-Methyl-1H-imidazol-2-yl)(2-phenyl-3-(1-tosyl-1H-indol-5-yl)cyclobutyl)methanone (**29**): Prepared according to the general procedure for isolation-scale asymmetric experiments using 84.9 mg (0.40 mmol) 2-cinnamoyl-1-methylimidazole, 1.17 g (4.0 mmol) 1-toluenesulfonyl-5-vinyl-1H-indole, 61.3 mg (0.08 mmol) ***(R)*-AC-3**, and 8 mL toluene. The resulting material was purified by preparative HPLC eluting from 0.05% dioxane in H_2O to 85% MeCN/15% 0.05% dioxane in H_2O 165.1 mg (0.32 mmol, 81% yield) of three diastereomers (4:1 d.r.). Major diastereomer: 99% ee [Daicel CHIRALPAK AS-H, 5% to 50% iPrOH, 28 minutes, 1 mL/min, t_1=20.658 min, t_2=22.136 min]. White solid (mp = 155–159 °C). $[\alpha]_D^{22}$ = -35.2° (c = 0.3, CH_2Cl_2). ^{1}H NMR (500 MHz, $CDCl_3$) δ 7.68 (d, *J* = 8.5 Hz, 1H), 7.64 (d, *J* = 8.4 Hz, 2H), 7.43 (d, *J* = 3.7 Hz, 1H), 7.32 (s, 1H), 7.19-7.18 (m, 3H), 7.05 (s, 1H), 6.92-6.90 (m, 6H), 6.54 (d, *J* = 3.7 Hz, 1H), 5.03 (q, *J* = 9.0 Hz, 1H), 4.45 (t, *J* = 9.3 Hz, 1H), 4.06-4.01 (m, 4H), 2.77-2.74 (m, 2H), 2.35 (s, 3H). ^{13}C NMR (125 MHz, $CDCl_3$) δ 193.00, 144.61, 142.59, 139.48, 136.02, 135.26, 133.37, 130.64, 129.71, 129.41, 127.89, 127.60, 127.38, 126.66, 126.27, 125.75, 125.72, 120.18, 112.95, 109.42, 45.87, 43.75, 41.85, 36.26, 28.76, 21.56. HRMS (ESI) calculated for $[C_{30}H_{27}N_3O_3S]^+$ ($M+H^+$) requires *m/z* 510.1846, found 510.1839.

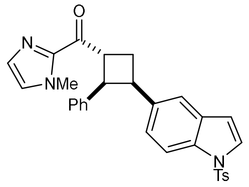

(1-Methyl-1H-imidazol-2-yl)(3-methyl-2-phenyl-3-(prop-1-en-2-yl)cyclobutyl)methanone (**30**): Prepared according to the general procedure isolation-scale asymmetric experiments using 86.1 mg (0.41 mmol) 2-cinnamoyl-1-methylimidazole, 0.45 mL (4.0 mmol) of 2,3-dimethylbutadiene, 61.0 mg (0.08 mmol) ***(R)*-AC-3**, and 8 mL toluene. The resulting material was purified by flash column chromatography using a gradient of 1:2 to 1:1 Et_2O/pentanes to give 58.0 mg (0.20 mmol, 49% yield) of two diastereomers (10:1 d.r.). Spectroscopic data for the major diastereomer was consistent with that previously reported.[12] Major diastereomer: 63% ee [Daicel CHIRALPAK AD-H, 5% to 30% iPrOH, 20 minutes, 1 mL/min, t_1= 5.75 min, t_2= 8.43 min]. $[\alpha]_D^{22}$ –52.5° (c0.640, CH_2Cl_2). ^{1}H NMR (500 MHz, $CDCl_3$) δ 7.31–7.25 (m, 4H), 7.19–7.16 (m, 1H), 7.18 (s, 1H), 7.04 (s, 1H), 4.91 (br s, 1H), 4.84 (p, *J* = 1.4 Hz, 1H), 4.79 (q, *J* = 9.5 Hz, 1H), 4.13 (d, *J* = 10.0 Hz, 1H) 3.99 (s, 3H), 2.28 (t, *J* = 9.9 Hz, 1H), 2.19 (dd, *J* = 10.3, 9.1 Hz, 1H), 1.78 (s, 3H), 1.10 (s, 3H).

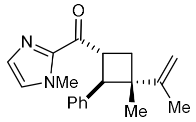

(3-Ethenyl-2-phenylcyclobutyl)(1-methyl-1H-imidazol-2-yl)methanone (**31**): Prepared according to the general procedure for isolation-scale asymmetric experiments using 85.1 mg (0.40 mmol) 2-cinnamoyl-1-methylimidazole, 2.2 mL (4.0 mmol) of 1,3-butadiene (15% in toluene), 63.2 mg (0.08 mmol) ***(R)*-AC-3**, and 6 mL toluene. The resulting material was purified by flash column chromatography using a gradient of 1:2 to 1:1 Et_2O/pentanes to give 78.8 mg (0.30 mmol, 74% yield) of two diastereomers (3:1 d.r.). Major Diastereomer: 87 % ee [Daicel CHIRALPAK AD, 5% to 50% iPrOH, 18 minutes, 1 mL/min, t_1=6.31 min, t_2=7.24 min]. Viscous oil. $[\alpha]_D^{22}$

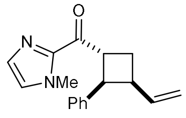

–121.4° (c2.500, CH_2Cl_2). ^{1}H NMR (500 MHz, $CDCl_3$) δ 7.28–7.25 (m, 2H), 7.20 (d, *J* = 7.6 Hz, 2H), 7.18 (s, 1H), 7.15 (tt, *J* = 7.2, 1.4 Hz, 1H), 7.05 (s, 1H), 5.76 (ddd, *J* = 17.0, 10.2, 8.5 Hz, 1H), 5.03 (dq, *J* = 17.0, 1.0 Hz, 1H), 4.97–4.91(m, 2H), 4.23 (t, *J* = 9.3 Hz, 1H), 3.33–3.27 (m, 1H), 2.52 (dt, *J* = 11.2, 8.7 Hz, 1H), 2.30 (ddd, *J* = 11.8, 9.5, 2.6 Hz, 1H). ^{13}C NMR (125 MHz, $CDCl_3$) δ 192.96, 142.62, 140.02, 138.84, 129.39, 128.00, 127.75, 125.98, 115.28, 44.21, 43.27, 40.53, 36.22, 28.86. HRMS (ESI) calculated for $[C_{17}H_{19}N_2O]^+$ $(M+H^+)$ requires *m/z* 267.1492, found 267.1491. <u>Minor Diastereomer</u>: 48% ee [Daicel CHIRALPAK AD, 5% to 50% iPrOH, 18 minutes, 1 mL/min, t_1=6.15 min, t_2=7.41 min]. Viscous oil. $[\alpha]_D^{22}$ –34.8° (c0.270, CH_2Cl_2). ^{1}H NMR (500 MHz, $CDCl_3$) δ 7.29–7.27 (m, 4H), 7.20–7.16 (m, 1H), 7.14 (s, 1H), 7.03 (s, 1H), 6.00 (ddd, *J* = 17.0, 10.2, 7.0 Hz, 1H), 5.09 (dt, *J* = 17.1, 1.5 Hz, 1H), 5.03 (dt, *J* = 10.3, 1.3 Hz, 1H), 4.49 (td, *J* = 9.7, 8.7 Hz, 1H), 4.02 (s, 3H), 3.76 (t, *J* = 9.7 Hz, 1H), 3.02 (pent, *J* =8.5 Hz, 1H), 2.59 (dt, *J* = 10.2, 8.4 Hz, 1H), 2.04 (q, *J* = 10.1 Hz, 1H). ^{13}C NMR (125 MHz, $CDCl_3$) δ 192.49, 142.62, 142.55, 140.74, 129.37, 128.32, 127.22, 126.80, 126.33, 114.50, 46.96, 44.91, 42.60, 36.17, 30.54. HRMS (ESI) calculated for $[C_{17}H_{19}N_2O]^+$ $(M+H^+)$ requires *m/z* 267.1492, found 267.1488.

(1-Methyl-1H-imidazol-2-yl)(1-phenylspiro[3.4]octan-2-yl)methanone (**34**): Prepared according to the general procedure for isolation-scale asymmetric experiments using 85.1 mg (0.40 mmol) 2-cinnamoyl-1-methylimidazole, 0.46 mL (4.0 mmol) methylenecyclopentane, 121.8 mg (0.16 mmol) **(*R*)-AC-3**, and 8 mL toluene, The resulting material was purified by flash column chromatography using a gradient of 1:2 to 1:1 Et_2O/pentanes to give 49.0 mg (0.17 mmol, 42% yield) of two of diastereomers (6:1 d.r.). Spectroscopic data for the major diastereomer were consistent with those previously reported.[12] <u>Major diastereomer</u>: 85% ee [Daicel CHIRALPAK OD-H, 5% to 50% iPrOH, 18 minutes, 1 mL/min, t_1=6.07 min, t_2=6.65 min]. $[\alpha]_D^{22}$ –71.0° (c0.600, CH_2Cl_2). ^{1}H NMR (500 MHz, $CDCl_3$) δ 7.28–7.27 (m, 4H), 7.19–7.16 (m, 1H), 7.17 (s, 1H), 7.02 (s, 1H), 4.79 (q, *J* = 9.5 Hz, 1H), 3.98 (s, 3H), 3.91 (d, *J* = 10.1 Hz, 1H), 2.21 (t, *J* = 9.6 Hz, 1H), 2.06 (t, *J* = 9.9 Hz, 1H), 1.83–1.72 (m, 2H), 1.55–1.46 (m, 4H), 1.33–1.26 (m, 2H).

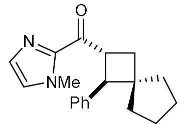

(1-Methyl-1H-imidazol-2-yl)(1-phenylspiro[3.3]heptan-2-yl)methanone (**35**): Prepared according to the general procedure for isolation-scale asymmetric experiments 85.0 mg (0.40 mmol) 2-cinnamoyl-1-methylimidazole, 0.37 mL (4.0 mmol) methylenecyclobutane, 121.0 mg (0.16 mmol) **(*R*)-AC-3**, and 8 mL toluene, The resulting material was purified by flash column chromatography using 1:1 Et_2O/pentanes to give 65.2 mg (0.23 mmol, 58% yield) of a single diastereomer (>20:1). 88% ee [Daicel CHIRALPAK AD, 5% to 50% iPrOH, 18 minutes, 1 mL/min, t_1=5.86 min, t_2=7.63 min]. White solid (mp = 80–82 °C). $[\alpha]_D^{22}$ –75.1° (c1.110, CH_2Cl_2). ^{1}H NMR (500 MHz, $CDCl_3$) δ 7.33–7.29 (m, 4H), 7.22–7.19 (m, 1H), 7.15 (s, 1H), 7.01 (s, 1H), 4.65 (q, *J* = 9.4 Hz, 1H), 3.97 (s, 3H), 3.67 (d, *J* = 9.8 Hz, 1H), 2.44 (dd, *J* = Hz, 1H), 2.20 (t, *J* = 10.3, 8.9 Hz, 1H), 2.11–2.06 (m, 1H), 2.00–1.94(m, 1H), 1.84–1.76 (m, 3H), 1.57–1.50 (m, 1H). ^{13}C NMR (125 MHz, $CDCl_3$) δ 192.75, 142.80, 140.01, 129.26, 128.16, 127.82, 127.21, 126.28, 50.21, 45.33, 40.57, 37.77, 36.18, 33.36, 29.38, 16.12. HRMS (ESI) calculated for $[C_{18}H_{21}N_2O]^+$ $(M+H^+)$ requires *m/z* 281.1648, found 281.1648.

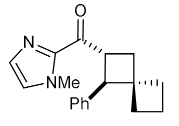

(3-(4-Bromo-2,5-dimethoxyphenyl)-2-(7-methoxy-2*H*-1,3-benzodioxol-5-yl)cyclobutyl)(1-methyl-1*H*-imidazol-2-yl)methanone (36): Prepared according to the general procedure for isolation-scale asymmetric experiments using 144.5 mg (0.50 mmol) (*E*)-3-(7-methoxybenzo[d][1,3]dioxol-5-yl)-1-(1-methyl-1*H*-imidazol-2-yl)prop-2-en-1-one, 582.1 mg (2.4 mmol) 4-bromo-2,5-methoxystyrene, 74.0 mg (0.10 mmol) **(*R*)-AC-3**, and 10 mL CH_2Cl_2. The resulting material was purified by flash column chromatography with 50% to 100% EtOAc in pentanes to give 173.0 mg (0.33 mmol, 65% yield) of two diastereomers (5:1 d.r., separated under these conditions). <u>Major Diastereomer:</u> White solid (mp = 78–82 °C, liquid phase is a *very* viscous oil). 93% ee [Daicel CHIRALPAK AD, 5% to 50% iPrOH, 20 minutes, 1 mL/min, t_1=13.00 min, t_2=14.46 min]. $[\alpha]_D^{22}$ –57.1° (c0.280, CH_2Cl_2). ^{1}H NMR (500 MHz, $CDCl_3$) δ 7.15 (s, 1H), 7.05 (s, 1H), 6.88 (s, 1H), 6.79 (s, 1H), 6.30 (d, *J* = 1.3 Hz, 1H), 6.20 (d, *J* = 1.3 Hz, 1H), 5.82 (dd, *J* = 4.5, 1.4 Hz, 2H), 4.75 (q, *J* = 8.1 Hz, 1H), 4.29 (dd, *J* = 9.6, 7.6 Hz, 1H), 4.21

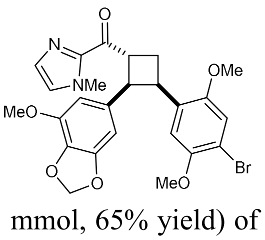

(td, J = 9.2, 6.2 Hz, 1H), 4.05 (s, 3H), 3.83 (s, 3H), 3.69 (s, 3H), 3.54 (s, 3H), 2.77–2.66 (m, 2H). ^{13}C NMR (125 MHz, $CDCl_3$) δ 192.82, 151.98, 149.80, 148.08, 142.76, 142.44, 134.99, 133.24, 129.77, 129.38, 127.33, 115.06, 112.76, 109.01, 107.10, 102.10, 101.06, 57.41, 56.29, 55.62, 46.03, 44.62, 36.24, 36.16, 26.76. HRMS (ESI) calculated for $[C_{25}H_{26}BrN_2O_6]^+$ (M+H$^+$) requires m/z 529.0969, found 529.0968. Minor Diastereomer: White solid (mp = 138–141 °C). 68% ee [Daicel CHIRALPAK AS-H, 5% to 30% EtOH, 35 minutes, 1 mL/min, t_1=17.84 min, t_2=18.80 min. $[\alpha]_D^{22}$ –17.8° (c0.540, CH_2Cl_2). ^{1}H NMR (500 MHz, $CDCl_3$) δ 7.15 (s, 1H), 7.05 (s, 1H), 7.00 (s, 1H), 6.98 (s, 1H), 6.51 (d, J = 1.3 Hz, 1H), 6.48 (d, J = 1.3 Hz, 1H), 5.90–5.89 (m, 2H), 4.44 (q, J = 9.2 Hz, 1H), 4.04 (s, 3H), 4.02 (q, 9.9 Hz, 1H), 3.88–3.83 (m, 1H), 3.87 (s, 3H), 3.84 (s, 3H), 3.70 (s, 3H), 2.83 (dt, J = 10.2, 8.6 Hz, 1H), 2.14 (q, J = 10.1 Hz, 1H). ^{13}C NMR (125 MHz, $CDCl_3$) δ 192.37, 151.86, 150.16, 148.77, 143.38, 142.59, 137.40, 133.69, 131.98, 129.46, 127.33, 115.87, 112.09, 109.18, 106.23, 101.23, 101.08, 57.12, 56.52, 56.14, 47.18, 46.32, 37.63, 36.18, 31.82. HRMS (ESI) calculated for $[C_{25}H_{26}BrN_2O_6]^+$ (M+H$^+$) requires m/z 529.0969, found 529.0967.

5. Racemic [2+2] Photocycloaddition Reactions

General Procedure for Racemic [2+2] Cycloadditions: An oven dried Schlenk tube was charged with the 1-methylimidazoyl enone (0.10 mmol, 1.0 equiv), styrene (1.0 mmol, 10.0 equiv.), **(±)-AC-3** (0.02 mmol, 0.2 equiv.), and 2 mL toluene. The Schlenk was sealed with a glass stopper and degassed *via* freeze-pump-thaw technique (3 x 5 min). This was then cooled to –78 °C and irradiated with a Kessil Lamp (H150) for 14 h. The reaction mixture was then diluted 2–3x with CH_2Cl_2 before addition of 2 mL sat. aq. $NaHCO_3$. The mixture was vigorously stirred for 60 s, the organic layer separated, and the aqueous layer extracted with CH_2Cl_2 (2 x 4 mL). The combined organics were dried over Na_2SO_4, concentrated, and analyzed by ^{1}H NMR vs. internal standard (phenanthrene) to determine conversion and diastereomeric ratio. The crude mixture was then purified *via* flash column chromatography using Et_2O/pentanes as the eluent.

2-(4-Methoxyphenyl)-3-phenylcyclobutyl)(1-methyl-1H-imidazol-2-yl)methanone ((±)-2): Prepared according to the general procedure for racemic experiments using 24.2 mg (0.10 mmol) (*E*)-3-(4-methoxyphenyl)-1-(1-methyl-1H-imidazol-2-yl)prop-2-en-1-one, 0.11 mL (1.0 mmol) styrene , 15.7 mg (0.02 mmol) **(±)-AC-3**, and 2 mL toluene. The resulting material was purified by flash column chromatography using a gradient of 1:2 to 1:1 Et_2O/pentanes to give a 65% NMR yield of two diastereomers (3:1 d.r.). Spectroscopic data were consistent with those reported above.

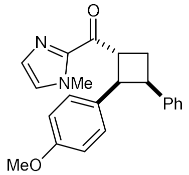

2-(2-Methoxyphenyl)-3-phenylcyclobutyl)(1-methyl-1H-imidazol-2-yl)methanone ((±)-3): Prepared according to the general procedure for racemic experiments using 23.3 mg (0.10 mmol) (*E*)-3-(2-methoxyphenyl)-1-(1-methyl-1H-imidazol-2-yl)prop-2-en-1-one, 0.11 mL (1.0 mmol) styrene , 15.6 mg (0.02 mmol) **(±)-AC-3**, and 2 mL toluene. The resulting material was purified by flash column chromatography using a gradient of 1:2 to 1:1 Et_2O/pentanes to give a 64% NMR yield of two diastereomers (6:1 d.r.). Spectroscopic data were consistent with those reported above.

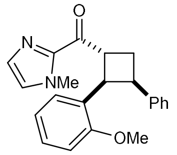

Methyl 4-(2-(1-methyl-1*H*-imidazole-2-carbonyl)-4-phenylcyclobutyl)benzoate ((±)-4): Prepared according to the general procedure for racemic experiments using 21.6 mg (0.10 mmol) 2-cinnamoyl-1-methylimidazole, 0.13 mL (1.0 mmol) phenyl vinyl sulfide, 30.1 mg (0.02 mmol) **(±)-AC-3**, and 2 mL toluene. The resulting material was purified by flash column chromatography using a 1:1 to 3:1 Et_2O/pentanes to give 7.9 mg (0.03 mmol, 29% yield) of two diastereomers (3:1 d.r.). Spectroscopic data were consistent with those reported above.

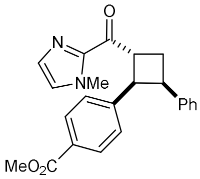

(1-Methyl-1H-imidazol-2-yl)(3-phenyl-2-(4-(trifluoromethyl)phenyl)cyclobutyl)methanone ((±)-5): Prepared according to the general procedure for racemic experiments using 28.0 mg (0.10 mmol) (*E*)-1-(1-methyl-1*H*-imidazol-2-yl)-3-(4-trifluoromethylphenyl)prop-2-en-1-one, 0.12 mL (1.0 mmol) styrene, 15.9 mg (0.02 mmol) BINOL-phosphoramide catalyst **(±)-AC-3**, and 2 mL toluene. The resulting material was purified by flash column chromatography using a gradient of 1:3 to 1:1 Et_2O/pentanes to give 23.5 mg (0.07 mmol, 70% yield) of two diastereomers (2:1 d.r.). Spectroscopic data were consistent with those reported above.

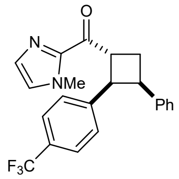

4-(2-(1-Methyl-1H-imidazole-2-carbonyl)-4-phenylcyclobutyl)benzonitrile ((±)-6): Prepared according to the general procedure for racemic experiments using 24.1 mg (0.10 mmol) (*E*)-4-(3-(1-methyl-1H-imidazol-2-yl)-3-oxoprop-1-en-1-yl)benzonitrile, 0.11 mL (1.0 mmol) styrene , 15.3 mg (0.02 mmol) **(±)-AC-3**, and 2 mL toluene. The resulting material was purified by flash column chromatography using a gradient of 1:1 to 2:1 Et_2O/pentanes to give a 30%

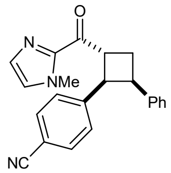

NMR yield of three diastereomers (5:1 d.r.). Spectroscopic data were consistent with those reported above.

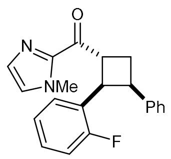

2-(2-Fluorophenyl)-3-phenylcyclobutyl)(1-methyl-1H-imidazol-2-yl)methanone ((±)-7): Prepared according to the general procedure for racemic experiments using 23.8 mg (0.10 mmol) (*E*)-3-(2-fluorophenyl)-1-(1-methyl-1H-imidazol-2-yl)prop-2-en-1-one, 0.11 mL (1.0 mmol) styrene , 15.8 mg (0.02 mmol) **(±)-AC-3**, and 2 mL toluene. The resulting material was purified by flash column chromatography using a gradient of 1:2 to 1:1 Et_2O/pentanes to give a 59% NMR yield of two diastereomers (10:1 d.r.). Spectroscopic data were consistent with those reported above.

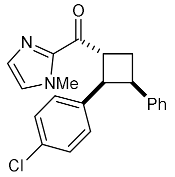

2-(4-Chlorophenyl)-3-phenylcyclobutyl)(1-methyl-1H-imidazol-2-yl)methanone ((±)-8): Prepared according to the general procedure for racemic experiments using 24.1 mg (0.10 mmol) (*E*)-3-(4-chlorophenyl)-1-(1-methyl-1H-imidazol-2-yl)prop-2-en-1-one, 0.11 mL (1.0 mmol) styrene , 15.3 mg (0.02 mmol) **(±)-AC-3**, and 2 mL toluene. The resulting material was purified by flash column chromatography using a gradient of 1:2 to 1:1 Et_2O/pentanes to give a 75% NMR yield of three diastereomers (5:1 d.r.). Spectroscopic data were consistent with those reported above.

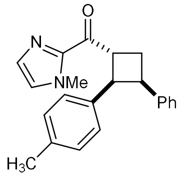

(1-Methyl-1H-imidazol-2-yl)(3-phenyl-2-(p-tolyl)cyclobutyl)methanone ((±)-9): Prepared according to the general procedure for racemic experiments using 22.6 mg (0.10 mmol) (*E*)-1-(1-methyl-1H-imidazol-2-yl)-3-(p-tolyl)prop-2-en-1-one, 0.11 mL (1.0 mmol) styrene , 14.9 mg (0.02 mmol) **(±)-AC-3**, and 2 mL toluene. The resulting material was purified by flash column chromatography using a gradient of 1:2 to 1:1 Et_2O/pentanes to give a 67% NMR yield of three diastereomers (3:1 d.r.). Spectroscopic data were consistent with those reported above.

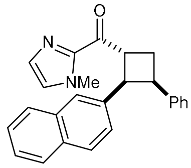

(1-Methyl-1H-imidazol-2-yl)(2-(naphthalen-2-yl)-3-phenylcyclobutyl)methanone ((±)-10): Prepared according to the general procedure for racemic experiments using 26.6 mg (0.10 mmol) (*E*)-1-(1-methyl-1H-imidazol-2-yl)-3-(naphthalen-2-yl)prop-2-en-1-one, 0.11 mL (1.0 mmol) styrene , 15.0 mg (0.02 mmol) **(±)-AC-3**, and 2 mL toluene. The resulting material was purified by flash column chromatography using a gradient of 1:2 to 1:1 Et_2O/pentanes to give a 59% NMR yield of two diastereomers (3:1 d.r.). Spectroscopic data were consistent with those reported above.

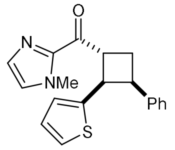

(1-Methyl-1H-imidazol-2-yl)(3-phenyl-2-(thiophen-2-yl)cyclobutyl)methanone ((±)-11): Prepared according to the general procedure for racemic experiments using 21.2 mg (0.10 mmol) (*E*)-1-(1-methyl-1H-imidazol-2-yl)-3-(thiophen-2-yl)prop-2-en-1-one, 0.11 mL (1.0 mmol) styrene , 15.0 mg (0.02 mmol) **(±)-AC-3**, and 2 mL toluene. The resulting material was purified by flash column chromatography using a gradient of 1:2 to 1:1 Et_2O/pentanes to give a 52% yield of two diastereomers (3:1 d.r.). Spectroscopic data were consistent with those reported above.

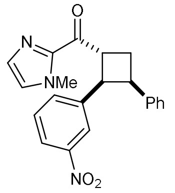

(1-Methyl-1H-imidazol-2-yl)(2-(3-nitrophenyl)-3-phenylcyclobutyl)methanone ((±)-12): Prepared according to the general procedure for racemic experiments using 26.2 mg (0.10 mmol) (*E*)-1-(1-methyl-1H-imidazol-2-yl)-3-(3-nitrophenyl)prop-2-en-1-one, 0.11 mL (1.0 mmol) styrene , 15.8 mg (0.02 mmol) **(±)-AC-3**, and 2 mL toluene. The resulting material was purified by flash column chromatography using a gradient of 1:1 to 2:1 Et_2O/pentanes to give a 33% NMR yield of two diastereomers (3:1 d.r.). Spectroscopic data were consistent with those reported above.

2-(2-Iodophenyl)-3-phenylcyclobutyl)(1-methyl-1H-imidazol-2-yl)methanone ((±)-13): Prepared

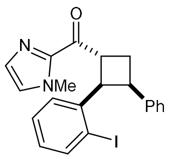

according to the general procedure for racemic experiments using 33.5 mg (0.10 mmol) (*E*)-3-(2-iodophenyl)-1-(1-methyl-1H-imidazol-2-yl)prop-2-en-1-one, 0.11 mL (1.0 mmol) styrene , 15.0 mg (0.02 mmol) **(±)-AC-3**, and 2 mL toluene. The resulting material was purified by flash column chromatography using a gradient of 1:2 to 1:1 Et_2O/pentanes to give a 49% NMR yield of two diastereomers (10:1 d.r.). Spectroscopic data were consistent with those reported above.

(2,3-Diphenylcyclobutyl)(1-methyl-1H-imidazol-2-yl)methanone ((±)-14): Prepared according to the

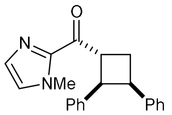

general procedure for racemic experiments using 22.4 mg (0.11 mmol) 2-cinnamoyl-1-methylimidazole, 0.12 mL (1.0 mmol) styrene, 16.4 mg (0.02 mmol) BINOL-phosphoramide catalyst **(±)-AC-3**, and 2 mL toluene. The resulting material was purified by flash column chromatography using a gradient of 1:2 to 1:1 Et_2O/pentanes to give 23.5 mg (0.07 mmol, 70% yield) of two diastereomers (7:1 d.r.). Spectroscopic data were consistent with those reported above.

(3-(2-Chlorophenyl)-2-phenylcyclobutyl)(1-methyl-1H-imidazol-2-yl)methanone (**(±)-15**): Prepared

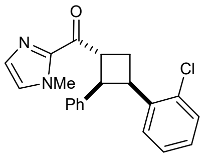

according to the general procedure for racemic experiments using 22.2 mg (0.10 mmol) 2-cinnamoyl-1-methylimidazole, 0.13 mL (1.0 mmol) 2-chlorostyrene, 16.5 mg (0.02 mmol) **(±)-AC-3**, and 2 mL toluene. The resulting material was purified by flash column chromatography using a 1:1 Et_2O/pentanes to give 19.7 mg (0.06 mmol, 54% yield) of two diastereomers (10:1 d.r.). Spectroscopic data were consistent with those reported above.

(3-(3-Chlorophenyl)-2-phenylcyclobutyl)(1-methyl-1H-imidazol-2-yl)methanone (**(±)-16**): Prepared

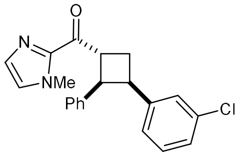

according to the general procedure for racemic experiments using 21.3 mg (0.10 mmol) 2-cinnamoyl-1-methylimidazole, 0.13 mL (1.0 mmol) 3-chlorostyrene, 15.6 mg (0.02 mmol) **(±)-AC-3**, and 2 mL toluene. The resulting material was purified by flash column chromatography using a 1:1 Et_2O/pentanes to give a 61% NMR yield of two diastereomers (4:1 d.r.). Spectroscopic data were consistent with those reported above.

(3-(4-Chlorophenyl)-2-phenylcyclobutyl)(1-methyl-1H-imidazol-2-yl)methanone (**(±)-17**): Prepared

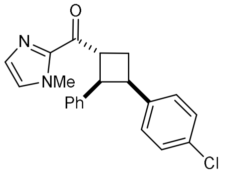

according to the general procedure for racemic experiments using 21.2 mg (0.10 mmol) 2-cinnamoyl-1-methylimidazole, 0.12 mL (1.0 mmol) 4-chlorostyrene, 15.3 mg (0.02 mmol) **(±)-AC-3**, and 2 mL toluene. The resulting material was purified by flash column chromatography using a 1:1 Et_2O/pentanes to give 26.7 mg (0.08 mmol, 76% yield) of two diastereomers (6:1 d.r.). Spectroscopic data were consistent with those reported above.

(3-(4-Bromophenyl)-2-phenylcyclobutyl)(1-methyl-1H-imidazol-2-yl)methanone (**(±)-18**): Prepared

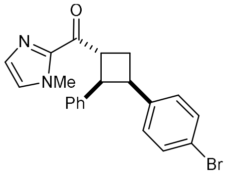

according to the general procedure for racemic experiments using 21.2 mg (0.10 mmol) 2-cinnamoyl-1-methylimidazole, 0.13 mL (1.0 mmol) 4-bromostyrene, 15.4 mg (0.02 mmol) **(±)-AC-3**, and 2 mL toluene. The resulting material was purified by flash column chromatography eluting from a gradient of 1:2 Et_2O/pentanes → 1:1 Et_2O/pentanes to give 3.4 mg (0.01 mmol, 9% yield) of two diastereomers (6:1 d.r.). Spectroscopic data were consistent with those reported above.

(1-Methyl-1H-imidazol-2-yl)(2-phenyl-3-(4-(trifluoromethyl)phenyl)cyclobutyl)methanone ((±)-19):

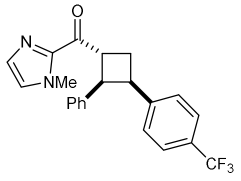

Prepared according to the general procedure for racemic experiments using 21.7 mg (0.10 mmol) 2-cinnamoyl-1-methylimidazole, 0.15 mL (1.0 mmol) 4-(trifluoromethyl)styrene, 16.1 mg (0.02 mmol) **(±)-AC-3**, and 2 mL toluene. The resulting material was purified by flash column chromatography eluting from a gradient of 1:2 Et_2O/pentanes → 1:1 Et_2O/pentanes to give 33.6 mg (0.09 mmol, 85% yield) of two diastereomers (5:1 d.r.). Spectroscopic data were consistent with those reported above.

(3-(4-*tert*-Butoxyphenyl)-2-phenylcyclobutyl)(1-methyl-1H-imidazol-2-yl)methanone ((±)-20):

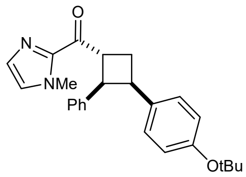

Prepared according to the general procedure for racemic experiments using 22.2 mg (0.10 mmol) 2-cinnamoyl-1-methylimidazole, 0.19 mL (1.0 mmol) 4-tert-butoxystyrene, 16.0 mg (0.02 mmol) **(±)-AC-3**, and 2 mL toluene. The resulting material was purified by flash column chromatography using a 1:1 Et_2O/pentanes to give 28.0 mg (0.07 mmol, 69% yield) of two diastereomers (4:1 d.r.). Spectroscopic data were consistent with those reported above.

(3-(4-Acetoxyphenyl)-2-phenylcyclobutyl)(1-methyl-1H-imidazol-2-yl)methanone ((±)-21): Prepared

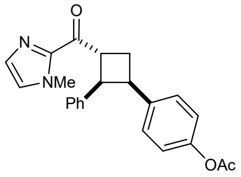

according to the general procedure for racemic experiments using 21.1 mg (0.10 mmol) 2-cinnamoyl-1-methylimidazole, 0.15 mL (1.0 mmol) 4-acetoxystyrene, 16.0 mg (0.02 mmol) **(±)-AC-3**, and 2 mL toluene. The resulting material was purified by flash column chromatography using a 1:1 Et_2O/pentanes to give 22.6 mg (0.06 mmol, 61% yield) of two diastereomers (8:1 d.r.). Spectroscopic data were consistent with those reported above.

3-(4-(((*tert*-Butyldimethylsilyl)oxy)methyl)phenyl)-2-phenylcyclobutyl)(1-methyl-1H-imidazol-2-yl)methanone ((±)-22):

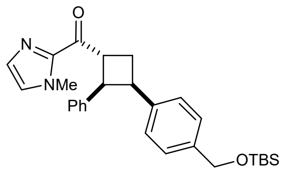

Prepared according to the general procedure for racemic experiments using 20.6 mg (0.10 mmol) 2-cinnamoyl-1-methylimidazole, 247.9 mg (1.0 mmol) *tert*-butyldimethyl(4-vinylbenzyloxy) silane, 15.4 mg (0.02 mmol) **(±)-AC-3**, and 2 mL toluene. The resulting material was purified by flash column chromatography eluting from a gradient of 1:2 Et_2O/pentanes → 1:1 Et_2O/pentanes to give 40.0 mg (0.09 mmol, 89% yield) of three diastereomers (3:1 d.r.). Spectroscopic data were consistent with those reported above.

(3-(4-(Acetoxymethyl)phenyl)-2-phenylcyclobutyl)(1-methyl-1H-imidazol-2-yl)methanone ((±)-23):

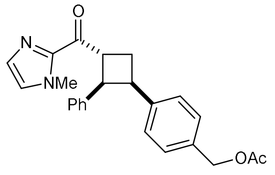

Prepared according to the general procedure for racemic experiments using 21.3 mg (0.10 mmol) 2-cinnamoyl-1-methylimidazole, 180.0 mg (1.0 mmol) 1-(acetoxymethyl)-4-vinylbenzene, 16.0 mg (0.02 mmol) **(±)-AC-3**, and 2 mL toluene. The resulting material was purified by flash column chromatography eluting from a gradient of 1:2 Et_2O/pentanes → 1:1 Et_2O/pentanes to give a 70% NMR yield of three diastereomers (3:1 d.r.). Spectroscopic data were consistent with those reported above.

(3-(4-(Azidomethyl)phenyl)-2-phenylcyclobutyl)(1-methyl-1H-imidazol-2-yl)methanone ((±)-24):

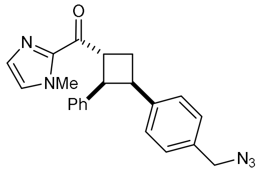

Prepared according to the general procedure for racemic experiments using 20.5 mg (0.10 mmol) 2-cinnamoyl-1-methylimidazole, 158.0 mg (1.0 mmol) 1-(azidomethyl)-4-vinylbenzene, 15.3 mg (0.02 mmol) **(±)-AC-3**, and 2 mL toluene. The resulting material was purified eluting from a gradient of 1:2 Et_2O/pentanes → 1:1 Et_2O/pentanes to give a 57% NMR yield of three diastereomers (3:1 d.r.). Spectroscopic data were consistent with those reported above.

***tert*-Butyl(4-(-3-(1-methyl-1H-imidazole-2-carbonyl)-2-phenylcyclobutyl)benzyl) carbamate ((±)-25):**

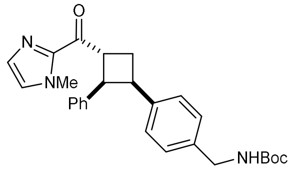

Prepared according to the general procedure for racemic experiments using 21.4 mg (0.10 mmol) 2-cinnamoyl-1-methylimidazole, 233 mg (1.0 mmol) *tert*-butyl 4-vinylbenzylcarbamate, 15.0 mg (0.02 mmol) **(±)-AC-3**, and 2 mL toluene. The resulting material was purified by flash column chromatography eluting from a gradient of 30% EtOAc/hexanes → 50% EtOAc/hexanes to give 27.4 mg (0.06 mmol, 61% yield) of three diastereomers (3:1 d.r.). Spectroscopic data were consistent with those reported above.

(1-Methyl-1H-imidazol-2-yl)(2-phenyl-3-(phenylthio)cyclobutyl)methanone ((**±)-26**):

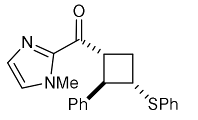

Prepared according to the general procedure for racemic experiments using 21.6 mg (0.10 mmol) 2-cinnamoyl-1-methylimidazole, 0.13 mL (1.0 mmol) phenyl vinyl sulfide, 30.1 mg (0.02 mmol) **(±)-AC-3**, and 2 mL toluene. The resulting material was purified by flash column chromatography using a 1:1 Et_2O/pentanes to give 7.9 mg (0.03 mmol, 29% yield) of two diastereomers (1.3:1 d.r.). Spectroscopic data were consistent with those reported above.

(1-Methyl-1H-imidazol-2-yl)(3-(naphthalen-2-yl)-2-phenylcyclobutyl)methanone ((**±)-27**):

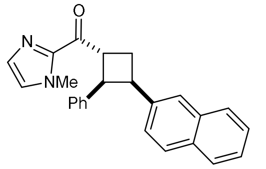

Prepared according to the general procedure for racemic experiments using 22.0 mg (0.10 mmol) 2-cinnamoyl-1-methylimidazole, 150.4 mg (1.0 mmol) 2-vinylnapthalene, 15.0 mg (0.02 mmol) **(±)-AC-3**, and 2 mL toluene. The resulting material was purified by flash column chromatography eluting from a gradient of 1:2 Et_2O/hexanes → 1:1 Et_2O/hexanes to give 33.1 mg (0.09 mmol, 87% yield) of three diastereomers (2:1 d.r.). Spectroscopic data were consistent with those reported above.

(3-(Benzofuran-5-yl)-2-phenylcyclobutyl)(1-methyl-1H-imidazol-2-yl)methanone ((**±)-28**):

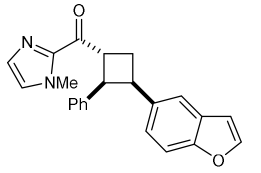

Prepared according to the general procedure for racemic experiments using 20.7 mg (0.10 mmol) 2-cinnamoyl-1-methylimidazole, 142.0 mg (1.0 mmol) 5-vinyl-1-benzofuran, 15.4 mg (0.02 mmol) **(±)-AC-3**, and 2 mL toluene. The resulting material was purified by flash column chromatography eluting from a gradient of 1:2 Et_2O/pentanes → 1:1 Et_2O/pentanes to give 30.2 mg (0.09 mmol, 87% yield) of three diastereomers (4:1 d.r.). Spectroscopic data were consistent with those reported above.

(1-Methyl-1H-imidazol-2-yl)((1S,2S,3S)-2-phenyl-3-(1-tosyl-1H-indol-5-yl)cyclobutyl)methanone

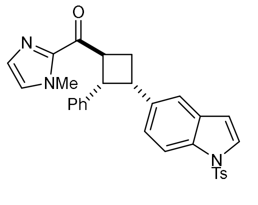

((**±)-29**): Prepared according to the general procedure for racemic experiments using 21.4 mg (0.10 mmol) 2-cinnamoyl-1-methylimidazole, 295.1 mg (1.0 mmol) 1-toluenesulfonyl-5-vinyl-1H-indole, 15.6 mg (0.02 mmol) **(±)-AC-3**, and 2 mL toluene. The resulting material was purified by flash column chromatography eluting from 20% acetone/pentanes to give an 80% NMR yield of three diastereomers (4:1 d.r.). Spectroscopic data were consistent with those reported above.

(1-Methyl-1H-imidazol-2-yl)(3-methyl-2-phenyl-3-(prop-1-en-2-yl)cyclobutyl)methanone ((**±)-30**):

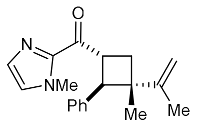

Prepared according to the general procedure for racemic experiments using 22.0 mg (0.10 mmol) 2-cinnamoyl-1-methylimidazole, 0.11 mL (1.0 mmol) 2,3-dimethylbutadiene, 15.8 mg (0.02 mmol) **(±)-AC-3**, and 2 mL toluene. The resulting material was purified by flash column chromatography using a gradient of 1:2 to 1:1 Et_2O/pentanes to give 17.7 mg (0.06 mmol, 57% yield) of two diastereomers (10:1 d.r.). Spectroscopic data were consistent with those reported above.

(3-Ethenyl-2-phenylcyclobutyl)(1-methyl-1H-imidazol-2-yl)methanone ((±)-**30**): Prepared according to the general procedure for racemic experiments using 21.1 mg (0.10 mmol) 2-cinnamoyl-1-methylimidazole, 0.55 mL (1.0 mmol) 1,3-butadiene (15% in toluene), 15.8 mg (0.02 mmol) **(±)-AC-3**, and 2 mL toluene. The resulting material was purified by flash column chromatography using a gradient of 1:2 to 1:1 Et_2O/pentanes to give 17.0 mg (0.06 mmol, 64% yield) of two diastereomers (3:1 d.r.). Spectroscopic data were consistent with those reported above.

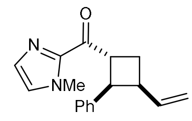

(1-Methyl-1H-imidazol-2-yl)(1-phenylspiro[3.4]octan-2-yl)methanone ((±)-**34**): Prepared according to the general procedure for racemic experiments using 21.6 mg (0.10 mmol) 2-cinnamoyl-1-methylimidazole, 0.08 mL (1.0 mmol) methylenecyclopentane, 30.7 mg (0.04 mmol) **(±)-AC-3**, and 2 mL toluene. The resulting material was purified by flash column chromatography using a gradient of 1:2 to 1:1 Et_2O/pentanes to give 14.2 mg (0.05 mmol, 47% yield) of two diastereomers (6:1 d.r.). Spectroscopic data were consistent with those reported above.

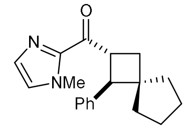

(1-Methyl-1H-imidazol-2-yl)(1-phenylspiro[3.3]heptan-2-yl)methanone ((±)-**35**): Prepared according to the general procedure for racemic experiments using 22.4 mg (0.11 mmol) 2-cinnamoyl-1-methylimidazole, 0.10 mL (1.0 mmol) methylenecyclobutane, 34.0 mg (0.04 mmol) **(±)-AC-3**, and 2 mL toluene. The resulting material was purified by flash column chromatography using a 1:1 Et_2O/pentanes to give 11.0 mg (0.04 mmol, 37% yield) of a single diastereomer. Spectroscopic data were consistent with those reported above.

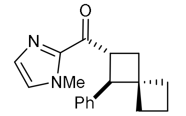

(3-(4-Bromo-2,5-dimethoxyphenyl)-2-(7-methoxy-2*H*-1,3-benzodioxol-5-yl)cyclobutyl)(1-methyl-1*H*-imidazol-2-yl)methanone (**(±)-36)** Prepared according to the general procedure for racemic experiments using 142.8 mg (0.50 mmol) (*E*)-3-(7-methoxybenzo[d][1,3]dioxol-5-yl)-1-(1-methyl-1*H*-imidazol-2-yl)prop-2-en-1-one, 244.1 mg (1.0 mmol) 4-bromo-2,5-methoxystyrene, 78.0 mg (0.10 mmol) **(±)-AC-3**, and 10 mL CH_2Cl_2. The resulting material was purified by flash column chromatography with 50% → 100% EtOAc in pentanes to give 137.0 mg (0.26 mmol, 52% yield) of two diastereomers (5:1 d.r., separated under these conditions). Spectroscopic data were consistent with those reported above.

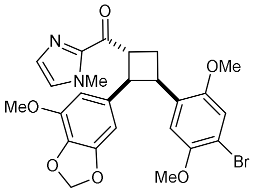

6. Cleavage Reaction of Complex Cycloadduct

Methyl 3-(4-bromo-2,5-dimethoxyphenyl)-2-(7-methoxy-2*H*-1,3-benzodioxol-5-yl)cyclobutane-1-carboxylate (37) A flame-dried 6 mL vial with a stir bar was charged with 100.5 mg (0.19 mmol, 1.0 equiv.) of the major diastereomer of cycloadduct **36** and 1.5 mL dry CH_2Cl_2, and the solution stirred under N_2 for 10 min. To this was added 30 µL (0.25 mmol, 1.3 equiv.) freshly distilled MeOTf, and the reaction mixture stirred for 3 h at room temperature. This solution was then concentrated *in vacuo* and dried under vacuum for 30 min to remove any remaining MeOTf. The resulting yellow foam was then taken up in 1.5 mL dry CH_2Cl_2, to which was added 0.32 mL (8.0 mmol, 40.0 equiv.) distilled MeOH and 4.5 mg (0.04 mmol, 0.2 equiv.) 1,4-diazabicyclo[2.2.2]octane (DABCO), and the reaction mixture stirred for 3 h at room temperature. The reaction mixture was concentrated and purified by flash column chromatography with 10% to 50% EtOAc/pentanes to give 63.4 mg (0.13 mmol, 70% yield) of a viscous semisolid. 92% ee [Daicel CHIRALPAK AD, 5% to 50% iPrOH, 20 minutes, 1 mL/min, t_1=9.30 min, t_2=10.73 min] $[\alpha]_D^{22}$ –64.2° (c1.090, CH_2Cl_2). ^{1}H NMR (500 MHz, $CDCl_3$) δ 6.79 (s, 1H) , 6.76 (s, 1H), 6.19 (d, *J* = 1.1 Hz, 1H), 6.01 (d, *J* = 1.1 Hz, 1H), 5.86–5.85 (m, 2H), 4.19 (td, *J* = Hz, 1H), 4.13 (t, *J* = 8.8 Hz, 1H), 3.81 (s, 3H), 3.73 (s, 3H), 3.68 (s, 3H), 3.49 (s, 3H), 3.43 (q, *J* = 8.5 Hz, 1H), 2.72 (dt, *J* = 12.1, 8.5 Hz, 1H), 2.57–2.51 (m, 1H). ^{13}C NMR (125 MHz, $CDCl_3$) δ 174.98, 152.04, 149.83, 148.18, 142.79, 134.38, 133.47, 129.21, 115.26, 112.33, 109.26, 106.87, 101.75, 101.16, 57.33, 56.32, 55.59, 51.98, 47.51, 41.52, 36.76, 25.53. HRMS (ESI) calculated for $[C_{22}H_{27}BrNO_7]^+$ ($M+NH_4^+$) requires *m/z* 496.0965, found 496.0965.

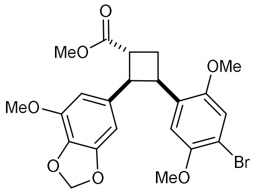

Methyl 3-(4-bromo-2,5-dimethoxyphenyl)-2-(7-methoxy-2*H*-1,3-benzodioxol-5-yl)cyclobutane-1-carboxylate ((±)-37) Racemic material prepared according to the procedure above for the analogous enriched product (±)-**36** using 86.2 mg (0.16 mmol, 1.0 equiv.) (3-(4-bromo-2,5-dimethoxyphenyl)-2-(7-methoxy-2*H*-1,3-benzodioxol-5-yl)cyclobutyl)(1-methyl-1*H*-imidazol-2-yl)methanone and 25 µL (0.20 mmol, 1.3 equiv.) MeOTf in 1 mL CH_2Cl_2, and then 0.25 mL (6.0 mmol, 40.0 equiv.) MeOH, and 3.5 mg (0.3 mmol, 0.2 equiv.) DABCO in 1 mL CH_2Cl_2. The resulting material was purified by flash column chromatography with 10% to 50% EtOAc in pentanes to give 12.0 mg (0.03 mmol, 15% yield) of two diastereomers (5:1 d.r., separated under these conditions). Spectroscopic data were consistent with those reported above.

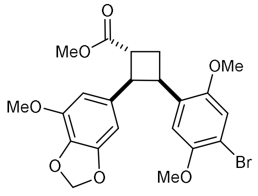

7. Mechanistic Experiments

7.1 Absorbance Data

Supplementary Figure 1. Comparative absorption spectra of the Brønsted acid catalyst (**AC-3**), substrate (**1**), and 20 and 100 mol% loading of acid catalyst in substrate dissolved in CH_2Cl_2. The spectra were all recorded in quartz tubes under ambient conditions.

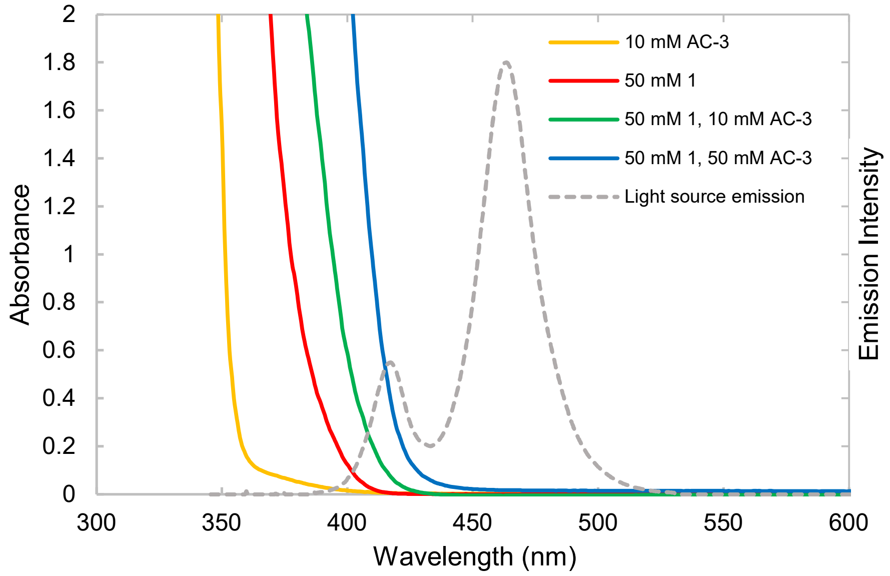

7.2 Stereoconvergence Experiments

Experimental Details: *cis*-Deuterostyrene (99% deuterium incorporation) and *trans*-deuterostyrene (92% deuterium incorporation) were prepared via hydrozirconation as reported by Russel.[22]

Reaction using *cis*-deuterostyrene (A): Reactions were prepared according to the general photochemical procedure for racemic experiments using 21.9 mg (0.10 mmol) 2-cinnamoyl-1-methylimidazole, 0.12 mL (1.0 mmol) *cis*-deuterostyrene, 16.5 mg (0.02 mmol) **(±)-A1**, and 2 mL toluene. The resulting material was purified by flash column chromatography using 1:1 Et_2O/pentanes to give 20.1 mg (0.06 mmol, 61% yield) of three diastereomers (5:1 d.r.).

Reaction using *trans*-deuterostyrene (B): Reactions were prepared according to the general photochemical procedure for racemic experiments using 22.1 mg (0.10 mmol) 2-cinnamoyl-1-methylimidazole, 0.12 mL (1.0 mmol) *trans*-deuterostyrene, 16.2 mg (0.02 mmol) **(±)-A1**, and 2 mL toluene. The resulting material was purified by flash column chromatography using 1:1 Et_2O/pentanes to give 20.4 mg (0.06 mmol, 62% yield) of three diastereomers (5:1 d.r.).

Supplementary Figure 2. Stereoconvergence experiments using (**A**) *cis*-deuterostyrene and (**B**) *trans*-deuterostyrene

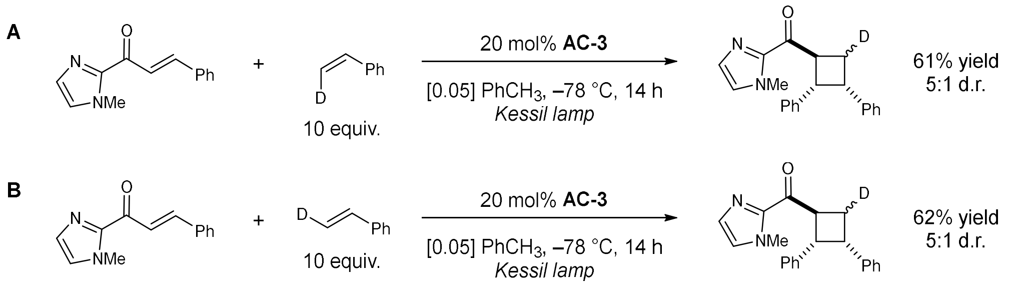

Supplementary Figure 3. ^{1}H NMRs of [2+2] cycloadduct mixture resulting from reaction with *cis*-deuterostyrene (spectrum 2) and *trans*-deuterostyrene (spectrum 1). Diastereomers of **14** are also shown.

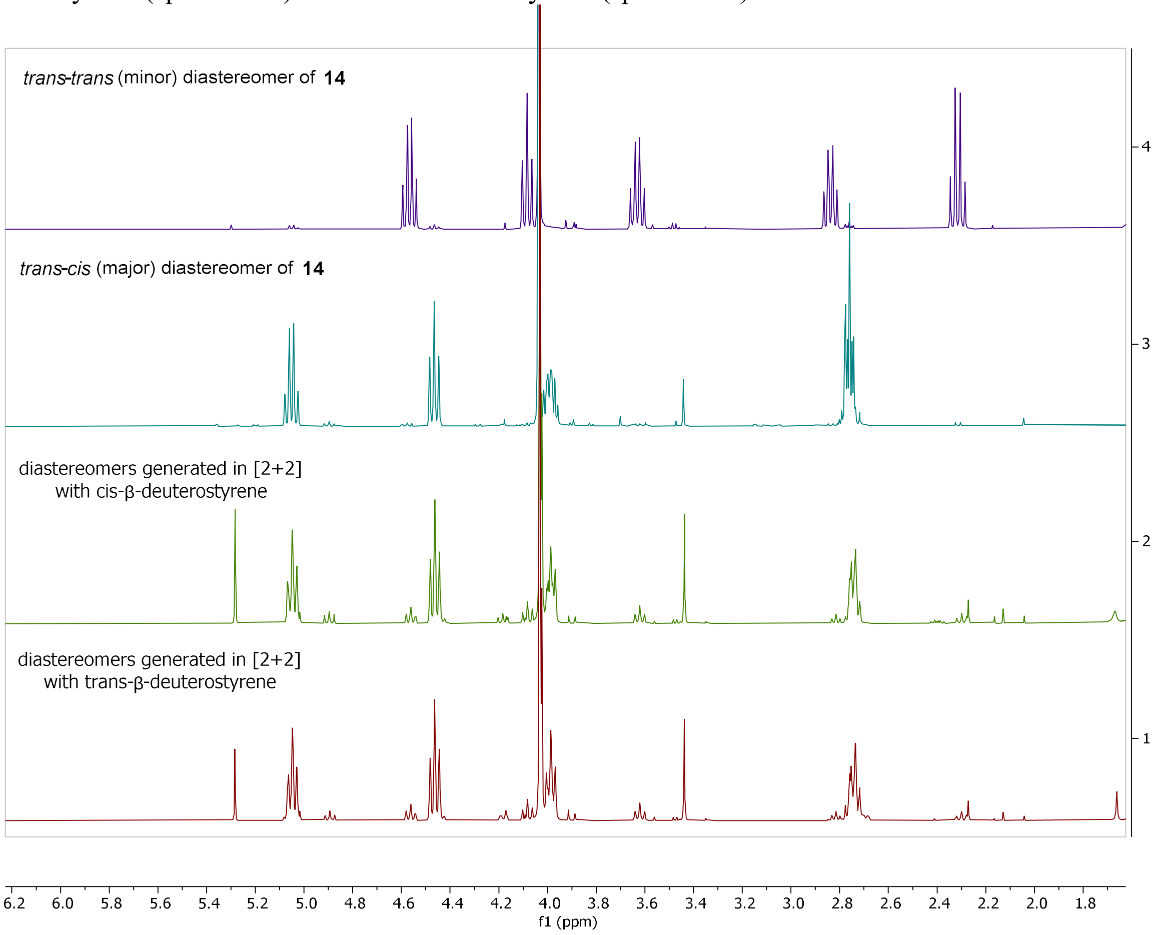

Supplementary Figure 4. Expanded ^{1}H NMRs of [2+2] cycloadduct mixture resulting from reaction with *cis*-deuterostyrene (spectrum 2) and *trans*-deuterostyrene (spectrum 1). Diastereomers of **14** are also shown.

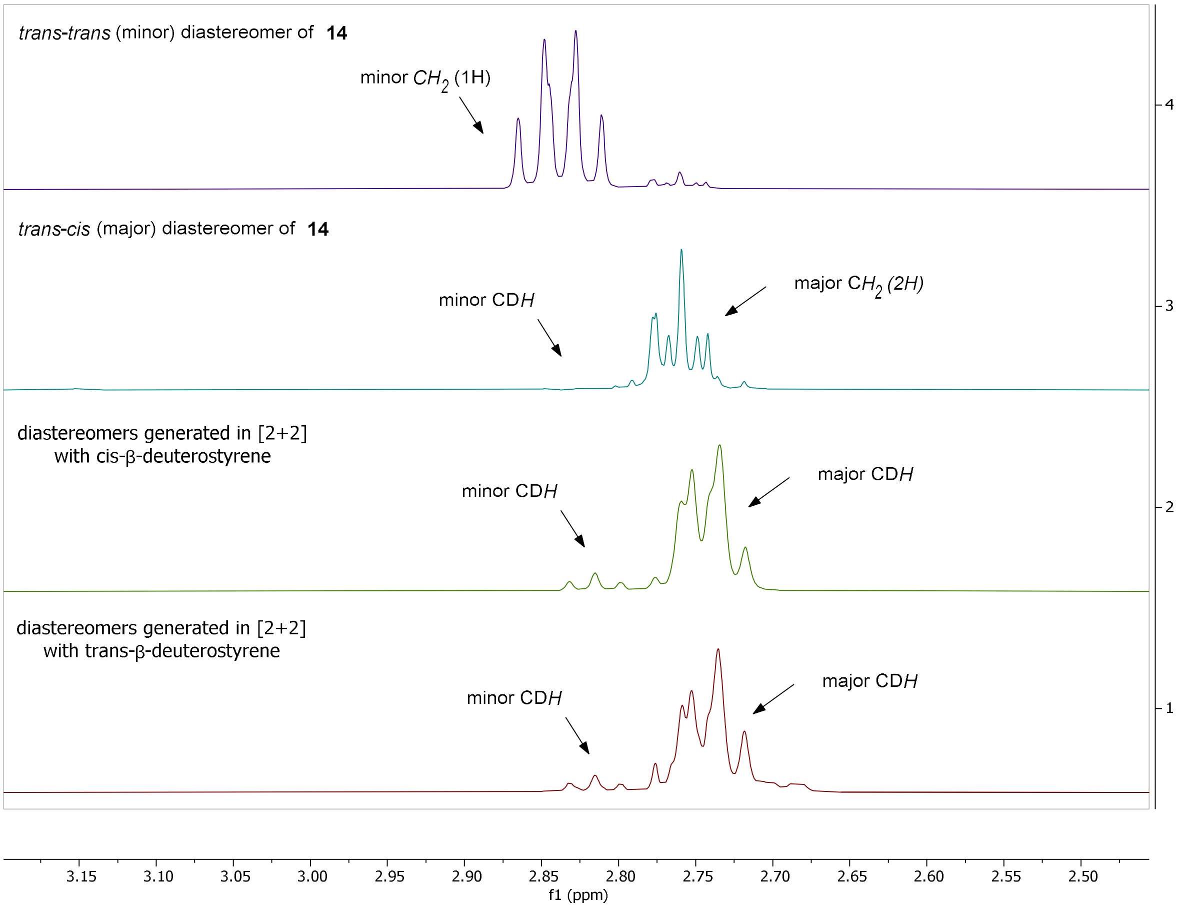

7.3 NMR Titration Experiments

Experimental Details: A stock solution of **AC-3** (11.0 mg, 0.014 mmol) was prepared in *d*8-toluene (700 µL) and added to a solution of **1** (2.1 mg, 0.01 mmol) in *d*8-toluene (500 µL) in a 7" NMR tube in 50 µL increments. Then a more concentrated stock solution of **AC-3** (94.6 mg, 0.12 mmol), prepared in *d*8-toluene (1.2 mL), was added to the same tube in 200 µL increments. ^{1}H NMR spectra were acquired on a 600 mHz instrument with a DCI-F cryoprobe. The titration was quantified by monitoring the chemical shift of the imidazole methyl protons (~3.4 ppm in **1** alone) of the substrate, which moved upfield with increasing **AC-3**. When multiple peaks appeared, chemical shifts were averaged.

Supplementary Figure 5. Stacked NMR spectra of titration of 0 to 11 equiv. of **AC-3** into **1**

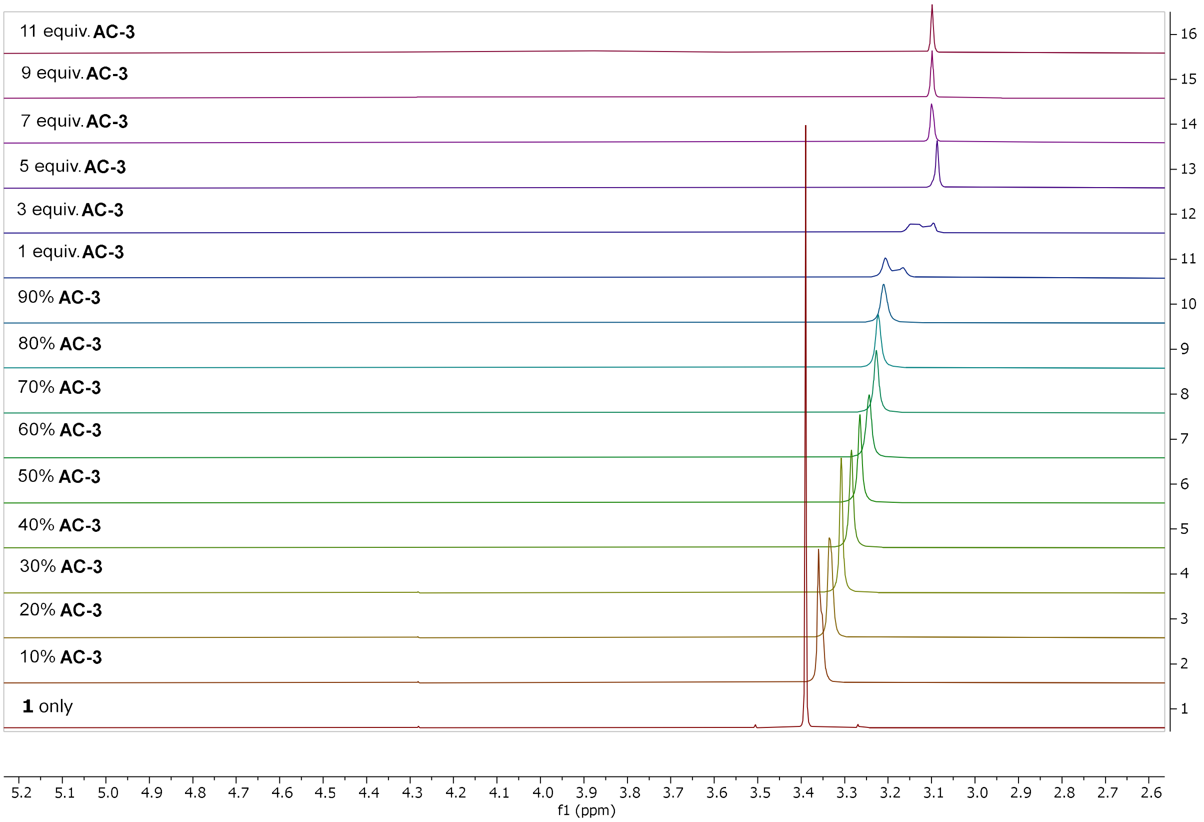

Supplementary Figure 6. Preliminary binding isotherm, measured vs. calculated

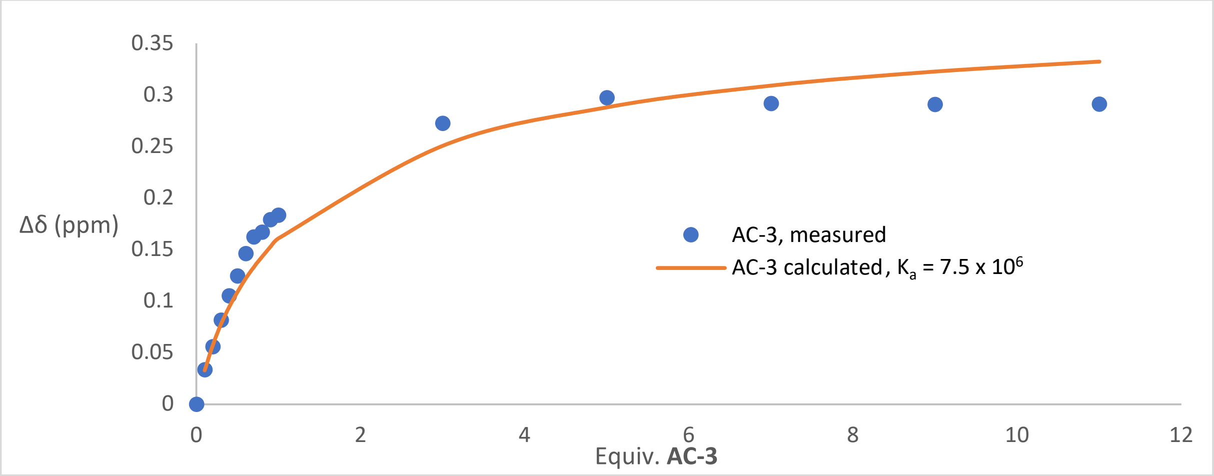

These data were fit to a 1:1 binding model following the method developed by Thordarson.[23] Microsoft Excel was used to estimate K_a by minimizing difference between experiment and predicted chemical shifts using nonlinear regression analysis.

8. Assignment of Diastereomers by 1D-NOE

Supplementary Figure 7. Observed nOe Enhancements

26 - Major Diastereomer(**1.2**:1)
(in $CDCl_3$)

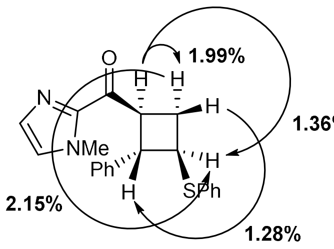

26 - Major Diastereomer(1.2:**1**)
(in $CDCl_3$)

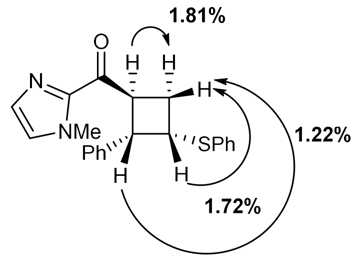

30 - Major Diastereomer(**10**:1)
(in $CDCl_3$)

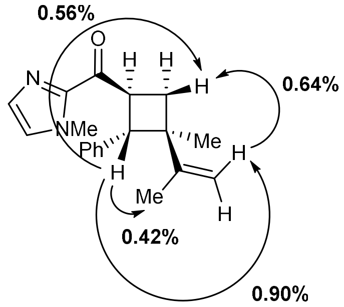

31 - Major Diastereomer(**3**:1)
(in $CDCl_3$)

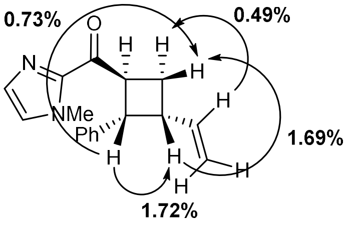

31 - Minor Diastereomer(3:**1**)
(in $CDCl_3$)

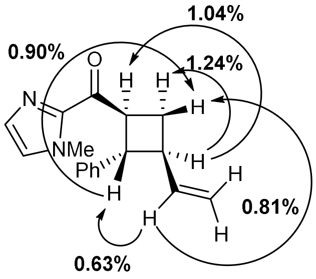

36 - Major Diastereomer(**5**:1)
(in C_6D_6)

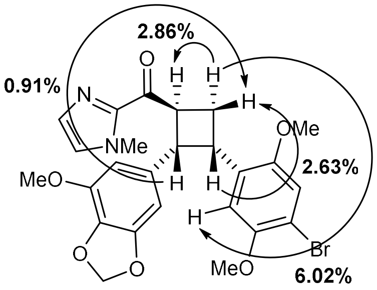

36 - Minor Diastereomer(5:**1**)
(in C_6D_6)

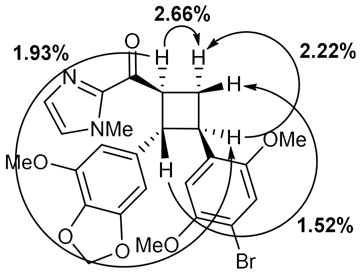

9. X-Ray Crystallographic Data

CCDC-2022776 and CCDC-2022777 contain full crystallographic data for the structures described in this manuscript. These data can be obtained free of charge from the Cambridge Crystallographic Data Centre.

((1R,2R,3R)-3-(4-Bromophenyl)-2-phenylcyclobutyl)(1-methyl-1H-imidazol-2-yl)methanone (18)

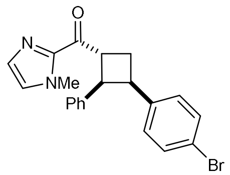

Data Collection: A colorless crystal with approximate dimensions 0.12 × 0.08 × 0.06 mm^3 was selected under oil under ambient conditions and attached to the tip of a MiTeGen MicroMount©. The crystal was mounted in a stream of cold nitrogen at 100(1) K and centered in the X-ray beam by using a video camera.

The crystal evaluation and data collection were performed on a Bruker D8 VENTURE PhotonIII four-circle diffractometer with Cu Kα (λ = 1.54178 Å) radiation and the detector to crystal distance of 4.0 cm.[24]

The initial cell constants were obtained from a 180° φ scan conducted at a 2θ = 50° angle with the exposure time of 1 second per frame. The reflections were successfully indexed by an automated indexing routine built in the APEX3 program. The final cell constants were calculated from a set of 9313 strong reflections from the actual data collection.

The data were collected by using the full sphere data collection routine to survey the reciprocal space to the extent of a full sphere to a resolution of 0.80 Å. A total of 67343 data were harvested by collecting 34 sets of frames with 0.5º scans in ω and φ with an exposure time 0.5-8.3 sec per frame. These highly redundant datasets were corrected for Lorentz and polarization effects. The absorption correction was based on fitting a function to the empirical transmission surface as sampled by multiple equivalent measurements.[25]

Structure Solution and Refinement: The systematic absences in the diffraction data were uniquely consistent for the space group $P2_12_12_1$ that yielded chemically reasonable and computationally stable results of refinement.[26–31]

A successful solution by the direct methods provided most non-hydrogen atoms from the *E*-map. The remaining non-hydrogen atoms were located in an alternating series of least-squares cycles and difference Fourier maps. All non-hydrogen atoms were refined with anisotropic displacement coefficients. All hydrogen atoms were included in the structure factor calculation at idealized positions and were allowed to ride on the neighboring atoms with relative isotropic displacement coefficients.

The absolute configuration was established based on resonant scattering effects: the three chiral centers C7, C8, C15 are all *R*. The crystal may contain 1.4(17) % of the opposite enantiomer.

The final least-squares refinement of 228 parameters against 3859 data resulted in residuals *R* (based on F^2 for $I \geq 2\sigma$) and *wR* (based on F^2 for all data) of 0.0192 and 0.0505, respectively. The final difference Fourier map was featureless.

Summary

Crystal Data for $C_{21}H_{19}BrN_2O$ (M=395.29 g/mol): orthorhombic, space group $P2_12_12_1$ (no. 19), a = 8.1192(6) Å, b = 10.5977(10) Å, c = 20.6785(17) Å, V = 1779.3(3) Å^3, Z = 4, T = 100.0 K, μ(CuKα) = 3.226 mm^{-1}, $Dcalc$ = 1.476 g/cm^3, 67343 reflections measured (8.552° ≤ 2Θ ≤ 159.016°), 3859 unique (R_{int} = 0.0257, R_{sigma} = 0.0109) which were used in all calculations. The final R_1 was 0.0192 (I > 2σ(I)) and wR_2 was 0.0505 (all data).

Supplementary Figure 8. A molecular drawing of yoon61 shown with 50% probability ellipsoids

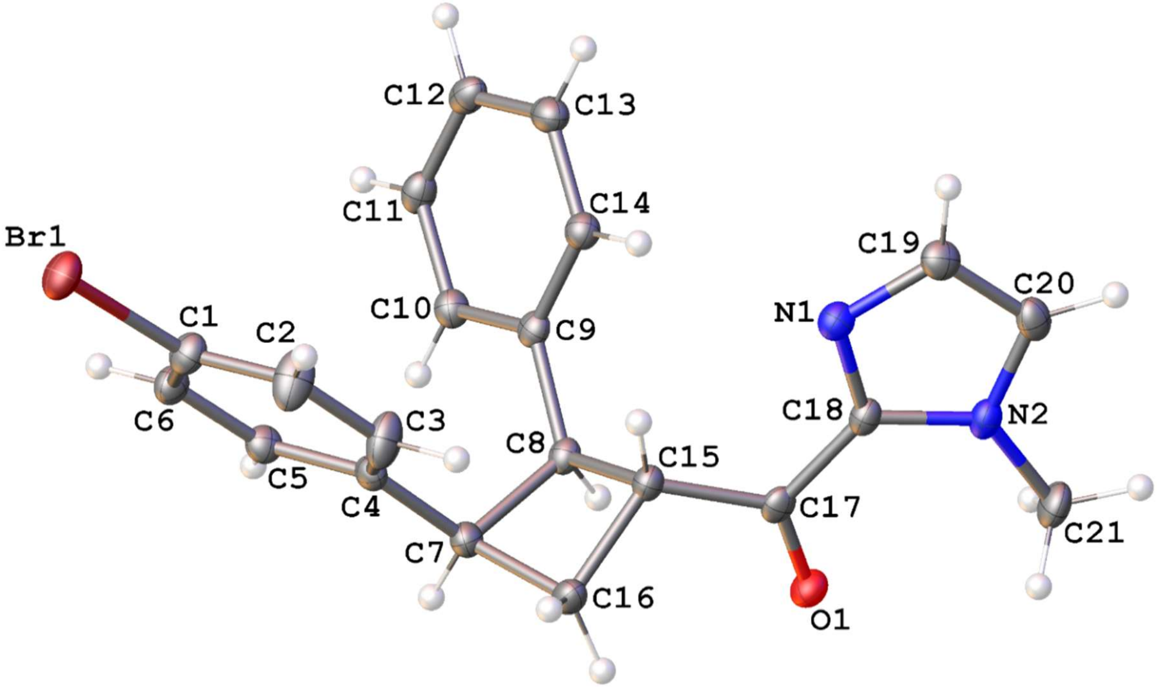

Supplementary Table 1. Crystal data and structure refinement for yoon61

Identification code	yoon61
Empirical formula	$C_{21}H_{19}BrN_2O$
Formula weight	395.29
Temperature/K	100.0
Crystal system	orthorhombic
Space group	$P2_12_12_1$
a/Å	8.1192(6)
b/Å	10.5977(10)
c/Å	20.6785(17)
α/°	90
β/°	90
γ/°	90
Volume/Å^3	1779.3(3)
Z	4
ρ_{calc}g/cm^3	1.476

μ/mm^{-1}	3.226
F(000)	808.0
Crystal size/mm^3	0.12 × 0.08 × 0.06
Radiation	CuKα (λ = 1.54178)
2Θ range for data collection/°	8.552 to 159.016
Index ranges	-10 ≤ h ≤ 10, -11 ≤ k ≤ 13, -26 ≤ l ≤ 25
Reflections collected	67343
Independent reflections	3859 [R_{int} = 0.0257, R_{sigma} = 0.0109]
Data/restraints/parameters	3859/0/228
Goodness-of-fit on F^2	1.049
Final R indexes [I>=2σ (I)]	R_1 = 0.0192, wR_2 = 0.0505
Final R indexes [all data]	R_1 = 0.0192, wR_2 = 0.0505
Largest diff. peak/hole / e Å^{-3}	0.52/-0.41
Flack parameter	0.014(17)

Supplementary Table 2. Fractional Atomic Coordinates (×10^4) and Equivalent Isotropic Displacement Parameters (Å^2×10^3) for yoon61. U_{eq} is defined as 1/3 of of the trace of the orthogonalised U_{IJ} tensor.

**Atom**	***x***	***y***	***z***	**U(eq)**
Br1	5073.7(3)	5024.5(2)	167.5(2)	24.31(7)
O1	3736.3(19)	-3544.1(13)	1740.6(7)	20.9(3)
N1	568(2)	-2460.4(16)	674.6(8)	18.2(3)
N2	691(2)	-4349.4(16)	1146.7(8)	18.0(3)
C1	5111(3)	3450.2(18)	611.7(9)	20.7(4)
C2	4851(4)	2351(2)	267.3(10)	31.0(5)
C3	4931(4)	1195.7(19)	588.0(10)	28.0(5)
C4	5247(2)	1129.8(18)	1248.8(9)	16.8(4)
C5	5489(2)	2258.5(19)	1580.6(9)	18.5(4)
C6	5429(3)	3422.8(19)	1270.3(10)	19.3(4)
C7	5354(2)	-98.1(19)	1616.4(9)	17.9(3)
C8	3642(2)	-624.0(18)	1859.3(9)	16.2(4)
C9	2248(2)	271.1(17)	1999.3(9)	15.9(4)
C10	2384(3)	1114.7(19)	2518.3(10)	18.2(4)
C11	1135(3)	1975.6(19)	2648.7(10)	19.5(4)
C12	-265(3)	2004.8(19)	2266.8(10)	20.9(4)
C13	-414(3)	1167.4(19)	1748.8(10)	21.0(4)
C14	833(2)	307.2(18)	1618.0(10)	18.4(4)
C15	3602(2)	-1508.8(18)	1253.4(10)	16.3(4)
C16	5504(3)	-1318.7(19)	1206.9(11)	19.7(4)
C17	3003(2)	-2835.5(18)	1377.4(9)	16.5(4)
C18	1456(2)	-3209.7(18)	1060.6(9)	16.4(4)
C19	-805(3)	-3132(2)	523.5(10)	20.5(4)
C20	-749(3)	-4301(2)	812.4(10)	20.6(4)
C21	1208(3)	-5428.5(19)	1537.1(11)	25.1(4)

Supplementary Table 3. Anisotropic Displacement Parameters (Å^2×10^3) for yoon61. The Anisotropic displacement factor exponent takes the form: $-2\pi^2[h^2a^{*2}U_{11}+2hka^*b^*U_{12}+\ldots]$.

Atom	U_{11}	U_{22}	U_{33}	U_{23}	U_{13}	U_{12}
Br1	35.55(12)	17.60(11)	19.78(11)	4.01(7)	0.43(8)	-0.57(11)
O1	23.3(7)	16.5(7)	22.8(7)	2.7(5)	-2.6(6)	1.3(6)
N1	21.7(8)	17.3(8)	15.6(7)	0.1(6)	-0.3(6)	0.3(6)
N2	22.4(8)	13.9(8)	17.8(8)	-0.9(6)	3.4(6)	-1.5(7)
C1	25.9(10)	16.8(8)	19.5(9)	2.8(7)	1.8(9)	-0.1(8)
C2	54.4(15)	23.5(10)	15.1(8)	1.2(8)	-4.3(10)	-6.8(11)
C3	47.0(13)	18.0(9)	19.0(9)	-2.2(7)	-1.0(10)	-7.0(11)
C4	14.4(9)	16.8(8)	19.2(8)	1.5(7)	0.3(8)	-2.5(7)
C5	19.8(9)	19.7(9)	16.1(8)	-1.0(7)	-2.2(7)	-0.9(7)
C6	22.2(10)	16.2(9)	19.4(9)	-2.6(7)	-1.1(8)	-0.3(7)
C7	16.7(8)	16.3(8)	20.8(8)	-0.4(7)	-1.6(7)	-1.3(8)
C8	19.9(9)	13.6(8)	15.0(8)	1.4(7)	-1.2(7)	-0.9(8)
C9	18.9(9)	13.5(9)	15.4(8)	2.9(7)	1.7(7)	-1.0(7)
C10	20.0(9)	18.1(9)	16.7(9)	0.6(8)	-0.8(7)	-2.0(8)
C11	24.0(10)	16.4(9)	18.1(9)	-1.4(7)	5.7(8)	-1.2(8)
C12	21.3(10)	16.5(8)	24.8(9)	3.2(7)	6.8(8)	1.5(8)
C13	18.1(9)	20.4(9)	24.4(9)	3.1(8)	-0.8(8)	0.1(8)
C14	19.9(9)	16.9(10)	18.6(9)	0.7(7)	-0.6(7)	-0.7(7)
C15	18.6(9)	13.2(9)	17.1(9)	-0.3(7)	0.5(7)	-0.5(7)
C16	17.8(10)	15.7(9)	25.5(10)	0.1(8)	3.2(8)	0.6(7)
C17	20.2(9)	13.9(8)	15.6(9)	-2.9(7)	3.7(7)	1.6(7)
C18	20.3(9)	12.8(9)	16.2(9)	-0.4(7)	2.4(7)	-0.3(7)
C19	22.2(10)	22.1(10)	17.3(9)	-3.1(8)	-1.5(8)	-0.2(8)
C20	21.4(10)	20.5(10)	20.0(9)	-5.7(8)	2.4(8)	-4.5(8)
C21	31.4(11)	14.2(9)	29.8(11)	3.1(8)	1.6(9)	-2.4(8)

Supplementary Table 4. Bond Lengths for yoon61

Atom	Atom	Length/Å	Atom	Atom	Length/Å
Br1	C1	1.9047(19)	C7	C8	1.580(3)
O1	C17	1.218(2)	C7	C16	1.551(3)
N1	C18	1.337(3)	C8	C9	1.505(3)
N1	C19	1.359(3)	C8	C15	1.565(3)
N2	C18	1.370(2)	C9	C10	1.401(3)
N2	C20	1.359(3)	C9	C14	1.394(3)
N2	C21	1.461(3)	C10	C11	1.390(3)
C1	C2	1.381(3)	C11	C12	1.384(3)
C1	C6	1.386(3)	C12	C13	1.396(3)
C2	C3	1.394(3)	C13	C14	1.389(3)
C3	C4	1.392(3)	C15	C16	1.560(3)
C4	C5	1.393(3)	C15	C17	1.510(3)
C4	C7	1.510(3)	C17	C18	1.471(3)
C5	C6	1.392(3)	C19	C20	1.376(3)

Supplementary Table 5. Bond Angles for yoon61

Atom	Atom	Atom	Angle/°	Atom	Atom	Atom	Angle/°
C18	N1	C19	105.57(17)	C10	C9	C8	119.36(17)
C18	N2	C21	129.16(18)	C14	C9	C8	121.89(17)
C20	N2	C18	106.89(17)	C14	C9	C10	118.73(18)
C20	N2	C21	123.89(18)	C11	C10	C9	120.66(19)
C2	C1	Br1	119.16(15)	C12	C11	C10	120.18(19)
C2	C1	C6	121.15(18)	C11	C12	C13	119.65(19)
C6	C1	Br1	119.68(15)	C14	C13	C12	120.21(19)
C1	C2	C3	119.22(18)	C13	C14	C9	120.58(18)
C4	C3	C2	121.30(18)	C16	C15	C8	87.22(15)
C3	C4	C5	117.80(18)	C17	C15	C8	115.39(16)
C3	C4	C7	123.24(17)	C17	C15	C16	116.71(16)
C5	C4	C7	118.96(17)	C7	C16	C15	89.78(15)
C6	C5	C4	121.96(18)	O1	C17	C15	121.44(18)
C1	C6	C5	118.56(18)	O1	C17	C18	121.73(18)
C4	C7	C8	114.43(15)	C18	C17	C15	116.78(17)
C4	C7	C16	116.66(15)	N1	C18	N2	110.89(17)
C16	C7	C8	87.05(14)	N1	C18	C17	124.49(17)
C9	C8	C7	120.04(16)	N2	C18	C17	124.55(18)
C9	C8	C15	121.06(16)	N1	C19	C20	110.17(19)
C15	C8	C7	88.57(14)	N2	C20	C19	106.46(18)

Supplementary Table 6. Torsion Angles for yoon61

A	B	C	D	Angle/°	A	B	C	D	Angle/°
Br1	C1	C2	C3	177.9(2)	C8	C15	C17	O1	-62.3(2)
Br1	C1	C6	C5	-178.48(16)	C8	C15	C17	C18	115.14(19)
O1	C17	C18	N1	177.69(19)	C9	C8	C15	C16	144.89(18)
O1	C17	C18	N2	1.1(3)	C9	C8	C15	C17	-96.7(2)
N1	C19	C20	N2	-0.2(2)	C9	C10	C11	C12	0.2(3)
C1	C2	C3	C4	0.8(4)	C10	C9	C14	C13	0.3(3)
C2	C1	C6	C5	0.3(4)	C10	C11	C12	C13	-0.2(3)
C2	C3	C4	C5	-0.3(4)	C11	C12	C13	C14	0.1(3)
C2	C3	C4	C7	-179.7(2)	C12	C13	C14	C9	-0.2(3)
C3	C4	C5	C6	-0.3(3)	C14	C9	C10	C11	-0.3(3)
C3	C4	C7	C8	-85.6(3)	C15	C8	C9	C10	-175.22(17)
C3	C4	C7	C16	13.9(3)	C15	C8	C9	C14	3.3(3)
C4	C5	C6	C1	0.3(3)	C15	C17	C18	N1	0.2(3)
C4	C7	C8	C9	-27.8(2)	C15	C17	C18	N2	-176.36(17)
C4	C7	C8	C15	97.73(17)	C16	C7	C8	C9	-145.87(17)
C4	C7	C16	C15	-95.58(18)	C16	C7	C8	C15	-20.31(14)
C5	C4	C7	C8	95.1(2)	C16	C15	C17	O1	38.0(3)
C5	C4	C7	C16	-165.46(18)	C16	C15	C17	C18	-144.57(18)
C6	C1	C2	C3	-0.9(4)	C17	C15	C16	C7	-137.69(17)
C7	C4	C5	C6	179.14(18)	C18	N1	C19	C20	-0.6(2)

C7	C8	C9	C10	-66.9(2)	C18	N2	C20	C19	0.9(2)
C7	C8	C9	C14	111.6(2)	C19	N1	C18	N2	1.2(2)
C7	C8	C15	C16	20.17(14)	C19	N1	C18	C17	-175.82(18)
C7	C8	C15	C17	138.53(17)	C20	N2	C18	N1	-1.3(2)
C8	C7	C16	C15	20.36(14)	C20	N2	C18	C17	175.69(18)
C8	C9	C10	C11	178.27(17)	C21	N2	C18	N1	-178.76(19)
C8	C9	C14	C13	-178.25(18)	C21	N2	C18	C17	-1.8(3)
C8	C15	C16	C7	-20.56(14)	C21	N2	C20	C19	178.49(19)

Supplementary Table 7. Hydrogen Atom Coordinates (Å×10^4) and Isotropic Displacement Parameters (Å^2×10^3) for yoon61

**Atom**	***x***	***y***	***z***	**U(eq)**
H2	4619.53	2382.77	-182.81	37
H3	4766.55	438.29	350.83	34
H5	5700.8	2232.8	2032.21	22
H6	5601.23	4182.29	1504.7	23
H7	6190.32	-63.96	1970.92	22
H8	3837.21	-1153.89	2252.08	19
H10	3338.36	1098.38	2783.72	22
H11	1242.39	2545.24	3000.88	23
H12	-1119.3	2591.64	2356.56	25
H13	-1371.71	1185.85	1485.01	25
H14	721.02	-261.34	1265.57	22
H15	3012.63	-1108.6	881.57	20
H16A	6147.7	-1994.33	1420.14	24
H16B	5902.89	-1170.14	760.94	24
H19	-1677.08	-2837.7	257.02	25
H20	-1557.85	-4946.31	783.94	25
H21A	2335.79	-5666.13	1420.27	38
H21B	467.27	-6141.6	1456.51	38
H21C	1167.21	-5201.51	1996.22	38

Equimolar (1:1) Complex of 1 and AC-3

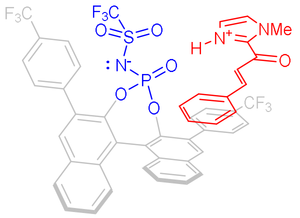

Data Collection: A yellow crystal with approximate dimensions 0.38 × 0.32 × 0.25 mm^3 was selected under oil under ambient conditions and attached to the tip of a MiTeGen MicroMount©. The crystal was mounted in a stream of cold nitrogen at 100(1) K and centered in the X-ray beam by using a video camera.

The crystal evaluation and data collection were performed on a Bruker D8 VENTURE PhotonIII four-circle diffractometer with Cu Kα (λ = 1.54178 Å) radiation and the detector to crystal distance of 4.0 cm.[24]

The initial cell constants were obtained from a 180° φ scan conducted at a 2θ = 50° angle with the exposure time of 1 second per frame. The reflections were successfully indexed by an automated indexing routine built in the APEX3 program. The final cell constants were calculated from a set of 9509 strong reflections from the actual data collection.

The data were collected by using the full sphere data collection routine to survey the reciprocal space to the extent of a full sphere to a resolution of 0.78 Å. A total of 203898 data were harvested by collecting 43 sets of frames with 0.9º scans in ω and φ with an exposure time 2.4 – 4 sec per frame. These highly redundant datasets were corrected for Lorentz and polarization effects. The absorption correction was based on fitting a function to the empirical transmission surface as sampled by multiple equivalent measurements.[25]

Structure Solution and Refinement: The systematic absences in the diffraction data were uniquely consistent for the space group $P2_12_12_1$ that yielded chemically reasonable and computationally stable results of refinement.[26–31]

A successful solution by the direct methods provided most non-hydrogen atoms from the *E*-map. The remaining non-hydrogen atoms were located in an alternating series of least-squares cycles and difference Fourier maps. All non-hydrogen atoms were refined with anisotropic displacement coefficients unless specified otherwise. All hydrogen atoms except H3(N3) were included in the structure factor calculation at idealized positions and were allowed to ride on the neighboring atoms with relative isotropic displacement coefficients.

The crystal structure consists of a $C_{35}H_{18}F_9NO_5PS$ anion, $C_{13}H_{13}N_2O$ cation, and two molecules of solvent Et_2O.

There is positional disorder in the structure. The CF_3 group at C7 is disordered over three positions in a 55.2(4):29.7(4):15.1(4) ratio. The disordered CF_3 groups were refined with restraints and constraints. The O7 diethyl ether is disordered over two positions with the major component contribution of 83.7(4) %. The minor disorder component was refined isotropically with geometry restraints. The O8 diethyl ether is disordered over two positions with the major component contribution of 64.7(13) %. Both disorder components were refined isotropically with geometry and atomic displacement parameter restraints.

The absolute structure was unequivocally established based on resonant scattering effects. The crystal is an inversion twin with a 2.3(11) % contribution of the inverted component.

The final least-squares refinement of 804 parameters against 11554 data resulted in residuals R (based on F^2 for $I \geq 2\sigma$) and wR (based on F^2 for all data) of 0.0266 and 0.0685, respectively. The final difference Fourier map was featureless.

Summary

Crystal Data for $C_{56}H_{51}F_9N_3O_8PS$ (M =1128.03 g/mol): orthorhombic, space group $P2_12_12_1$ (no. 19), a = 13.6603(16) Å, b = 16.608(2) Å, c = 23.617(3) Å, V = 5358.1(12) Å^3, Z = 4, T = 100.0 K, μ(CuKα) = 1.594 mm^{-1}, $Dcalc$ = 1.398 g/cm^3, 203898 reflections measured (6.506° ≤ 2Θ ≤ 158.416°), 11554 unique (R_{int} = 0.0437, R_{sigma} = 0.0215) which were used in all calculations. The final R_1 was 0.0266 (I > 2σ(I)) and wR_2 was 0.0685 (all data).

Supplementary Figure 9. A molecular drawing of the hydrogen bonding interactions between the cation and anion in yoon62a shown with 50% probability ellipsoids. Selected H atoms are shown. Minor disorder components and solvent molecules are omitted.

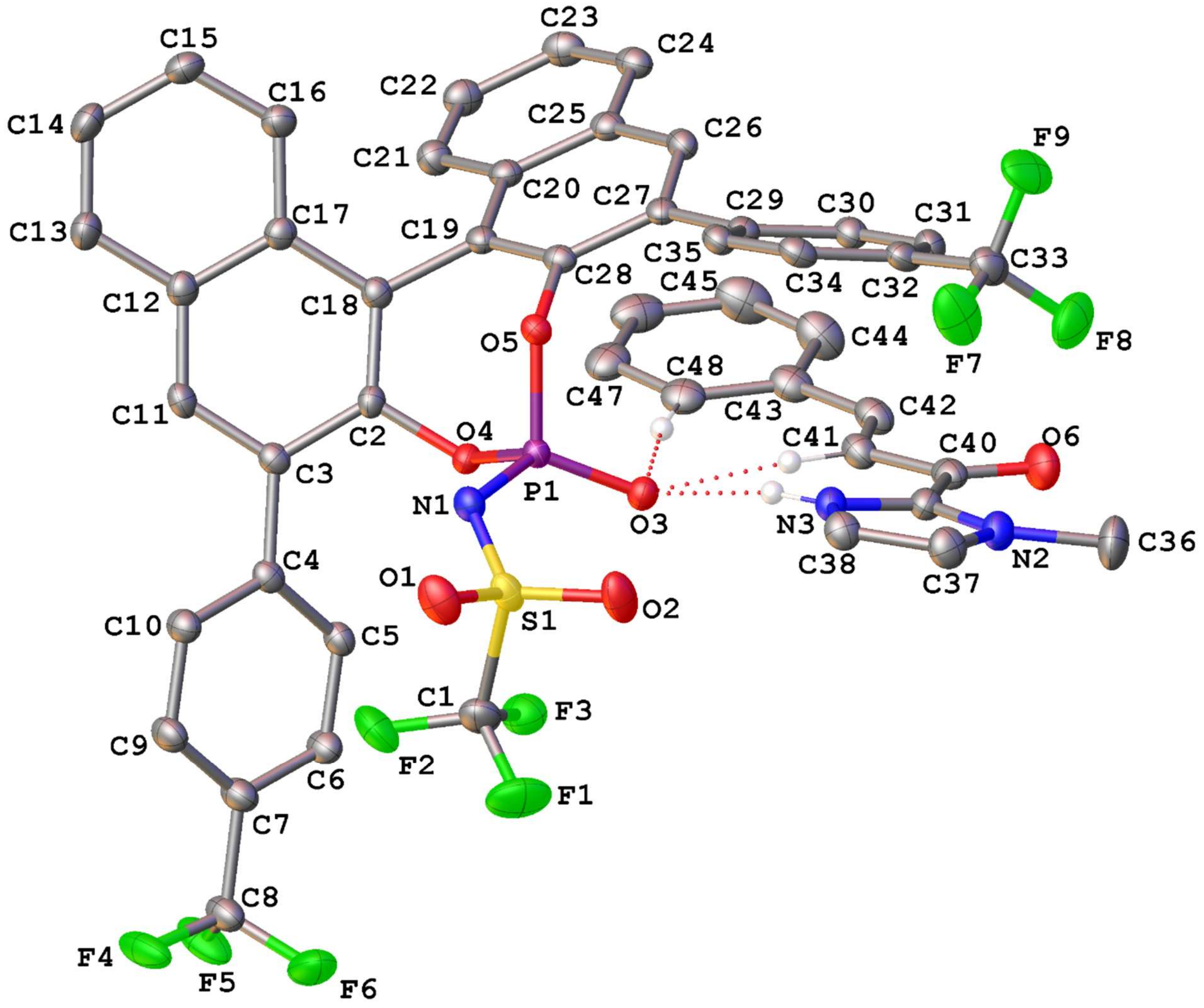

Supplementary Figure 10. A molecular drawing of the hydrogen bonding interactions between the cation and anion in yoon62a shown with 50% probability ellipsoids. Selected H atoms are shown. Minor disorder components and solvent molecules are omitted. (Same as Supplementary Figure 8, just fewer labels).

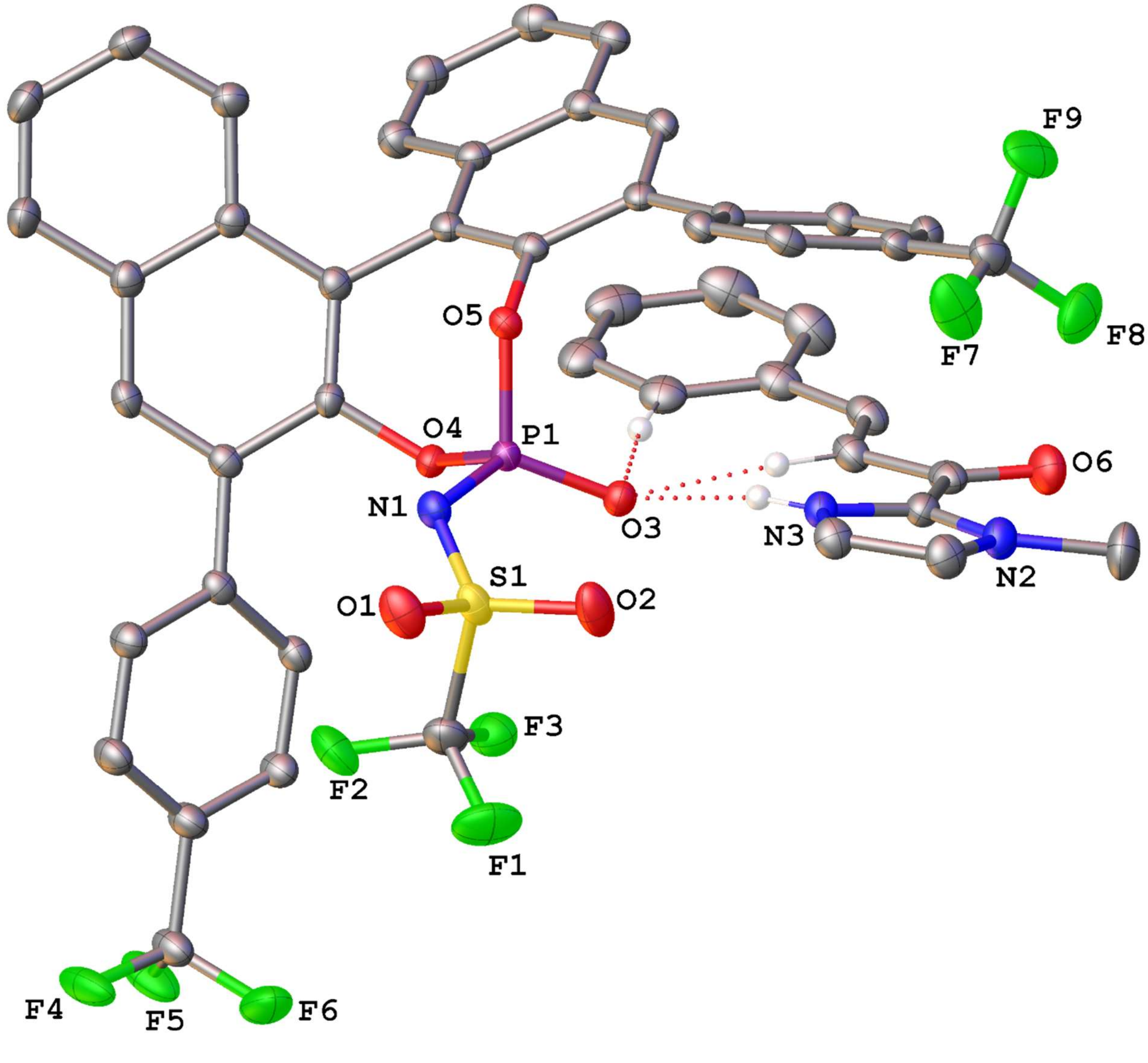

Supplementary Figure 11. A molecular drawing of the anion in yoon62a shown with 50% probability ellipsoids. All disorder components at C7 are shown whereas all H atoms are omitted.

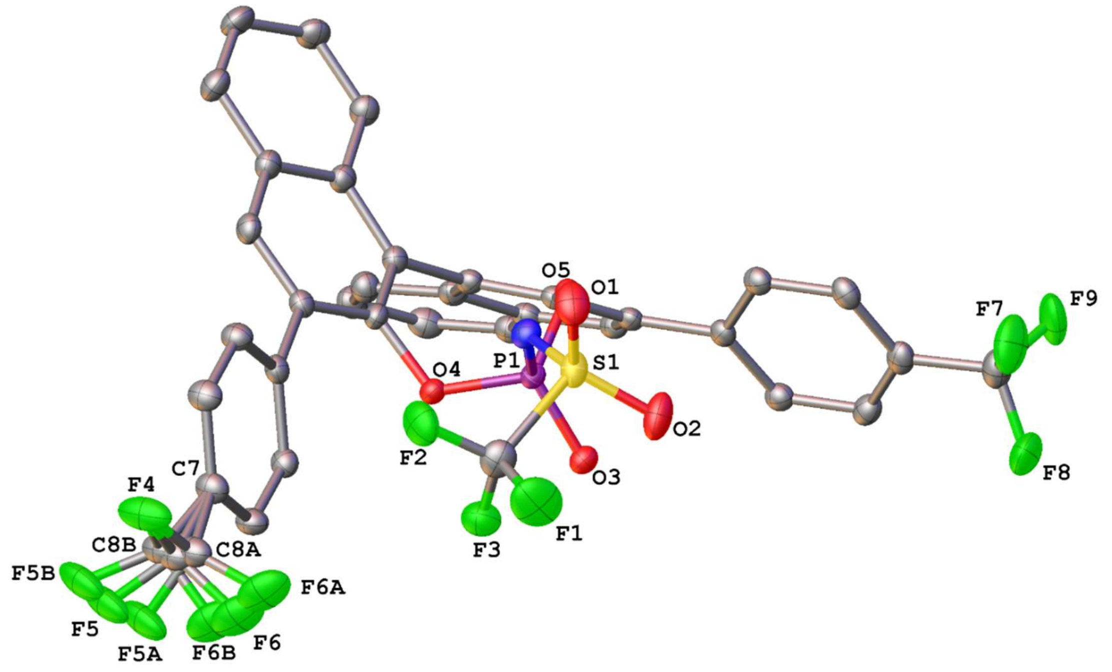

Supplementary Figure 12. A molecular drawing of yoon62a shown with 50% probability ellipsoids. The two diethyl ether solvent molecules are shown with their disorder components. All H atoms are omitted.

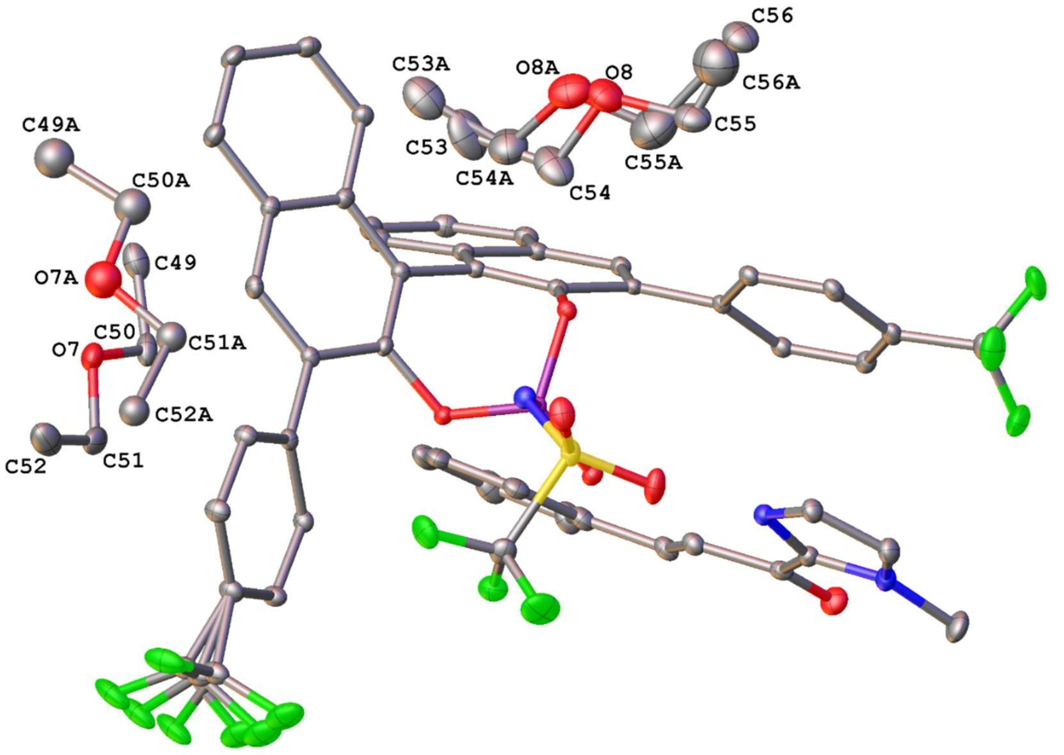

Supplementary Table 8. Crystal data and structure refinement for yoon62a

Identification code	yoon62a
Empirical formula	$[C_{13}H_{13}N_2O][C_{35}H_{18}F_9NO_5PS]\cdot 2Et_2O$
Formula weight	1128.03
Temperature/K	100.0
Crystal system	orthorhombic
Space group	$P2_12_12_1$
a/Å	13.6603(16)
b/Å	16.608(2)
c/Å	23.617(3)
α/°	90
β/°	90
γ/°	90
Volume/Å^3	5358.1(12)
Z	4
ρ_{calc}g/cm^3	1.398
μ/mm^{-1}	1.594
F(000)	2336.0
Crystal size/mm^3	0.38 × 0.32 × 0.25
Radiation	CuKα (λ = 1.54178)
2Θ range for data collection/°	6.506 to 158.416
Index ranges	$-17 \le h \le 17$, $-21 \le k \le 20$, $-28 \le l \le 30$
Reflections collected	203898
Independent reflections	11554 [R_{int} = 0.0437, R_{sigma} = 0.0215]
Data/restraints/parameters	11554/118/804
Goodness-of-fit on F^2	1.095
Final R indexes [I>=2σ (I)]	R_1 = 0.0266, wR_2 = 0.0684
Final R indexes [all data]	R_1 = 0.0268, wR_2 = 0.0685
Largest diff. peak/hole / e Å^{-3}	0.21/-0.28
Flack parameter	0.023(11)

Supplementary Table 9. Fractional Atomic Coordinates (×10^4) and Equivalent Isotropic Displacement Parameters (Å^2×10^3) for yoon62a. U_{eq} is defined as 1/3 of of the trace of the orthogonalised U_{IJ} tensor.

**Atom**	***x***	***y***	***z***	**U(eq)**
S1	5114.7(4)	2929.8(3)	2722.4(2)	22.34(10)
P1	4381.0(3)	3306.6(3)	3799.8(2)	15.68(9)
F1	6981.0(11)	2686.6(11)	2525.5(7)	52.3(4)
F2	6593.8(10)	3929.3(8)	2660.3(6)	38.2(3)
F3	6669.1(9)	3114.4(8)	3365.7(5)	33.4(3)
C8B	8874(12)	5536(8)	3193(3)	27.3(9)
F4	9069.0(9)	5768.5(10)	2662.4(6)	41.8(4)
F5B	9343(18)	6056(11)	3527(5)	43.8(17)
F6B	9325(17)	4833(9)	3273(7)	47.9(17)
C8	8904(4)	5392(3)	3154.6(15)	27.3(9)
F5	9435(3)	5770(3)	3547.3(13)	43.8(17)
F6	9275(3)	4651(2)	3115(2)	47.9(17)

C8A	8839(8)	5261(5)	3082(3)	27.3(9)
F5A	9471(8)	5406(5)	3500(3)	43.8(17)
F6A	9075(8)	4526(4)	2898(3)	47.9(17)
C33	1886.2(15)	-369.6(12)	3744.8(9)	29.5(4)
F7	2064.6(13)	-418.9(9)	3192.6(6)	48.9(4)
F8	2448.0(12)	-925.7(8)	3994.4(7)	47.0(4)
F9	962.9(10)	-612.9(8)	3821.1(7)	45.8(4)
O1	4936.4(13)	3168.4(10)	2149.3(6)	32.6(3)
O2	5076.6(13)	2079.6(9)	2842.2(6)	33.9(4)
O3	4789.3(9)	2582.2(8)	4075.4(5)	19.2(3)
O4	4751.6(9)	4068.6(7)	4156.7(5)	17.3(2)
O5	3214.5(9)	3364.1(7)	3852.9(5)	17.0(2)
N1	4570.5(12)	3485.2(9)	3143.5(7)	20.9(3)
C1	6418.9(16)	3182.6(13)	2826.1(9)	28.9(4)
C2	4284.0(13)	4813.9(10)	4079.8(7)	17.0(3)
C3	4808.0(13)	5439.8(10)	3799.3(7)	17.3(3)
C4	5847.2(13)	5362.1(11)	3615.6(7)	17.8(3)
C5	6548.9(14)	4946.6(11)	3934.3(8)	19.4(4)
C6	7525.4(14)	4959.4(12)	3778.9(8)	22.4(4)
C7	7819.1(14)	5378.9(12)	3297.4(8)	24.1(4)
C9	7131.9(15)	5785.5(13)	2973.7(8)	24.3(4)
C10	6155.2(14)	5778.0(12)	3132.0(8)	21.4(4)
C11	4322.8(14)	6160.7(11)	3722.9(8)	20.0(3)
C12	3345.1(14)	6274.0(11)	3892.3(8)	20.1(4)
C13	2846.8(15)	7009.5(11)	3776.3(9)	24.8(4)
C14	1882.1(15)	7097.2(12)	3917.2(9)	27.3(4)
C15	1370.8(15)	6461.5(12)	4180.4(9)	25.2(4)
C16	1834.3(14)	5753.3(12)	4307.4(8)	22.1(4)
C17	2837.4(14)	5641.4(11)	4173.9(8)	19.1(3)
C18	3352.0(13)	4907.3(11)	4290.4(8)	17.7(3)
C19	2888.0(13)	4247.3(11)	4622.0(8)	17.7(3)
C20	2559.4(13)	4374.2(11)	5192.9(8)	18.6(3)
C21	2685.0(14)	5115.8(12)	5484.4(8)	22.9(4)
C22	2398.6(15)	5197.4(12)	6036.4(9)	25.0(4)
C23	1960.5(14)	4547.9(13)	6328.0(8)	24.9(4)
C24	1831.5(14)	3825.2(12)	6061.0(8)	21.9(4)
C25	2134.9(13)	3716.5(11)	5490.8(8)	19.2(4)
C26	2041.3(13)	2960.1(11)	5221.8(8)	18.6(3)
C27	2390.7(12)	2818.9(11)	4683.2(8)	16.9(3)
C28	2826.3(13)	3482.5(11)	4396.7(7)	16.7(3)
C29	2299.2(12)	2005.8(11)	4426.9(8)	17.4(3)
C30	2385.0(13)	1321.2(11)	4769.5(8)	20.4(4)
C31	2268.2(14)	555.1(12)	4550.8(9)	22.5(4)
C32	2056.3(13)	458.4(12)	3977.7(8)	22.3(4)
C34	1954.4(13)	1129.0(12)	3632.3(8)	21.5(4)

C35	2078.1(13)	1896.6(11)	3851.7(8)	19.6(3)
O6	4700.5(12)	-5.6(9)	5287.7(6)	31.3(3)
N2	4660.8(12)	-307.6(10)	4099.1(7)	24.2(3)
N3	4543.0(12)	972.7(10)	3938.2(7)	19.5(3)
C36	4778(2)	-1102.4(13)	4368.2(11)	36.8(5)
C37	4548.9(15)	-209.2(12)	3527.5(9)	26.5(4)
C38	4473.1(15)	595.3(12)	3429.2(8)	23.1(4)
C39	4656.6(13)	422.4(11)	4348.9(8)	20.5(4)
C40	4706.9(13)	572.9(12)	4967.2(8)	22.0(4)
C41	4729.5(14)	1412.4(12)	5152.3(8)	22.5(4)
C42	4701.4(14)	1599.0(13)	5703.7(8)	25.1(4)
C43	4650.8(14)	2409.5(13)	5941.9(9)	26.4(4)
C44	4499.4(18)	2501.1(16)	6523.0(10)	35.5(5)
C45	4409.8(19)	3259.9(17)	6762.5(10)	40.4(5)
C46	4482.4(16)	3940.0(15)	6427.2(11)	35.8(5)
C47	4642.8(15)	3859.4(14)	5849.8(10)	30.4(4)
C48	4723.3(14)	3105.4(13)	5604.9(9)	26.7(4)
O7	5399.6(14)	7273.6(10)	4732.1(8)	32.2(5)
C49	3991(3)	7173(2)	5320.1(15)	44.3(8)
C50	4945(3)	6799(2)	5157.1(15)	35.6(8)
C51	6336(2)	6981.9(17)	4584.8(14)	39.6(7)
C52	6702(2)	7442(2)	4081.6(16)	51.3(9)
O7A	4344(9)	7401(8)	5264(7)	59(4)
C49A	3106(14)	8205(10)	5675(10)	67(6)
C50A	3368(10)	7379(9)	5473(9)	56(5)
C51A	4715(11)	6629(8)	5136(11)	40(6)
C52A	5740(9)	6720(9)	4914(6)	39(4)
O8	1287(4)	3835(4)	2319(3)	51.6(13)
C53	2121(7)	4877(7)	2795(3)	74(3)
C54	2165(5)	4035(6)	2590(3)	64(2)
C55	1206(4)	3018(4)	2179(2)	48.6(14)
C56	243(5)	2885(5)	1893(3)	49.2(14)
O8A	1378(9)	4157(8)	2359(5)	59(3)
C53A	1924(9)	5374(10)	2762(5)	67(4)
C54A	2103(9)	4487(10)	2722(6)	64(4)
C55A	1414(13)	3309(8)	2334(9)	95(6)
C56A	595(15)	3008(10)	1967(8)	92(6)

Supplementary Table 10. Anisotropic Displacement Parameters (Å^2×10^3) for yoon62a. The Anisotropic displacement factor exponent takes the form: $-2\pi^2[h^2a^{*2}U_{11}+2hka^*b^*U_{12}+\ldots]$.

**Atom**	**U_{11}**	**U_{22}**	**U_{33}**	**U_{23}**	**U_{13}**	**U_{12}**
S1	29.3(2)	19.7(2)	18.0(2)	-1.15(16)	4.85(17)	-5.17(18)
P1	16.09(19)	14.61(19)	16.34(19)	1.21(16)	1.69(16)	-0.35(16)
F1	39.9(8)	62.8(10)	54.4(9)	-19.0(8)	16.1(7)	13.0(7)
F2	36.2(7)	39.9(7)	38.4(7)	7.3(6)	5.2(6)	-16.0(6)
F3	28.9(6)	38.0(7)	33.4(7)	2.1(5)	-0.8(5)	1.8(5)

C8B	20.2(12)	35(2)	26.7(14)	10.6(15)	0.6(12)	-2.7(15)
F4	22.0(6)	69.2(10)	34.2(7)	25.4(7)	4.5(5)	-1.8(6)
F5B	25.3(14)	67(5)	38.9(10)	17(2)	-9.4(8)	-22(4)
F6B	36(2)	50(2)	58(4)	14(3)	22(3)	16.9(19)
C8	20.2(12)	35(2)	26.7(14)	10.6(15)	0.6(12)	-2.7(15)
F5	25.3(14)	67(5)	38.9(10)	17(2)	-9.4(8)	-22(4)
F6	36(2)	50(2)	58(4)	14(3)	22(3)	16.9(19)
C8A	20.2(12)	35(2)	26.7(14)	10.6(15)	0.6(12)	-2.7(15)
F5A	25.3(14)	67(5)	38.9(10)	17(2)	-9.4(8)	-22(4)
F6A	36(2)	50(2)	58(4)	14(3)	22(3)	16.9(19)
C33	28.4(10)	23.7(10)	36.3(11)	-2.8(9)	6.2(9)	-5.0(8)
F7	76.3(11)	31.6(7)	38.8(8)	-13.9(6)	15.6(7)	-11.4(7)
F8	51.2(9)	20.2(6)	69.6(10)	-5.0(6)	-8.3(8)	2.7(6)
F9	33.5(7)	31.7(7)	72.1(10)	-13.2(7)	9.4(7)	-16.6(6)
O1	44.4(9)	34.2(8)	19.3(7)	-0.6(6)	3.3(6)	-9.3(7)
O2	52.2(10)	20.0(7)	29.4(7)	-3.3(6)	11.0(7)	-7.1(7)
O3	20.9(6)	17.1(6)	19.5(6)	1.2(5)	1.0(5)	1.5(5)
O4	16.0(6)	15.1(6)	20.9(6)	1.7(5)	-0.2(5)	0.5(5)
O5	16.5(6)	17.7(6)	16.8(6)	0.9(5)	0.9(5)	-2.0(5)
N1	22.3(8)	20.3(7)	20.2(7)	4.2(6)	1.5(6)	-0.7(6)
C1	27.4(10)	30.9(10)	28.3(10)	-1.0(8)	9.7(8)	1.4(8)
C2	18.9(8)	13.3(8)	18.7(8)	0.8(6)	-1.5(7)	-0.4(6)
C3	17.4(8)	18.2(8)	16.4(7)	-0.6(7)	-0.2(7)	-2.8(6)
C4	18.5(8)	17.4(8)	17.3(8)	-1.6(6)	-0.1(6)	-3.3(7)
C5	20.5(8)	19.5(8)	18.2(8)	2.7(7)	0.5(7)	-2.8(7)
C6	19.2(8)	26.3(9)	21.7(9)	5.1(8)	-2.0(7)	0.0(7)
C7	19.6(9)	28.8(10)	24.0(9)	4.7(8)	1.8(7)	-2.9(8)
C9	22.2(9)	29.1(10)	21.6(9)	6.9(8)	2.1(7)	-2.2(8)
C10	19.9(9)	24.3(9)	19.9(9)	4.0(7)	-0.5(7)	0.0(7)
C11	22.2(8)	16.4(8)	21.3(8)	2.3(7)	0.9(7)	-4.3(7)
C12	22.7(9)	17.1(8)	20.7(9)	-0.8(7)	0.2(7)	-0.6(7)
C13	27.9(9)	17.2(8)	29.2(10)	2.3(8)	2.5(8)	1.8(7)
C14	29.4(10)	19.5(9)	33.0(11)	2.2(8)	0.1(8)	7.6(8)
C15	20.3(9)	25.6(10)	29.7(10)	-0.9(8)	2.5(8)	5.2(7)
C16	21.0(9)	21.2(9)	24.2(9)	-0.6(7)	2.8(7)	0.4(7)
C17	19.7(8)	17.3(8)	20.2(8)	-1.4(7)	0.2(7)	0.3(7)
C18	18.4(8)	16.9(8)	17.9(8)	-0.6(6)	0.0(7)	-1.2(7)
C19	13.3(7)	18.8(8)	20.9(9)	2.5(7)	0.3(7)	0.3(6)
C20	14.0(8)	20.9(9)	20.8(9)	0.5(7)	0.5(6)	1.5(7)
C21	22.1(9)	21.8(9)	24.8(9)	-0.5(7)	0.8(7)	-1.2(7)
C22	24.1(9)	24.6(10)	26.2(10)	-4.6(8)	-0.2(7)	0.2(8)
C23	22.8(9)	33.3(10)	18.7(9)	-0.7(8)	0.9(7)	2.2(8)
C24	17.4(8)	27.3(9)	20.9(9)	3.3(7)	0.9(7)	-0.1(7)
C25	14.2(8)	22.9(9)	20.5(9)	1.3(7)	-0.9(7)	0.7(7)
C26	15.8(8)	20.0(8)	19.9(8)	4.2(7)	-0.6(6)	-1.1(7)

C27	11.5(7)	18.2(8)	21.2(8)	1.5(7)	-1.6(6)	-0.2(6)
C28	13.5(7)	21.0(8)	15.7(8)	0.8(6)	-0.7(6)	-0.1(6)
C29	12.4(7)	18.4(8)	21.4(8)	0.9(7)	1.4(6)	-2.3(6)
C30	17.1(8)	21.9(9)	22.4(9)	2.6(7)	0.2(7)	-1.6(7)
C31	21.1(9)	18.9(9)	27.4(10)	4.1(7)	3.9(7)	-0.8(7)
C32	17.0(8)	21.0(9)	29.0(10)	-0.9(7)	5.4(7)	-3.3(7)
C34	17.0(8)	25.8(9)	21.8(9)	-1.8(7)	2.5(7)	-5.2(7)
C35	15.5(8)	21.3(9)	21.9(9)	3.1(7)	0.8(7)	-2.5(6)
O6	37.9(8)	29.1(7)	27.0(7)	9.4(6)	-1.5(6)	0.6(6)
N2	25.7(8)	18.9(8)	28.1(8)	1.6(6)	-1.5(7)	-2.1(6)
N3	19.9(7)	17.6(7)	21.0(8)	1.6(6)	-1.5(6)	-0.8(6)
C36	49.2(14)	18.6(9)	42.7(12)	7.0(9)	-4.8(11)	-0.3(9)
C37	29.7(10)	23.2(9)	26.5(9)	-3.5(8)	-1.5(8)	-3.8(8)
C38	24.5(9)	23.3(9)	21.5(9)	-0.6(7)	-2.9(7)	-3.3(7)
C39	17.9(8)	18.8(8)	24.9(9)	1.8(7)	0.1(7)	-0.3(7)
C40	15.5(8)	27.2(9)	23.4(9)	4.8(8)	-1.1(7)	-0.5(7)
C41	20.4(9)	25.1(9)	22.0(9)	4.0(7)	-1.4(7)	-3.8(7)
C42	20.8(9)	31.5(10)	22.9(9)	3.8(8)	-2.6(7)	-6.3(8)
C43	20.5(9)	34.0(11)	24.8(10)	-1.2(8)	-3.5(8)	-6.0(8)
C44	38.9(12)	41.4(12)	26.2(10)	-2.5(9)	-2.1(9)	-10.9(10)
C45	38.7(12)	53.6(14)	29.0(11)	-10.6(11)	1.2(10)	-10.2(12)
C46	23.6(10)	40.6(12)	43.3(13)	-15.9(10)	-4.7(9)	-2.0(9)
C47	19.5(9)	33.2(11)	38.6(12)	-2.4(9)	-8.4(8)	0.4(8)
C48	18.4(9)	35.0(11)	26.6(9)	-1.2(8)	-5.7(7)	-1.4(8)
O7	36.3(10)	20.6(9)	39.8(10)	1.2(7)	-4.1(8)	-1.3(7)
C49	51(2)	43.3(17)	38.3(16)	-3.1(14)	-0.8(14)	-17.9(17)
C50	50.0(19)	23.4(13)	33.5(16)	1.9(12)	-12.0(14)	-10.5(15)
C51	40.5(16)	25.0(13)	53.3(18)	-14.7(12)	-11.4(13)	4.4(11)
C52	37.9(16)	50.0(19)	66(2)	-21.5(17)	8.4(15)	-3.7(14)
O8	49(2)	63(3)	42.9(19)	1(2)	-12.7(15)	-3(2)
C53	88(5)	94(7)	40(3)	-24(4)	-1(3)	-31(5)
C54	66(3)	81(5)	45(3)	19(3)	-24(2)	-17(3)
C55	42(3)	59(3)	45(3)	11(2)	-11(2)	4(2)
C56	46(3)	60(3)	42(2)	6(2)	-14(2)	-1(3)
O8A	63(5)	70(6)	44(4)	17(4)	1(3)	13(4)
C53A	69(6)	92(9)	39(4)	0(6)	-5(4)	-16(6)
C54A	66(6)	64(6)	62(6)	10(5)	-16(5)	5(5)
C55A	103(8)	82(7)	100(8)	17(6)	-6(6)	15(6)
C56A	90(12)	92(10)	94(11)	11(8)	6(9)	-7(9)

Supplementary Table 11. Bond Lengths for yoon62a

Atom	Atom	Length/Å	Atom	Atom	Length/Å
S1	O1	1.4311(15)	C20	C21	1.421(3)
S1	O2	1.4410(15)	C20	C25	1.423(3)
S1	N1	1.5468(17)	C21	C22	1.368(3)
S1	C1	1.847(2)	C22	C23	1.413(3)
P1	O3	1.4772(13)	C23	C24	1.367(3)
P1	O4	1.6026(13)	C24	C25	1.420(3)
P1	O5	1.6013(13)	C25	C26	1.414(3)
P1	N1	1.5992(16)	C26	C27	1.379(3)
F1	C1	1.331(2)	C27	C28	1.424(2)
F2	C1	1.322(3)	C27	C29	1.485(3)
F3	C1	1.324(3)	C29	C30	1.400(3)
C8B	F4	1.337(6)	C29	C35	1.403(3)
C8B	F5B	1.335(6)	C30	C31	1.383(3)
C8B	F6B	1.333(6)	C31	C32	1.394(3)
C8B	C7	1.486(16)	C32	C34	1.387(3)
F4	C8	1.339(4)	C34	C35	1.387(3)
F4	C8A	1.338(5)	O6	C40	1.223(2)
C8	F5	1.334(4)	N2	C36	1.474(3)
C8	F6	1.334(4)	N2	C37	1.368(3)
C8	C7	1.520(6)	N2	C39	1.348(3)
C8A	F5A	1.334(6)	N3	C38	1.359(2)
C8A	F6A	1.334(6)	N3	C39	1.342(2)
C8A	C7	1.497(11)	C37	C38	1.360(3)
C33	F7	1.329(3)	C39	C40	1.483(3)
C33	F8	1.338(3)	C40	C41	1.461(3)
C33	F9	1.337(2)	C41	C42	1.339(3)
C33	C32	1.499(3)	C42	C43	1.460(3)
O4	C2	1.405(2)	C43	C44	1.396(3)
O5	C28	1.403(2)	C43	C48	1.407(3)
C2	C3	1.425(2)	C44	C45	1.387(4)
C2	C18	1.376(3)	C45	C46	1.383(4)
C3	C4	1.490(2)	C46	C47	1.388(3)
C3	C11	1.380(3)	C47	C48	1.384(3)
C4	C5	1.401(3)	O7	C50	1.419(4)
C4	C10	1.400(3)	O7	C51	1.412(4)
C5	C6	1.384(3)	C49	C50	1.493(5)
C6	C7	1.393(3)	C51	C52	1.499(5)
C7	C9	1.386(3)	O7A	C50A	1.422(8)
C9	C10	1.386(3)	O7A	C51A	1.411(7)
C11	C12	1.407(3)	C49A	C50A	1.496(8)
C12	C13	1.425(3)	C51A	C52A	1.501(8)
C12	C17	1.424(3)	O8	C54	1.399(8)
C13	C14	1.367(3)	O8	C55	1.401(8)

C14	C15	1.410(3)	C53	C54	1.481(10)
C15	C16	1.369(3)	C55	C56	1.495(7)
C16	C17	1.418(3)	O8A	C54A	1.420(8)
C17	C18	1.434(3)	O8A	C55A	1.412(7)
C18	C19	1.489(2)	C53A	C54A	1.495(8)
C19	C20	1.437(3)	C55A	C56A	1.501(8)
C19	C28	1.380(3)			

Supplementary Table 12. Bond Angles for yoon62a

Atom	Atom	Atom	Angle/°	Atom	Atom	Atom	Angle/°
O1	S1	O2	116.81(9)	C15	C16	C17	120.70(18)
O1	S1	N1	111.17(9)	C12	C17	C18	118.57(17)
O1	S1	C1	103.10(10)	C16	C17	C12	118.55(17)
O2	S1	N1	116.15(9)	C16	C17	C18	122.84(17)
O2	S1	C1	103.39(10)	C2	C18	C17	118.69(16)
N1	S1	C1	104.05(9)	C2	C18	C19	120.07(16)
O3	P1	O4	106.99(7)	C17	C18	C19	121.22(16)
O3	P1	O5	112.94(7)	C20	C19	C18	121.24(16)
O3	P1	N1	121.13(8)	C28	C19	C18	120.07(16)
O5	P1	O4	103.07(7)	C28	C19	C20	118.55(16)
N1	P1	O4	108.19(8)	C21	C20	C19	122.96(17)
N1	P1	O5	103.05(8)	C21	C20	C25	118.35(17)
F4	C8B	C7	113.5(10)	C25	C20	C19	118.61(17)
F5B	C8B	F4	105.8(8)	C22	C21	C20	120.87(18)
F5B	C8B	C7	118.7(13)	C21	C22	C23	120.66(19)
F6B	C8B	F4	107.1(9)	C24	C23	C22	120.00(18)
F6B	C8B	F5B	105.2(8)	C23	C24	C25	120.75(18)
F6B	C8B	C7	105.7(12)	C24	C25	C20	119.35(17)
F4	C8	C7	111.3(3)	C26	C25	C20	119.79(16)
F5	C8	F4	107.0(3)	C26	C25	C24	120.84(17)
F5	C8	F6	106.0(4)	C27	C26	C25	122.32(17)
F5	C8	C7	112.5(3)	C26	C27	C28	116.84(16)
F6	C8	F4	107.8(4)	C26	C27	C29	120.11(16)
F6	C8	C7	111.9(3)	C28	C27	C29	123.04(16)
F4	C8A	C7	112.8(6)	O5	C28	C27	118.98(16)
F5A	C8A	F4	106.5(6)	C19	C28	O5	117.31(15)
F5A	C8A	F6A	104.5(6)	C19	C28	C27	123.71(17)
F5A	C8A	C7	109.1(7)	C30	C29	C27	119.71(16)
F6A	C8A	F4	106.2(6)	C30	C29	C35	118.19(17)
F6A	C8A	C7	117.1(7)	C35	C29	C27	122.02(16)
F7	C33	F8	106.53(18)	C31	C30	C29	121.45(18)
F7	C33	F9	106.65(18)	C30	C31	C32	119.52(18)
F7	C33	C32	112.83(17)	C31	C32	C33	119.61(18)
F8	C33	C32	112.51(18)	C34	C32	C33	120.34(18)
F9	C33	F8	105.86(17)	C34	C32	C31	119.96(18)

F9	C33	C32	111.98(17)	C35	C34	C32	120.41(18)
C2	O4	P1	118.96(11)	C34	C35	C29	120.45(17)
C28	O5	P1	117.13(11)	C37	N2	C36	123.00(18)
S1	N1	P1	126.18(10)	C39	N2	C36	128.11(17)
F1	C1	S1	110.18(16)	C39	N2	C37	108.89(17)
F2	C1	S1	110.38(15)	C39	N3	C38	109.47(16)
F2	C1	F1	108.56(18)	C38	C37	N2	107.12(18)
F2	C1	F3	108.58(18)	N3	C38	C37	107.27(17)
F3	C1	S1	110.93(14)	N2	C39	C40	125.60(17)
F3	C1	F1	108.14(18)	N3	C39	N2	107.26(16)
O4	C2	C3	118.32(15)	N3	C39	C40	127.04(17)
C18	C2	O4	118.26(15)	O6	C40	C39	118.45(18)
C18	C2	C3	123.40(16)	O6	C40	C41	124.38(18)
C2	C3	C4	123.41(16)	C41	C40	C39	117.14(16)
C11	C3	C2	116.89(16)	C42	C41	C40	120.72(18)
C11	C3	C4	119.64(16)	C41	C42	C43	126.10(19)
C5	C4	C3	122.56(16)	C44	C43	C42	119.1(2)
C10	C4	C3	118.77(16)	C44	C43	C48	118.5(2)
C10	C4	C5	118.43(17)	C48	C43	C42	122.41(18)
C6	C5	C4	120.64(17)	C45	C44	C43	120.9(2)
C5	C6	C7	120.14(17)	C46	C45	C44	120.2(2)
C6	C7	C8B	120.2(4)	C45	C46	C47	119.7(2)
C6	C7	C8	118.0(2)	C48	C47	C46	120.7(2)
C6	C7	C8A	118.7(3)	C47	C48	C43	120.1(2)
C9	C7	C8B	118.7(4)	C51	O7	C50	112.4(2)
C9	C7	C8	122.1(2)	O7	C50	C49	109.5(3)
C9	C7	C8A	120.5(3)	O7	C51	C52	108.8(2)
C9	C7	C6	119.92(18)	C51A	O7A	C50A	112.9(9)
C10	C9	C7	119.93(18)	O7A	C50A	C49A	108.2(9)
C9	C10	C4	120.93(18)	O7A	C51A	C52A	108.6(9)
C3	C11	C12	122.32(17)	C54	O8	C55	113.9(5)
C11	C12	C13	120.90(17)	O8	C54	C53	109.8(6)
C11	C12	C17	119.77(17)	O8	C55	C56	108.6(5)
C17	C12	C13	119.32(17)	C55A	O8A	C54A	112.7(8)
C14	C13	C12	120.34(18)	O8A	C54A	C53A	107.7(7)
C13	C14	C15	120.33(18)	O8A	C55A	C56A	109.3(9)
C16	C15	C14	120.71(19)				

Supplementary Table 13. Hydrogen Bonds for yoon62a

D	H	A	d(D-H)/Å	d(H-A)/Å	d(D-A)/Å	D-H-A/°
N3	H3	O3	0.86(3)	1.87(3)	2.714(2)	170(2)
C41	H41	O3	0.95	2.27	3.202(2)	166.2
C48	H48	O3	0.95	2.79	3.716(2)	164.9

Supplementary Table 14. Torsion Angles for yoon62a

A	B	C	D	Angle/°	A	B	C	D	Angle/°
P1	O4	C2	C3	-109.76(16)	C13	C12	C17	C18	-179.60(18)
P1	O4	C2	C18	71.67(19)	C13	C14	C15	C16	1.1(3)
P1	O5	C28	C19	76.47(18)	C14	C15	C16	C17	-0.4(3)
P1	O5	C28	C27	-103.82(16)	C15	C16	C17	C12	-1.5(3)
C8B	C7	C9	C10	-167.2(6)	C15	C16	C17	C18	-179.05(18)
F4	C8B	C7	C6	165.1(6)	C16	C17	C18	C2	171.51(18)
F4	C8B	C7	C9	-27.4(12)	C16	C17	C18	C19	-7.2(3)
F4	C8	C7	C6	175.7(2)	C17	C12	C13	C14	-2.1(3)
F4	C8	C7	C9	-6.0(4)	C17	C18	C19	C20	-59.9(2)
F4	C8A	C7	C6	-172.2(4)	C17	C18	C19	C28	124.48(19)
F4	C8A	C7	C9	18.7(8)	C18	C2	C3	C4	174.51(17)
F5B	C8B	C7	C6	-69.7(10)	C18	C2	C3	C11	-2.5(3)
F5B	C8B	C7	C9	97.9(9)	C18	C19	C20	C21	-2.4(3)
F6B	C8B	C7	C6	47.9(9)	C18	C19	C20	C25	-179.08(16)
F6B	C8B	C7	C9	-144.5(8)	C18	C19	C28	O5	0.0(2)
C8	C7	C9	C10	-177.8(3)	C18	C19	C28	C27	-179.67(16)
F5	C8	C7	C6	-64.2(4)	C19	C20	C21	C22	-177.10(18)
F5	C8	C7	C9	114.1(3)	C19	C20	C25	C24	178.22(16)
F6	C8	C7	C6	55.1(4)	C19	C20	C25	C26	-0.3(3)
F6	C8	C7	C9	-126.7(3)	C20	C19	C28	O5	-175.70(15)
C8A	C7	C9	C10	169.4(4)	C20	C19	C28	C27	4.6(3)
F5A	C8A	C7	C6	-54.2(6)	C20	C21	C22	C23	-0.7(3)
F5A	C8A	C7	C9	136.7(4)	C20	C25	C26	C27	3.2(3)
F6A	C8A	C7	C6	64.1(7)	C21	C20	C25	C24	1.4(3)
F6A	C8A	C7	C9	-105.0(5)	C21	C20	C25	C26	-177.08(17)
C33	C32	C34	C35	177.75(17)	C21	C22	C23	C24	0.9(3)
F7	C33	C32	C31	-155.72(19)	C22	C23	C24	C25	0.1(3)
F7	C33	C32	C34	27.6(3)	C23	C24	C25	C20	-1.3(3)
F8	C33	C32	C31	-35.1(3)	C23	C24	C25	C26	177.23(17)
F8	C33	C32	C34	148.18(18)	C24	C25	C26	C27	-175.33(17)
F9	C33	C32	C31	83.9(2)	C25	C20	C21	C22	-0.5(3)
F9	C33	C32	C34	-92.7(2)	C25	C26	C27	C28	-2.1(3)
O1	S1	N1	P1	165.06(12)	C25	C26	C27	C29	178.19(16)
O1	S1	C1	F1	-69.98(17)	C26	C27	C28	O5	178.46(15)
O1	S1	C1	F2	49.90(16)	C26	C27	C28	C19	-1.9(3)
O1	S1	C1	F3	170.31(15)	C26	C27	C29	C30	-35.1(2)
O2	S1	N1	P1	28.27(17)	C26	C27	C29	C35	141.56(18)
O2	S1	C1	F1	52.12(17)	C27	C29	C30	C31	177.56(17)
O2	S1	C1	F2	172.00(14)	C27	C29	C35	C34	-177.06(16)
O2	S1	C1	F3	-67.60(16)	C28	C19	C20	C21	173.22(17)
O3	P1	O4	C2	-164.13(12)	C28	C19	C20	C25	-3.4(3)
O3	P1	O5	C28	66.70(14)	C28	C27	C29	C30	145.22(18)
O3	P1	N1	S1	4.19(17)	C28	C27	C29	C35	-38.1(3)

O4	P1	O5	C28	-48.39(13)	C29	C27	C28	O5	-1.9(2)
O4	P1	N1	S1	128.09(12)	C29	C27	C28	C19	177.82(17)
O4	C2	C3	C4	-4.0(3)	C29	C30	C31	C32	-0.3(3)
O4	C2	C3	C11	179.06(15)	C30	C29	C35	C34	-0.3(3)
O4	C2	C18	C17	-174.91(15)	C30	C31	C32	C33	-177.35(17)
O4	C2	C18	C19	3.8(2)	C30	C31	C32	C34	-0.7(3)
O5	P1	O4	C2	-44.83(14)	C31	C32	C34	C35	1.1(3)
O5	P1	N1	S1	-123.22(12)	C32	C34	C35	C29	-0.6(3)
N1	S1	C1	F1	173.89(15)	C35	C29	C30	C31	0.8(3)
N1	S1	C1	F2	-66.23(16)	O6	C40	C41	C42	-3.0(3)
N1	S1	C1	F3	54.17(17)	N2	C37	C38	N3	0.2(2)
N1	P1	O4	C2	63.85(14)	N2	C39	C40	O6	-4.1(3)
N1	P1	O5	C28	-160.90(12)	N2	C39	C40	C41	177.80(17)
C1	S1	N1	P1	-84.61(14)	N3	C39	C40	O6	171.78(19)
C2	C3	C4	C5	-37.0(3)	N3	C39	C40	C41	-6.3(3)
C2	C3	C4	C10	148.69(18)	C36	N2	C37	C38	-179.30(19)
C2	C3	C11	C12	-2.3(3)	C36	N2	C39	N3	179.1(2)
C2	C18	C19	C20	121.38(19)	C36	N2	C39	C40	-4.3(3)
C2	C18	C19	C28	-54.2(2)	C37	N2	C39	N3	0.0(2)
C3	C2	C18	C17	6.6(3)	C37	N2	C39	C40	176.58(18)
C3	C2	C18	C19	-174.66(16)	C38	N3	C39	N2	0.1(2)
C3	C4	C5	C6	-173.24(18)	C38	N3	C39	C40	-176.37(18)
C3	C4	C10	C9	173.99(18)	C39	N2	C37	C38	-0.1(2)
C3	C11	C12	C13	-176.15(18)	C39	N3	C38	C37	-0.2(2)
C3	C11	C12	C17	2.6(3)	C39	C40	C41	C42	175.00(18)
C4	C3	C11	C12	-179.38(16)	C40	C41	C42	C43	-175.45(18)
C4	C5	C6	C7	-0.9(3)	C41	C42	C43	C44	171.8(2)
C5	C4	C10	C9	-0.5(3)	C41	C42	C43	C48	-6.5(3)
C5	C6	C7	C8B	167.5(7)	C42	C43	C44	C45	-177.5(2)
C5	C6	C7	C8	178.4(2)	C42	C43	C48	C47	178.15(19)
C5	C6	C7	C8A	-169.1(4)	C43	C44	C45	C46	-0.8(4)
C5	C6	C7	C9	0.1(3)	C44	C43	C48	C47	-0.1(3)
C6	C7	C9	C10	0.4(3)	C44	C45	C46	C47	0.2(4)
C7	C9	C10	C4	-0.2(3)	C45	C46	C47	C48	0.5(3)
C10	C4	C5	C6	1.1(3)	C46	C47	C48	C43	-0.5(3)
C11	C3	C4	C5	139.87(19)	C48	C43	C44	C45	0.8(3)
C11	C3	C4	C10	-34.4(3)	C50	O7	C51	C52	172.1(3)
C11	C12	C13	C14	176.64(19)	C51	O7	C50	C49	176.1(2)
C11	C12	C17	C16	-176.02(17)	C50A	O7A	C51A	C52A	179.4(17)
C11	C12	C17	C18	1.6(3)	C51A	O7A	C50A	C49A	171.6(19)
C12	C13	C14	C15	0.2(3)	C54	O8	C55	C56	-179.9(6)
C12	C17	C18	C2	-6.0(3)	C55	O8	C54	C53	-171.7(6)
C12	C17	C18	C19	175.28(17)	C54A	O8A	C55A	C56A	177.1(14)
C13	C12	C17	C16	2.8(3)	C55A	O8A	C54A	C53A	-174.1(14)

Supplementary Table 15. Hydrogen Atom Coordinates (Å×10^4) and Isotropic Displacement Parameters (Å^2×10^3) for yoon62a

Atom	*x*	*y*	*z*	U(eq)
H5	6352.46	4653.03	4260.3	23
H6	7996.41	4681.51	4001.12	27
H9	7330.36	6068.96	2643.73	29
H10	5688.05	6059.21	2909.07	26
H11	4661.08	6595.71	3549.8	24
H13	3186.85	7441.11	3600.22	30
H14	1554.43	7588.8	3837.35	33
H15	697.51	6525.38	4270.51	30
H16	1480.26	5332.3	4486.94	27
H21	2971.48	5560.56	5293.36	27
H22	2495.37	5695.85	6225.66	30
H23	1755.53	4613.29	6709.62	30
H24	1535.6	3390.84	6259	26
H26	1726.44	2534.1	5419.01	22
H30	2526.57	1384.75	5160.74	25
H31	2331.81	97.74	4789.64	27
H34	1798.83	1061.8	3243.1	26
H35	2012.77	2351.78	3611.06	23
H3	4545(18)	1483(16)	3991(10)	23
H36A	4222.77	-1204.81	4620.59	55
H36B	5387.31	-1110.92	4587.51	55
H36C	4803.31	-1520.01	4075.37	55
H37	4528.14	-624.6	3251.31	32
H38	4387.25	846.66	3071.35	28
H41	4764.62	1830.33	4878.36	27
H42	4715.65	1163.46	5964.34	30
H44	4457.3	2037.51	6757.43	43
H45	4298.3	3312.65	7157.81	49
H46	4422.81	4459.84	6591.41	43
H47	4697.86	4326.89	5620.18	37
H48	4827.78	3057.61	5208.66	32
H49A	3565.12	7204.89	4987.02	66
H49B	4107.76	7716.47	5467.88	66
H49C	3675.63	6844.68	5612.25	66
H50A	5376.69	6763.4	5492.68	43
H50B	4831.89	6247.38	5012.63	43
H51A	6298.27	6401.12	4492.89	48
H51B	6792.14	7050.83	4907.06	48
H52A	6229.46	7396.39	3771.33	77
H52B	7333.84	7220.88	3960.76	77
H52C	6783.49	8010.13	4183.87	77
H49D	3120.28	8580.81	5355.61	101

H49E	3578.41	8378.24	5962.77	101
H49F	2447.77	8196.06	5840.01	101
H50C	3318.2	6988.92	5788.33	68
H50D	2914.2	7209.25	5168.83	68
H51C	4298.07	6365.89	4847.3	47
H51D	4717.05	6289.74	5480.16	47
H52D	5737.23	7083.59	4587.1	59
H52E	5991.57	6192.68	4799.23	59
H52F	6159.1	6944.69	5211.66	59
H53A	1905.9	5229.53	2486.49	111
H53B	2771.42	5044.53	2924.22	111
H53C	1656.18	4913.23	3110.05	111
H54A	2717.82	3973.63	2321.99	76
H54B	2276.13	3666.55	2912.99	76
H55A	1248	2685.11	2525.62	58
H55B	1746.99	2860.71	1922.69	58
H56A	222.69	3192.41	1538.98	74
H56B	-286.7	3064.9	2142.56	74
H56C	162.28	2310.77	1810.18	74
H53D	2027.13	5620.75	2389.64	100
H53E	2379.14	5610.74	3035.91	100
H53F	1249.76	5470.08	2885.39	100
H54C	2764.48	4382.51	2567.26	77
H54D	2059.75	4238.22	3102.38	77
H55C	1350.77	3081.38	2719.5	114
H55D	2050.93	3134.19	2175.29	114
H56D	641.05	3258.38	1591.82	138
H56E	-33.79	3148.37	2140.29	138
H56F	643.54	2422.15	1928.48	138

Supplementary Table 16. Atomic Occupancy for yoon62a

**Atom**	***Occupancy***	**Atom**	***Occupancy***	**Atom**	***Occupancy***
C8B	0.151(4)	F5B	0.151(4)	F6B	0.151(4)
C8	0.552(4)	F5	0.552(4)	F6	0.552(4)
C8A	0.297(4)	F5A	0.297(4)	F6A	0.297(4)
O7	0.837(4)	C49	0.837(4)	H49A	0.837(4)
H49B	0.837(4)	H49C	0.837(4)	C50	0.837(4)
H50A	0.837(4)	H50B	0.837(4)	C51	0.837(4)
H51A	0.837(4)	H51B	0.837(4)	C52	0.837(4)
H52A	0.837(4)	H52B	0.837(4)	H52C	0.837(4)
O7A	0.163(4)	C49A	0.163(4)	H49D	0.163(4)
H49E	0.163(4)	H49F	0.163(4)	C50A	0.163(4)
H50C	0.163(4)	H50D	0.163(4)	C51A	0.163(4)
H51C	0.163(4)	H51D	0.163(4)	C52A	0.163(4)
H52D	0.163(4)	H52E	0.163(4)	H52F	0.163(4)

O8	0.647(13)	C53	0.647(13)	H53A	0.647(13)
H53B	0.647(13)	H53C	0.647(13)	C54	0.647(13)
H54A	0.647(13)	H54B	0.647(13)	C55	0.647(13)
H55A	0.647(13)	H55B	0.647(13)	C56	0.647(13)
H56A	0.647(13)	H56B	0.647(13)	H56C	0.647(13)
O8A	0.353(13)	C53A	0.353(13)	H53D	0.353(13)
H53E	0.353(13)	H53F	0.353(13)	C54A	0.353(13)
H54C	0.353(13)	H54D	0.353(13)	C55A	0.353(13)
H55C	0.353(13)	H55D	0.353(13)	C56A	0.353(13)
H56D	0.353(13)	H56E	0.353(13)	H56F	0.353(13)

10. Computational Data

Computational Methods: All geometry optimizations were conducted using DFT[32] as implemented in the Jaguar 9.1 suite[33] of ab initio quantum chemistry programs with Becke's three-parameter exchange functional B3LYP including Grimme's D3 dispersion correction levels of theory,[34-39] together with Pople's 6-31G** basis set.[40-45] Analytical vibrational frequencies within the harmonic approximation were calculated using the 6-31G** basis to confirm proper convergence to well-defined minima or saddle points on the potential energy surface. Additional single point calculations on each optimized geometry were carried out using time-dependent DFT (TDDFT) as implemented in the Q-Chem 5.0 suite[46] of ab initio quantum chemistry programs with Handy's Coulomb-attenuating range-separated functional CAM-B3LYP including Grimme's D3 dispersion correction levels of theory.[47] Standard double-ζ quality 6-31G** basis set was used. Eigenstates of the time-dependent Hamiltonians were constructed based on configuration interaction singles (CIS), that is equivalent to the Tamm-Dancoff Approximation (TDA).[48]

Supplementary Computational Analysis

Supplementary Figure 13. PMO diagram for **1** and **1–H$^+$–AC-4**. Each molecule was fragmented into the cinnamoyl part and the remaining portion of the molecule.

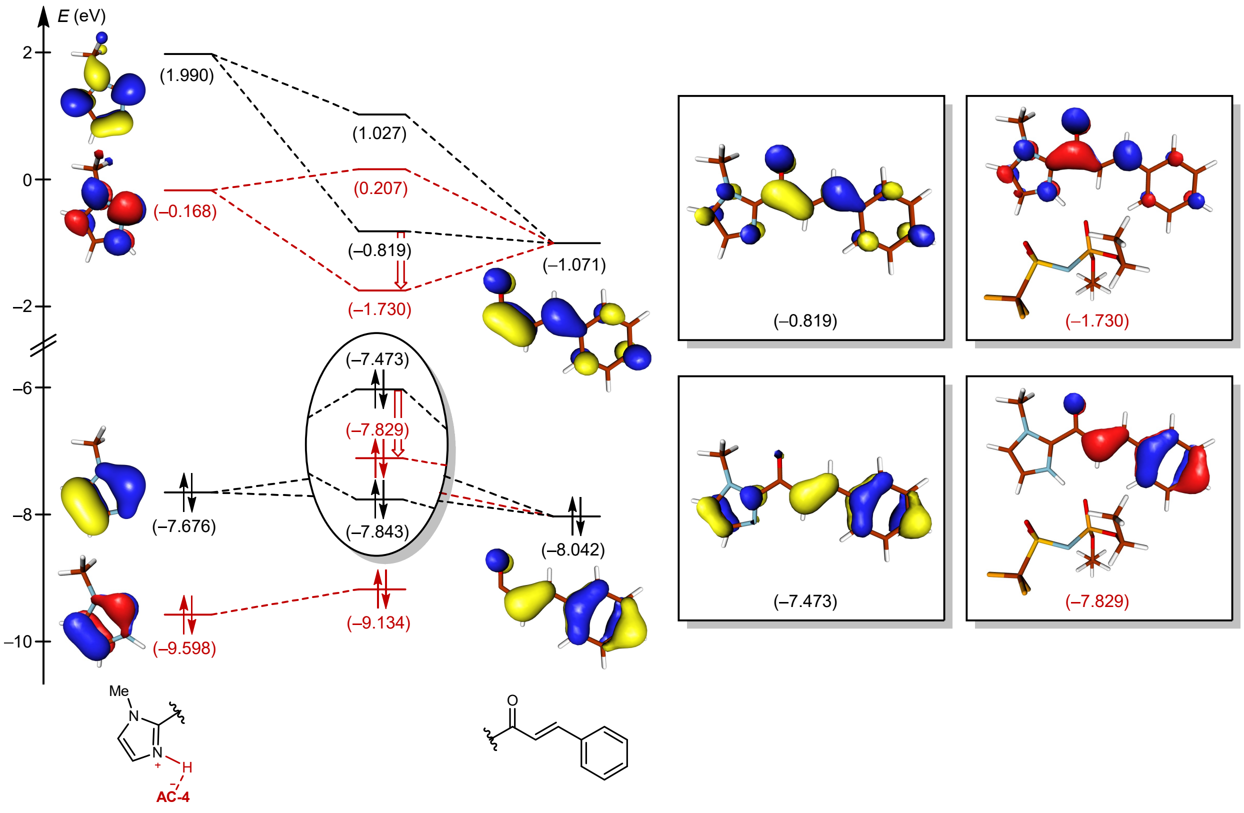

To understand the broken conjugation on FMO, we first fragmented the compound **1** and **1H$^+$–AC-4** into the cinnamoyl part and the remaining portion of the molecule. Our computation results revealed that the highest π orbital in cinnamoyl (–8.04 eV) has a node on the carbonyl carbon. Therefore, the resonance between the interacting orbitals is interrupted between this cinnamoyl π and the highest π in the imidazole ring in **1** (–7.68 eV) and the HOMO becomes dominated by the cinnamoyl-π orbital. The resulting spatial distance between the proton and the HOMO orbital leads to a modest energetic change of only 0.37 eV.

Supplementary Table 17. TDDFT calculated singlet excited states for **1**. Only orbital contributions > 10% are shown.

State	Energy (nm)	Energy (eV)	f	Contributions
1	334.8	3.703	0.00004	D(53) → V(1) (86.1%)
2	282.2	4.394	1.16149	D(56) --> V(1) (93.0%)
3	249.1	4.978	0.00653	D(54) --> V(1) (61.7%)<br>D(56) --> V(3) (20.3%)
4	243.7	5.088	0.08326	D(55) --> V(1) (91.6%)
5	231.2	5.363	0.00282	D(51) --> V(1) (81.4%)<br>D(51) --> V(2) (12.1%)
6	208.2	5.956	0.04560	D(52) --> V(1) (89.8%)
7	200.4	6.186	0.04391	D(54) --> V(1) (14.4%)<br>D(56) --> V(2) (61.5%)
8	194.9	6.362	0.06528	D(54) --> V(1) (20.5%)<br>D(54) --> V(2) (11.5%)<br>D(56) --> V(2) (15.1%)<br>D(56) --> V(3) (38.1%)

Supplementary Table 18. TDDFT calculated singlet excited states for **1–H$^+$–AC-4**. Only orbital contributions > 10% are shown.

State	Energy (nm)	Energy (eV)	f	Contributions
1	338.7	3.661	0.00014	D(122) --> V(1) (79.5%)
2	309.2	4.010	0.87410	D(129) --> V(1) (91.9%)
3	271.4	4.569	0.04493	D(127) --> V(1) (79.3%)<br>D(129) --> V(3) (10.3%)
4	264.2	4.693	0.00107	D(128) --> V(1) (97.6%)
5	234.3	5.291	0.01716	D(126) --> V(1) (79.2%)
6	232.8	5.326	0.15182	D(125) --> V(1) (80.5%)
7	220.7	5.618	0.00229	D(124) --> V(1) (70.1%)
8	213.4	5.809	0.04765	D(129) --> V(2) (70.0%)<br>D(129) --> V(3) (10.7%)

Supplementary Figure 14. Natural transition orbital (NTO) pairs of 2nd singlet excitation ($S_0 \rightarrow S_2$) for (a) **1** and (b) **1–H$^+$–AC-4**. Each pair contributes more than 96% to the related excited state.

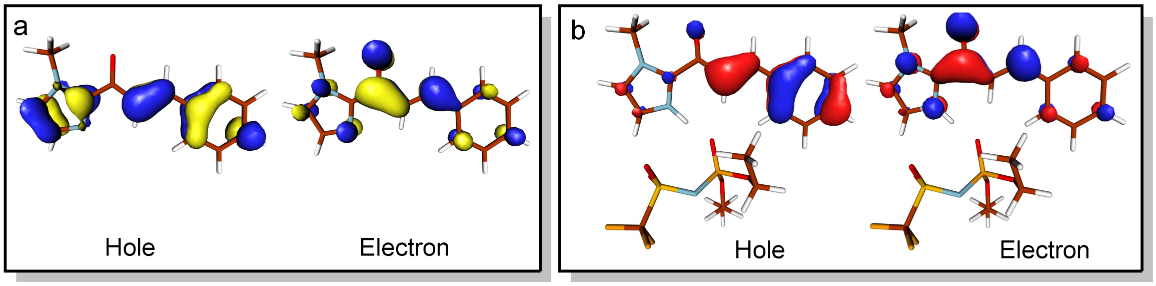

Supplementary Table 19. Cartesian coordinates of the optimized geometries. The cartesian coordinates of optimized geometries are given below in the standard XYZ format, and units are in Å.

================================

1

================================

O	6.427155495	-0.013798882	12.497465134
N	6.380298138	-0.552938819	9.674313545
N	6.262246132	1.651308775	9.317615509
C	6.474403381	-1.845172048	10.348769188
H	5.596687317	-2.009943008	10.975714684
H	7.353263378	-1.869160295	10.993429184
H	6.544090748	-2.624626160	9.587005615
C	6.316075325	-0.363703907	8.321063995
H	6.328906536	-1.195492744	7.632079601
C	6.243782997	1.002601027	8.123047829
H	6.180833817	1.533893704	7.182896137
C	6.346301079	0.699837625	10.247368813
C	6.375602245	0.931176066	11.702610016
C	6.336915970	2.346733093	12.118599892
H	6.287567139	3.084631205	11.326156616
C	6.340665817	2.670814991	13.426256180
H	6.372227192	1.840114236	14.131397247
C	6.296317101	4.011807919	14.006219864
C	6.216101646	4.141147137	15.405135155
H	6.196213245	3.243177414	16.017929077
C	6.158703327	5.394928455	16.009717941
H	6.094607353	5.471621513	17.091623306
C	6.183991432	6.548630238	15.225126266
H	6.140460491	7.528513432	15.692538261
C	6.268150330	6.438290119	13.832900047
H	6.291178703	7.333881855	13.218034744

C	6.323471546	5.187180042	13.229275703
H	6.393694401	5.118445396	12.148109436

==============================

1–H⁺–AC-4

==============================

P	-1.407773614	0.520450652	-1.871919990
O	-0.164231136	0.664911628	-1.019078016
N	-1.522876382	1.393084526	-3.231966972
S	-0.239011571	1.750511408	-4.092240334
O	0.379219294	3.045600891	-3.744997501
O	0.703656375	0.622903526	-4.302136421
O	-1.618558407	-1.047259450	-2.152693748
C	-2.404579163	-2.970228910	-3.332762480
C	-2.588449001	-1.477125049	-3.139763832
O	-2.736045361	0.898019850	-1.031085014
C	-2.333542824	3.306372881	-0.571093976
C	-3.291325808	2.238179684	-1.082799077
F	-1.968050838	2.971671104	-5.689023972
C	-1.057345867	1.994343758	-5.744192123
F	-1.656311989	0.859720528	-6.136800766
F	-0.123743474	2.321418524	-6.648033142
H	-3.123731136	-3.344753504	-4.069211960
H	-1.393488646	-3.183697939	-3.691421986
H	-2.558938265	-3.504368305	-2.390373707
H	-3.594598293	-1.238955021	-2.772246361
H	-2.418439865	-0.924975991	-4.070244312
H	-2.845417976	4.275095940	-0.563575327
H	-2.005577087	3.081357718	0.447150946
H	-1.458104610	3.391085386	-1.220987558
H	-3.590227842	2.449120045	-2.114103079

H	-4.186699867	2.180752277	-0.456925184
C	2.545766354	2.367328644	1.087745190
O	3.353884935	2.886400223	1.862089992
N	4.155934334	2.533445597	-0.853134990
C	5.257688046	3.192629814	-0.143432140
C	4.180199623	2.197299719	-2.187635899
C	2.957149744	2.162981033	-0.336523533
N	2.246168375	1.604953885	-1.334408879
H	1.249796391	1.210805058	-1.265318036
C	2.977432728	1.614094496	-2.487123251
H	5.598648071	2.565735340	0.679095328
H	4.917398453	4.140673637	0.270473778
H	6.063294888	3.356174469	-0.860095203
H	5.036273003	2.407986641	-2.807801485
H	2.563154697	1.232409239	-3.409425974
C	1.202345371	1.926411033	1.430601001
H	0.591178358	1.497008562	0.648649037
C	0.712462544	2.046100616	2.686457396
H	1.355920553	2.490784645	3.444935322
C	-0.621438026	1.632383823	3.102500439
C	-1.031252742	1.866956353	4.428695202
C	-1.527670860	1.012682557	2.212691069
H	-0.341636807	2.341548443	5.122364998
C	-2.306092978	1.503654957	4.855424881
H	-2.607722282	1.691848874	5.881597996
C	-3.194145679	0.899906158	3.962406874
H	-4.189340591	0.617787480	4.294123173
C	-2.799879313	0.656728029	2.641683102
H	-3.481677055	0.190041915	1.937102437
H	-1.245238423	0.808451891	1.187050581

Supplementary Table 20. Vibrational frequencies (in cm-1) of the optimized structures

==============================

1

==============================

30.53	40.09	60.45	98.84	118.22	148.12
186.67	204.87	216.07	238.68	286.09	310.51
407.45	413.42	453.60	501.17	546.47	587.62
630.06	632.42	667.74	702.96	711.64	752.69
767.92	791.86	798.37	855.63	867.26	906.88
913.04	935.59	939.37	977.26	1001.96	1013.67
1043.64	1047.24	1060.91	1085.16	1106.57	1116.51
1153.65	1197.91	1198.92	1218.30	1242.97	1257.31
1322.91	1335.41	1348.08	1371.79	1374.33	1406.65
1451.44	1463.81	1487.27	1492.28	1504.27	1527.35
1540.42	1552.41	1630.56	1655.99	1668.89	1732.38
3073.41	3155.86	3158.09	3166.80	3172.90	3179.67
3189.55	3199.48	3206.71	3224.60	3248.68	3276.49

==============================

1–H$^+$–AC-4

==============================

2.79	16.72	20.78	28.35	37.84	39.83
44.90	51.62	53.03	59.95	65.33	71.93
79.37	89.47	91.21	104.63	113.82	120.57
135.01	163.16	169.31	180.98	186.81	196.62
199.82	205.22	228.16	254.20	257.02	269.70
274.91	285.81	292.15	299.84	317.11	323.34
348.20	349.77	407.11	410.08	417.82	436.64
450.82	458.75	498.68	499.03	513.07	542.34
543.94	550.65	564.25	586.70	630.58	631.55
634.62	644.62	702.14	703.99	716.00	750.22

757.96 779.07 793.15 794.80 800.51 824.98

832.63 844.06 866.85 891.48 897.17 927.50

931.43 934.42 947.98 978.40 992.50 1013.29

1016.27 1031.24 1052.15 1058.24 1062.27 1065.98

1071.74 1078.31 1105.65 1122.75 1123.86 1129.91

1131.58 1152.50 1153.05 1163.85 1187.39 1191.51

1197.62 1199.02 1223.98 1225.15 1229.81 1242.44

1246.17 1257.57 1263.58 1312.81 1322.68 1328.71

1332.82 1365.82 1378.05 1396.65 1406.92 1408.78

1419.62 1439.32 1440.36 1446.59 1467.06 1486.65

1493.70 1496.81 1505.19 1508.44 1510.16 1516.34

1520.79 1523.82 1537.60 1539.78 1544.67 1627.31

1648.31 1660.63 1675.20 1730.92 2559.50 3040.20

3049.66 3051.18 3066.34 3091.16 3097.39 3115.34

3123.98 3125.02 3137.03 3149.05 3164.27 3176.42

3184.13 3186.36 3187.91 3199.53 3208.65 3227.68

3245.00 3268.12 3306.06

12. NMR Data

Supplementary Figure 15a. ^{1}H NMR of **AC-3**.

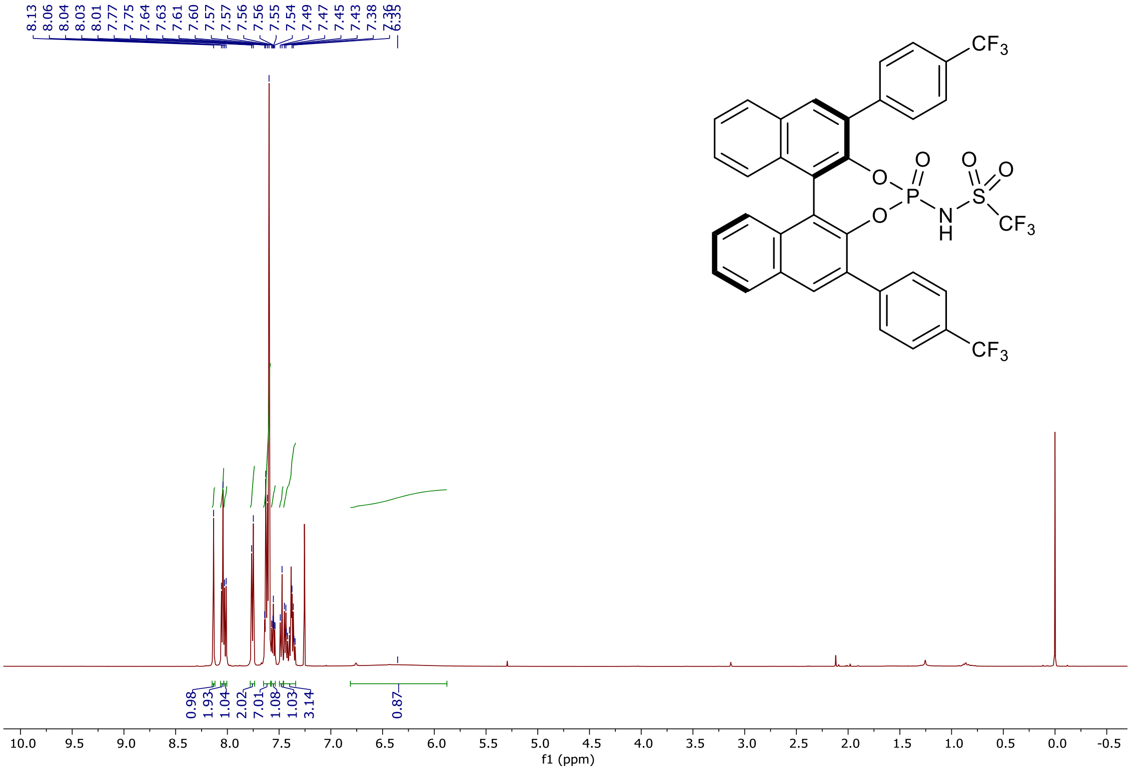

Supplementary Figure 15b. ^{13}C NMR of **AC-3**.

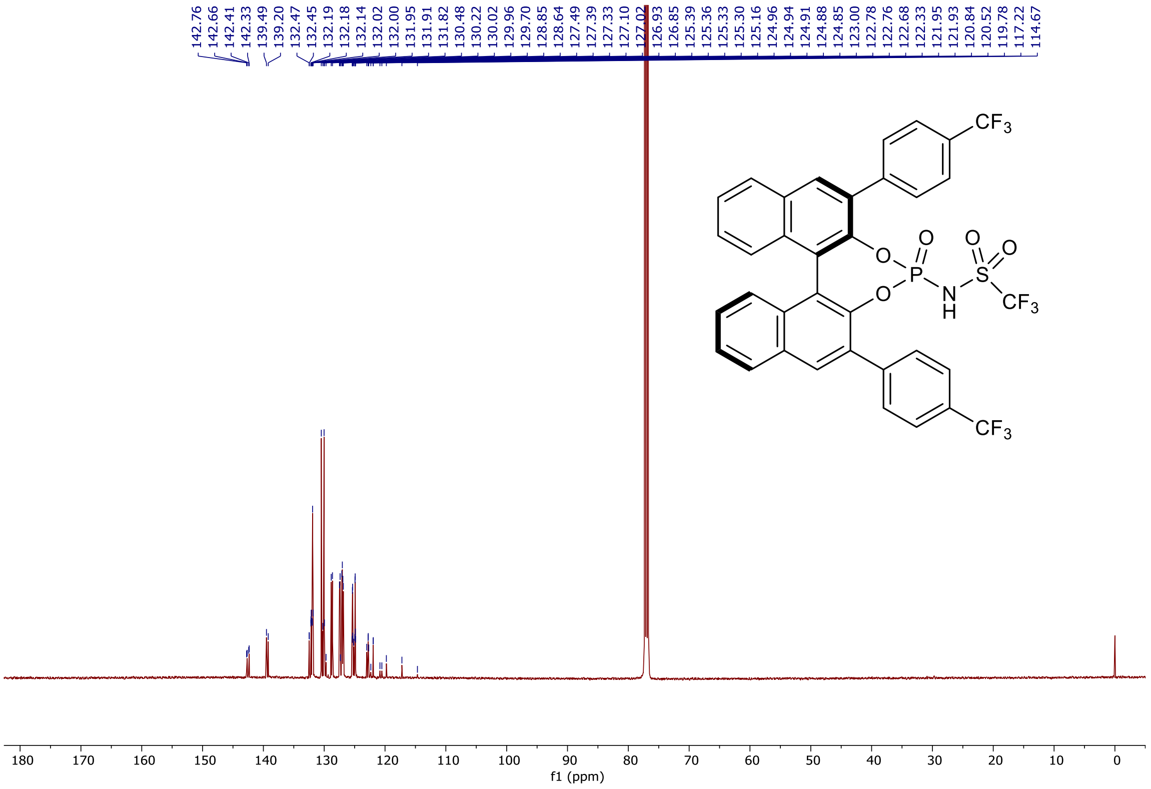

Supplementary Figure 15c. ^{19}F NMR of **AC-3**.

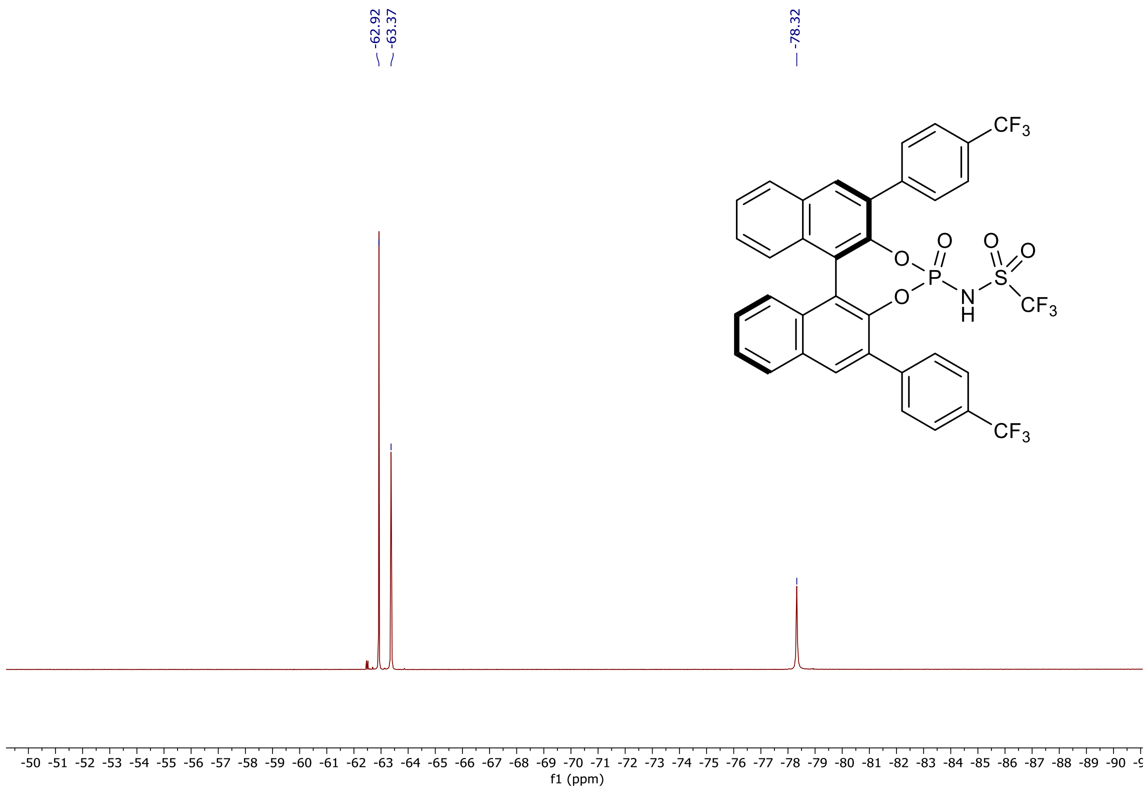

Supplementary Figure 15d. ^{31}P NMR of **AC-3**.

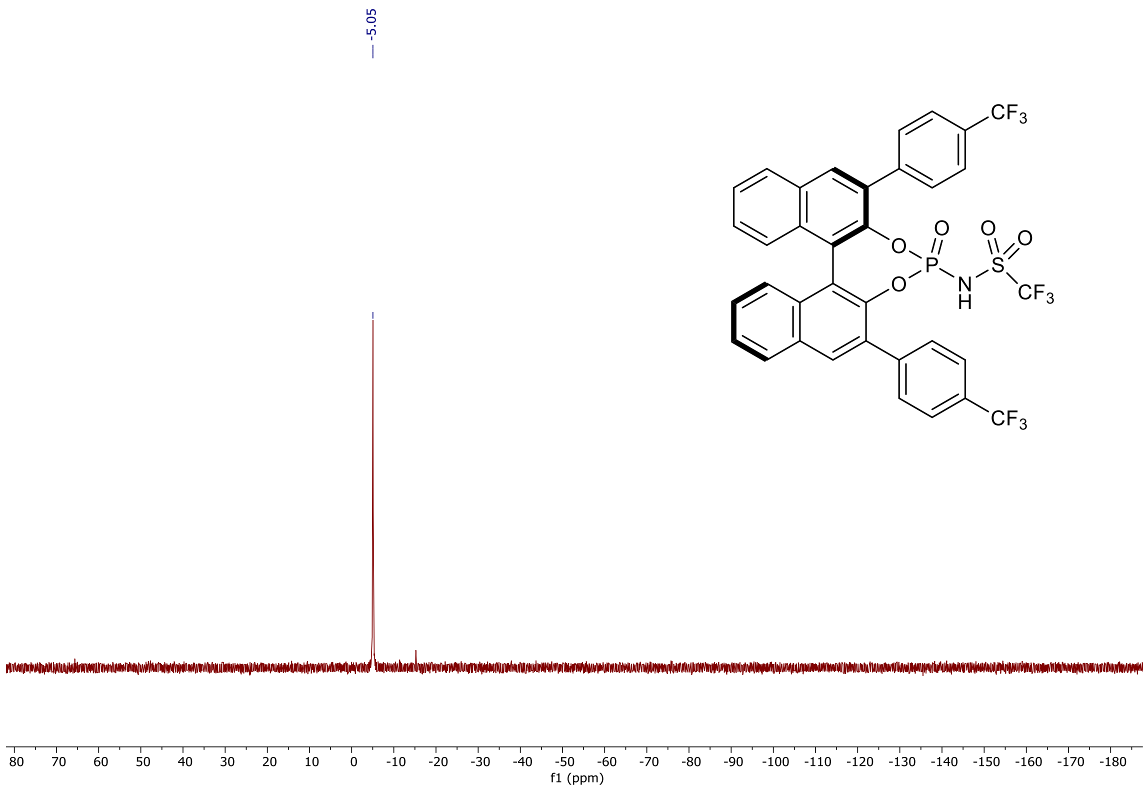

Supplementary Figure 16a. ^{1}H NMR of **4a**.

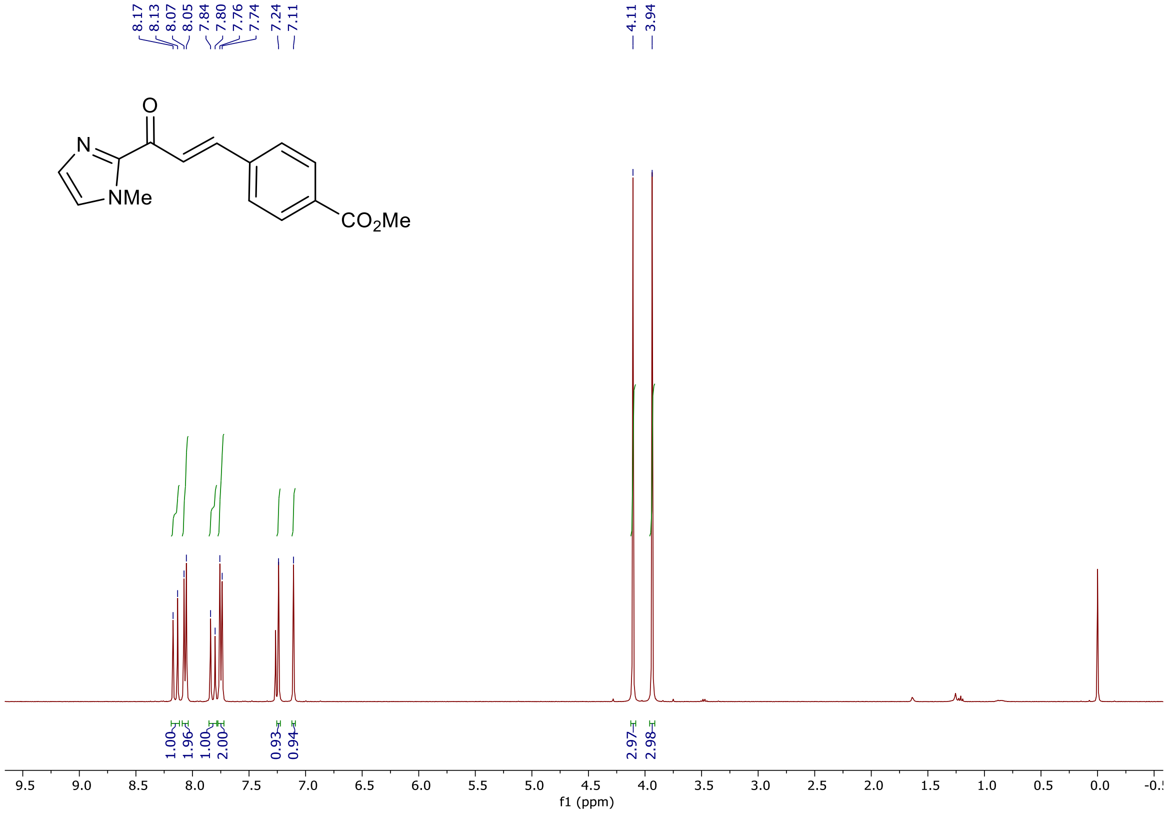

Supplementary Figure 16b. ^{13}C NMR of **4a**.

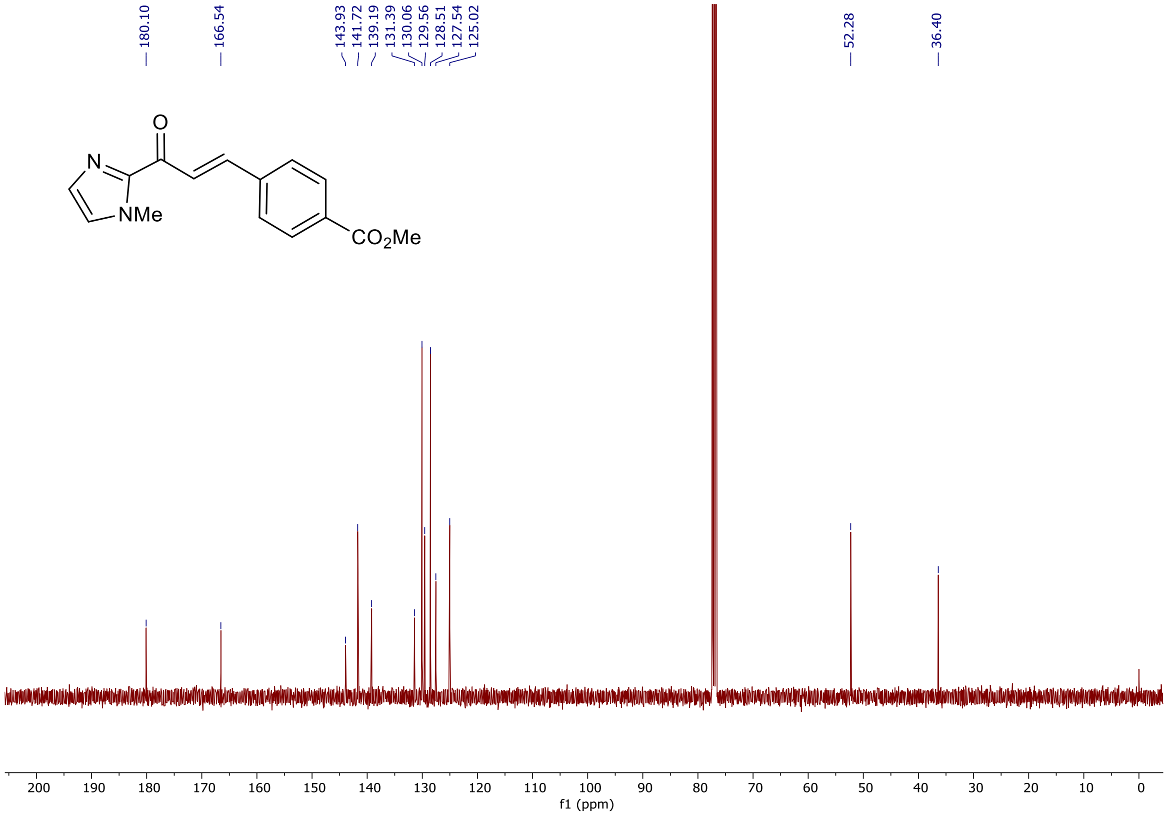

Supplementary Figure 17a. ^{1}H NMR of **6a**.

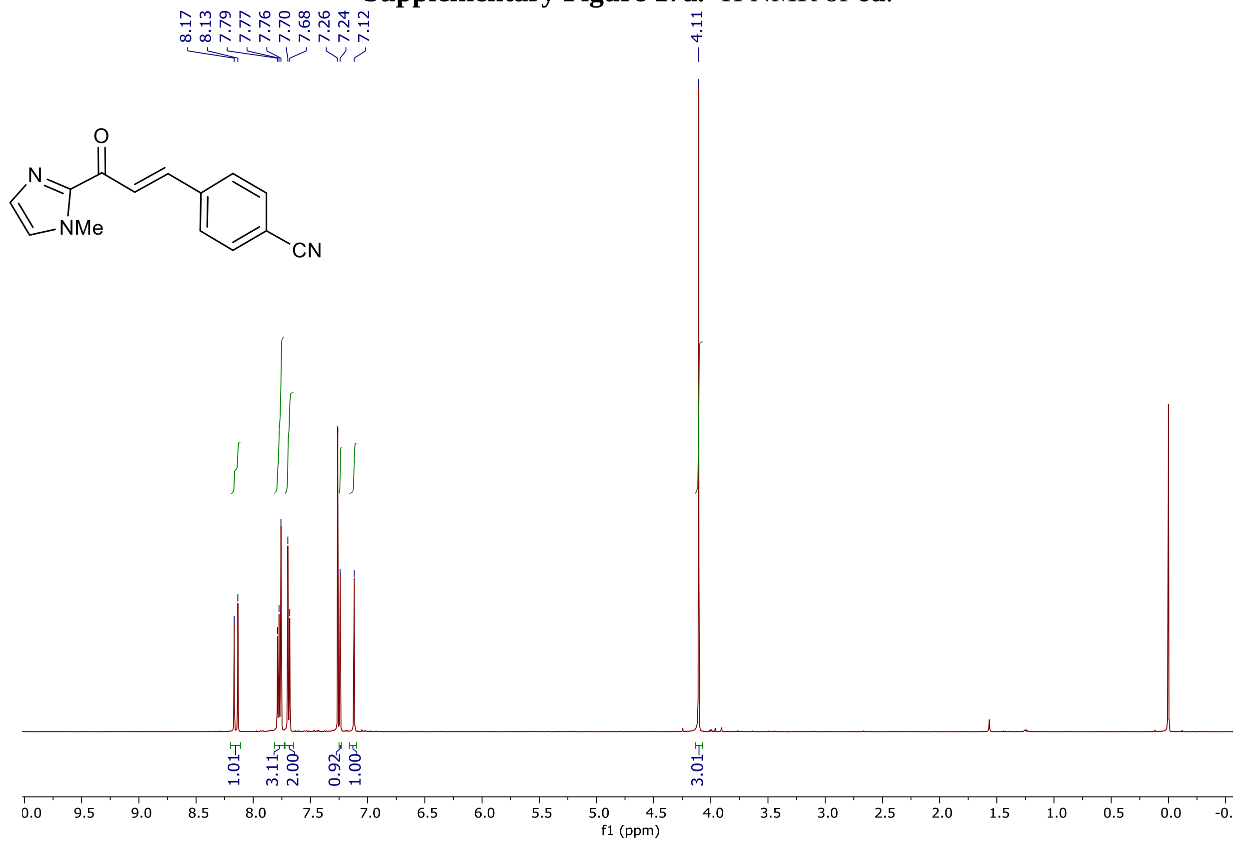

Supplementary Figure 17b. ^{13}C NMR of **6a**.

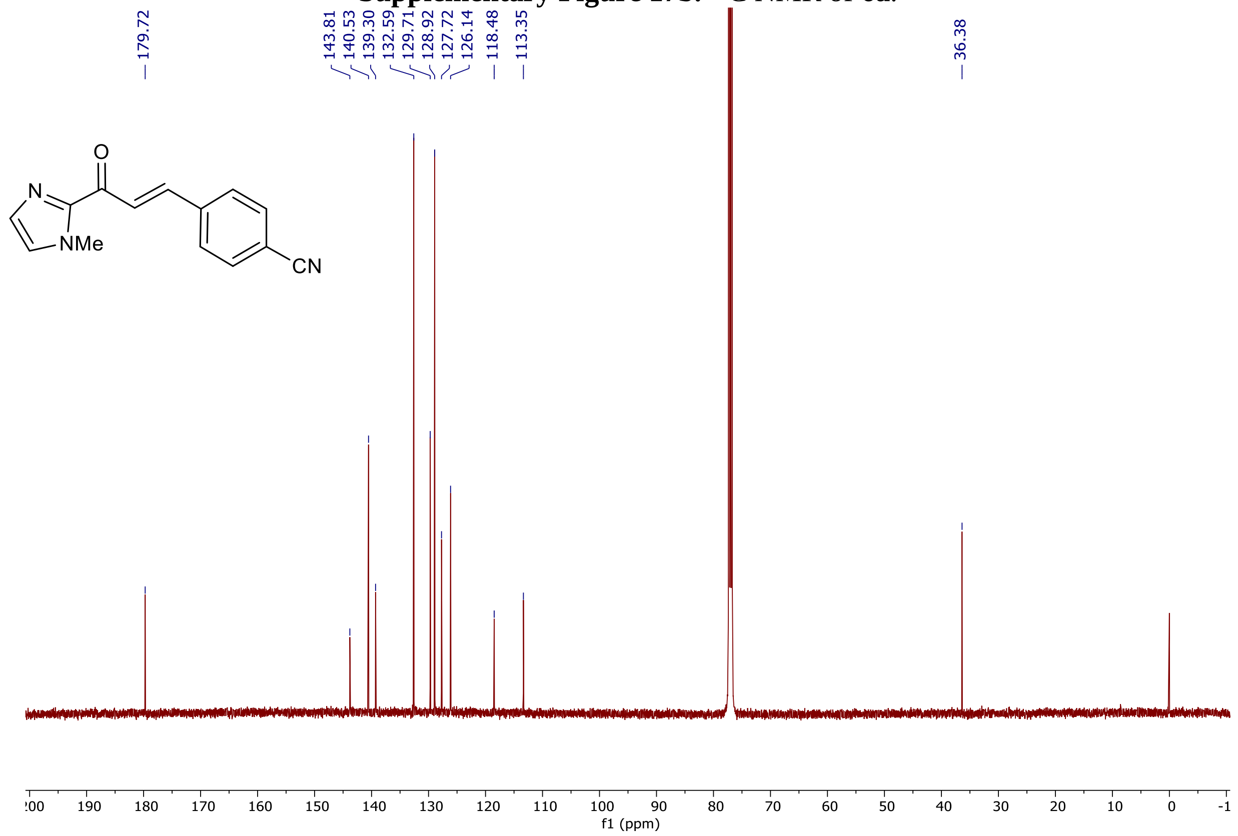

Supplementary Figure 18a. ^{1}H NMR of **7a**.

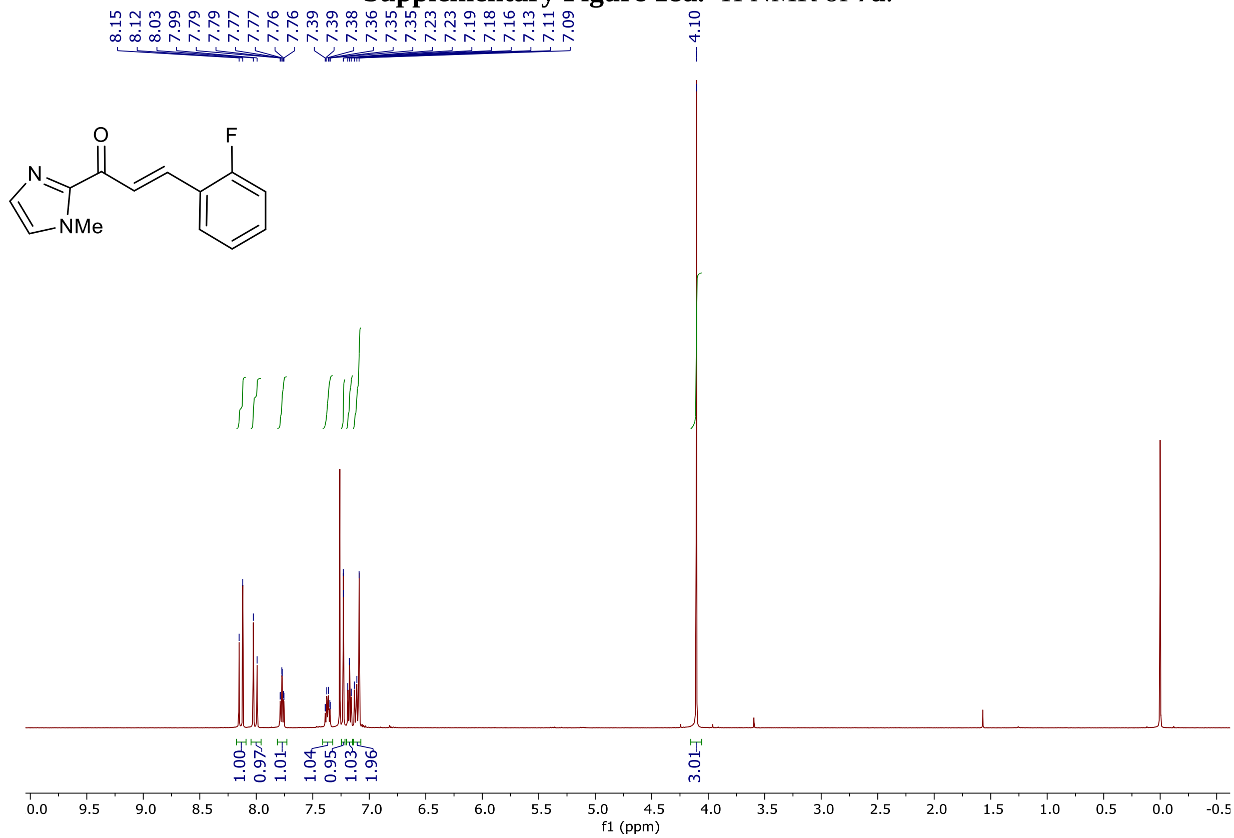

Supplementary Figure 18b. ^{13}C NMR of **7a**.

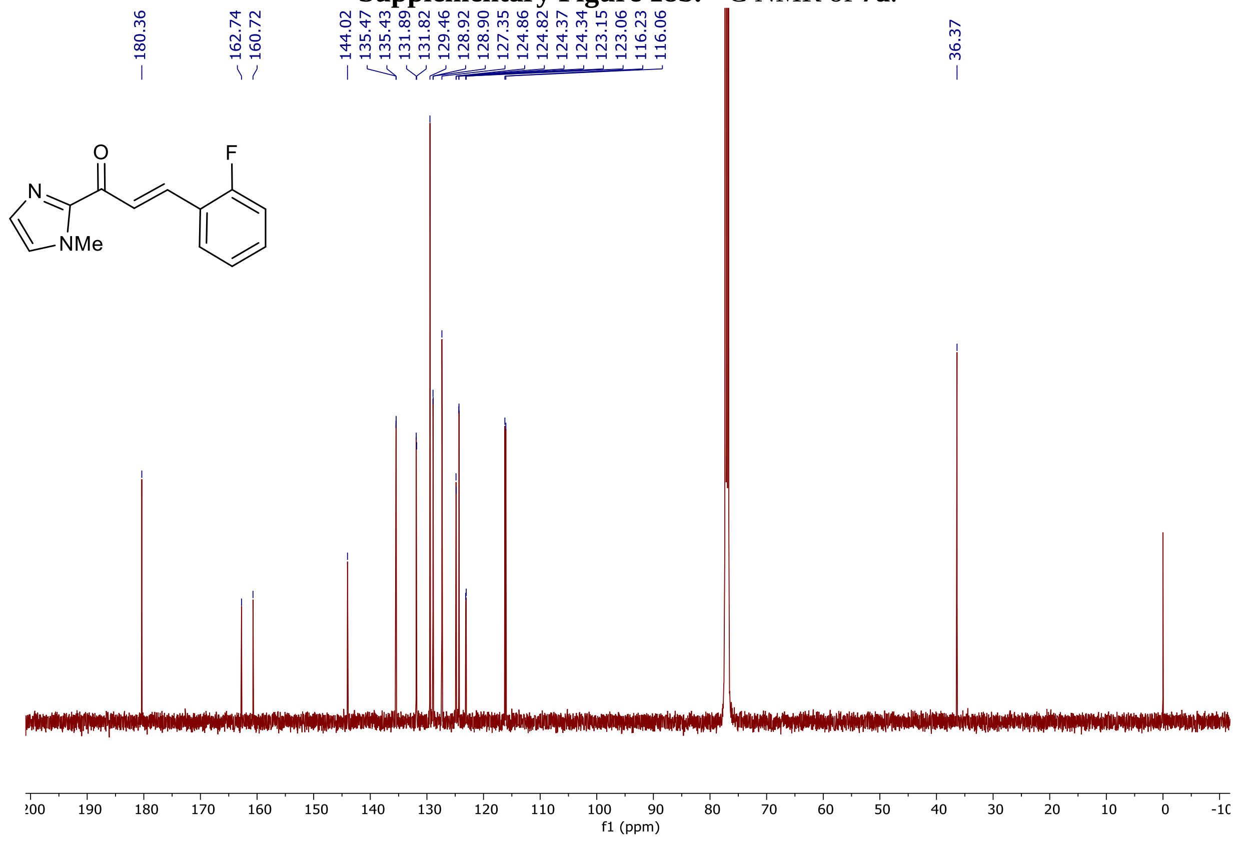

Supplementary Figure 18c. ^{19}F NMR of **7a**.

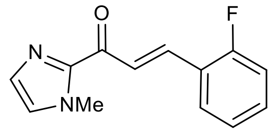

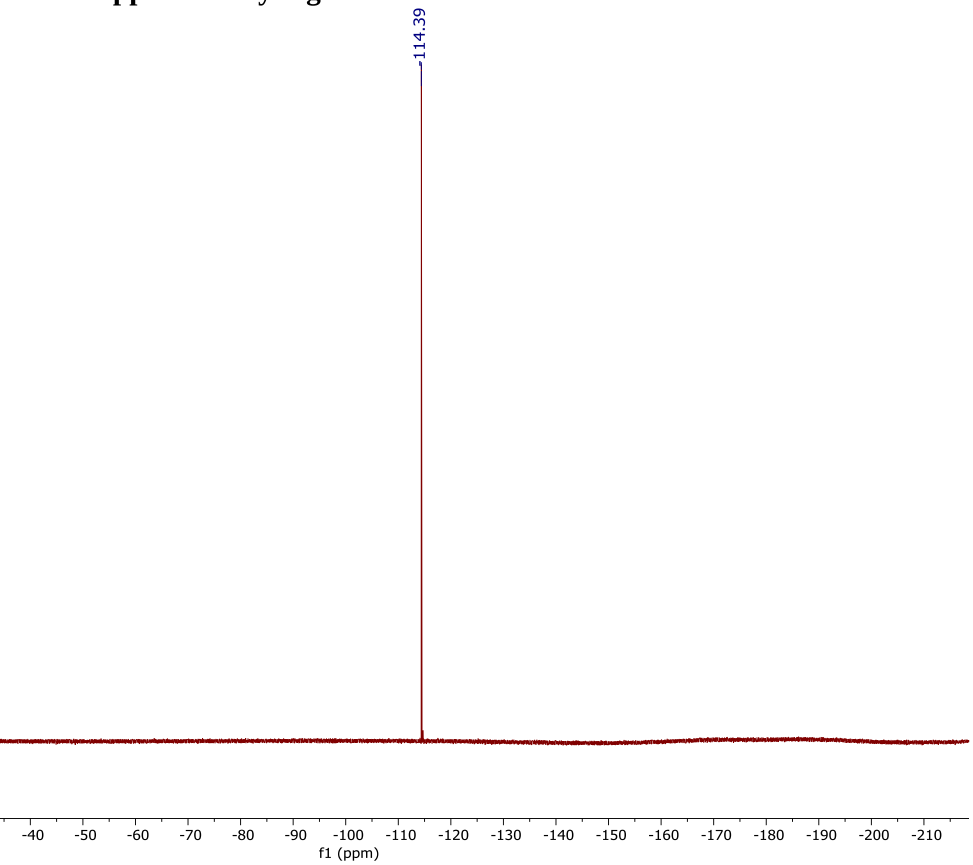

Supplementary Figure 19a. ^{1}H NMR of **10a**.

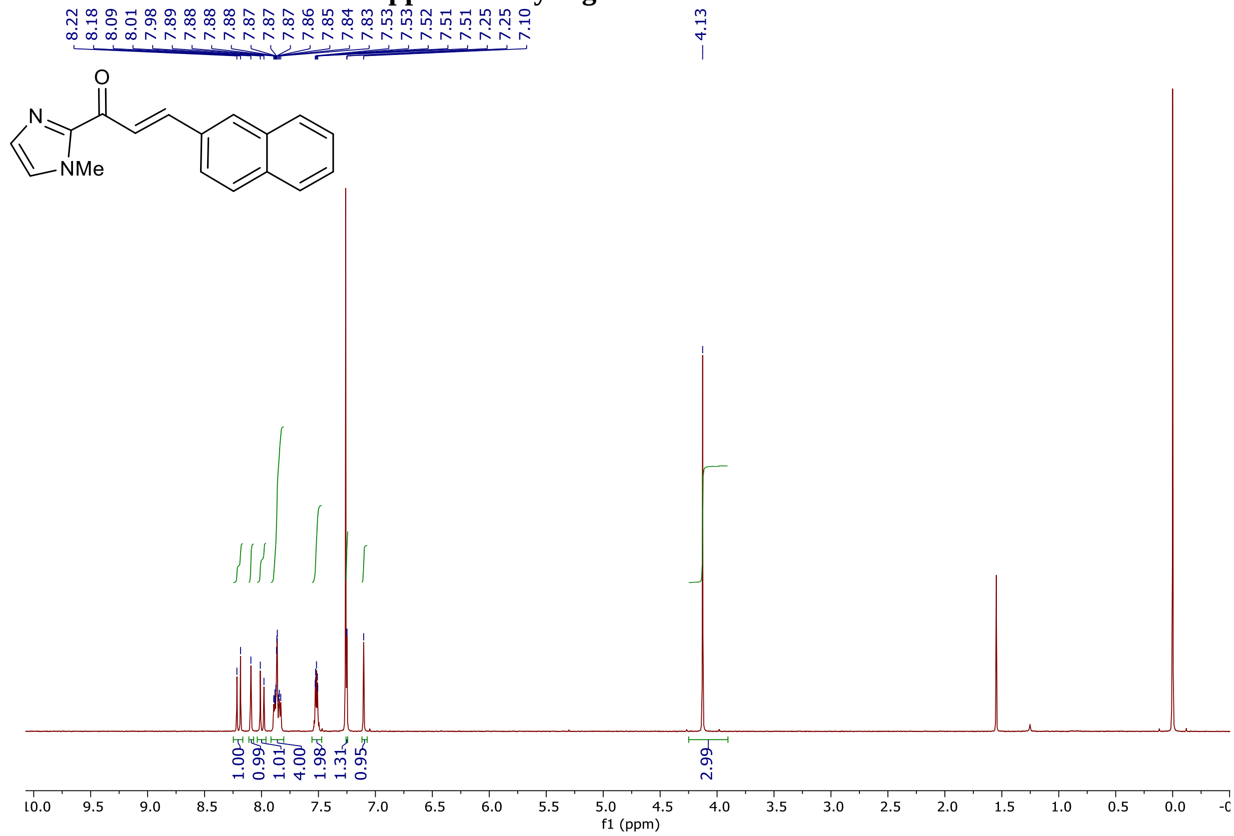

Supplementary Figure 19b. ^{13}C NMR of **10a**.

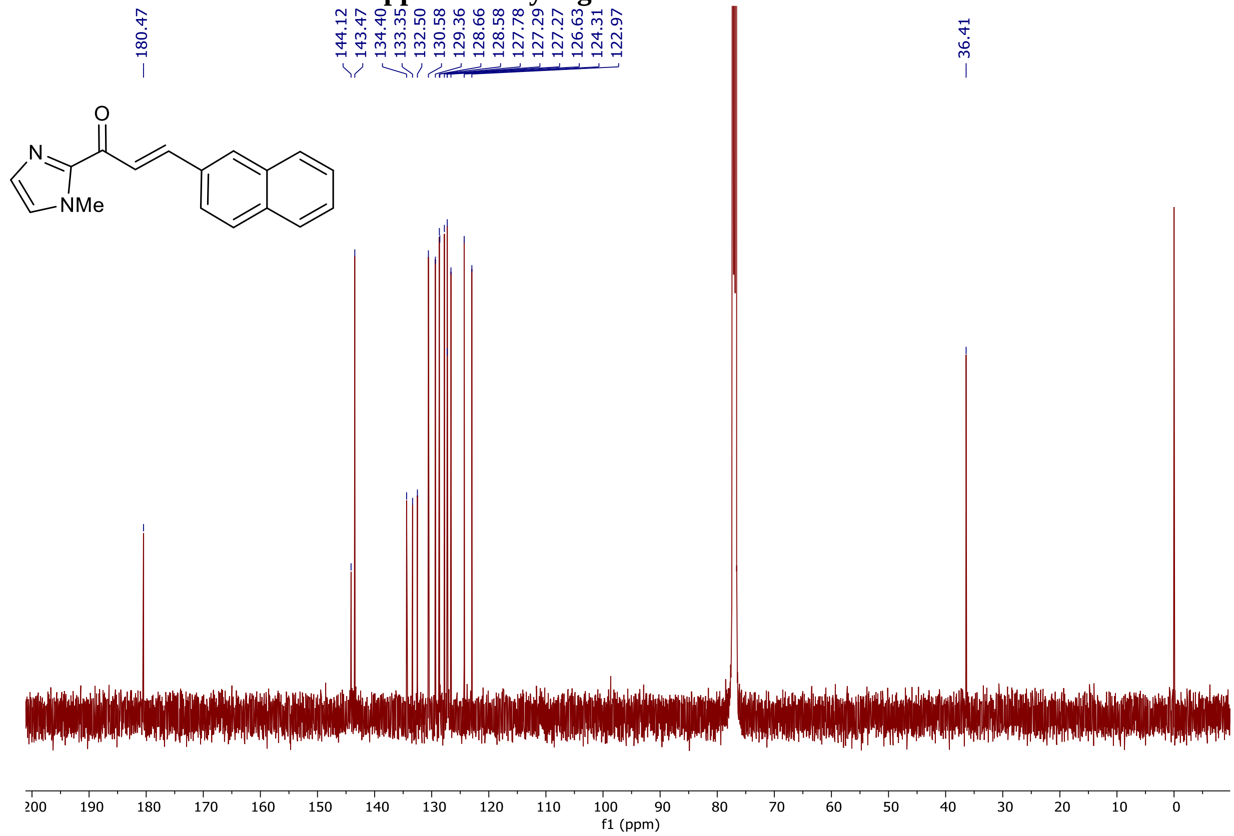

Supplementary Figure 20a. ^{1}H NMR of **12a**.

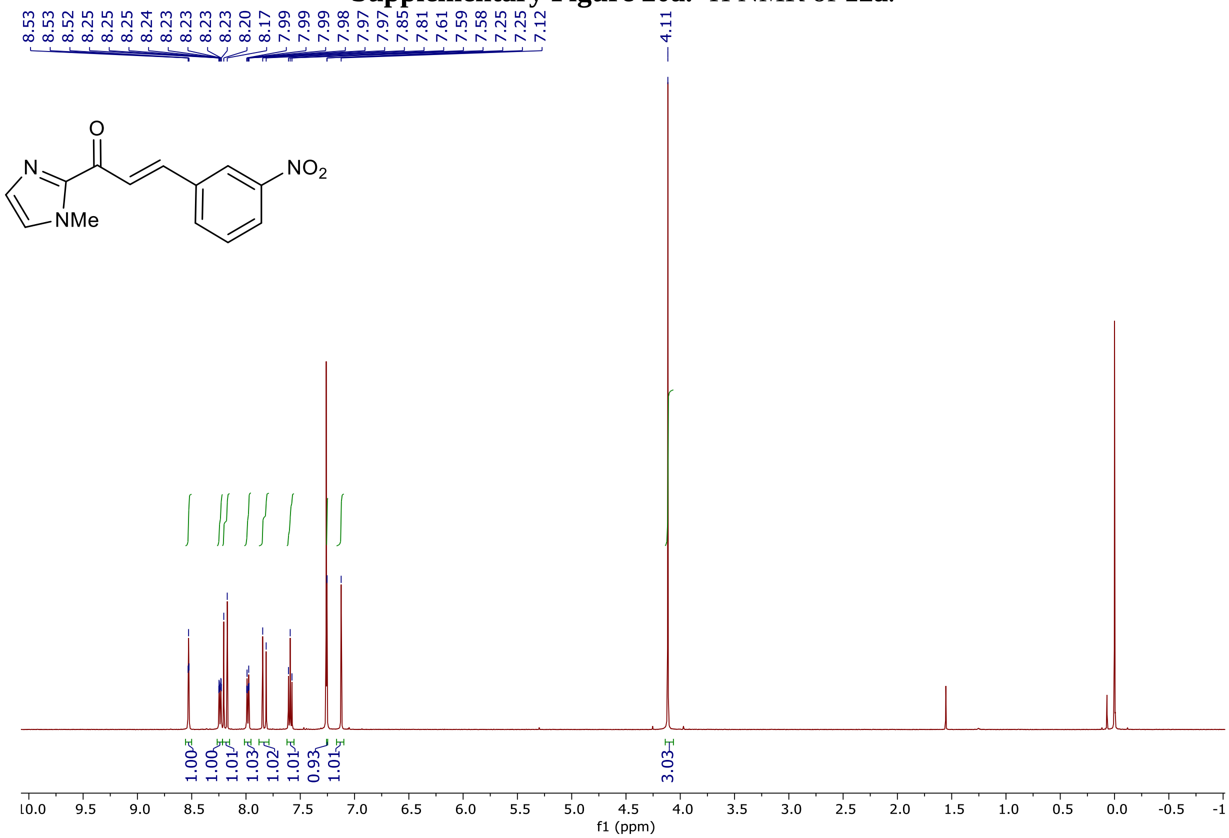

Supplementary Figure 20b. ^{13}C NMR of **12a**.

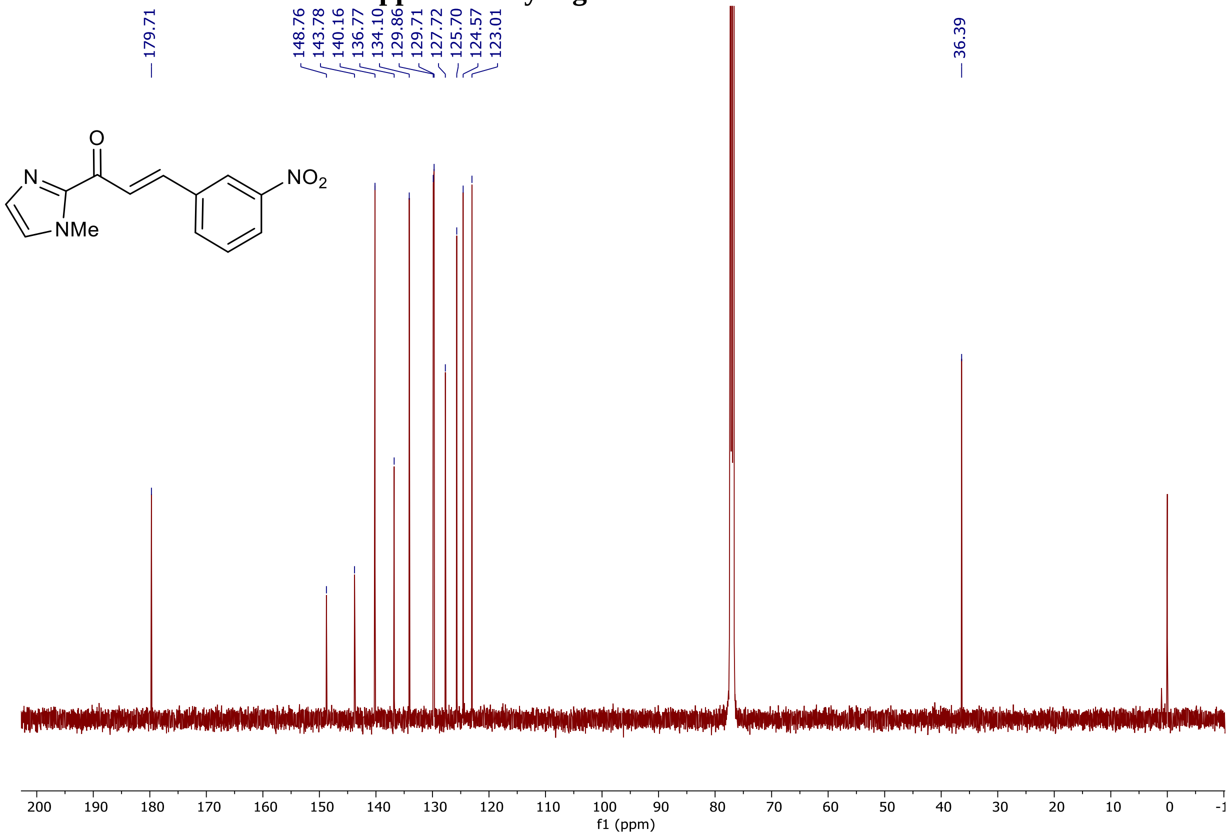

Supplementary Figure 21a. ^{1}H NMR of **36a**.

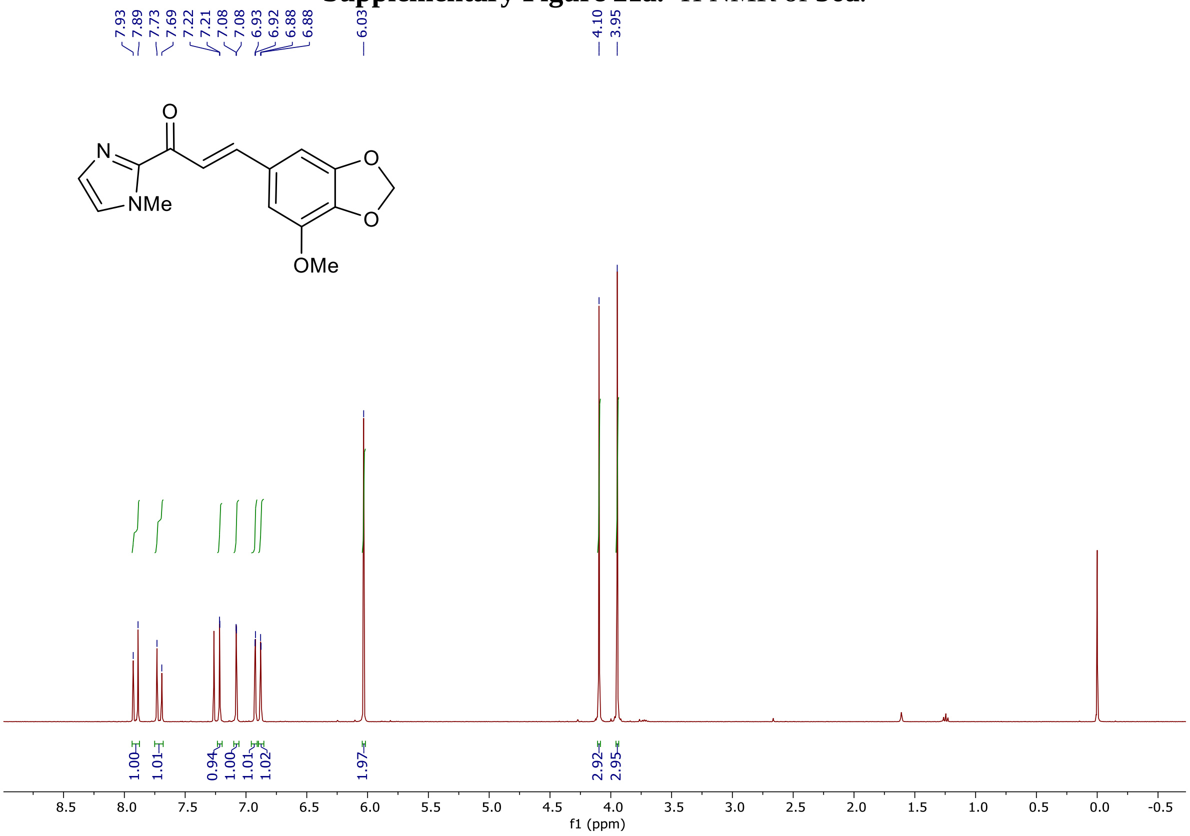

Supplementary Figure 21b. ^{13}C NMR of **36a**.

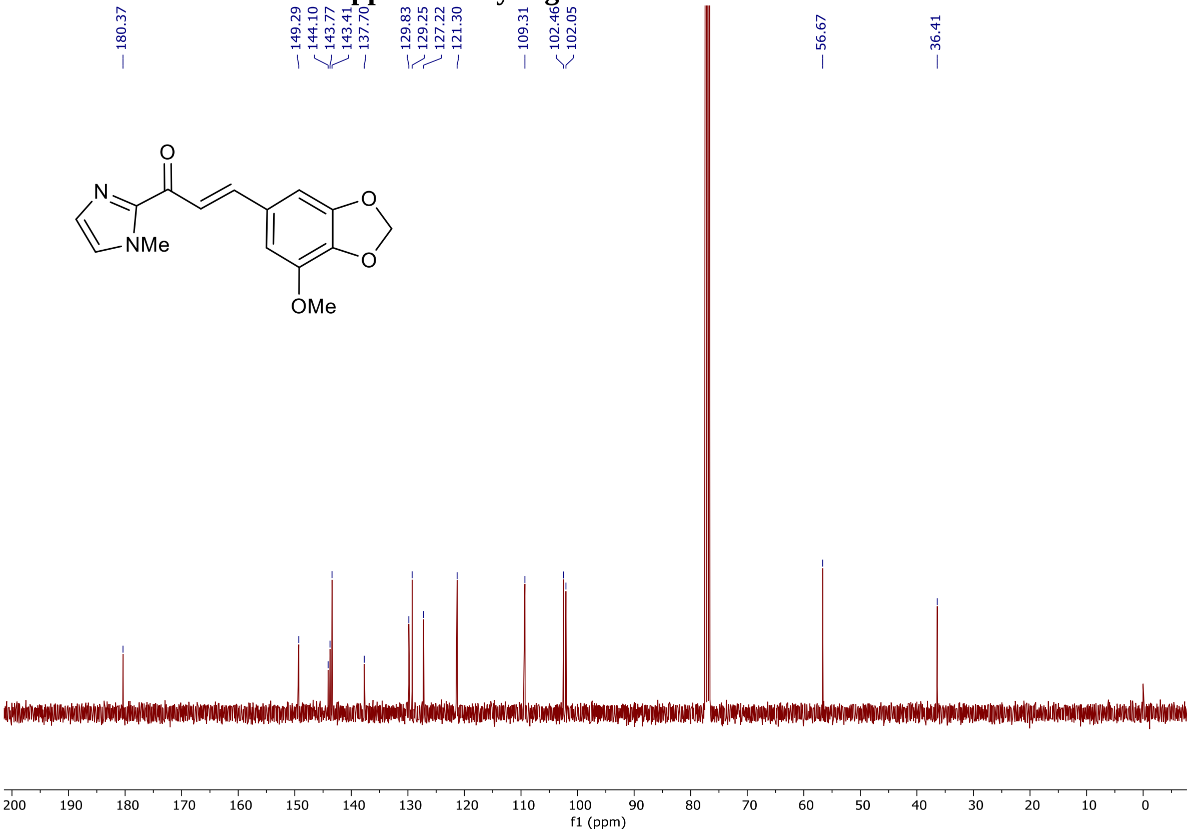

Supplementary Figure 22a. ^{1}H NMR of **4**.

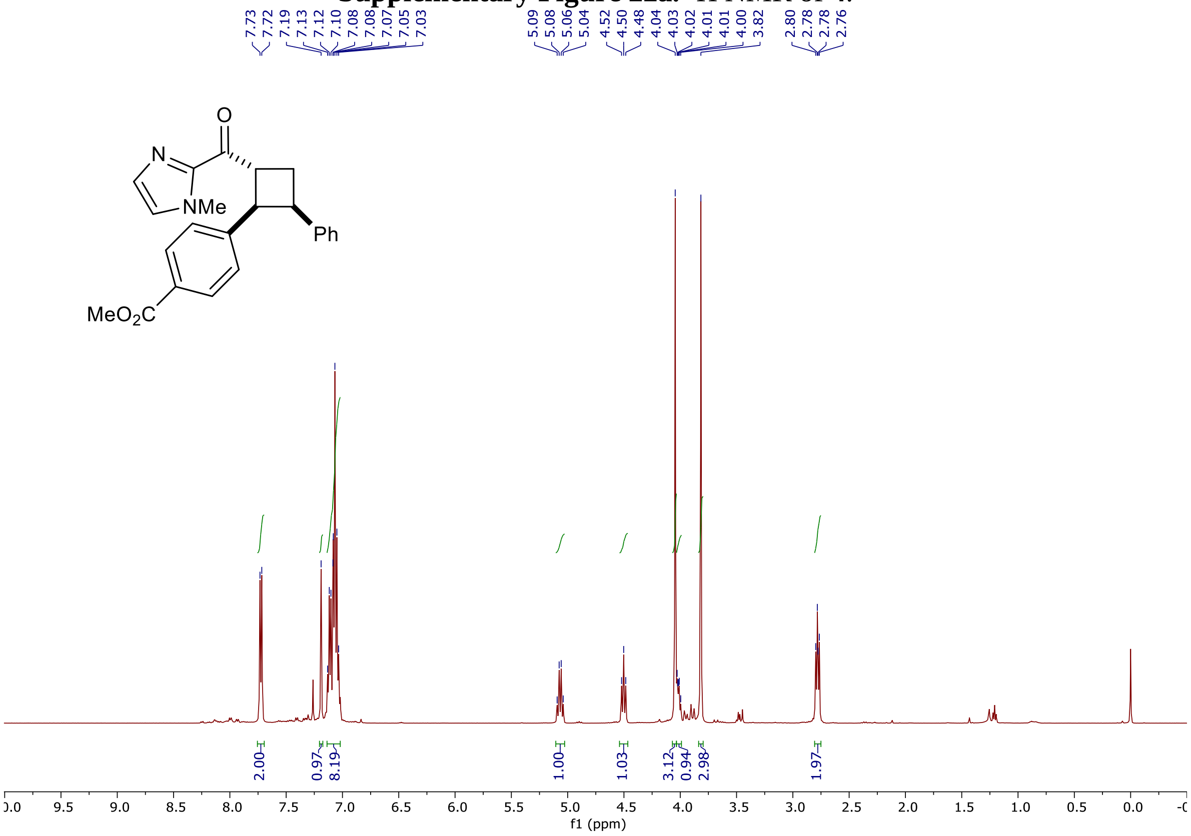

Supplementary Figure 22b. ^{13}C NMR of **4**.

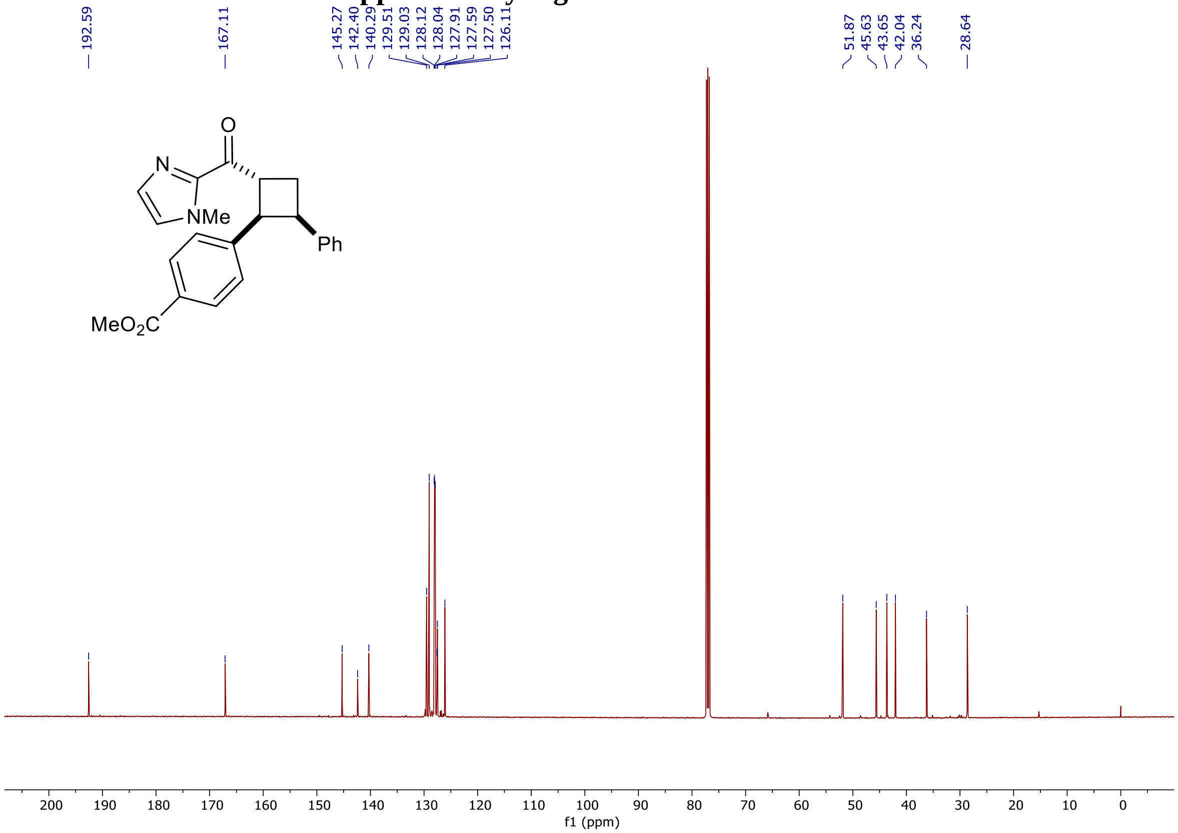

Supplementary Figure 23a. ^{1}H NMR of **6**.

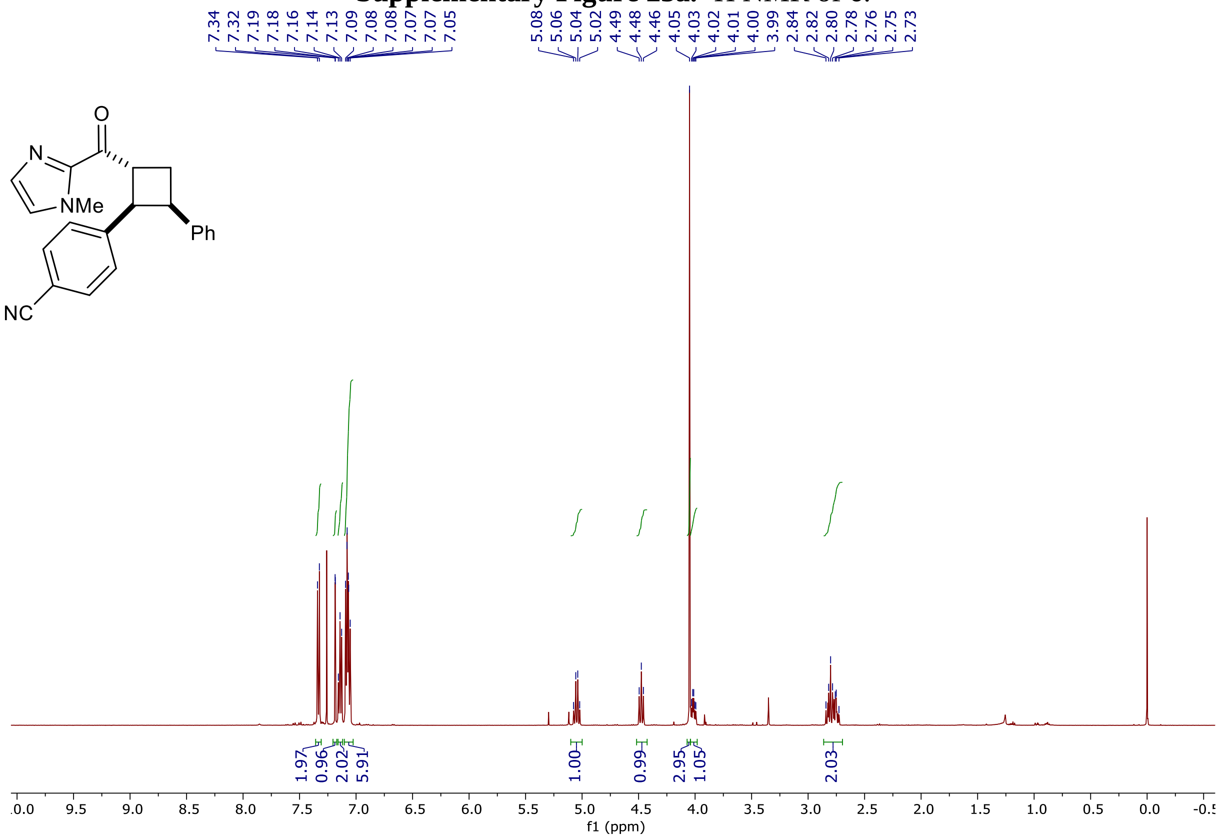

Supplementary Figure 23b. ^{13}C NMR of **6**.

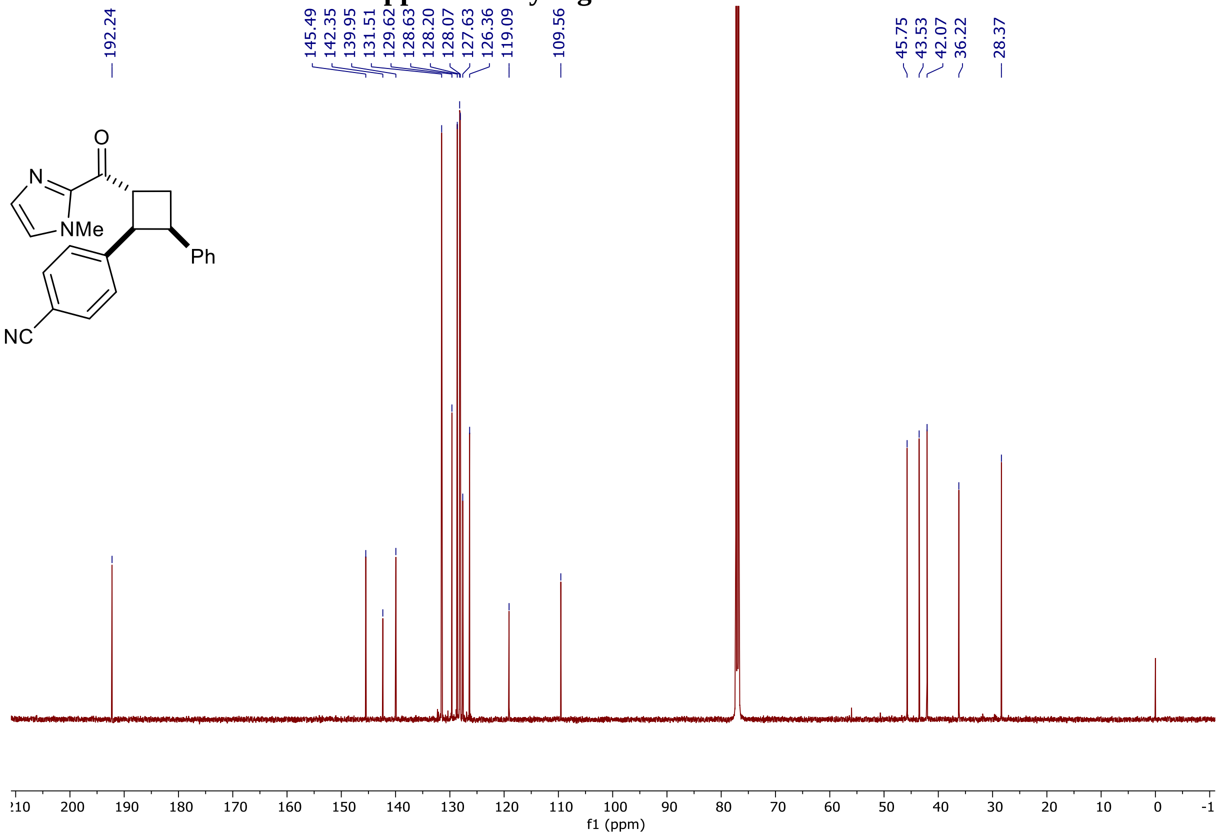

Supplementary Figure 24a. ^{1}H NMR of **7**.

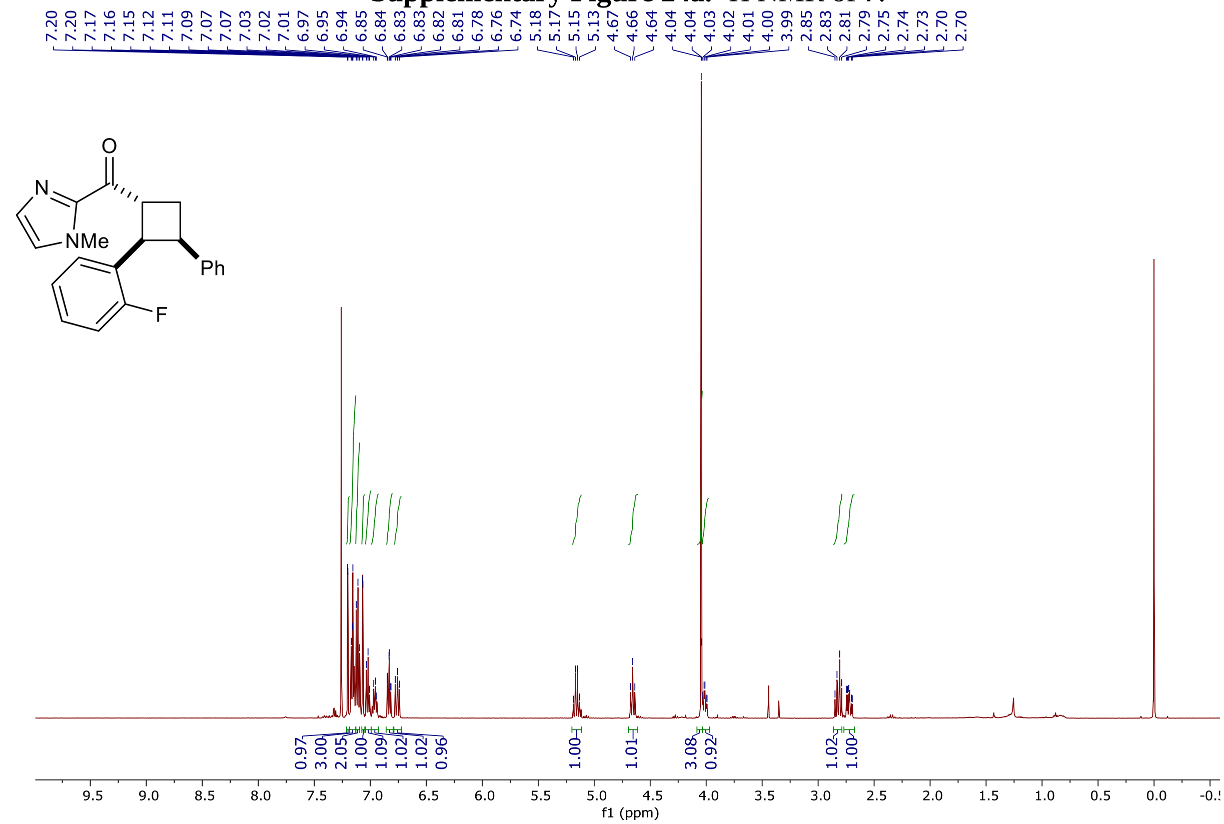

Supplementary Figure 24b. ^{13}C NMR of **7**.

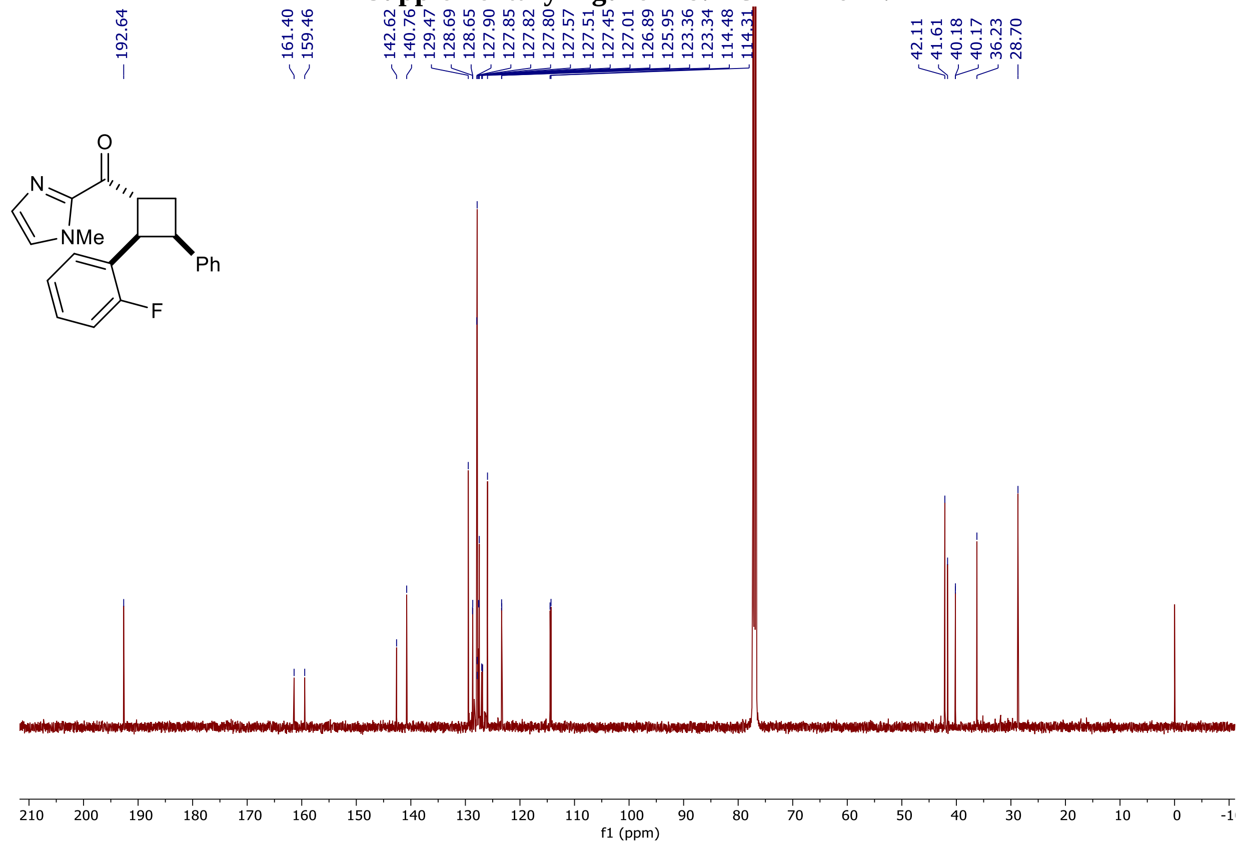

Supplementary Figure 24c. ^{19}F NMR of **7**.

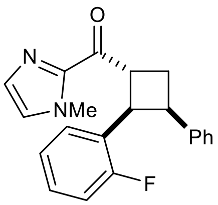

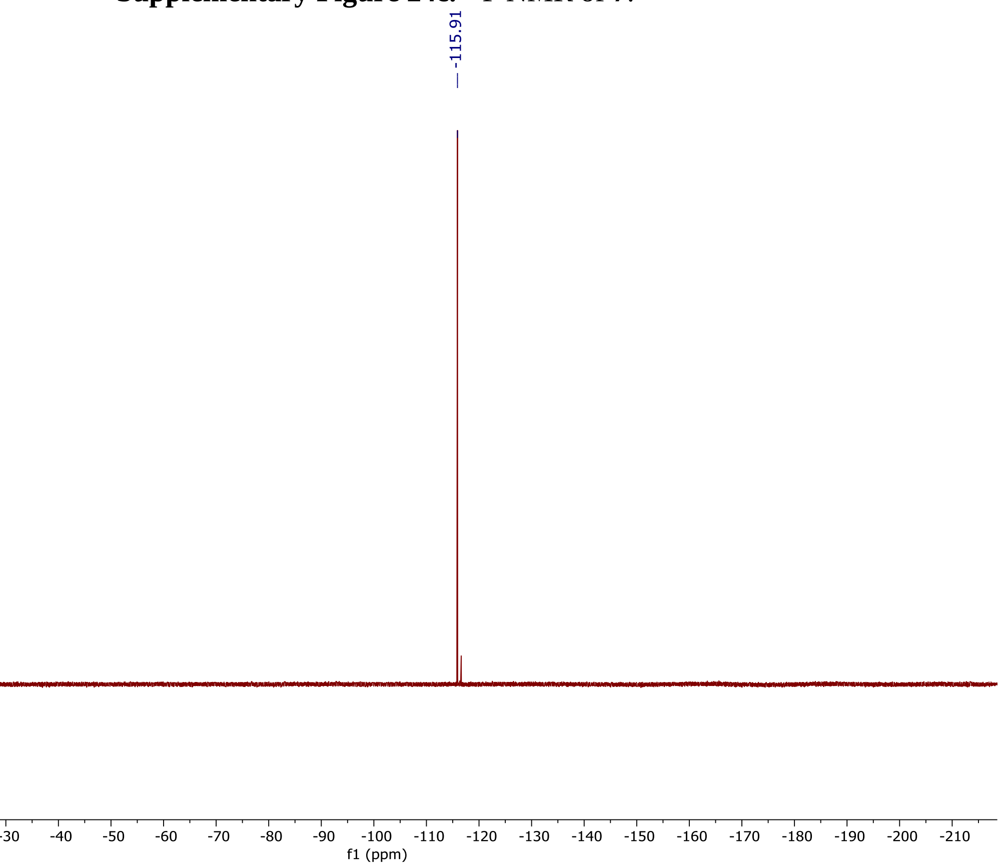

Supplementary Figure 25a. ^{1}H NMR of **8**.

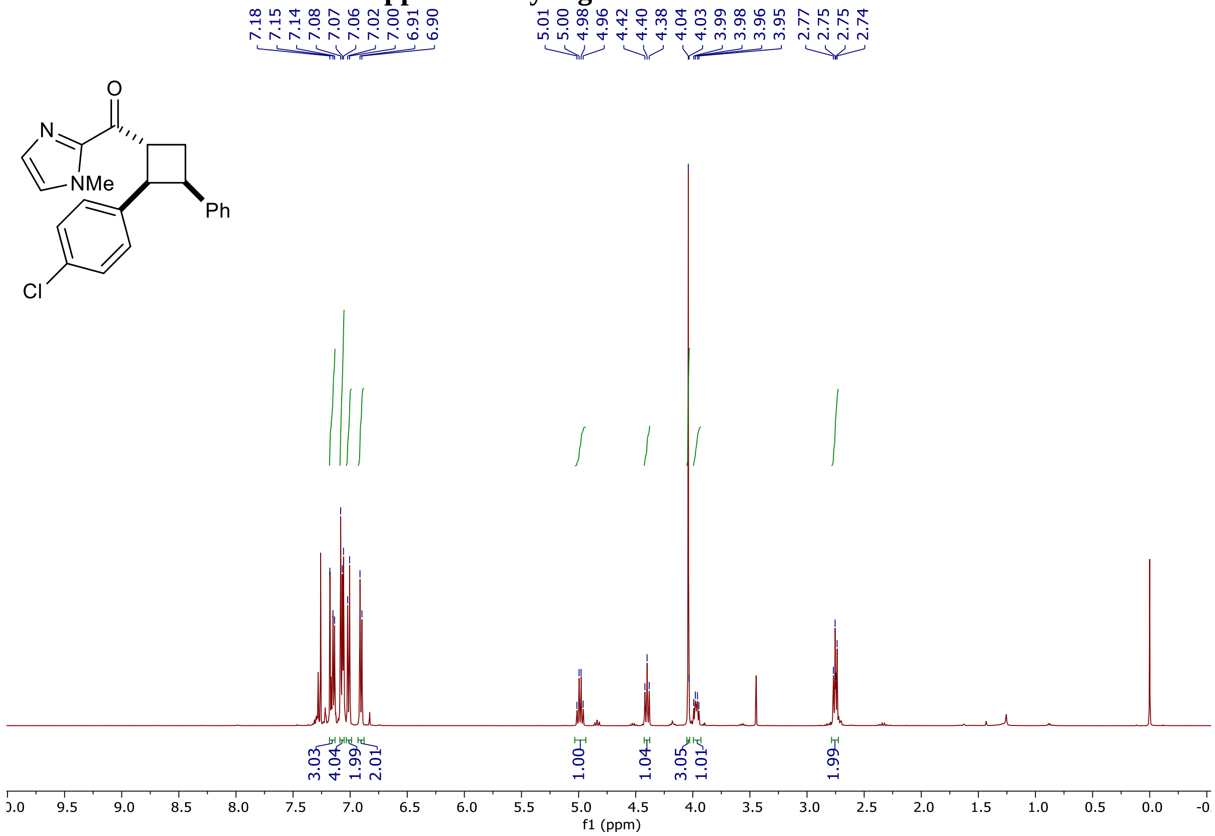

Supplementary Figure 25b. ^{13}C NMR of **8**.

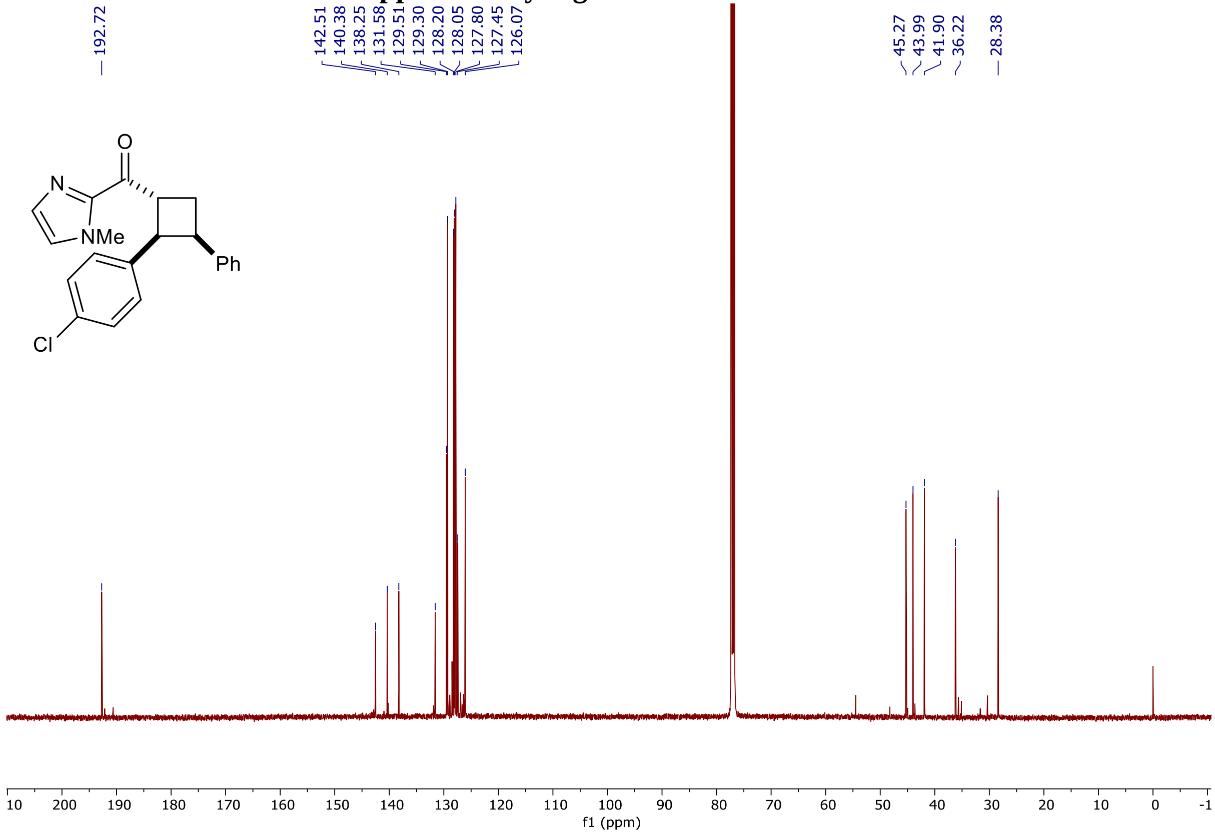

Supplementary Figure 26a. ^{1}H NMR of **9**.

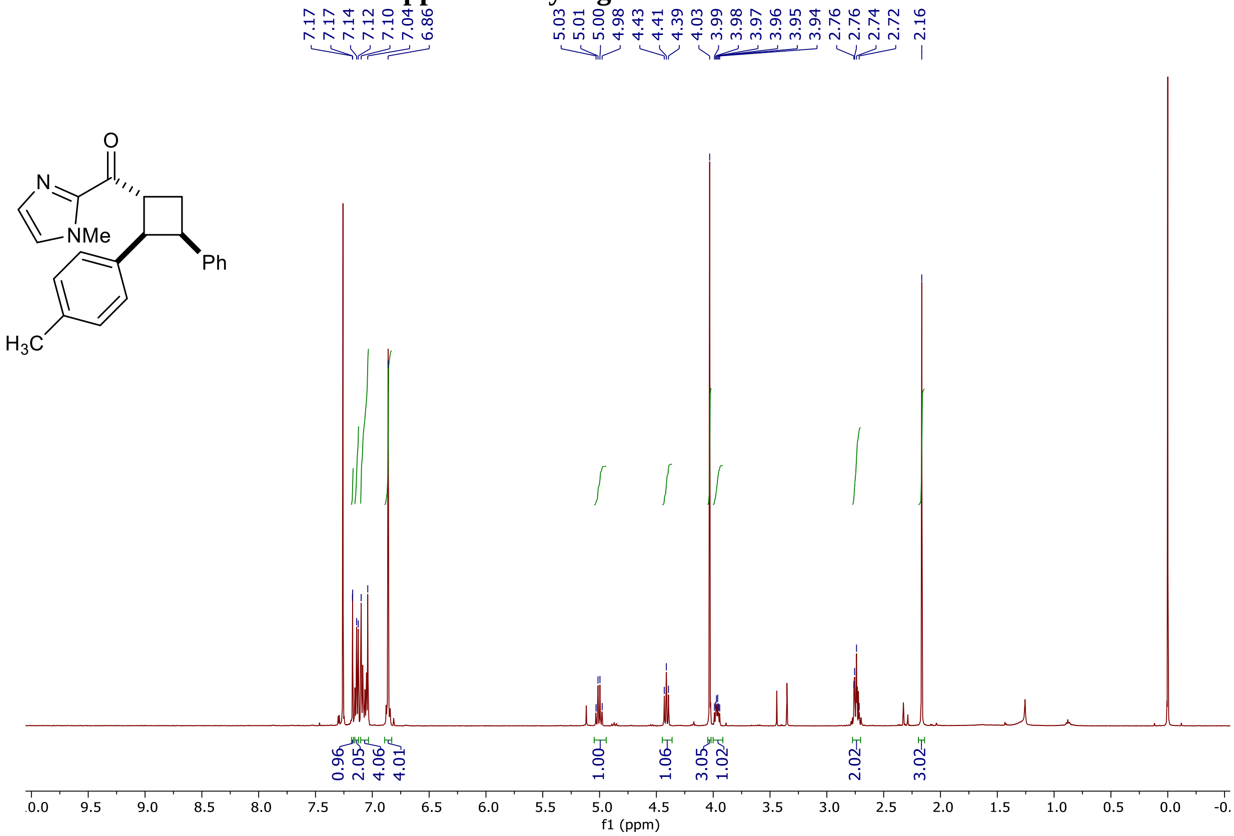

Supplementary Figure 26b. ^{13}C NMR of **9**.

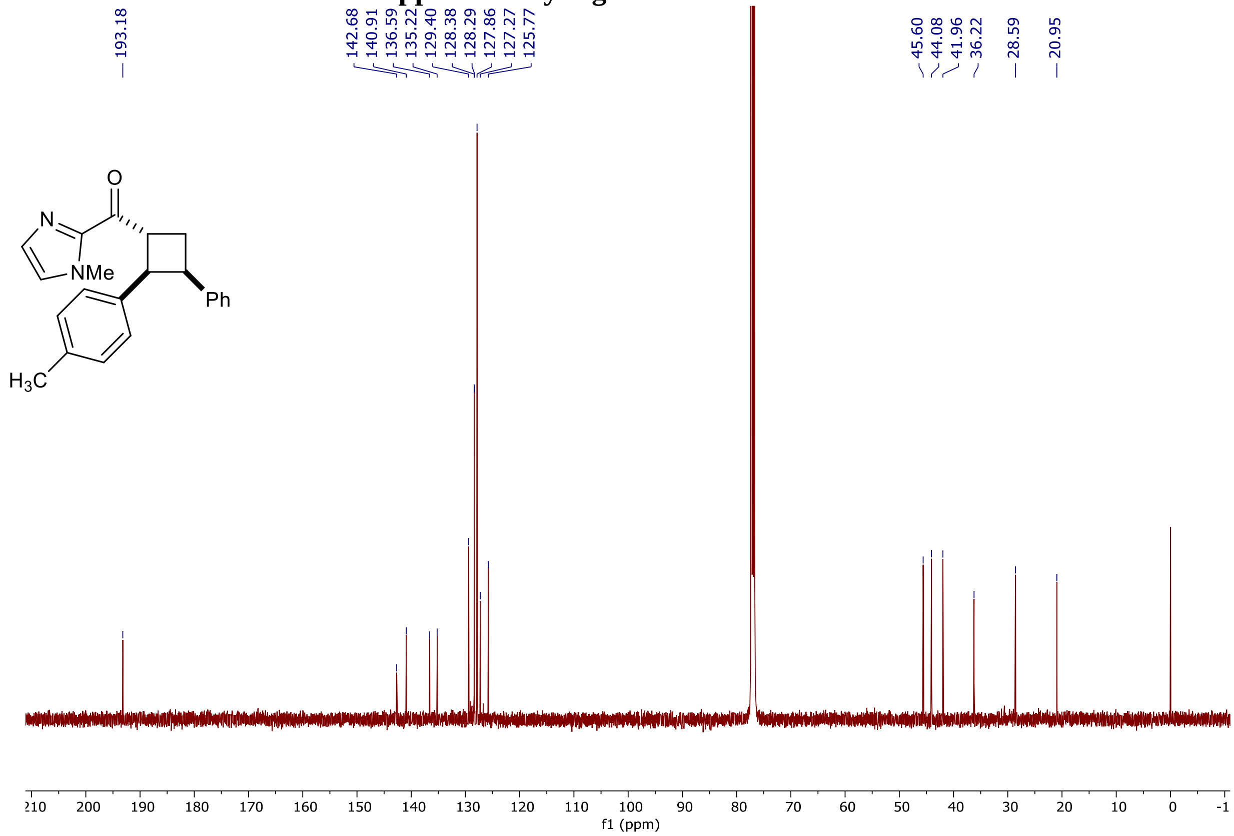

Supplementary Figure 27a. ^{1}H NMR of **10**.

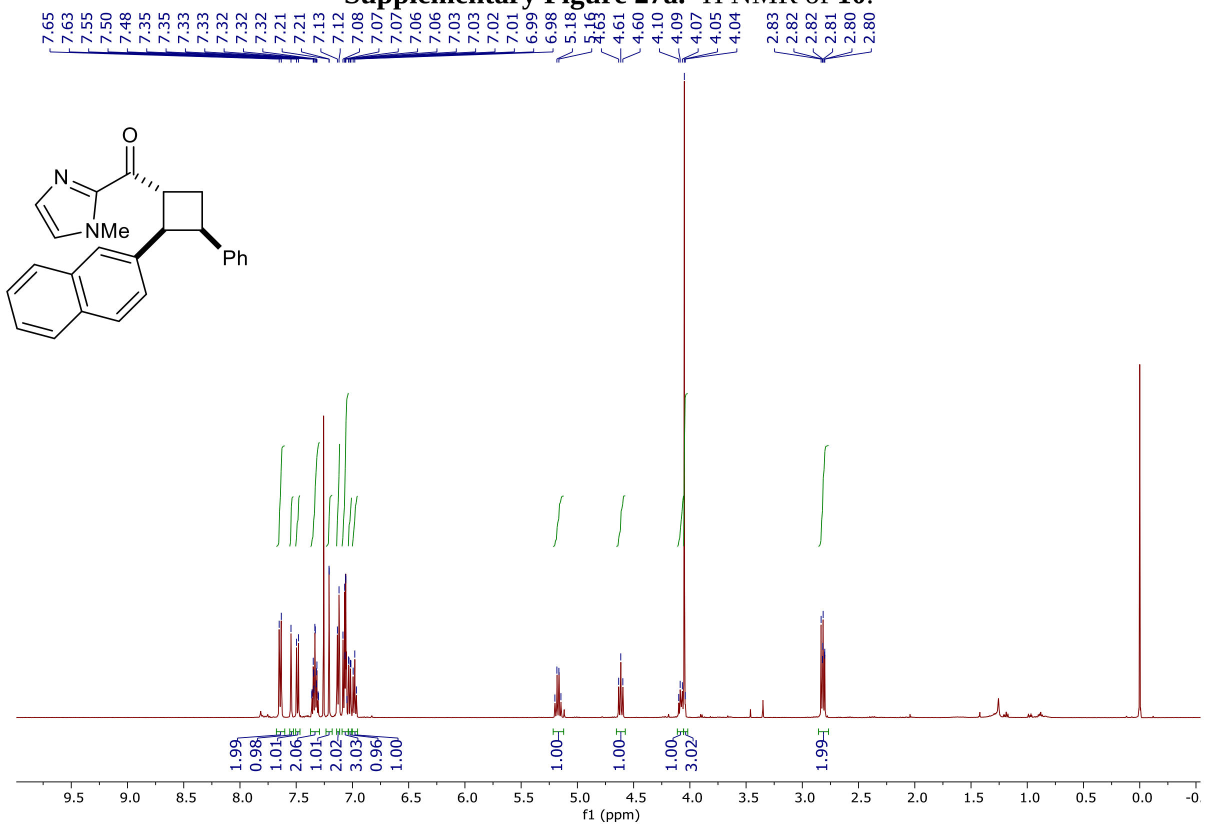

Supplementary Figure 27b. ^{13}C NMR of **10**.

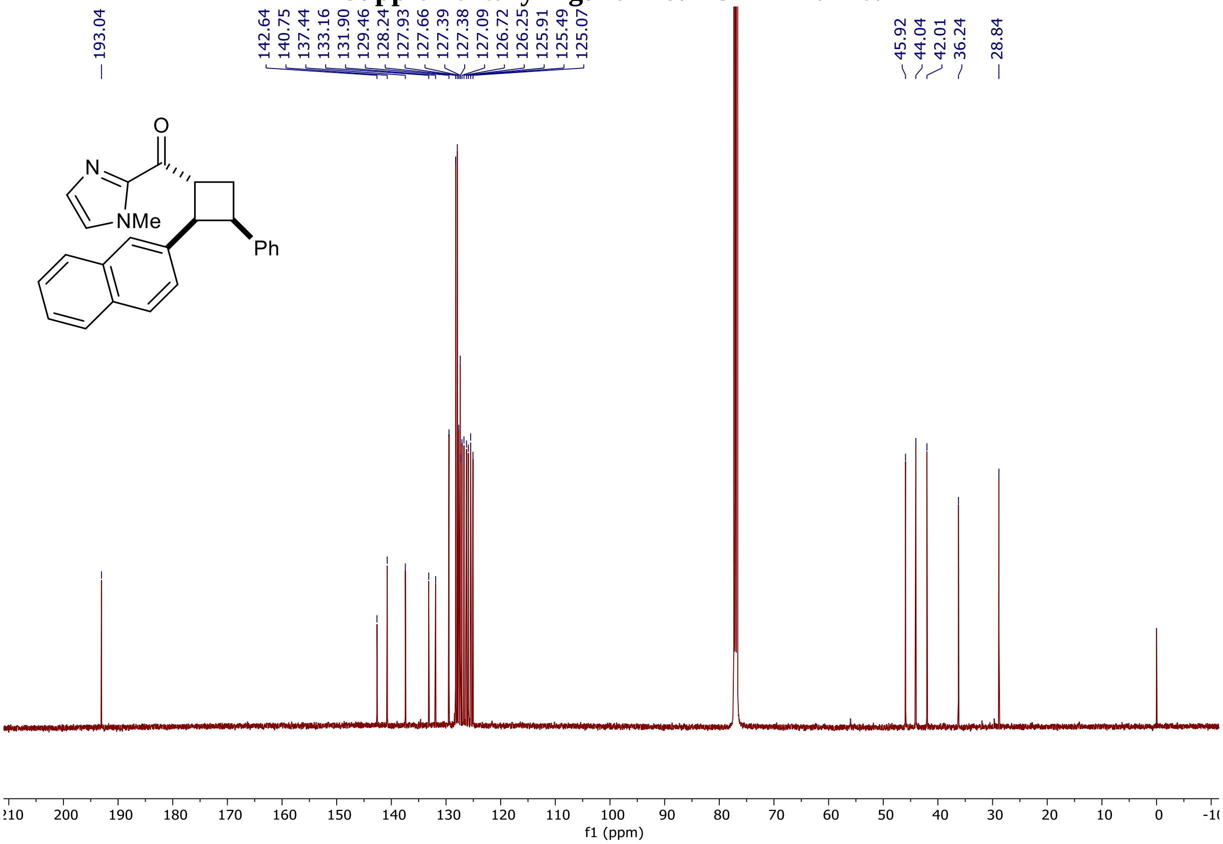

Supplementary Figure 28a. ^{1}H NMR of **12**.

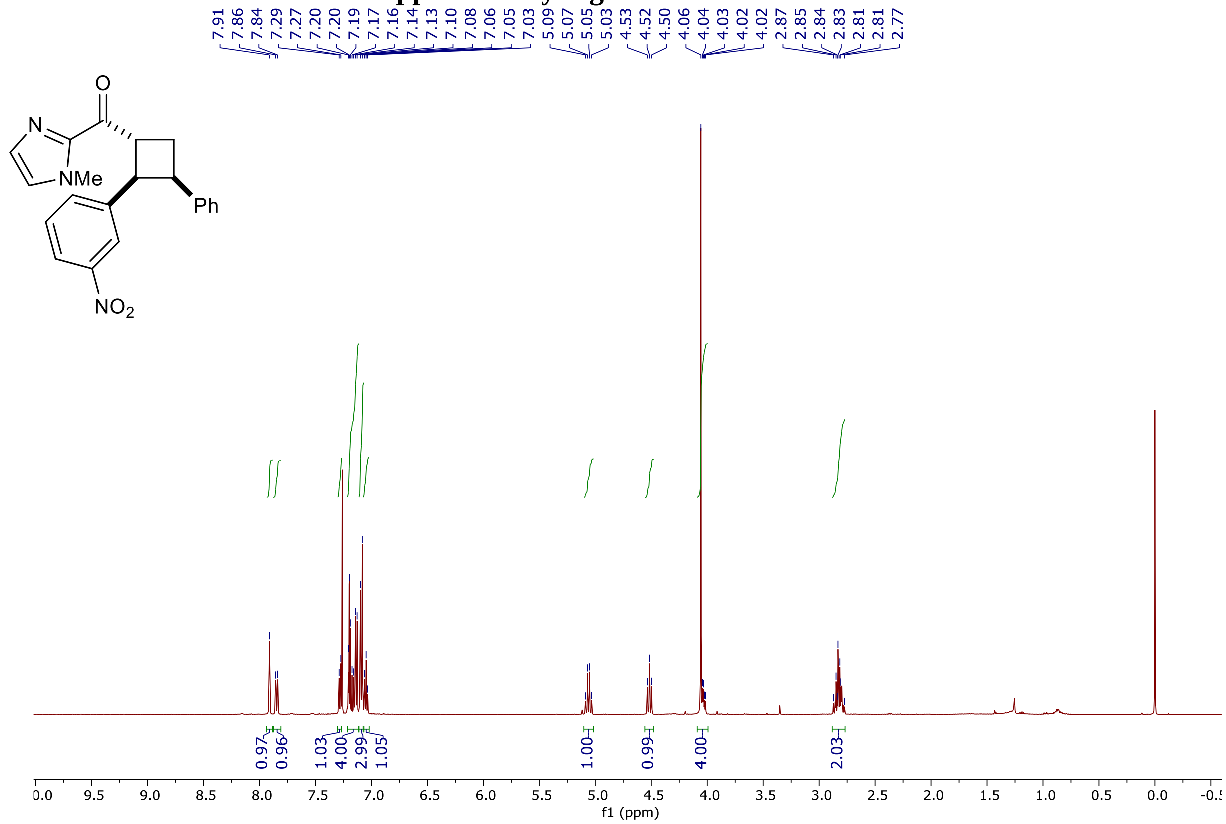

Supplementary Figure 28b. ^{13}C NMR of **12**.

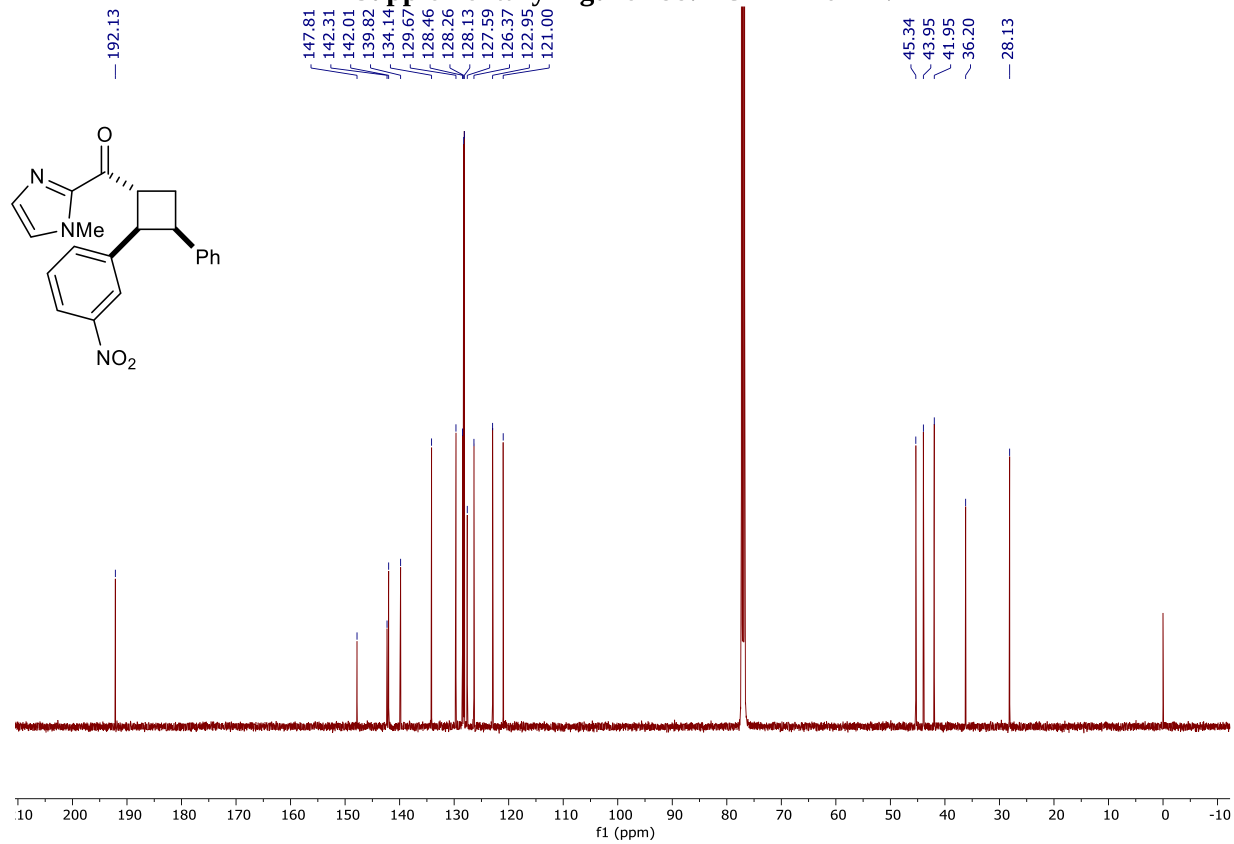

Supplementary Figure 29a. ^{1}H NMR of **13**.

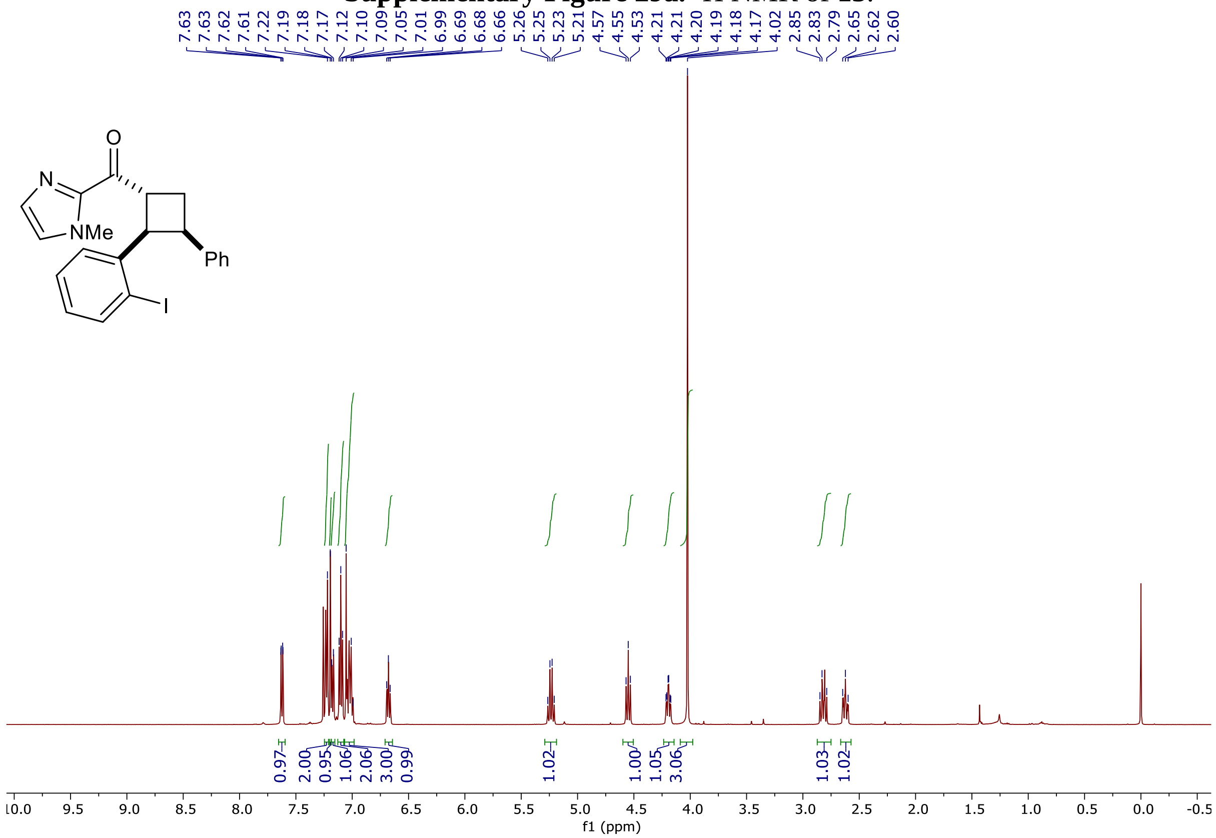

Supplementary Figure 29b. ^{13}C NMR of **13**.

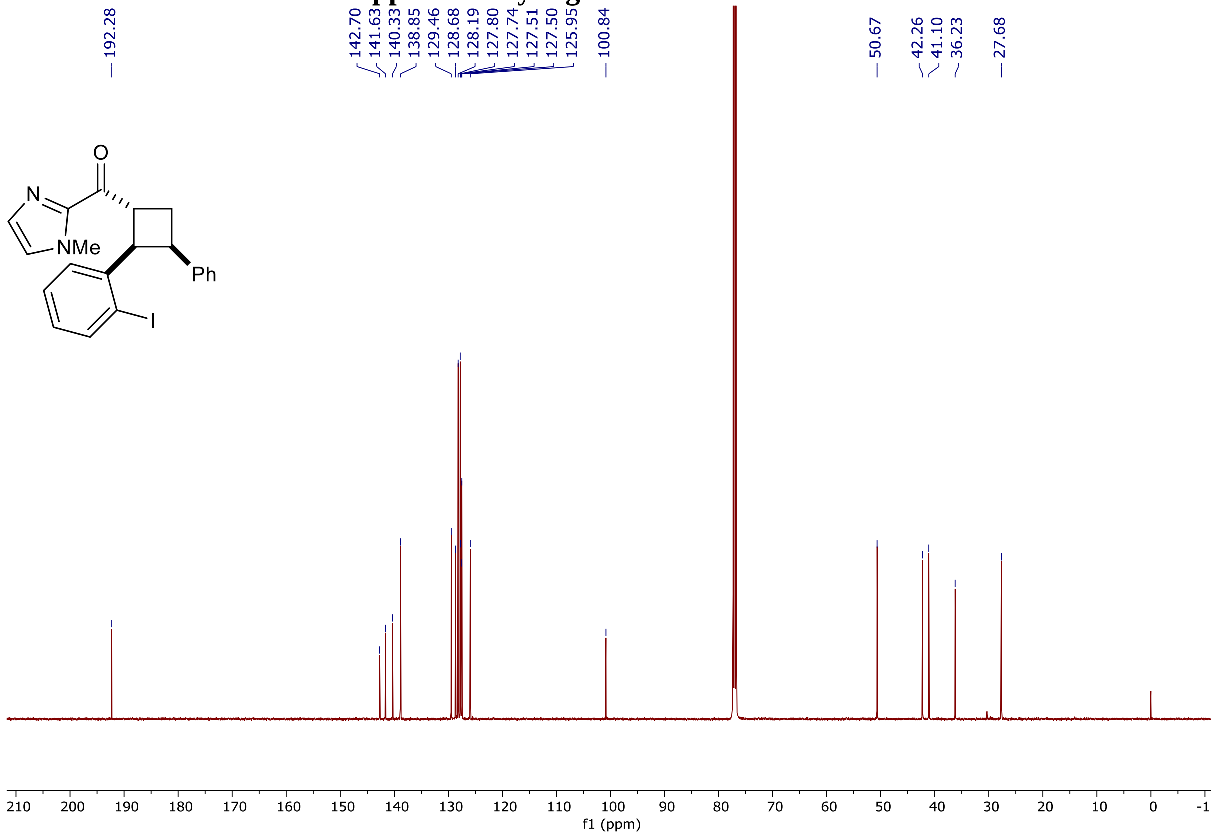

Supplementary Figure 30a. ^{1}H NMR of **15**.

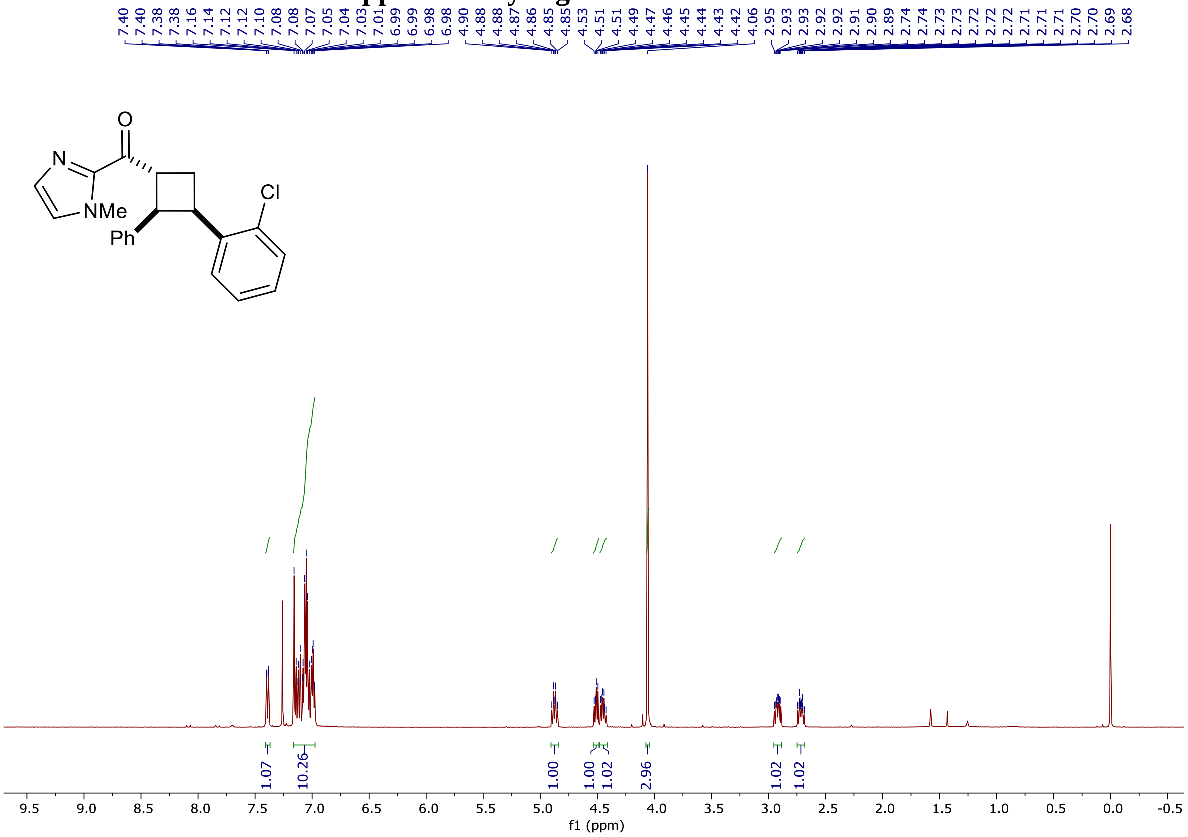

Supplementary Figure 30b. ^{13}C NMR of **15**.

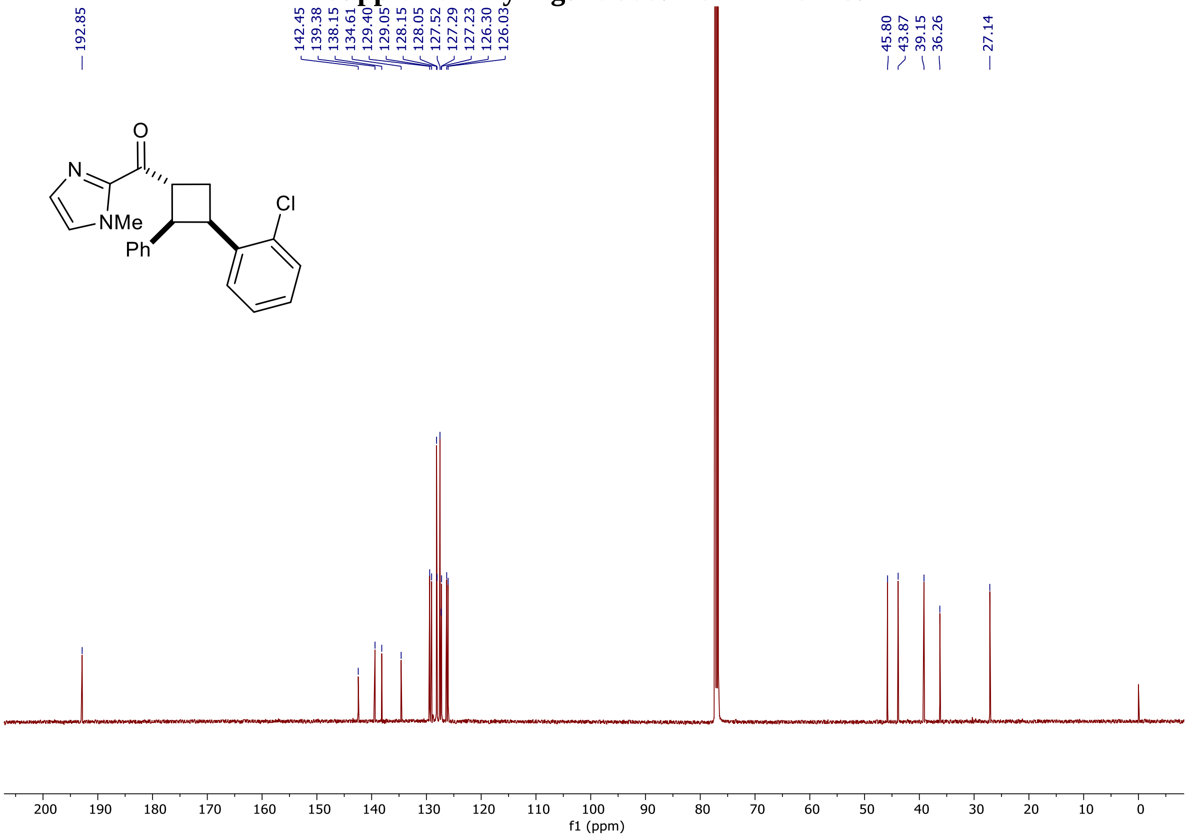

Supplementary Figure 31a. ^{1}H NMR of **16**.

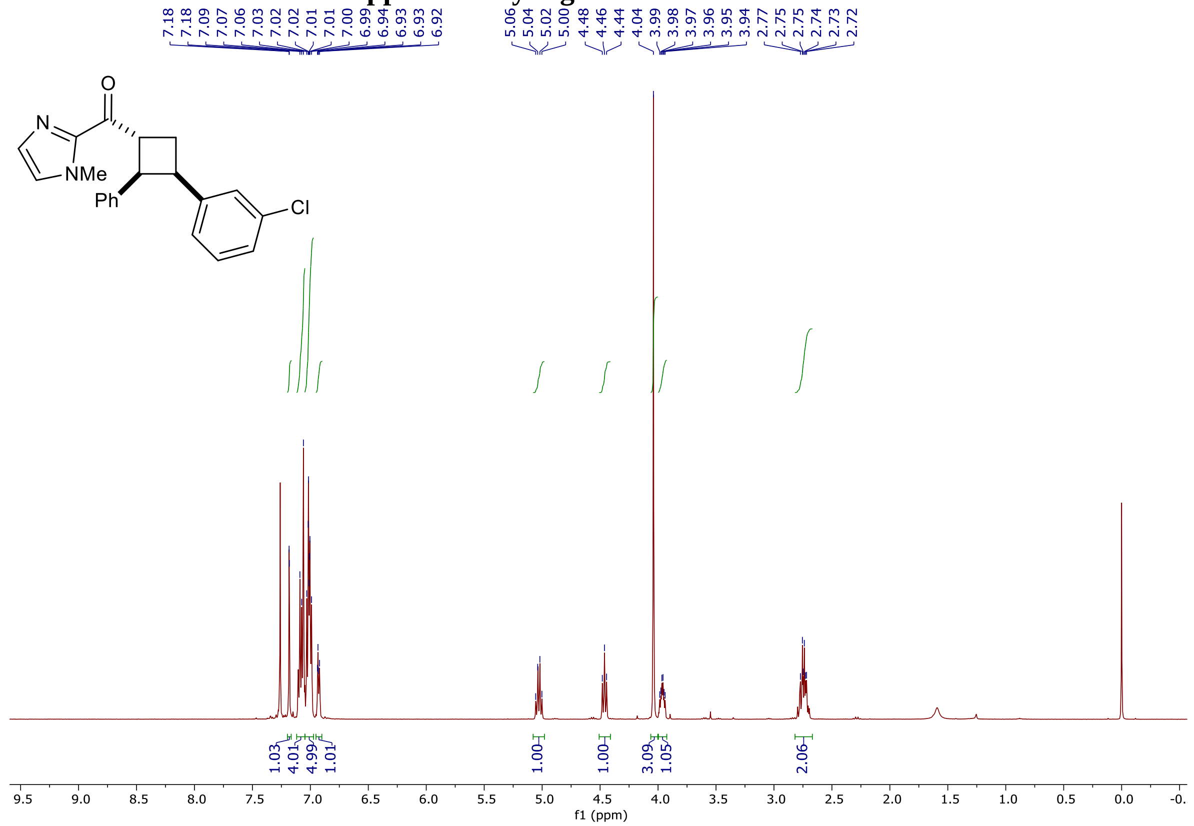

Supplementary Figure 31b. ^{13}C NMR of **16**.

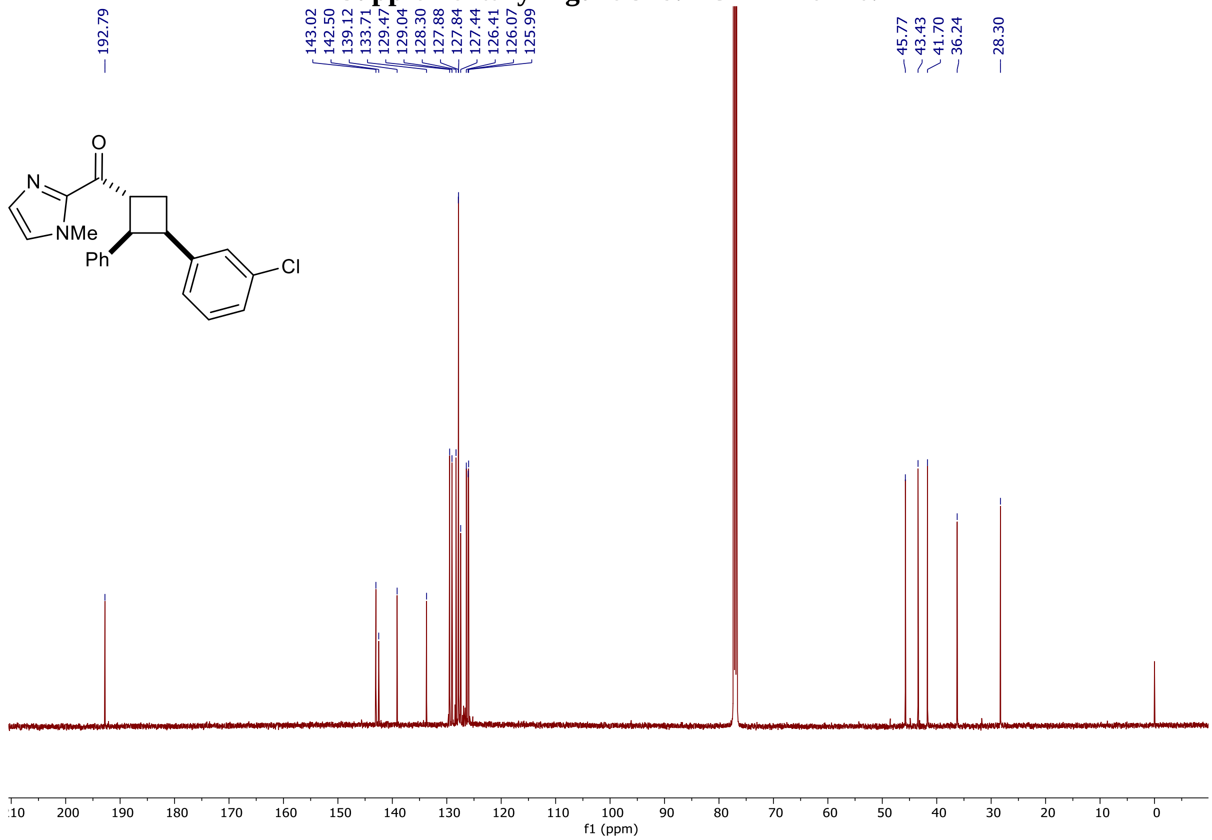

Supplementary Figure 32a. ^{1}H NMR of **17**.

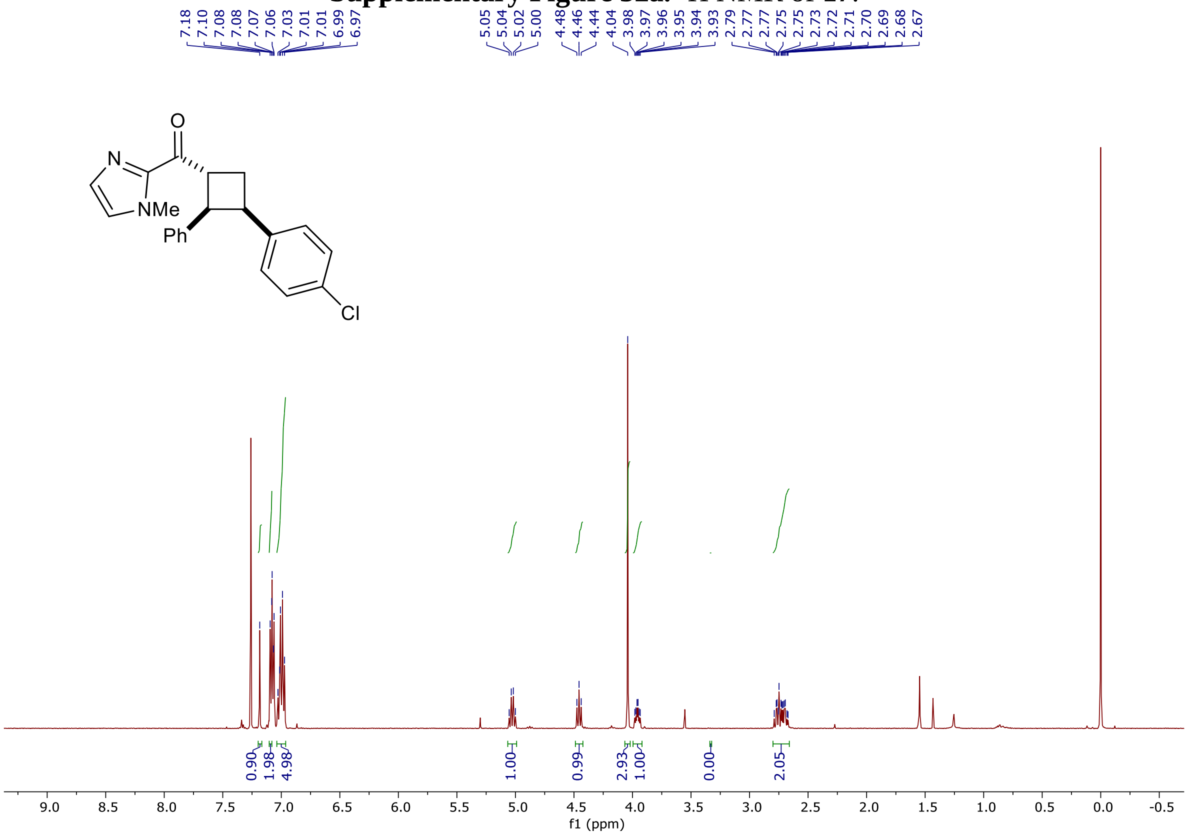

Supplementary Figure 32b. ^{13}C NMR of **17**.

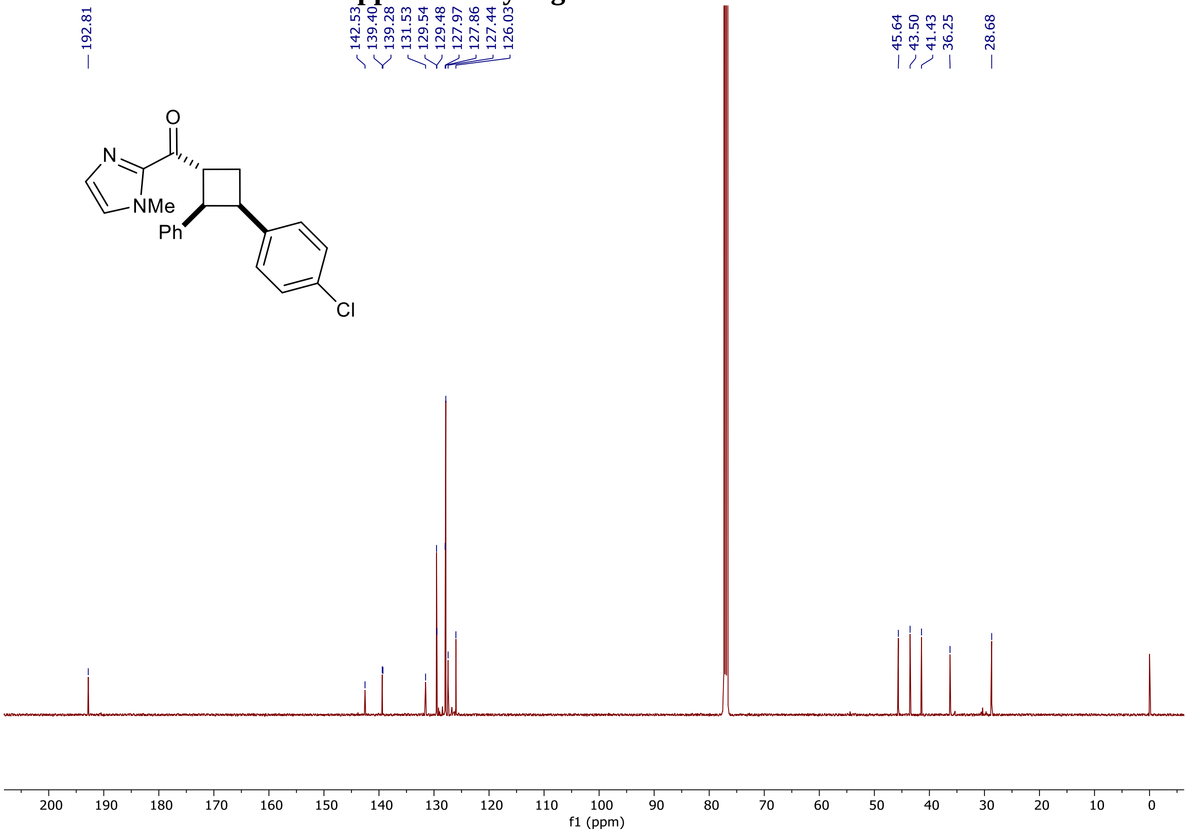

Supplementary Figure 33a. ^{1}H NMR of **18**.

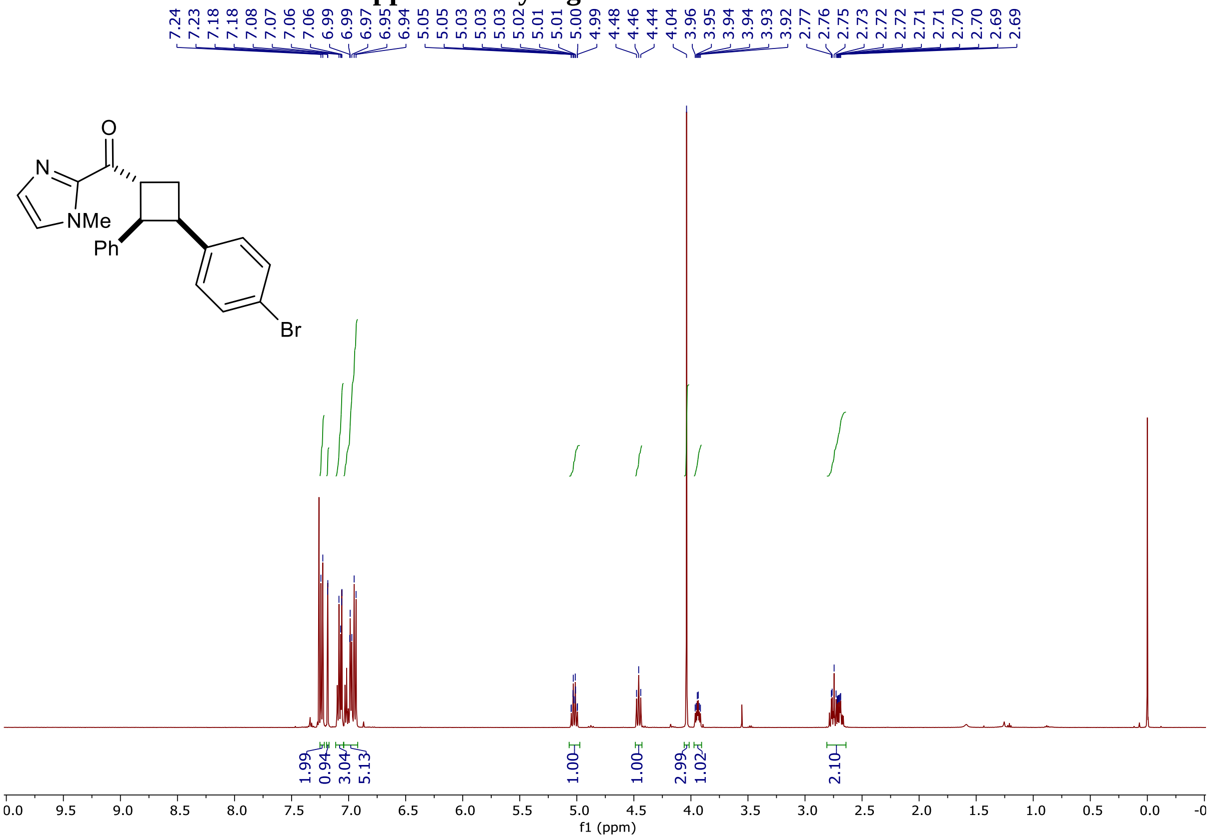

Supplementary Figure 33b. ^{13}C NMR of **18**.

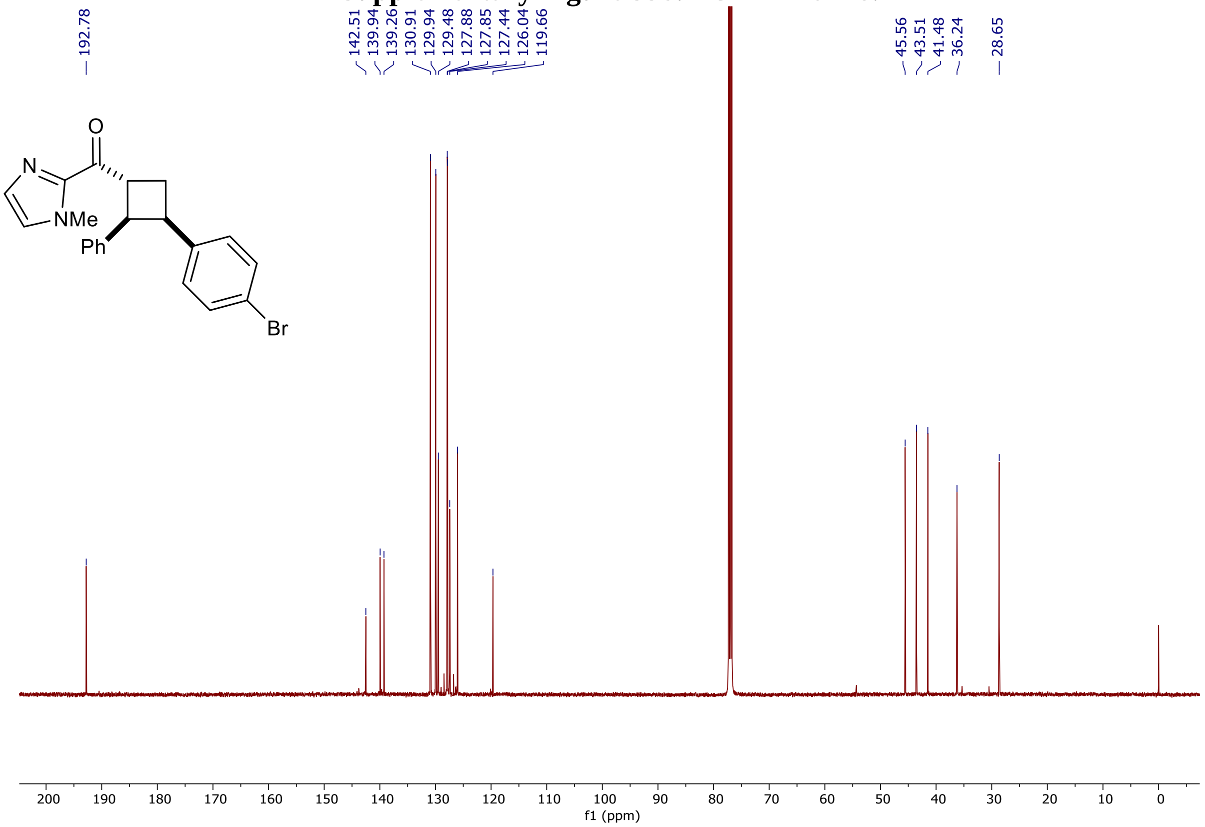

Supplementary Figure 34a. ^{1}H NMR of 19.

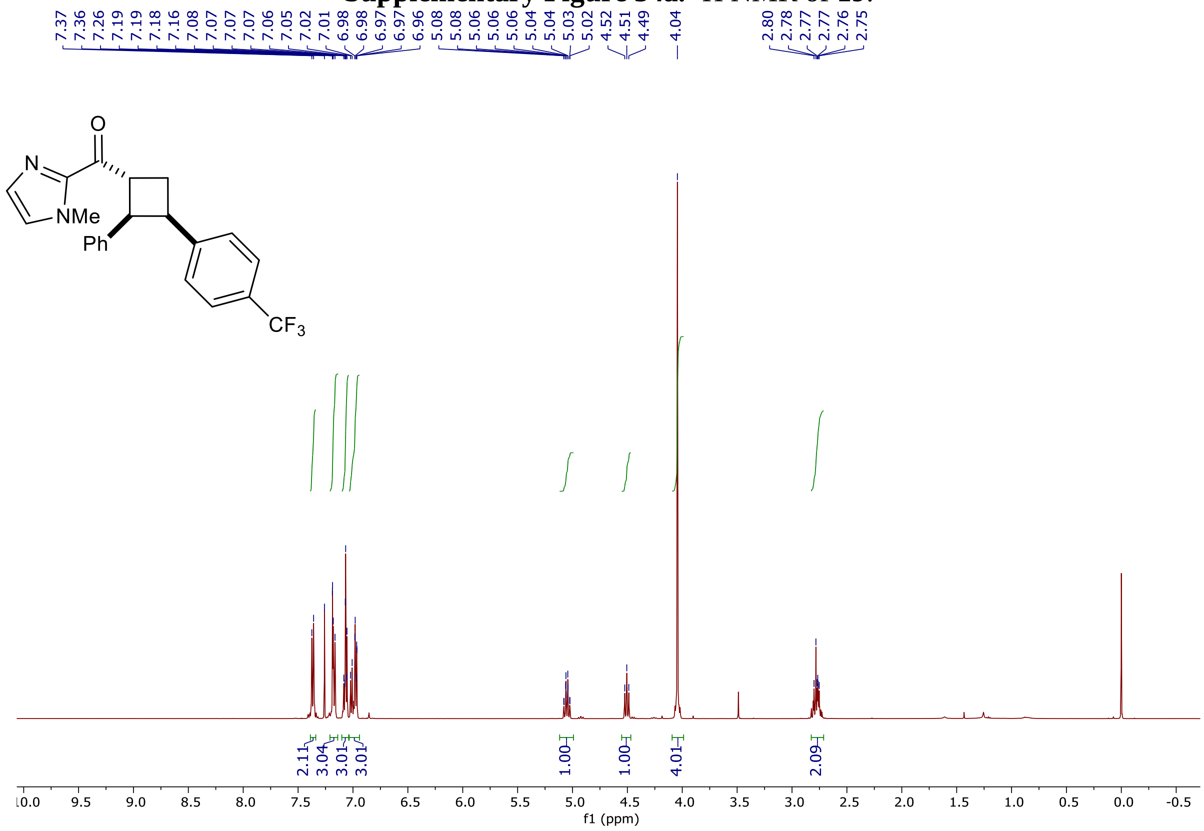

Supplementary Figure 34b. ^{13}C NMR of 19.

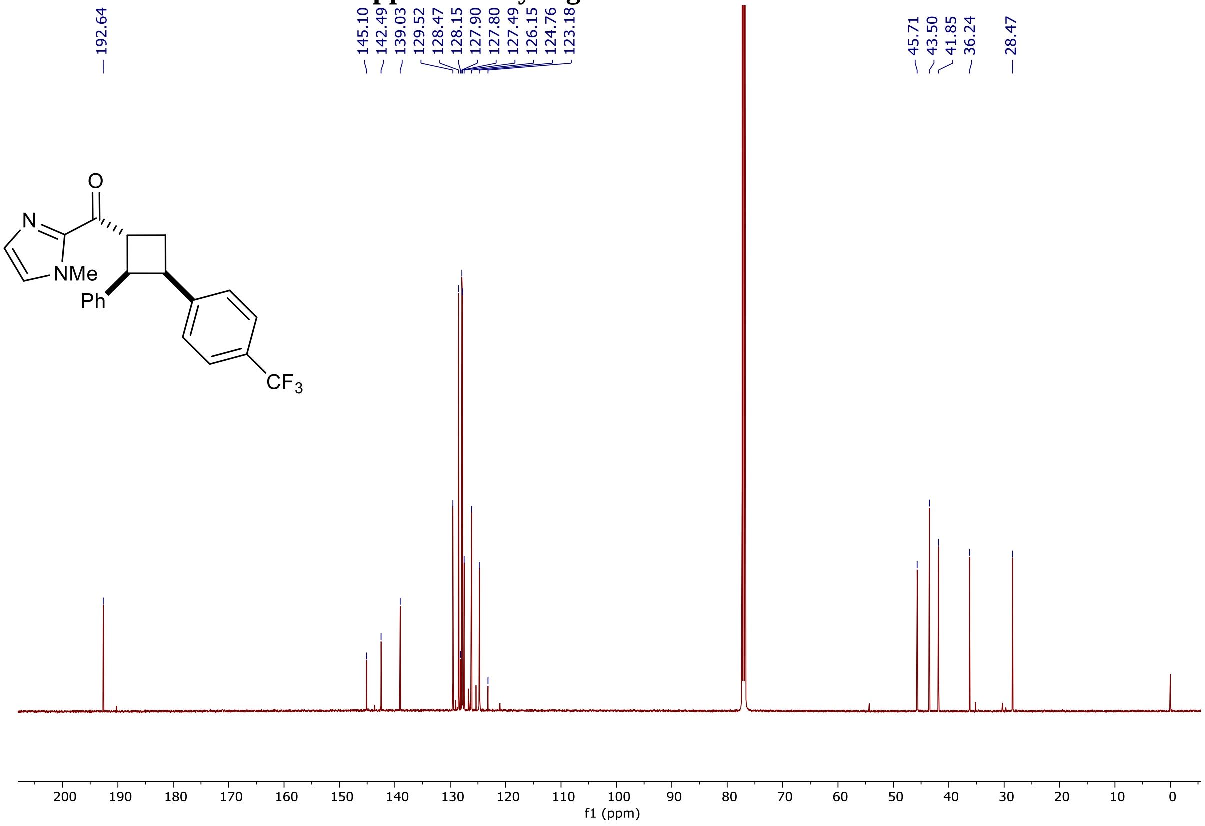

Supplementary Figure 35a. ^{1}H NMR of **20**.

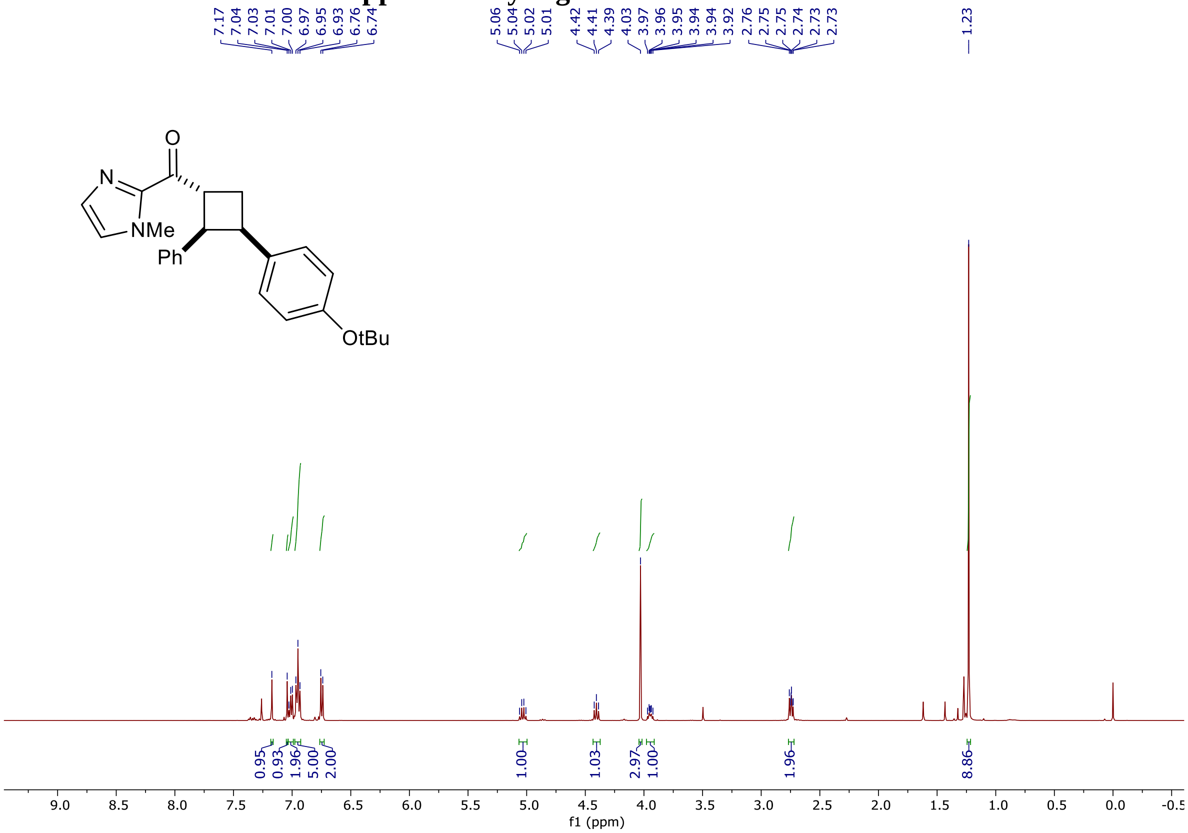

Supplementary Figure 35b. ^{13}C NMR of **20**.

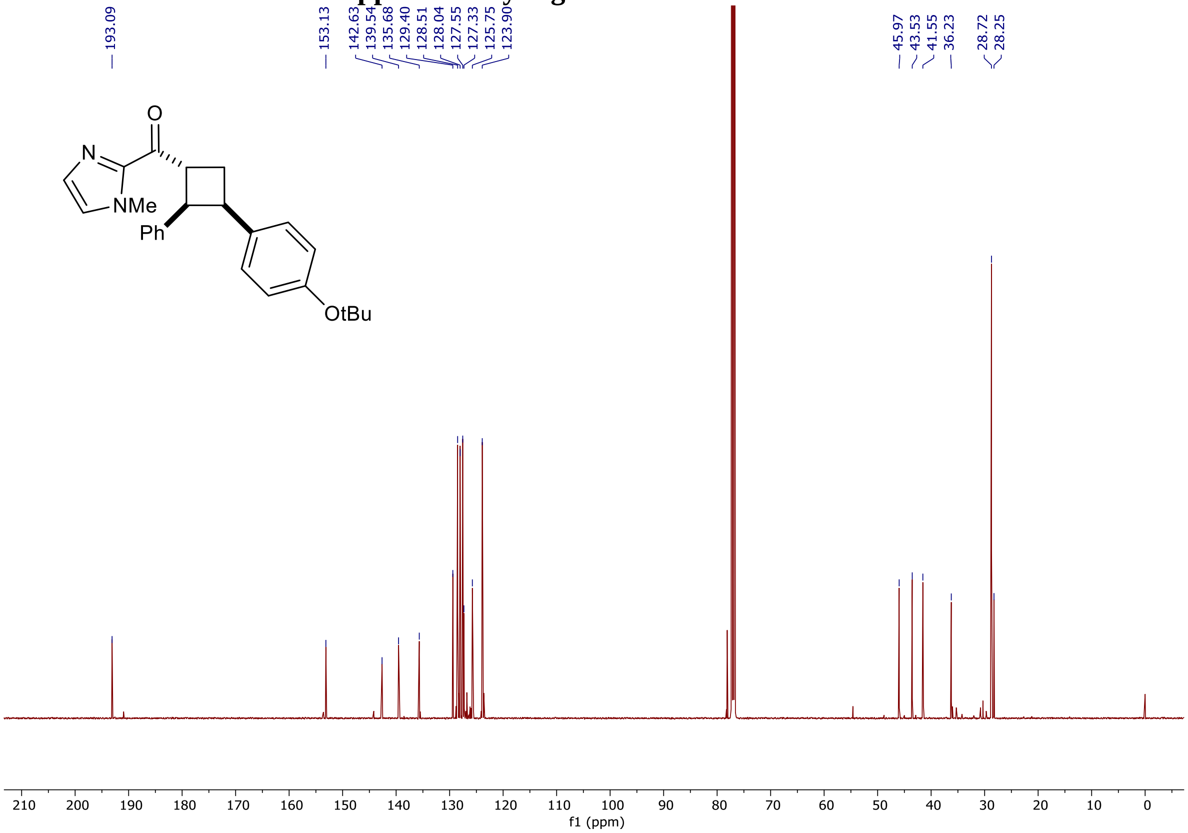

Supplementary Figure 36a. ^{1}H NMR of **21**.

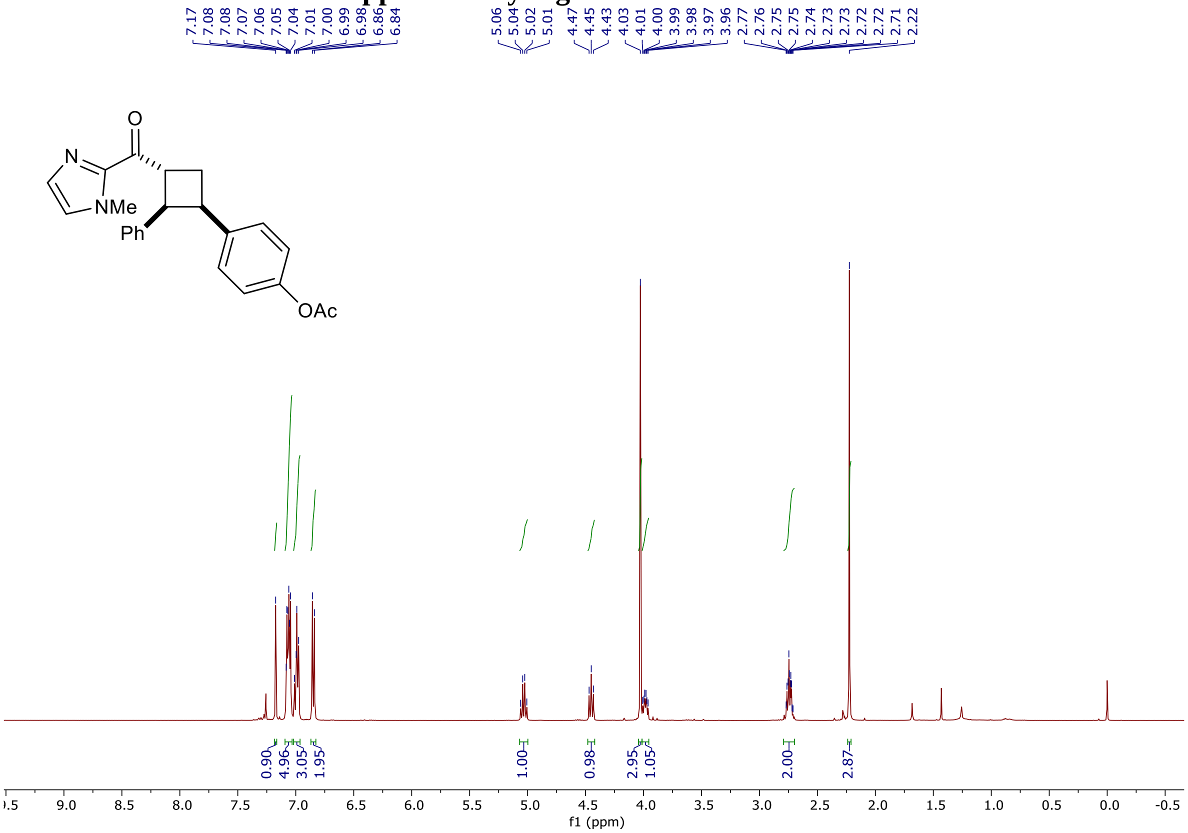

Supplementary Figure 36b. ^{13}C NMR of **21**.

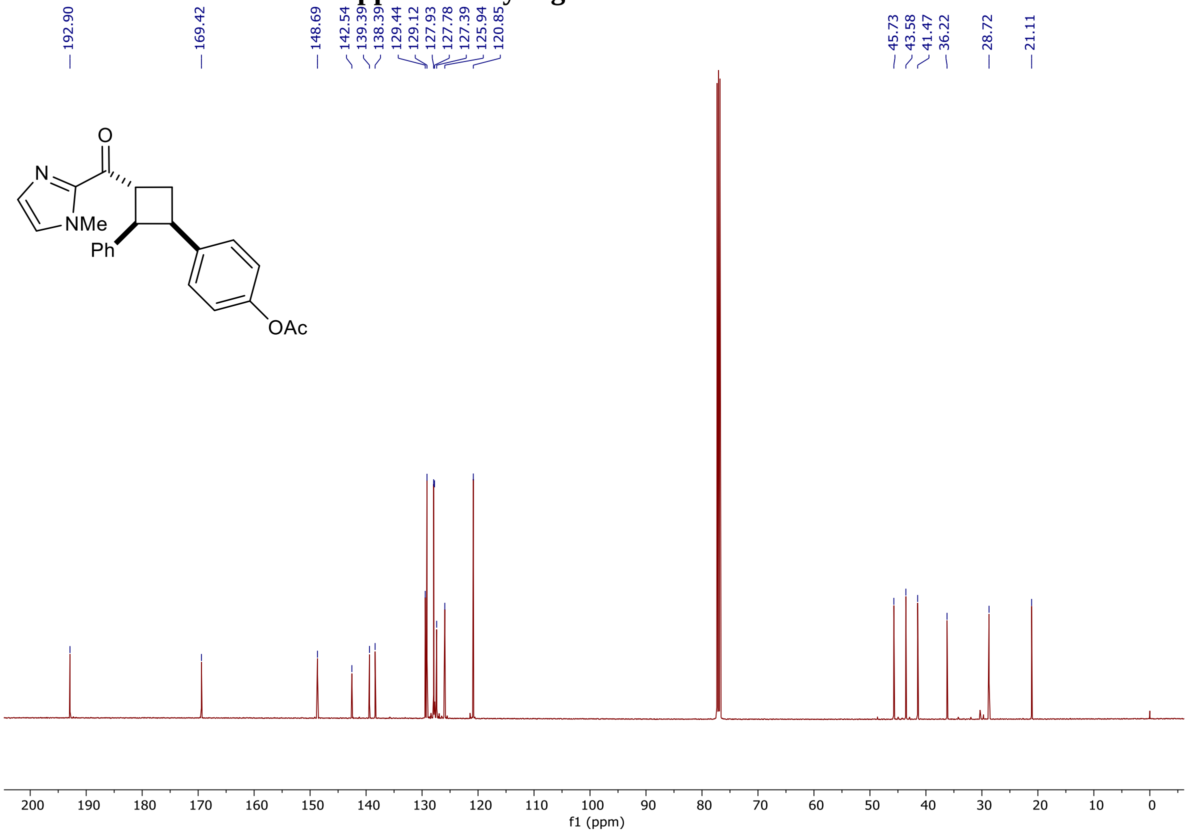

Supplementary Figure 37a. ^{1}H NMR of **22**.

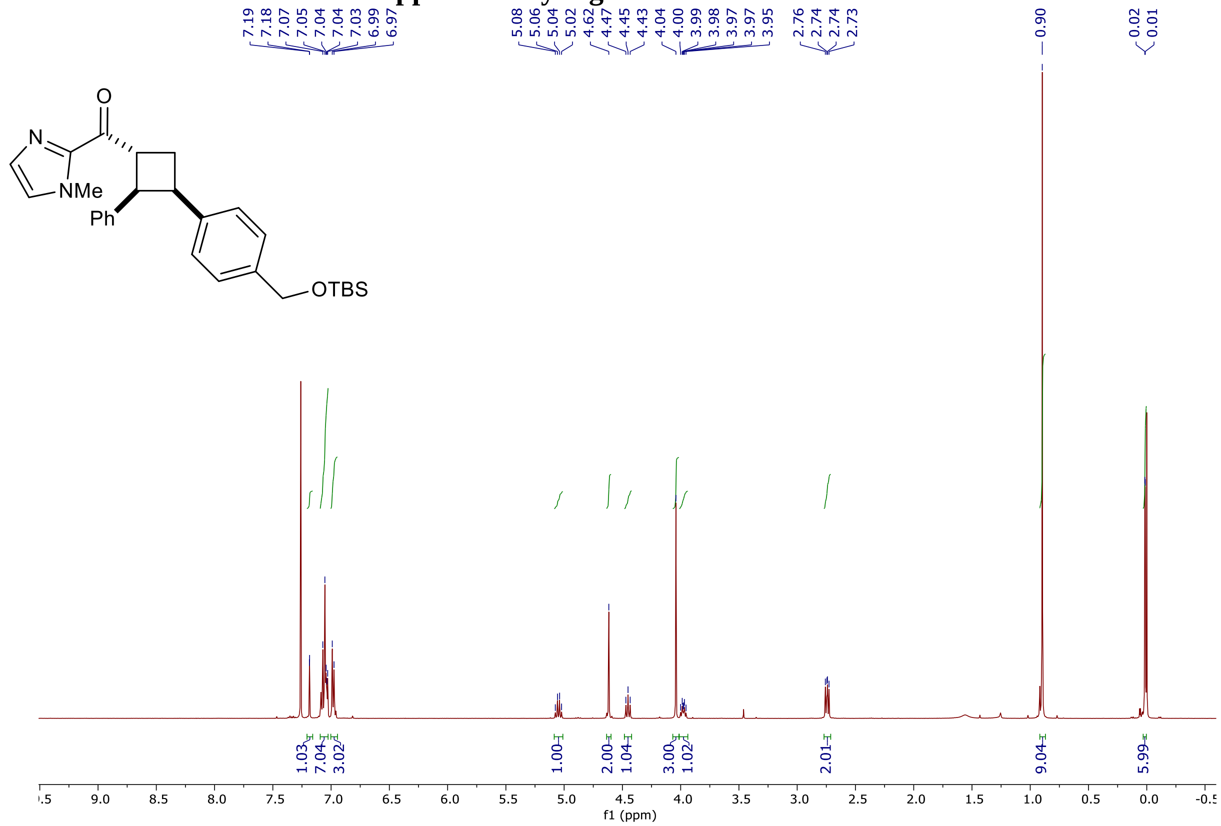

Supplementary Figure 37b. ^{13}C NMR of **22**.

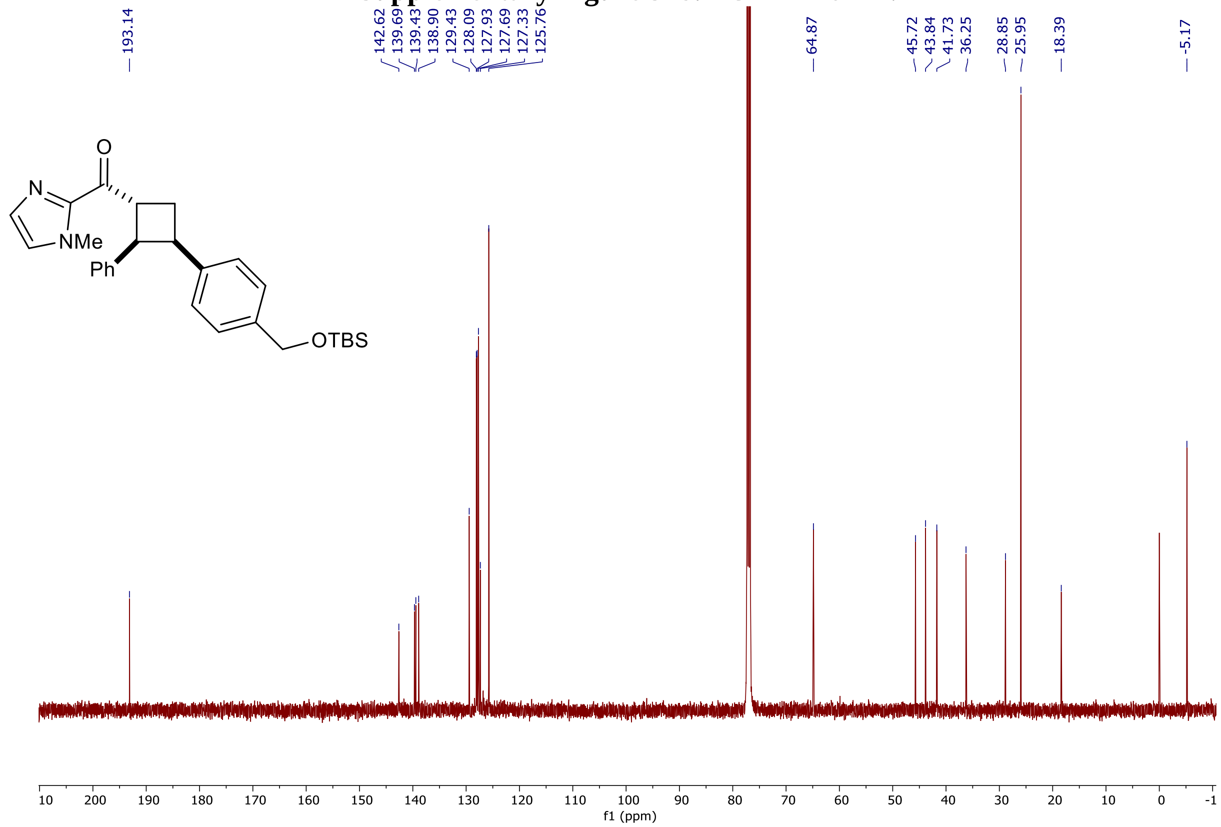

Supplementary Figure 38a. ^{1}H NMR of **23**.

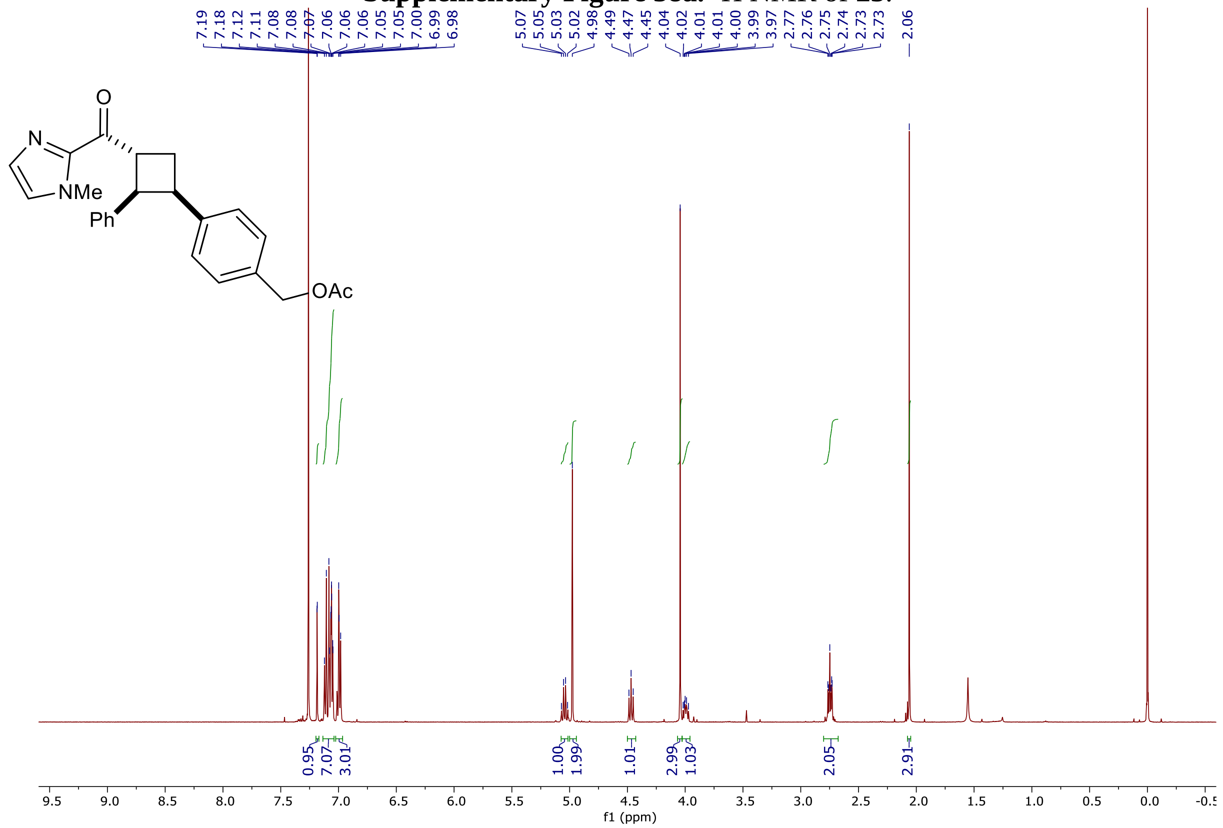

Supplementary Figure 38b. ^{13}C NMR of **23**.

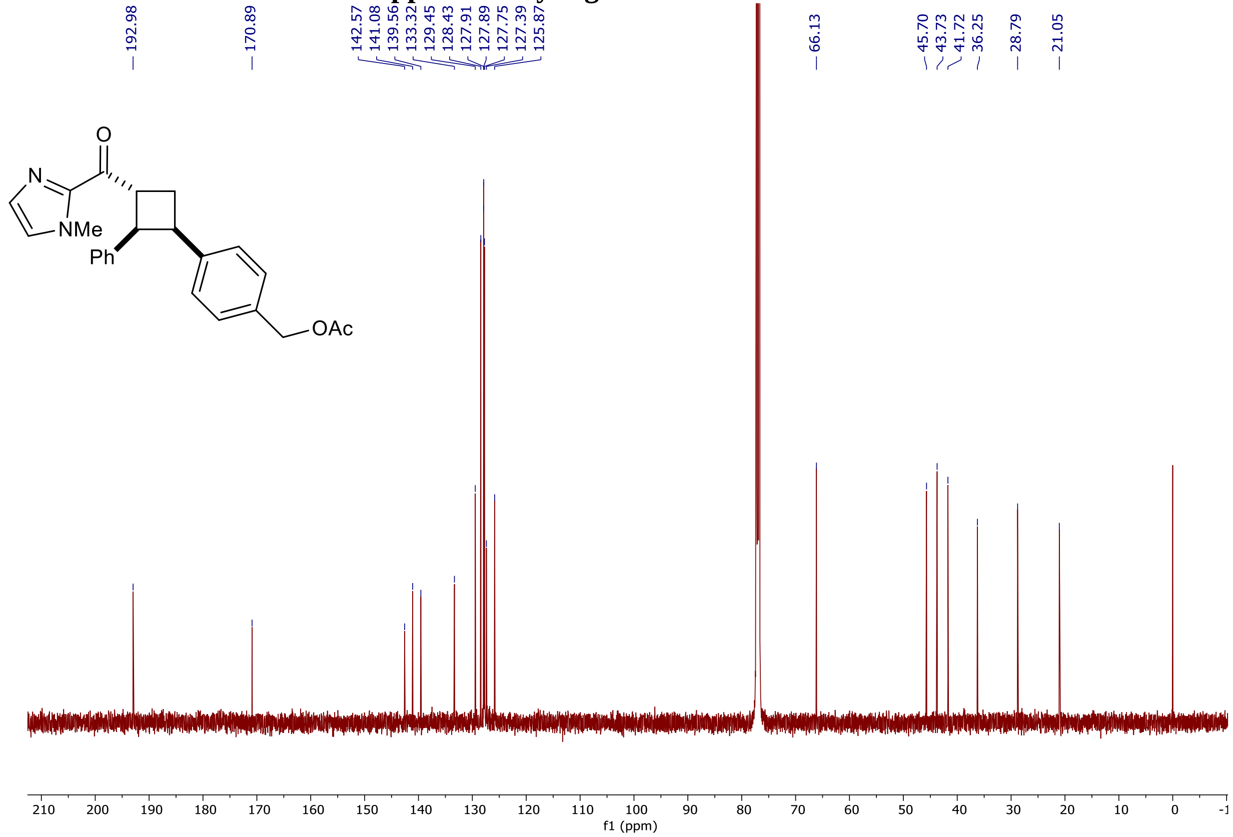

Supplementary Figure 39a. ^{1}H NMR of **24**.

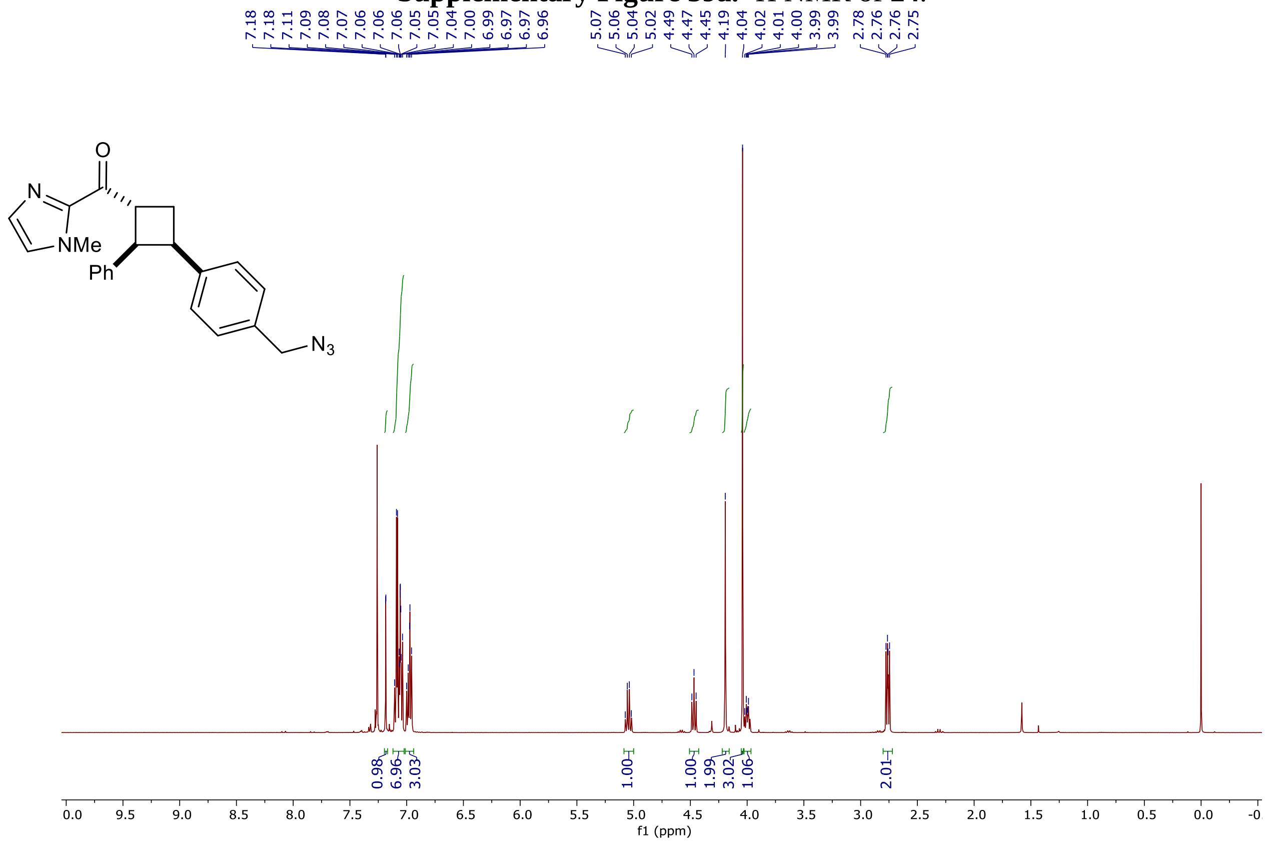

Supplementary Figure 39b. ^{13}C NMR of **24**.

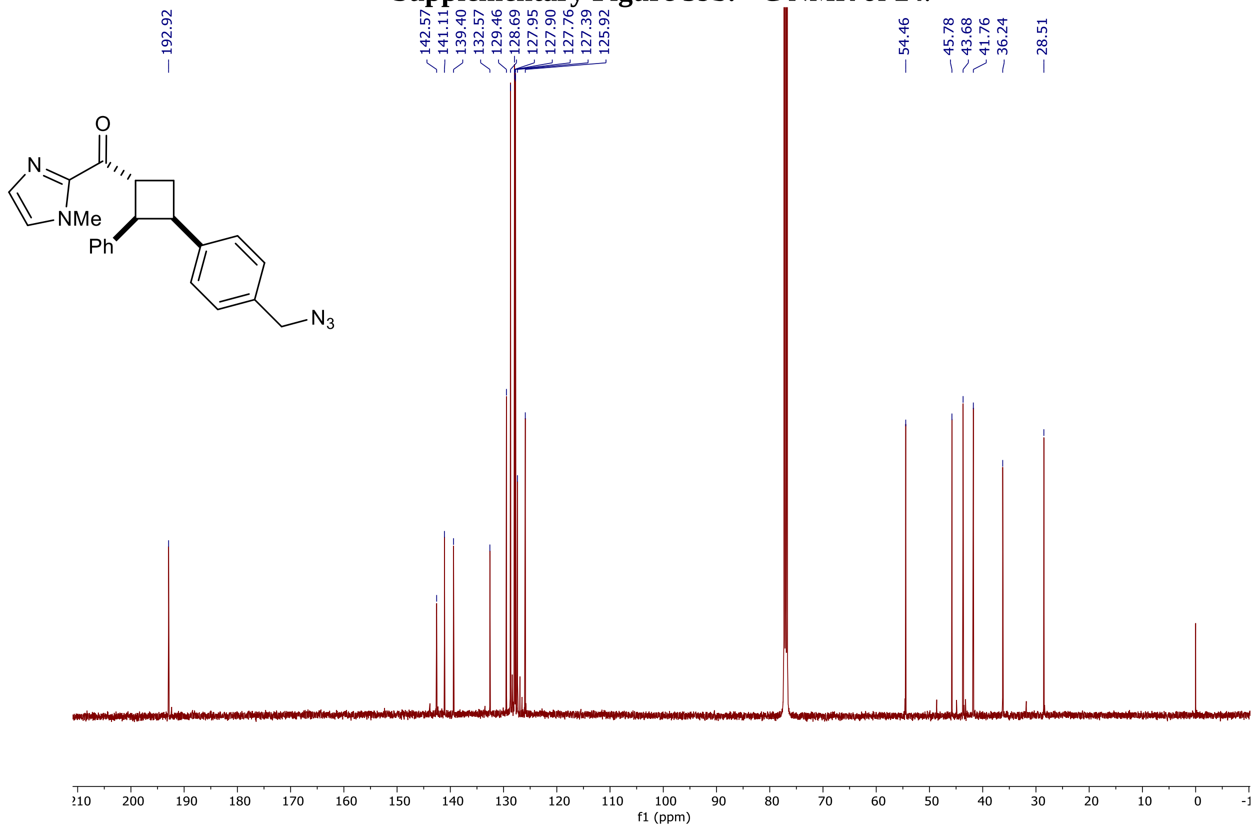

Supplementary Figure 40a. ^{1}H NMR of **25**.

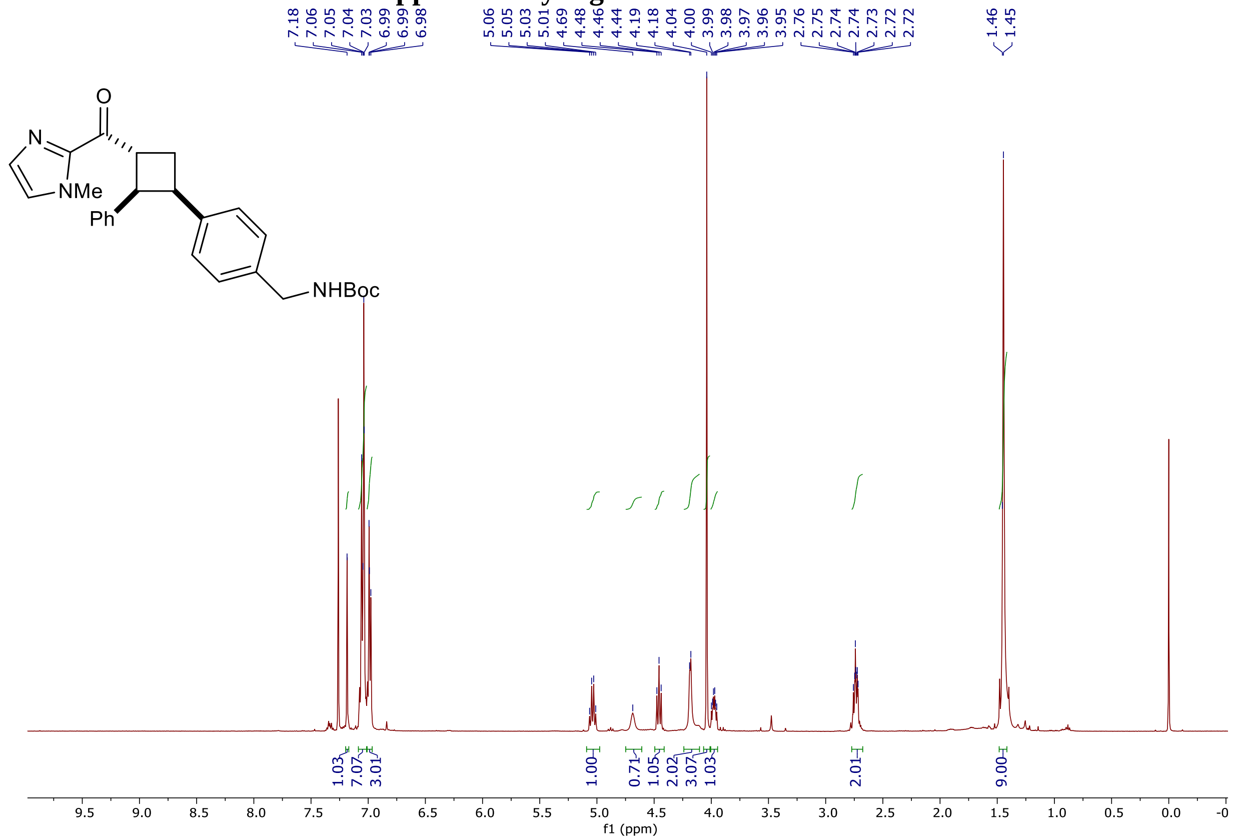

Supplementary Figure 40b. ^{13}C NMR of **25**.

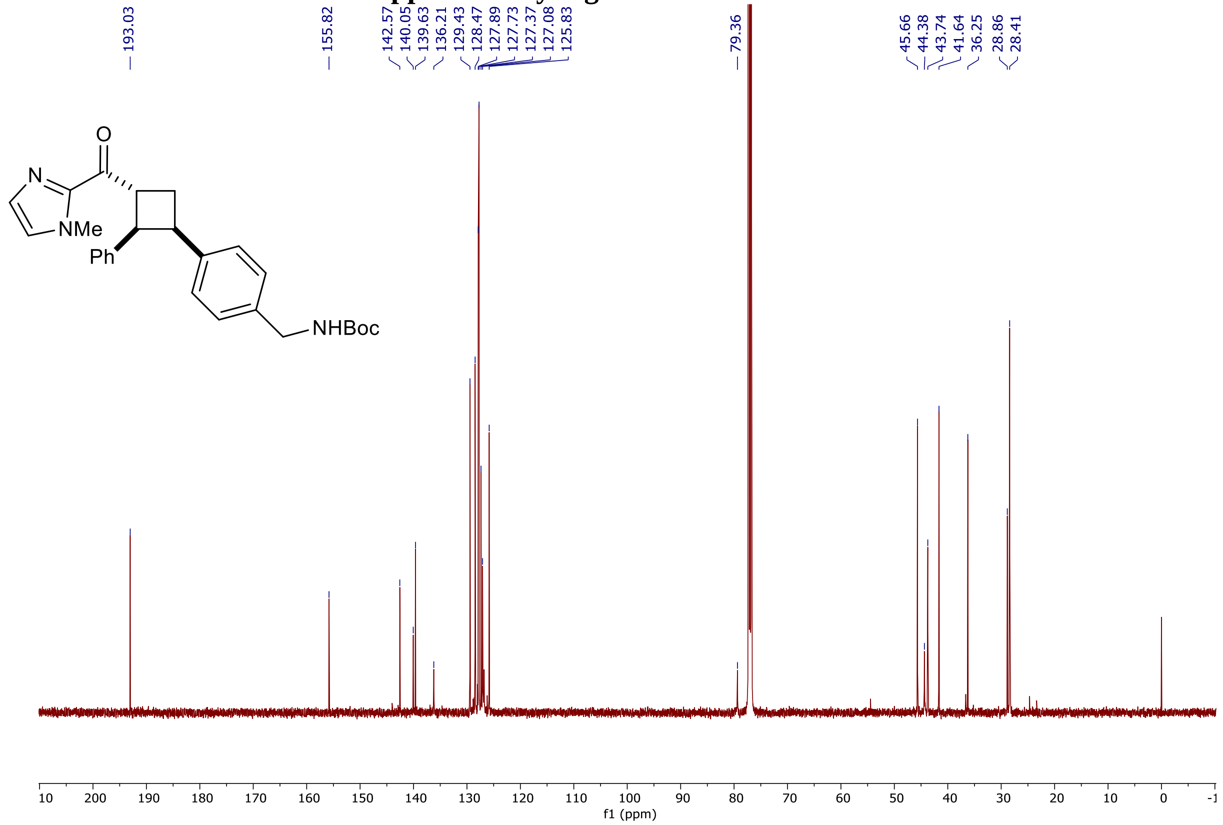

Supplementary Figure 41a. ^{1}H NMR of **27**.

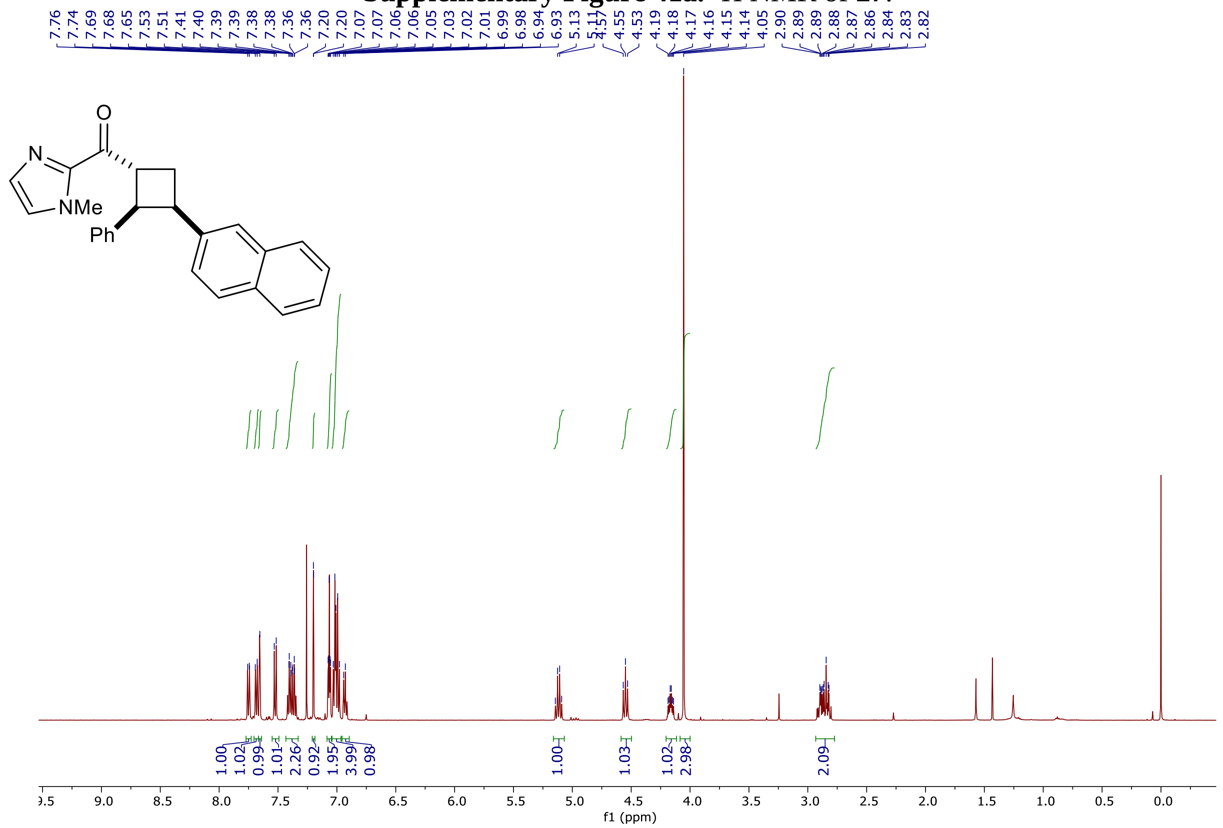

Supplementary Figure 41b. ^{13}C NMR of **27**.

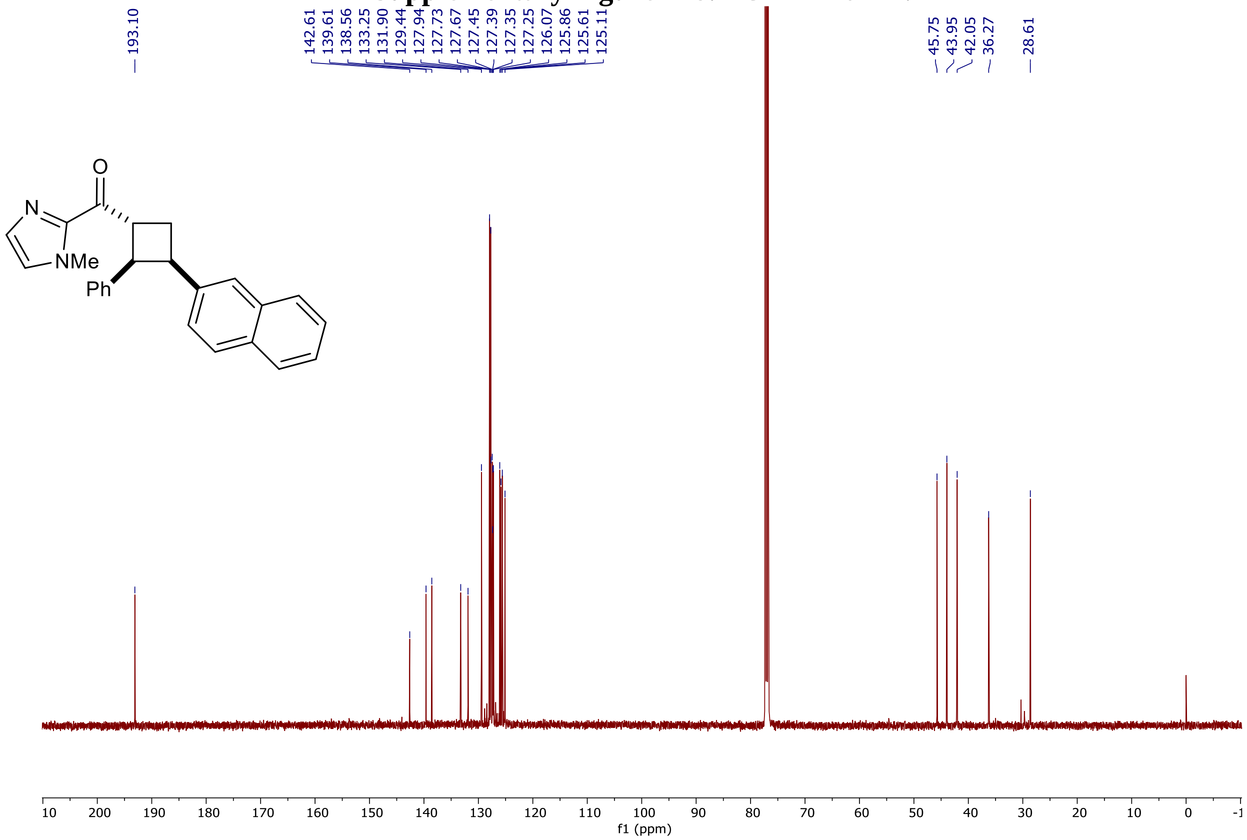

Supplementary Figure 42a. ^{1}H NMR of **28**.

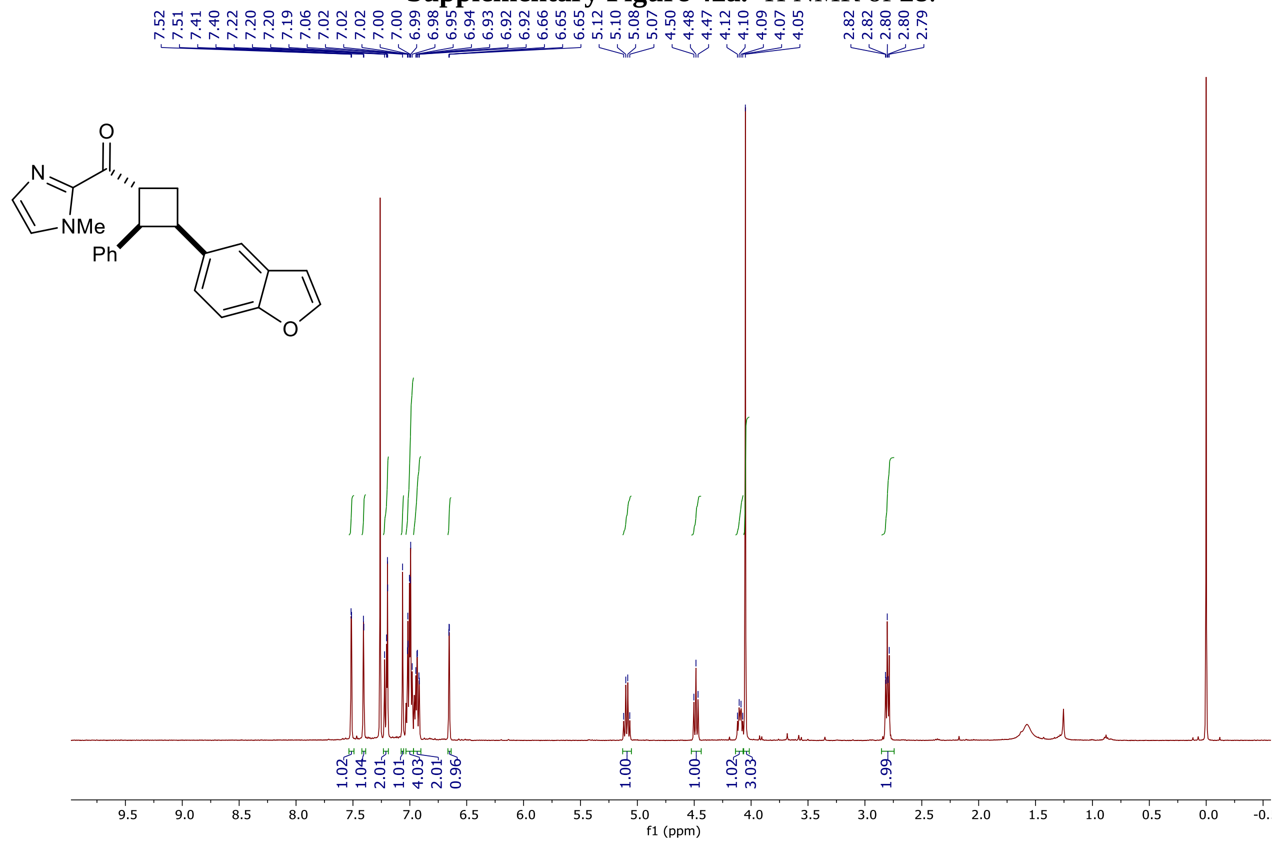

Supplementary Figure 42b. ^{13}C NMR of **28**.

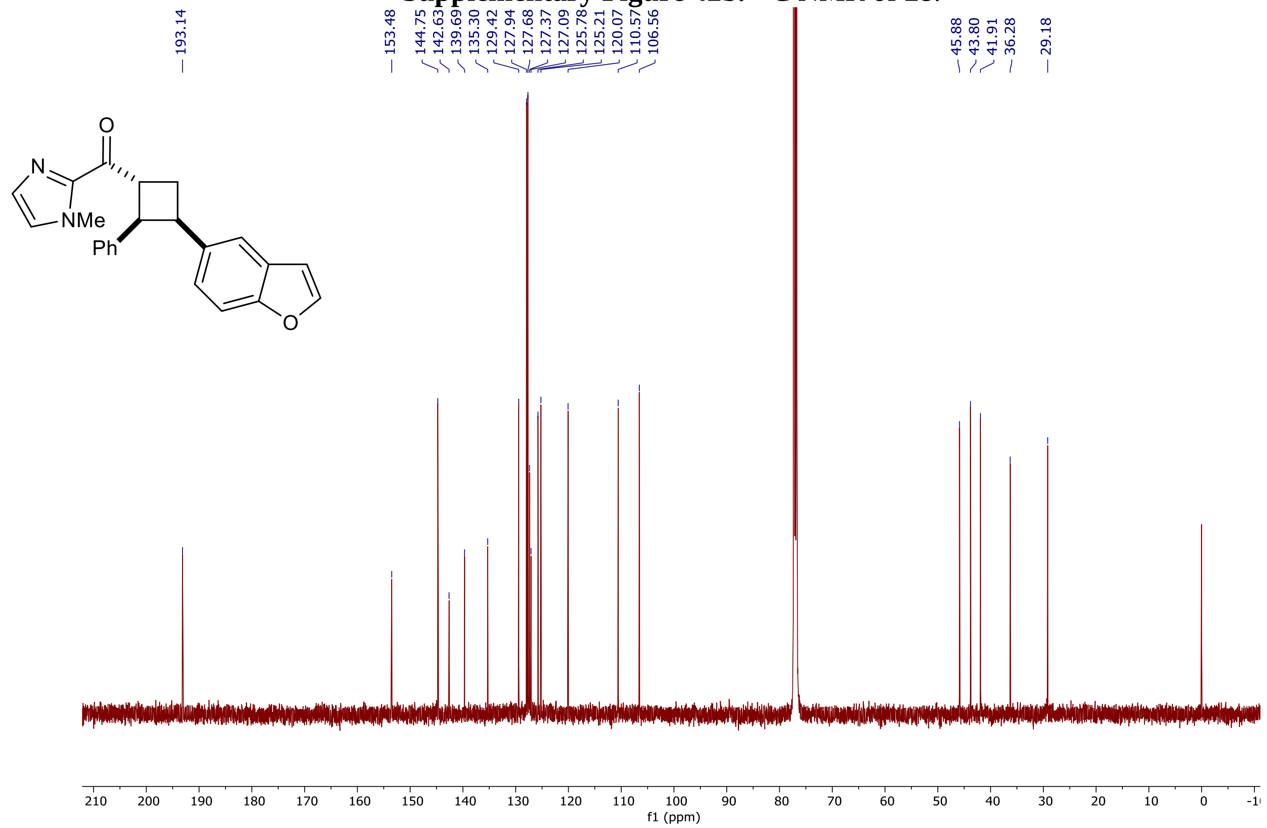

Supplementary Figure 43a. ^{1}H NMR of **29**.

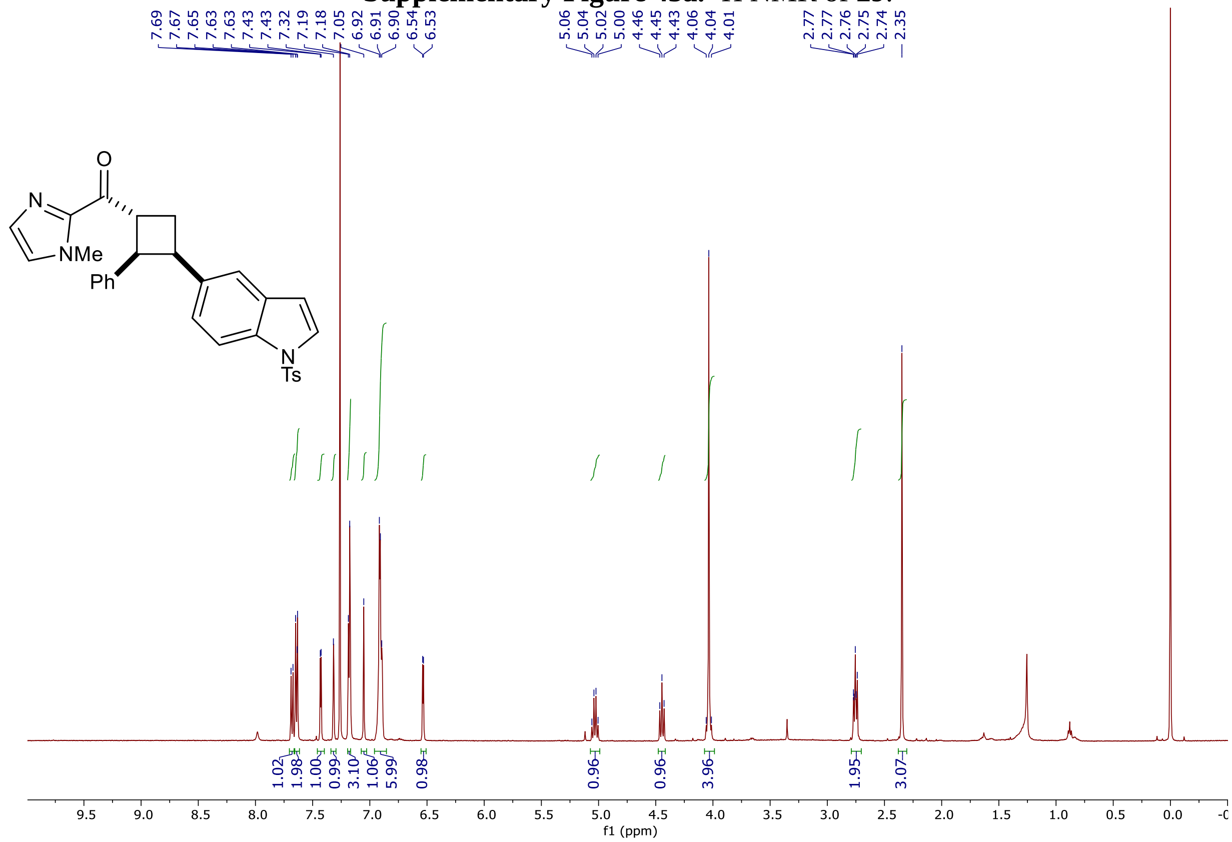

Supplementary Figure 43b. ^{13}C NMR of **29**.

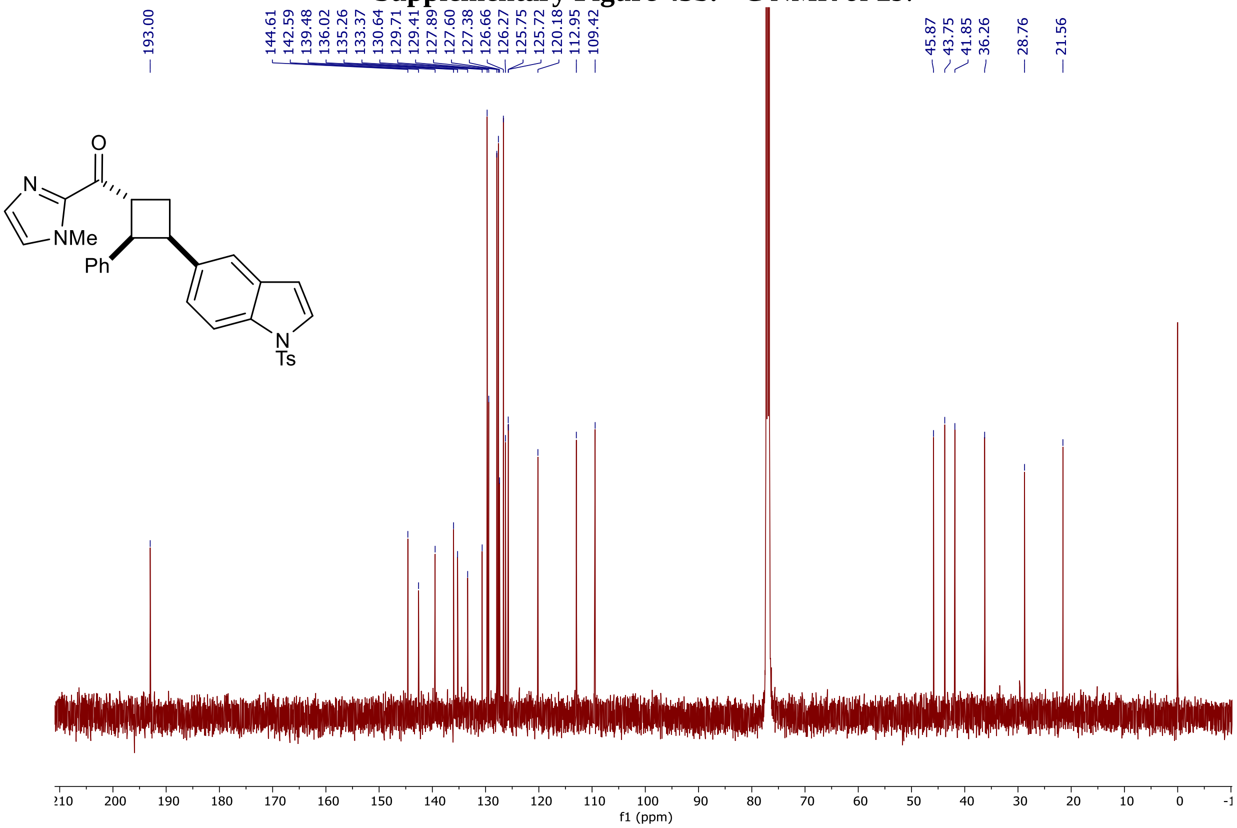

Supplementary Figure 44a. ^{1}H NMR of Major Diastereomer of **31**.

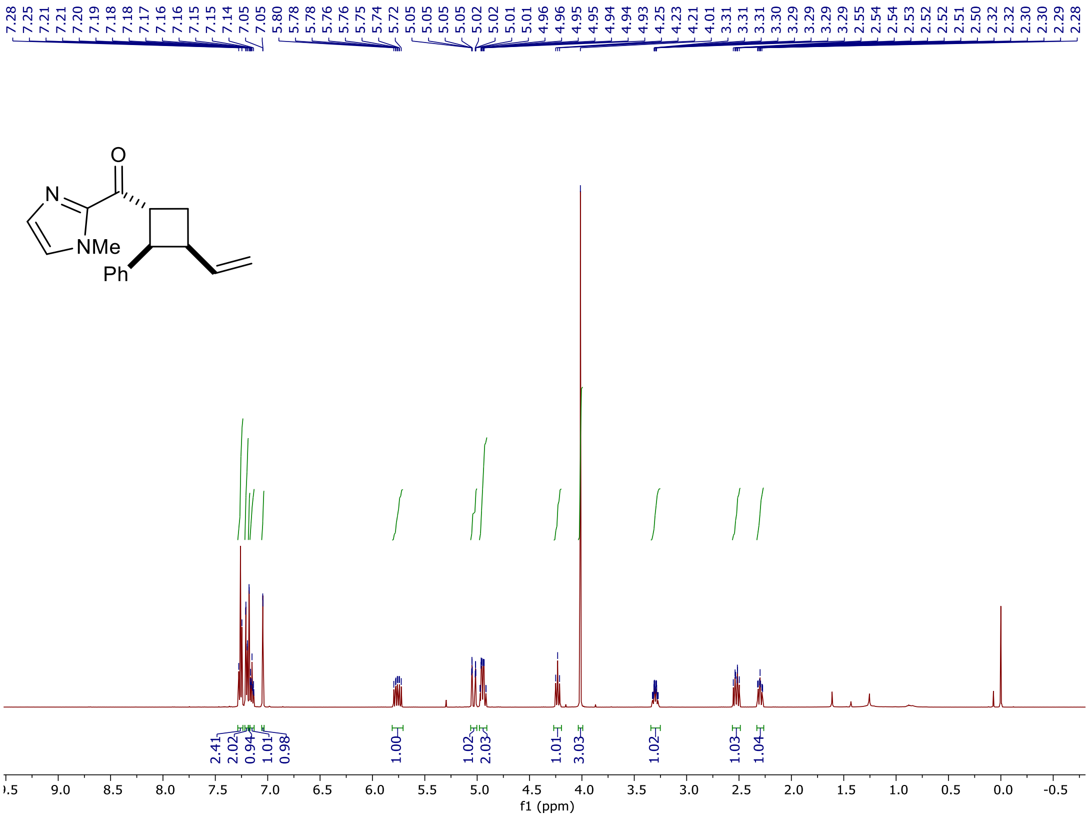

Supplementary Figure 44b. ^{13}C NMR of Major Diastereomer of **31**.

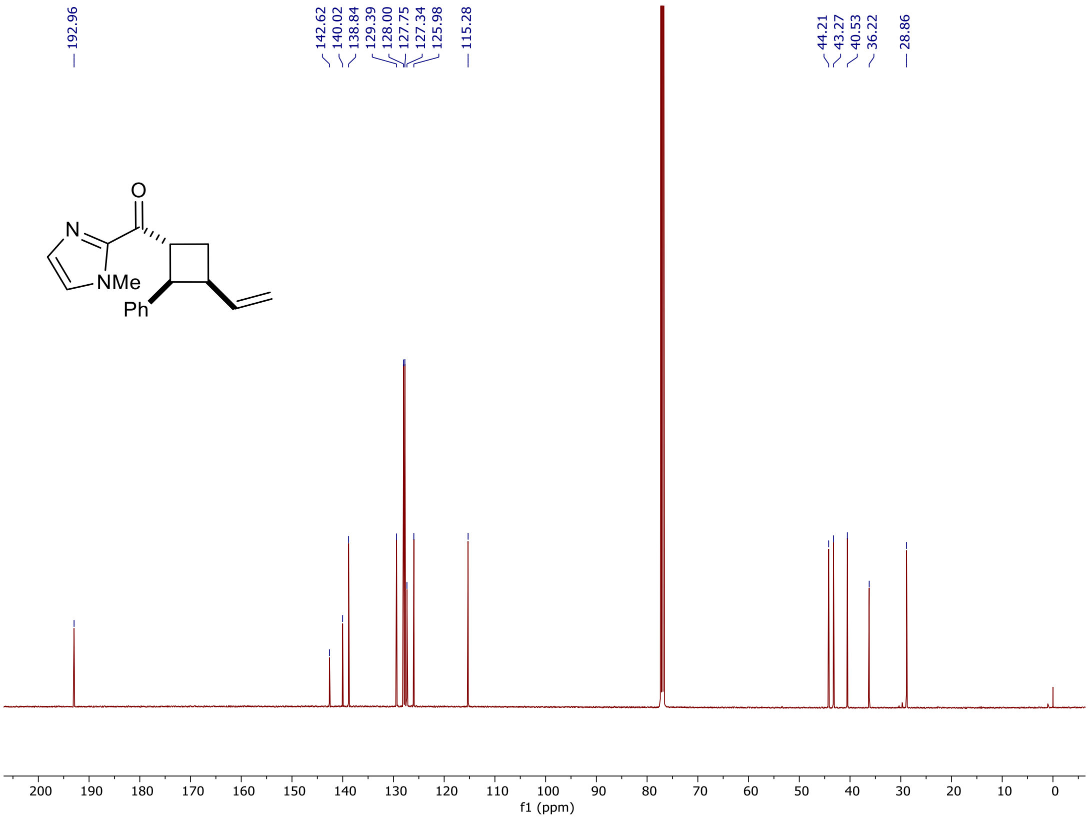

Supplementary Figure 45a. ^{1}H NMR of Minor Diastereomer of **31**.

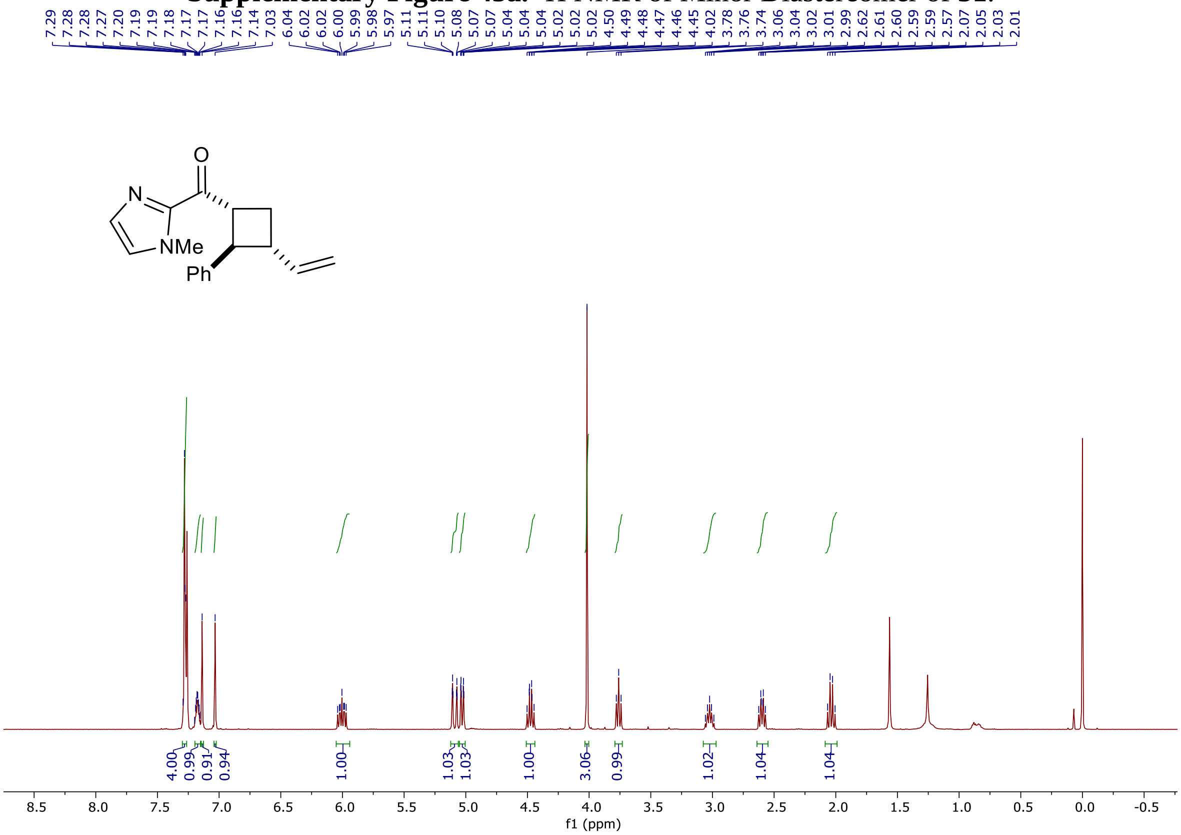

Supplementary Figure 45b. ^{13}C NMR of Minor Diastereomer of **31**.

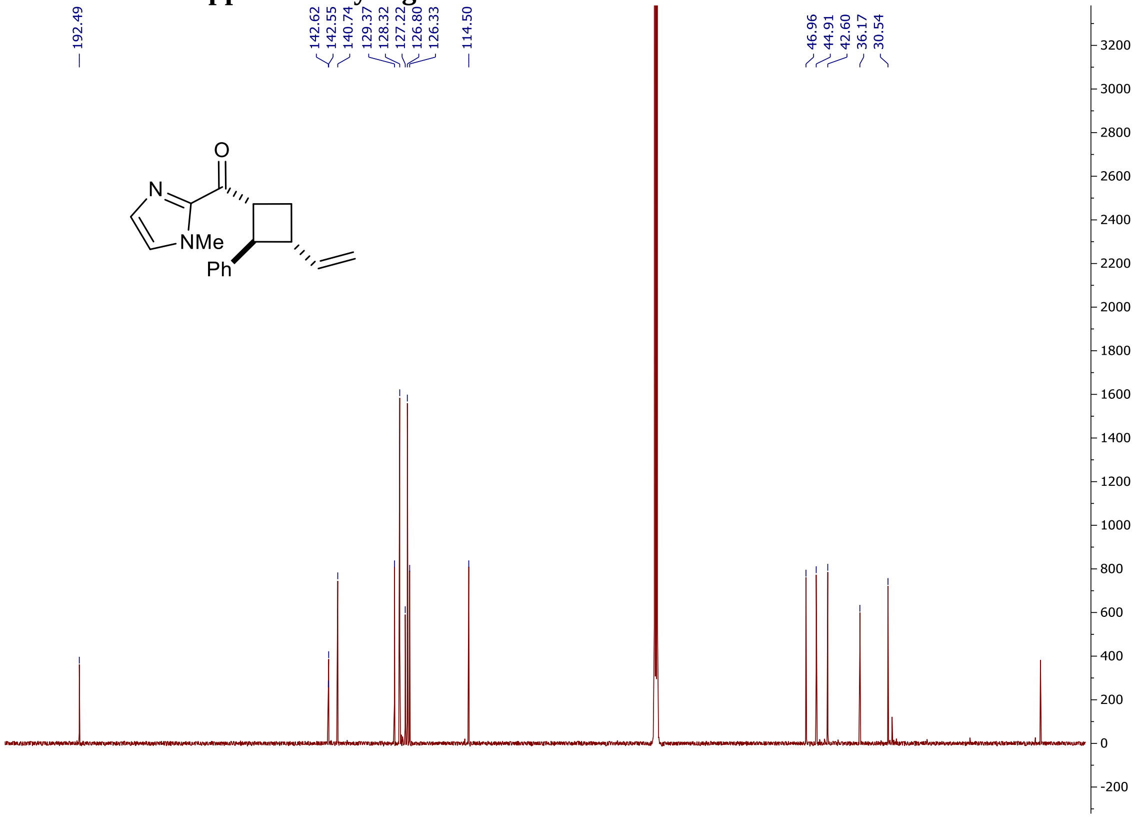

Supplementary Figure 46a. ^{1}H NMR of **35**.

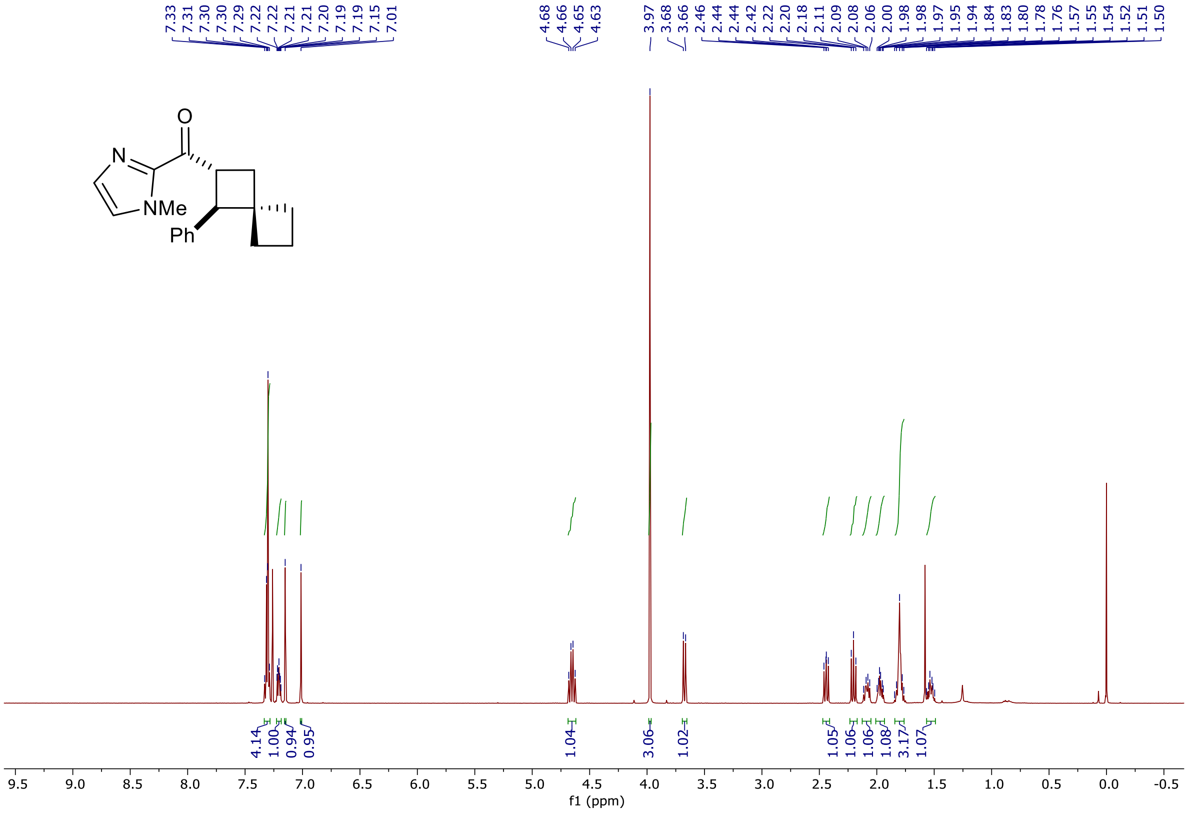

Supplementary Figure 46b. ^{13}C NMR of **35**.

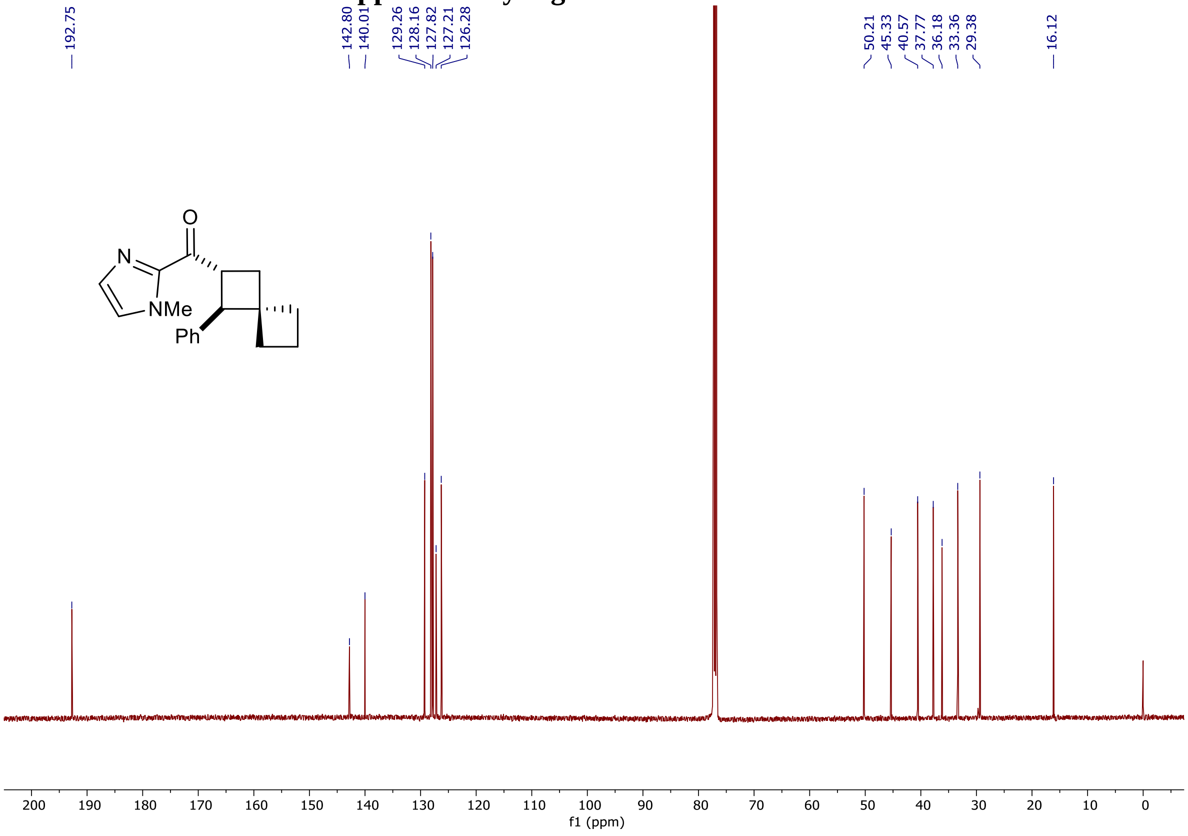

Supplementary Figure 47a. ^{1}H NMR of Major Diastereomer of **36**.

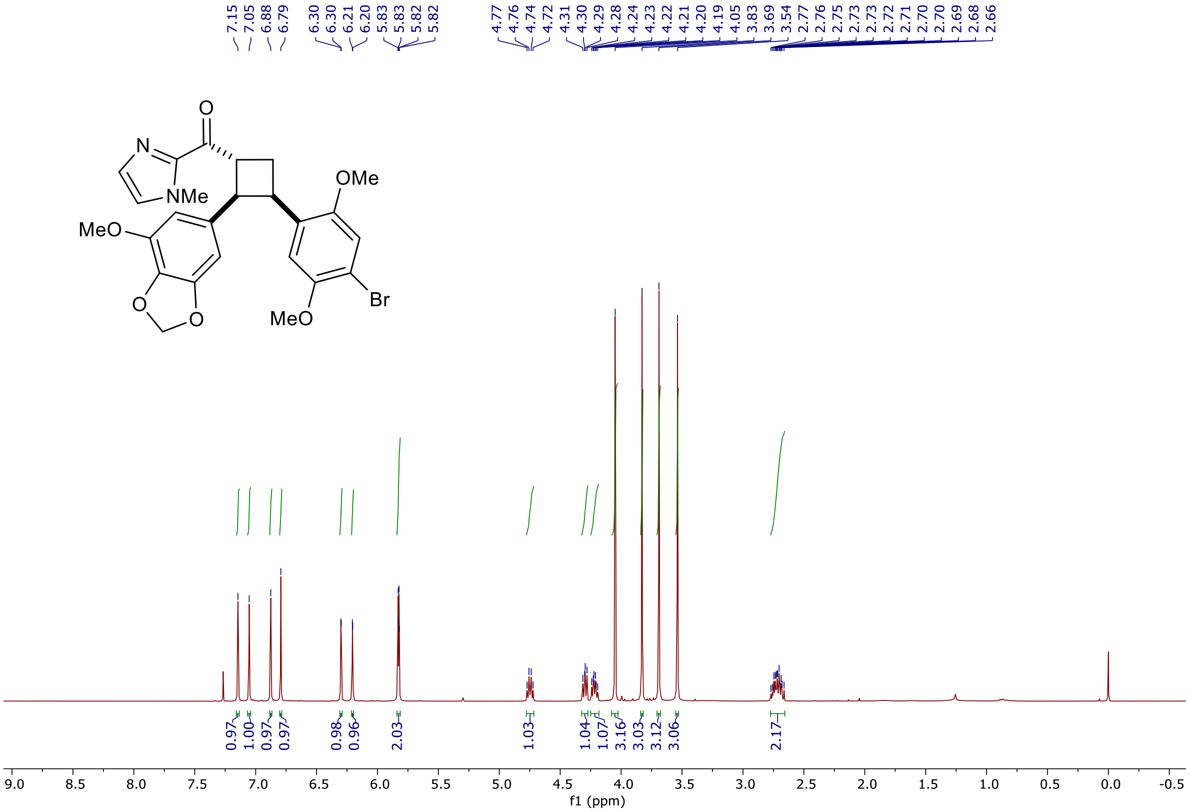

Supplementary Figure 47b. ^{13}C NMR of Major Diastereomer of **36**.

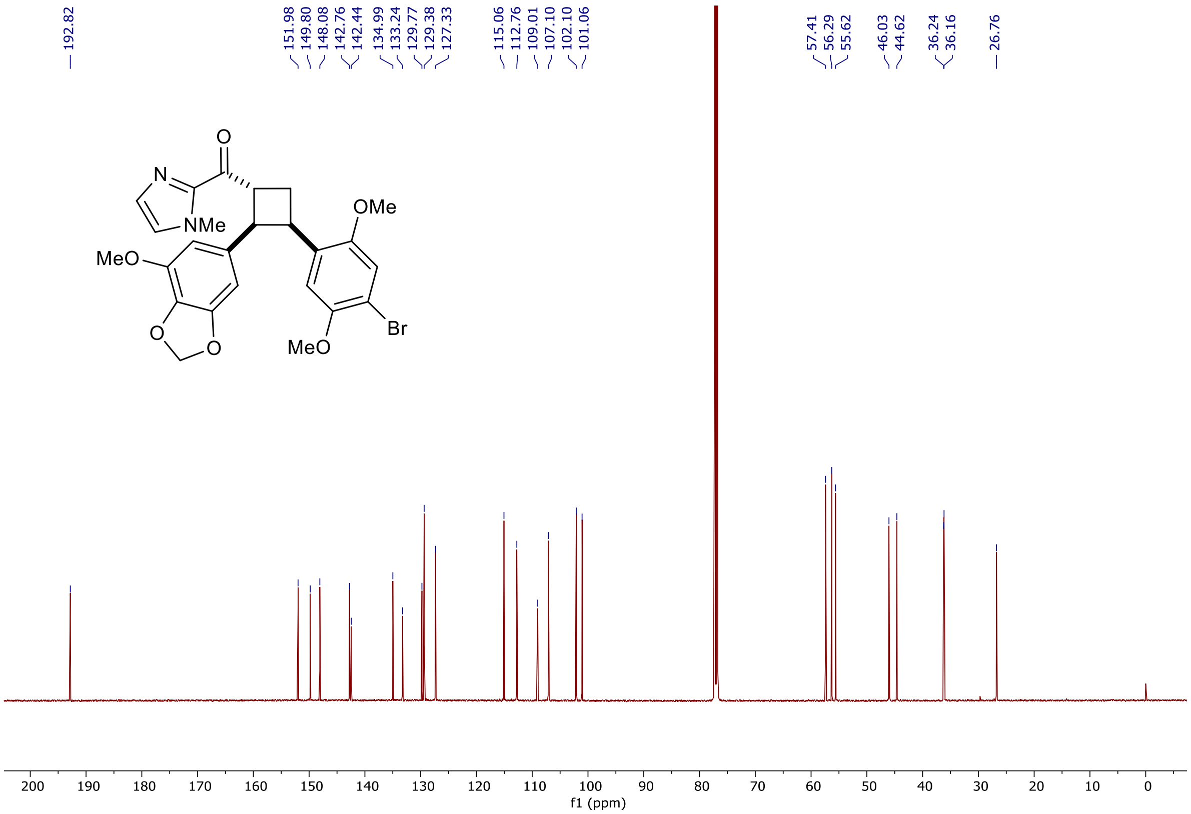

Supplementary Figure 48a. ^{1}H NMR of Minor Diastereomer of **36**.

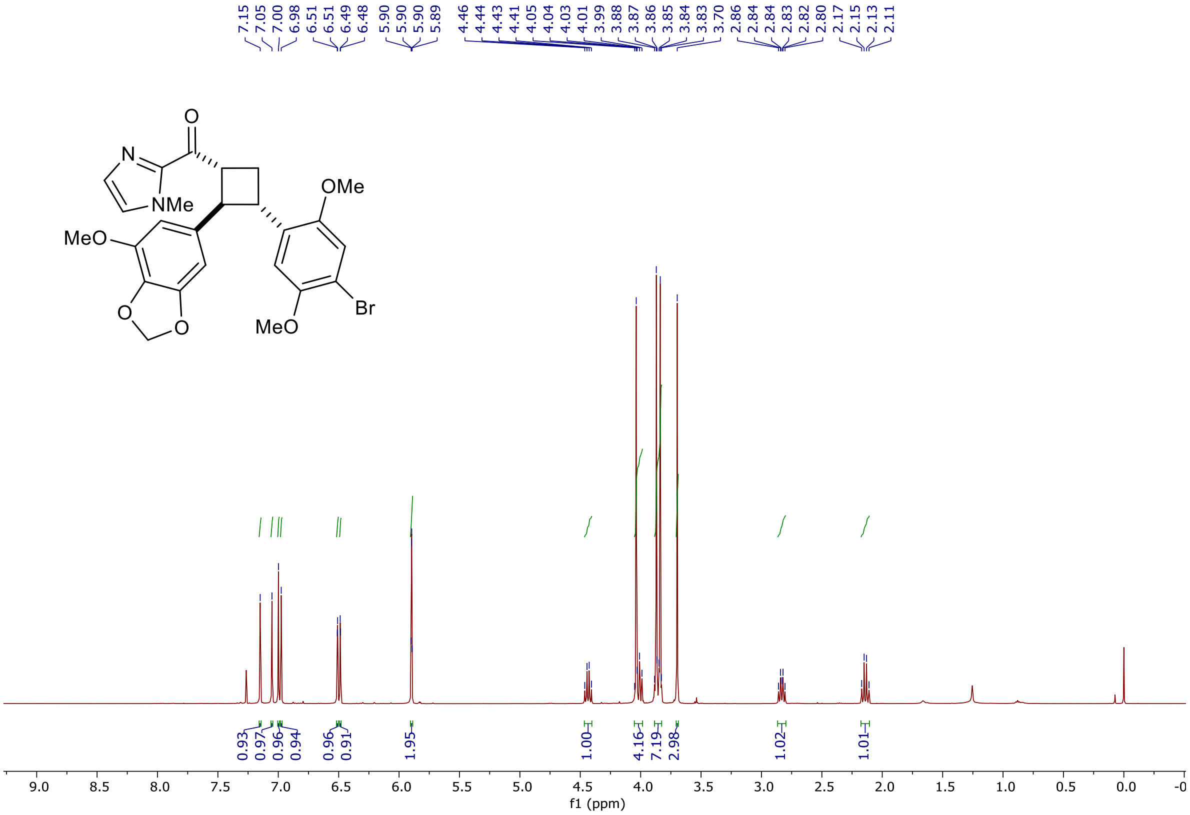

Supplementary Figure 48b. ^{13}C NMR of Minor Diastereomer of **36**.

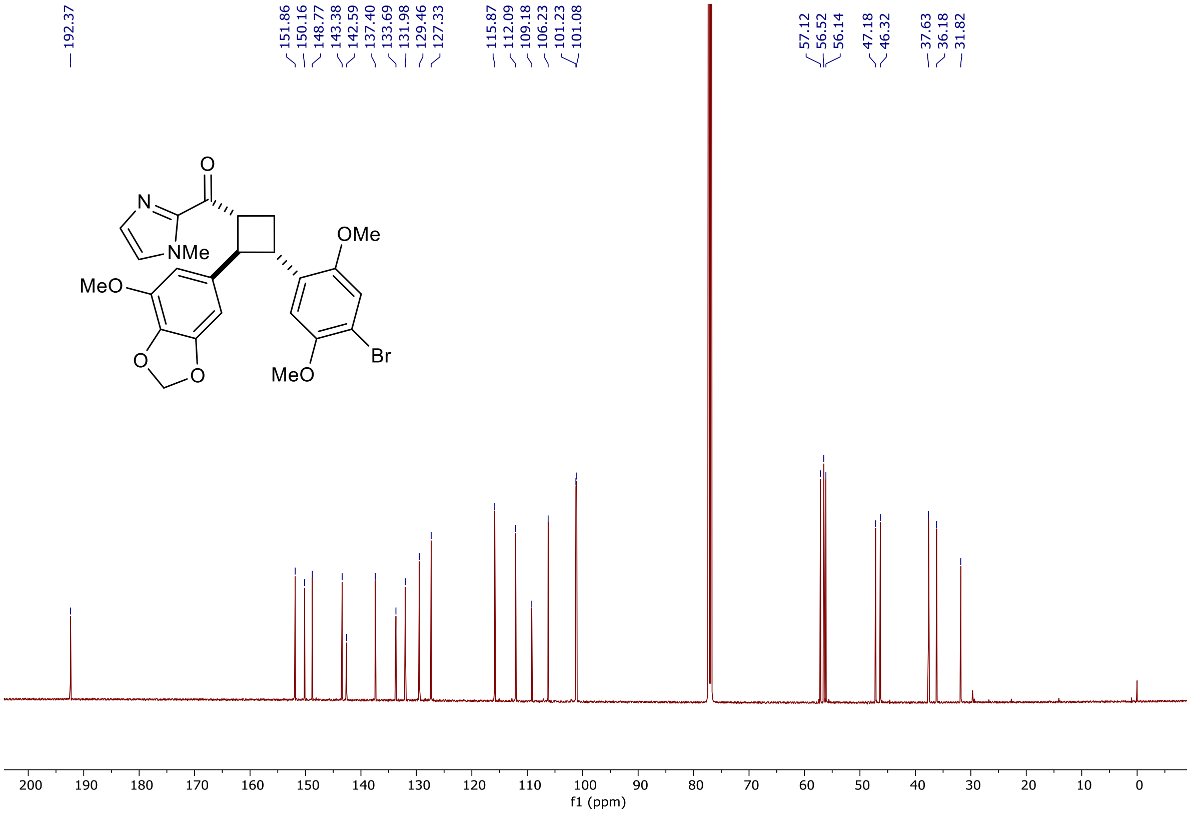

Supplementary Figure 49a. ^{1}H NMR of **37**.

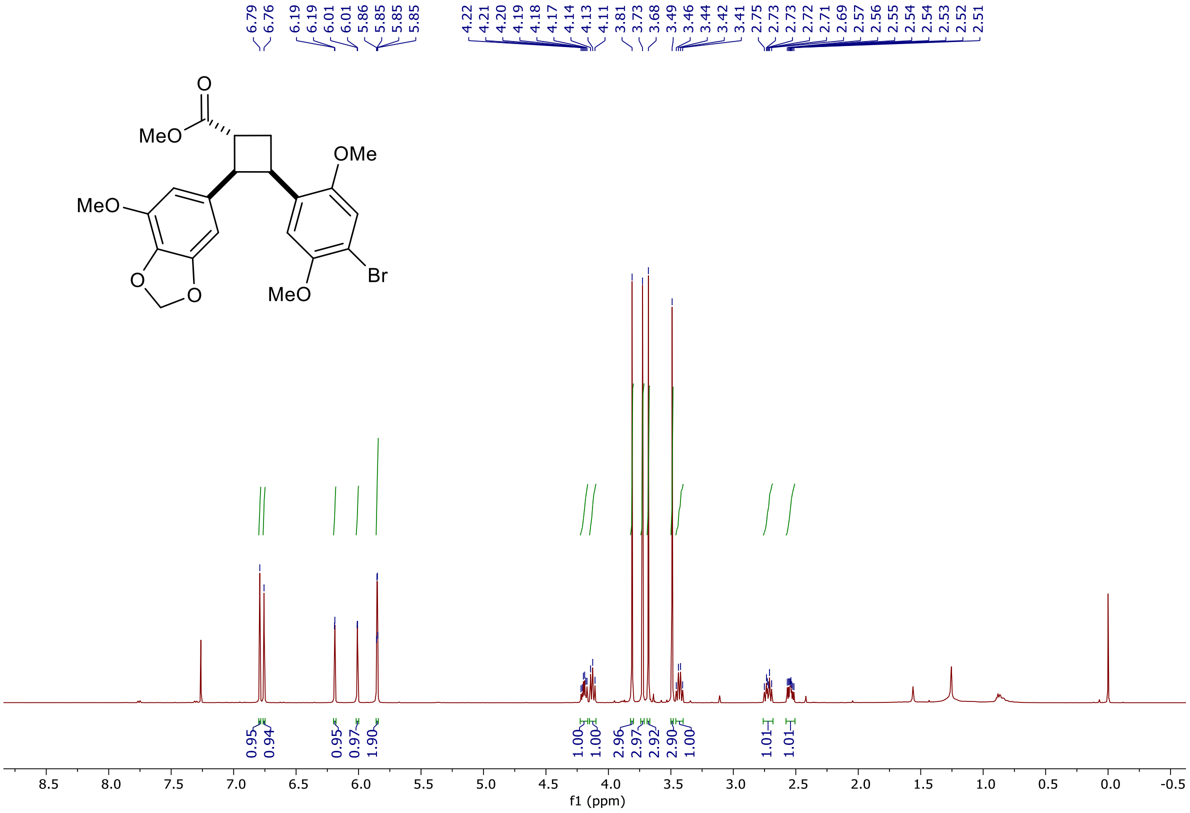

Supplementary Figure 49b. ^{13}C NMR of **37**.

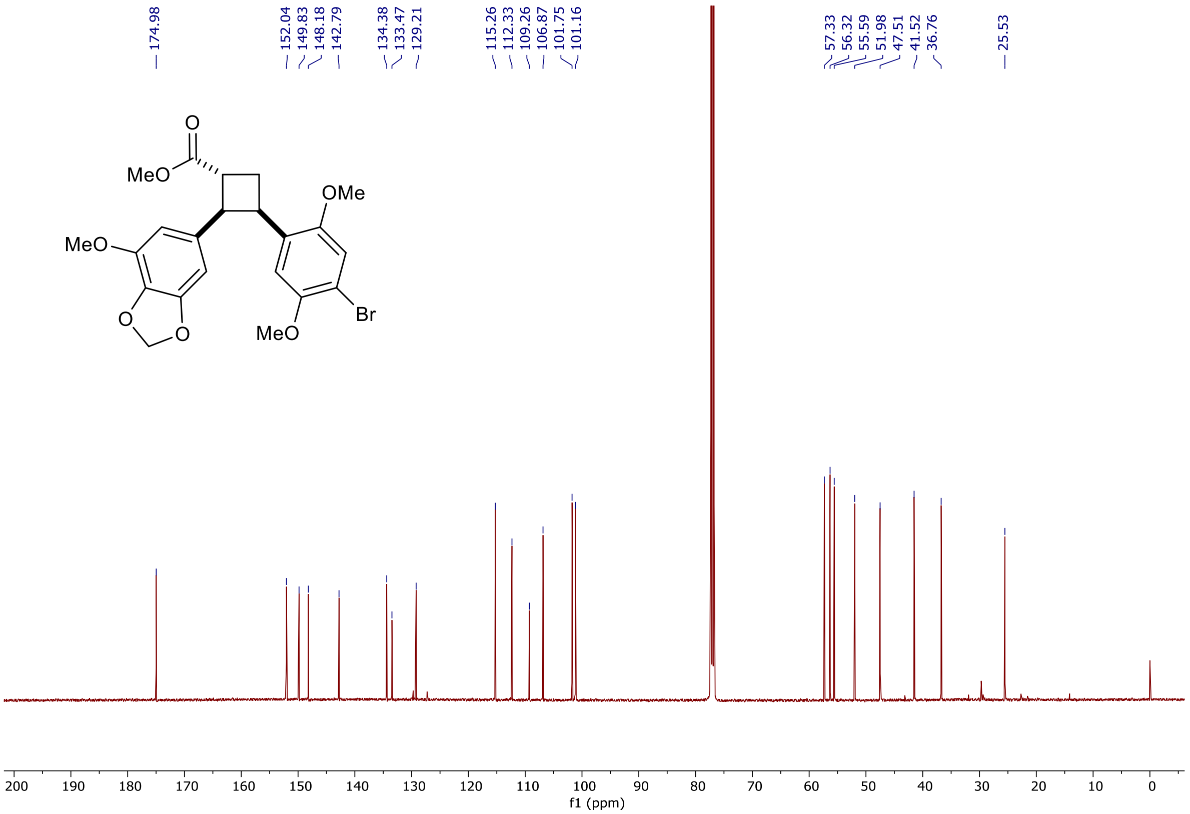

13. HPLC Data

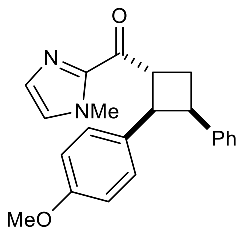

2 – Major Diastereomer
Run Details: HPLC, Daicel CHIRALPAK OD-H,
10.00 µL, gradient 5% to 50% iPrOH/hexanes,
18 minutes, 1 mL/min, 285.0 nm.

Supplementary Figure 50a. Racemic Chromatogram of Major Diastereomer of **2**.

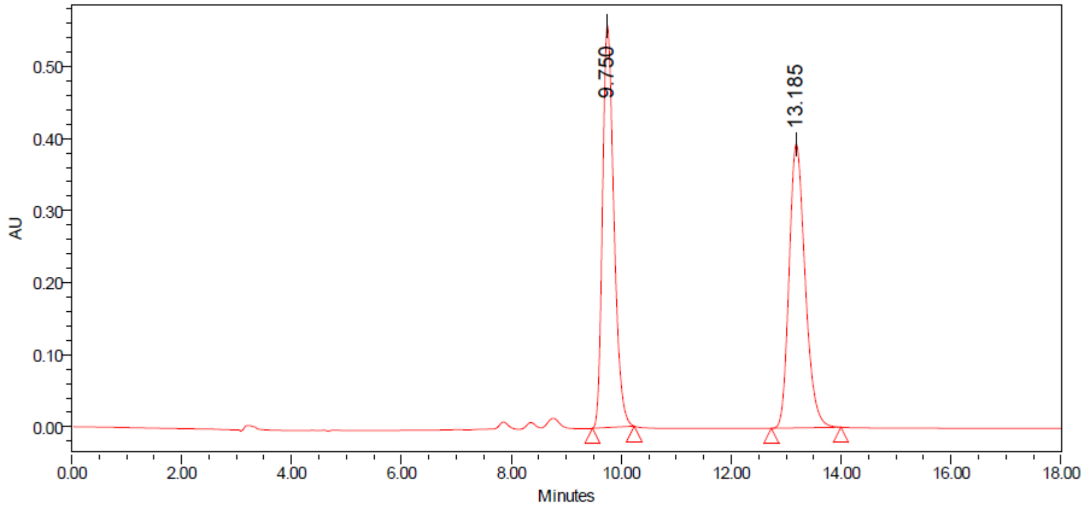

Peak	% Area	Retention Time	Area	Height
1	50.13	9.750	8015689	557798
2	49.87	13.185	7974187	394103
Total	100.00			

Supplementary Figure 50b. Scalemic Chromatogram of Major Diastereomer of **2**.

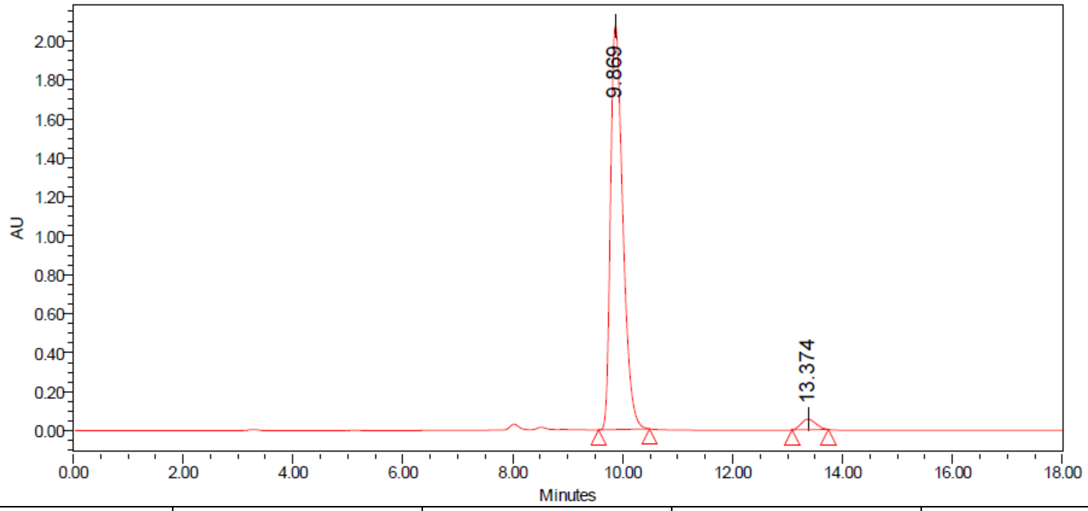

Peak	% Area	Retention Time	Area	Height
1	96.97	9.869	32171708	2072111
2	3.03	13.374	1004823	53875
Total	100.00			

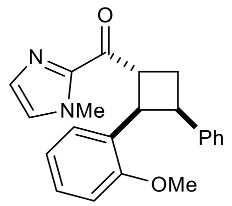

3 – Major Diastereomer
Run Details: HPLC, Daicel CHIRALPAK OD-H,
10.00 µL, gradient 5% to 50% iPrOH/hexanes,
18 minutes, 1 mL/min, 285.0 nm.

Supplementary Figure 51a. Racemic Chromatogram of Major Diastereomer of **3**.

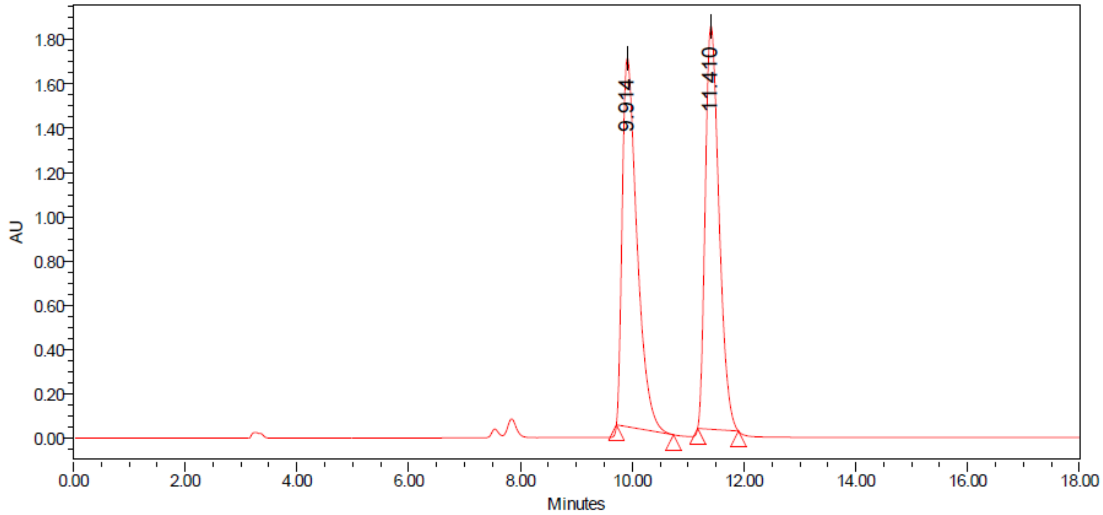

Peak	% Area	Retention Time	Area	Height
1	49.57	9.914	30595009	1665034
2	50.43	11.410	31128716	1821414
Total	100.00			

Supplementary Figure 51b. Scalemic Chromatogram of Major Diastereomer of **3**.

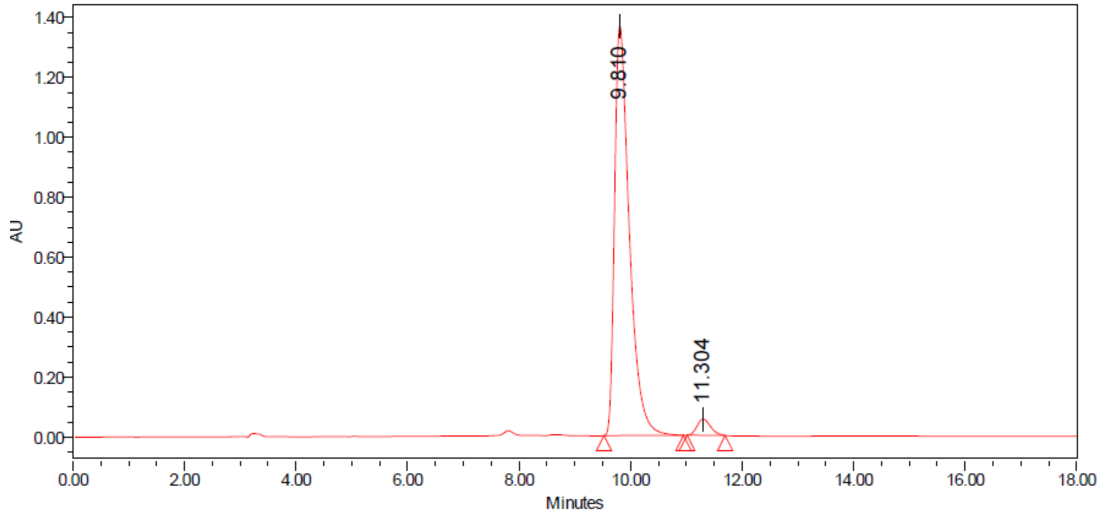

Peak	% Area	Retention Time	Area	Height
1	96.51	9.810	25105171	1366802
2	3.49	11.304	907184	54271
Total	100.00			
Total	100.00			

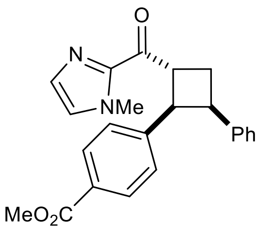

4 – Major Diastereomer
Run Details: HPLC, Daicel CHIRALPAK IC,
10.00 µL, gradient 5% to 50% iPrOH/hexanes,
18 minutes, 1 mL/min, 285.0 nm.

Supplementary Figure 52a. Racemic Chromatogram of Major Diastereomer of **4**.

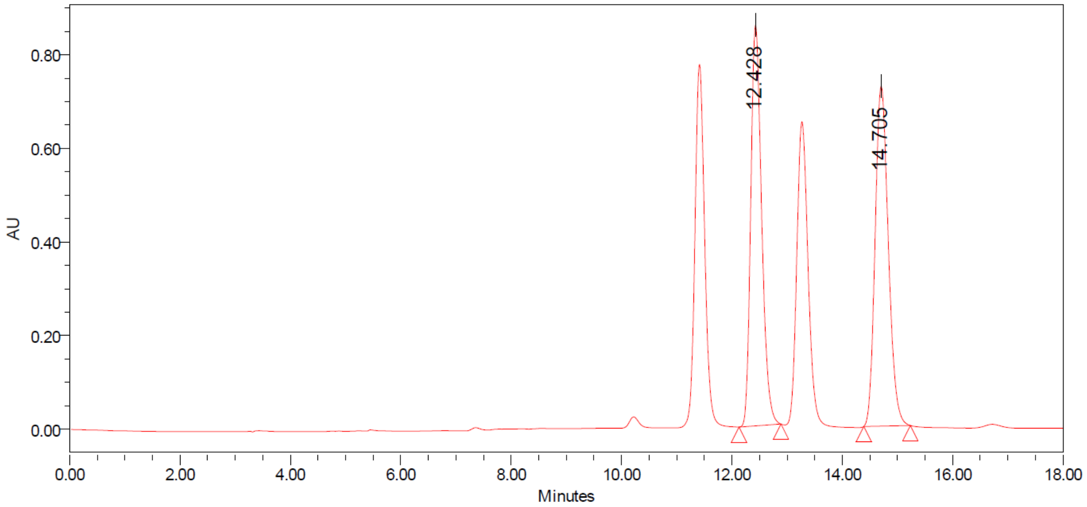

Peak	% Area	Retention Time	Area	Height
1	49.30	12.428	11713056	856183
2	50.70	14.705	12046092	726276
Total	100.00			

Supplementary Figure 52b. Scalemic Chromatogram of Major Diastereomer of **4**.

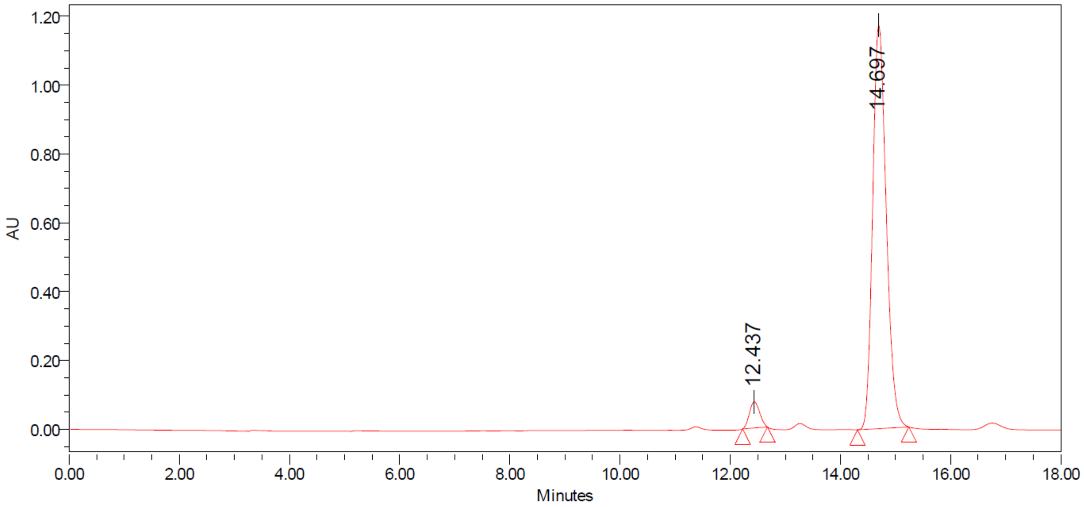

Peak	% Area	Retention Time	Area	Height
1	4.69	12.437	984839	76126
2	95.31	14.697	20027600	1171914
Total	100.00			

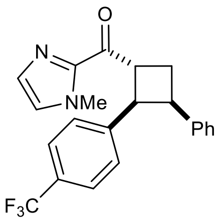

5 – Major Diastereomer
Run Details: HPLC, Daicel CHIRALPAK OD-H,
10.00 µL, gradient 5% to 50% iPrOH/hexanes,
18 minutes, 1 mL/min, 285.0 nm.

Supplementary Figure 53a. Racemic Chromatogram of Major Diastereomer of **5**.

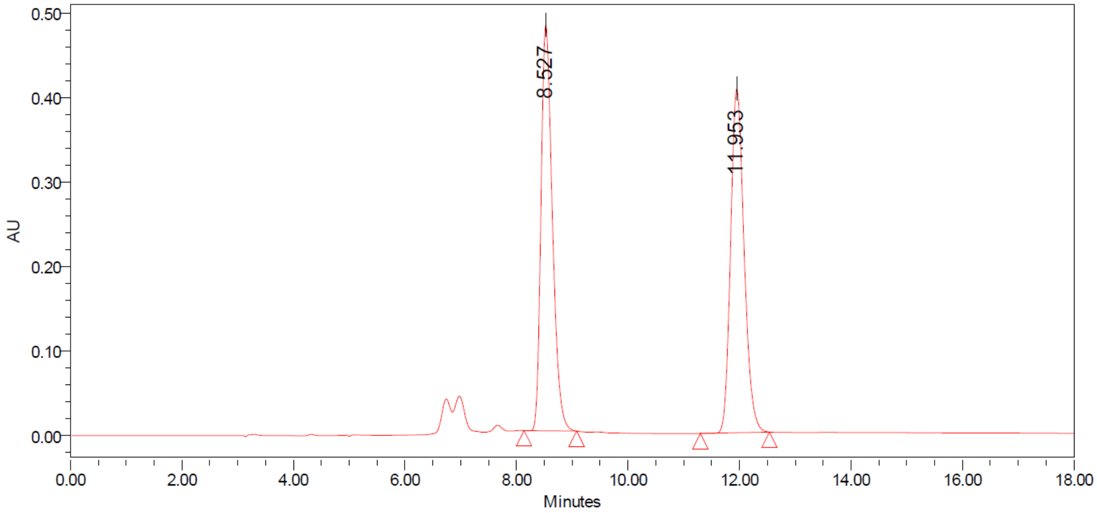

Peak	% Area	Retention Time	Area	Height
1	49.80	8.527	6786719	480179
2	50.20	11.953	6842091	407223
Total	100.00			

Supplementary Figure 53b. Scalemic Chromatogram of Major Diastereomer of **5**.

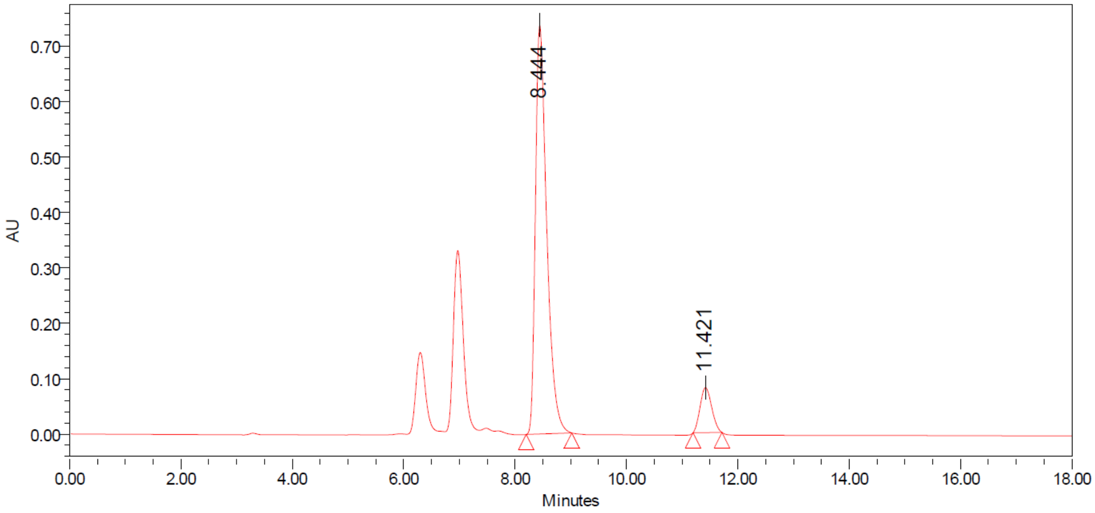

Peak	% Area	Retention Time	Area	Height
1	89.84	8.444	103377818	738048
2	10.16	11.421	1173611	81243
Total	100.00			

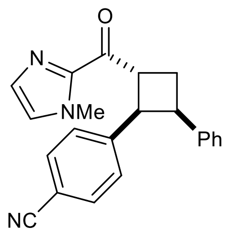

6 – Major Diastereomer
Run Details: HPLC, Daicel CHIRALPAK OD-H,
10.00 µL, gradient 5% to 50% iPrOH/hexanes,
18 minutes, 1 mL/min, 285.0 nm.

Supplementary Figure 54a. Racemic Chromatogram of Major Diastereomer of **6**.

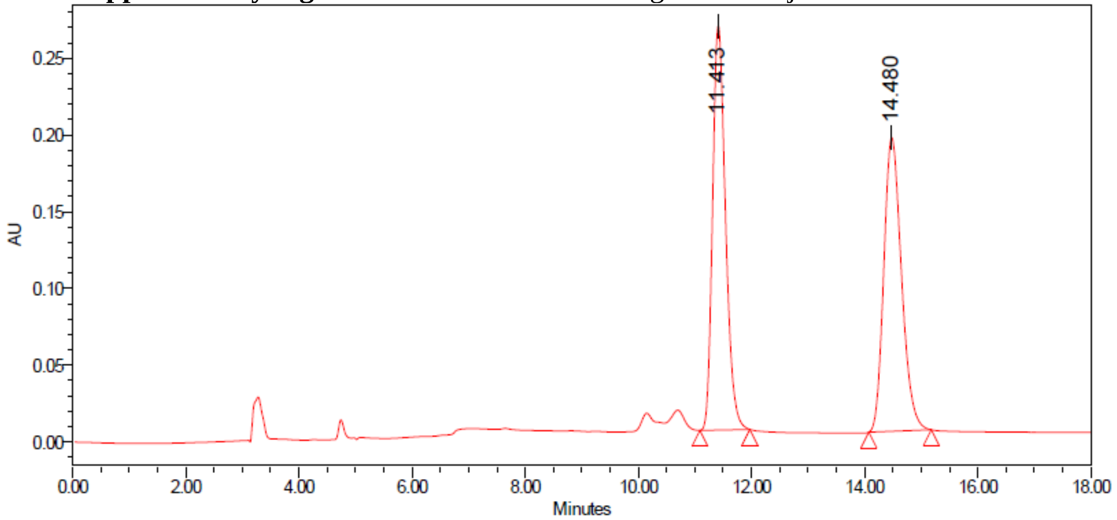

Peak	% Area	Retention Time	Area	Height
1	50.09	11.413	4177021	263466
2	49.91	14.480	4161384	191452
Total	100.00			

Supplementary Figure 54b. Scalemic Chromatogram of Major Diastereomer of **6**.

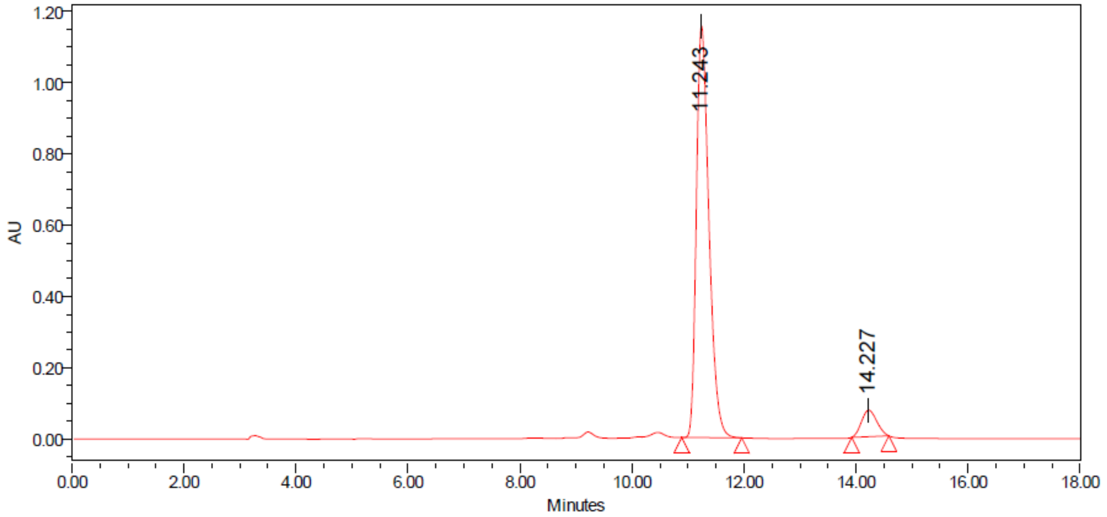

Peak	% Area	Retention Time	Area	Height
1	92.76	11.243	18002516	1154979
2	7.24	14.227	1404431	74207
Total	100.00			

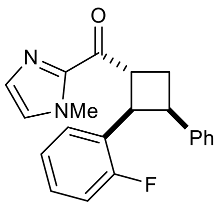

7 – Major Diastereomer
Run Details: HPLC, Daicel CHIRALPAK AD-H,
10.00 µL, gradient 5% to 50% iPrOH/hexanes,
18 minutes, 1 mL/min, 285.0 nm.

Supplementary Figure 55a. Racemic Chromatogram of Major Diastereomer of **7**.

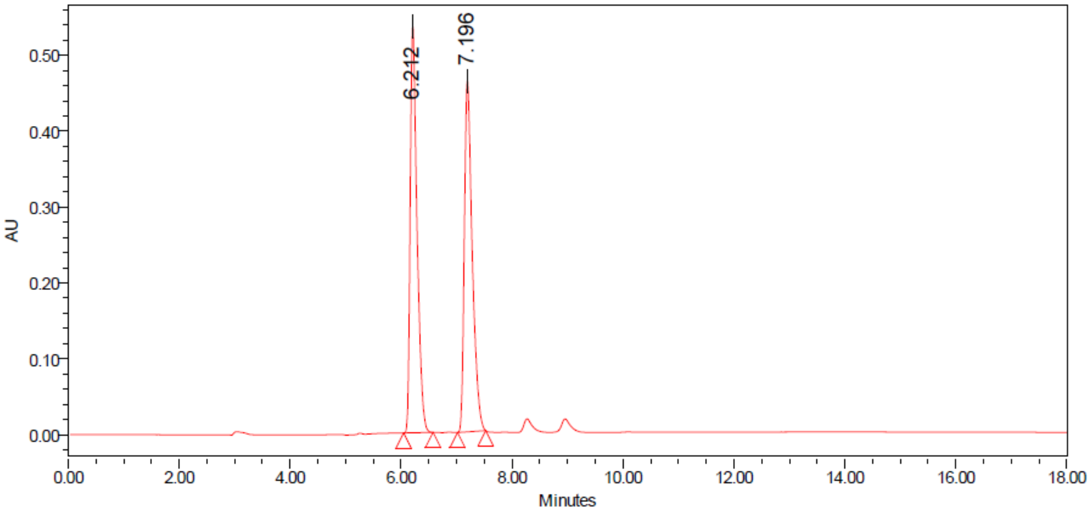

Peak	% Area	Retention Time	Area	Height
1	49.98	6.212	4565931	536238
2	50.02	7.196	4568814	462728
Total	100.00			

Supplementary Figure 55b. Scalemic Chromatogram of Major Diastereomer of **7**.

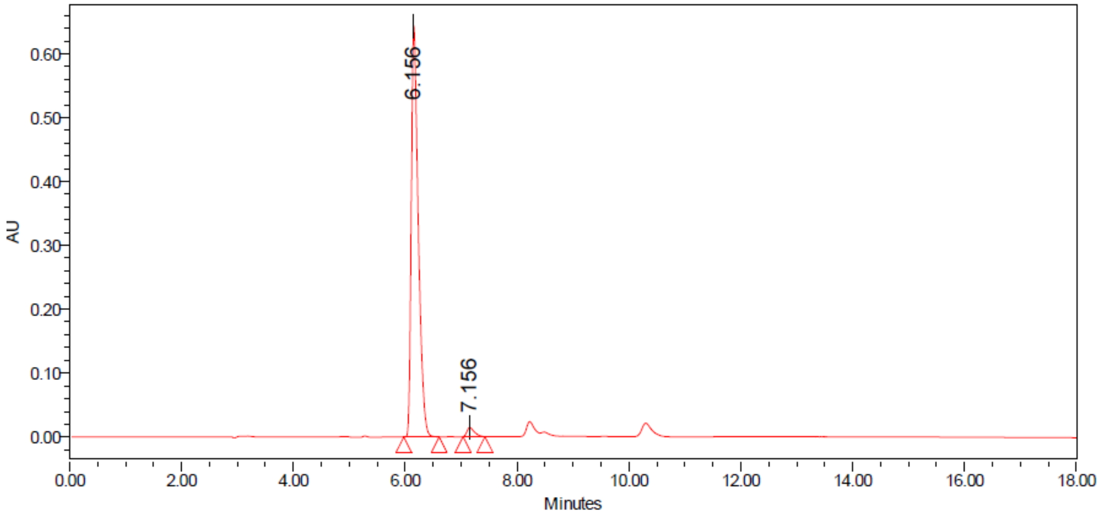

Peak	% Area	Retention Time	Area	Height
1	97.69	6.156	5575042	643845
2	2.31	7.156	131780	14391
Total	100.00			

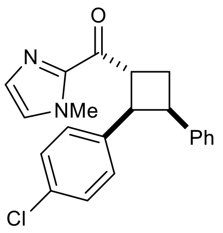

8 – Major Diastereomer
Run Details: HPLC, Daicel CHIRALPAK OD-H,
10.00 µL, gradient 5% to 50% iPrOH/hexanes,
18 minutes, 1 mL/min, 285.0 nm.

Supplementary Figure 56a. Racemic Chromatogram of Major Diastereomer of **8**.

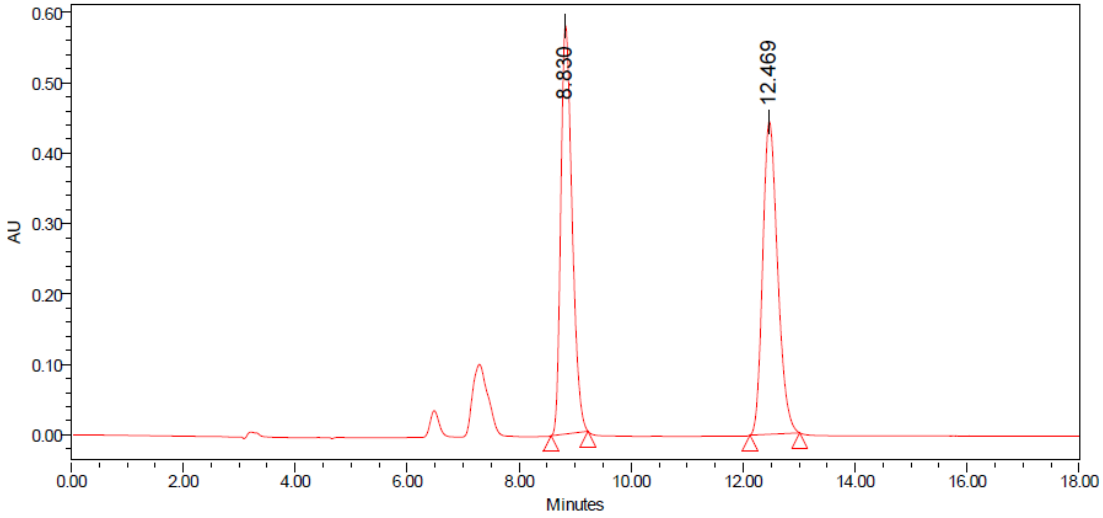

Peak	% Area	Retention Time	Area	Height
1	49.66	8.830	8123700	580289
2	50.34	12.469	8235993	444490
Total	100.00			

Supplementary Figure 56b. Scalemic Chromatogram of Major Diastereomer of **8**.

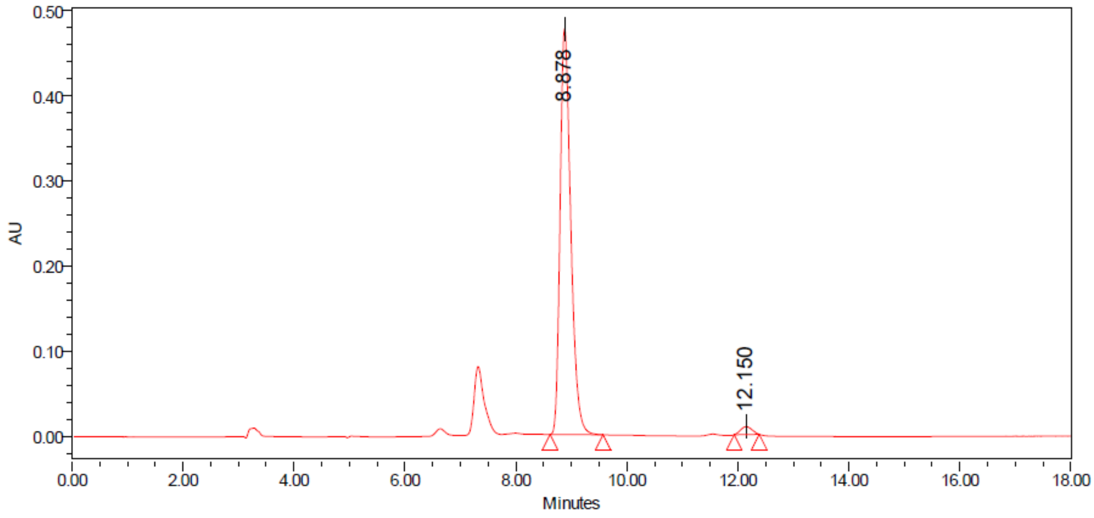

Peak	% Area	Retention Time	Area	Height
1	97.96	8.878	6372481	476435
2	2.04	12.150	132649	9274
Total	100.00			

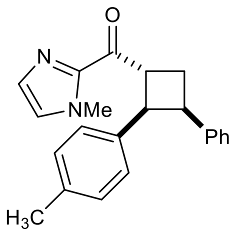

9 – Major Diastereomer
Run Details: HPLC, Daicel CHIRALPAK OD-H,
10.00 µL, gradient 5% to 50% iPrOH/hexanes,
18 minutes, 1 mL/min, 285.0 nm.

Supplementary Figure 57a. Racemic Chromatogram of Major Diastereomer of **9**.

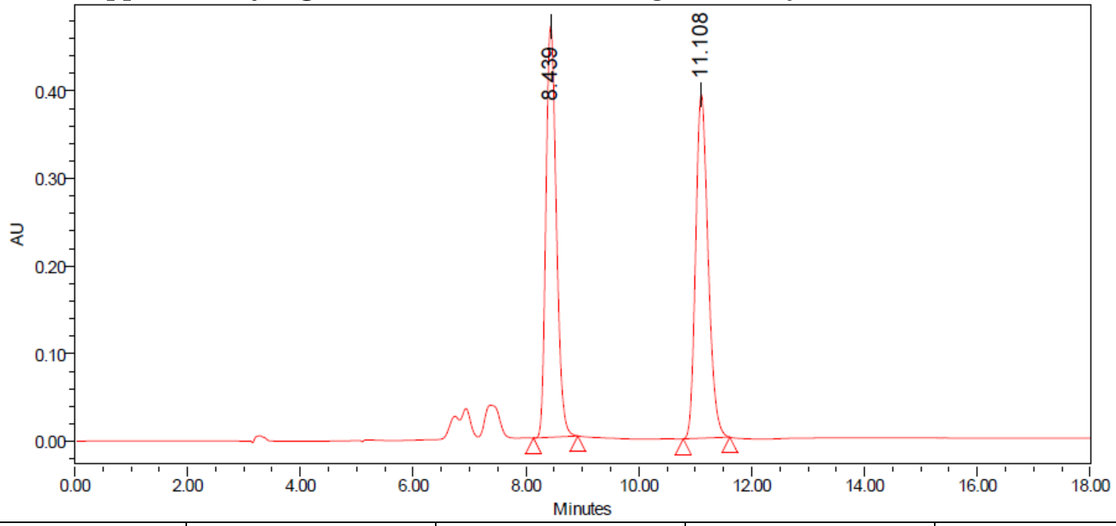

Peak	% Area	Retention Time	Area	Height
1	49.98	8.439	6025706	469776
2	50.02	11.108	6031563	393085
Total	100.00			

Supplementary Figure 57b. Scalemic Chromatogram of Major Diastereomer of **9**.

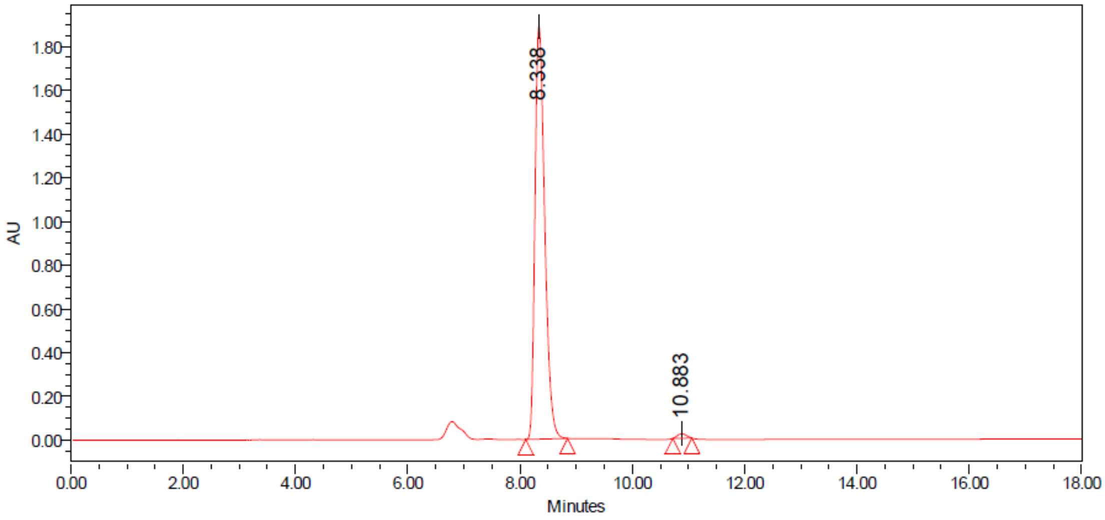

Peak	% Area	Retention Time	Area	Height
1	98.99	8.338	22801875	1888333
2	1.01	10.883	232310	20538
Total	100.00			

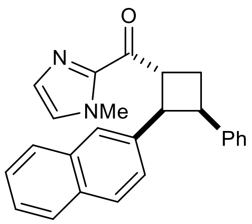

10 – Major Diastereomer
Run Details: HPLC, Daicel CHIRALPAK OD-H,
10.00 µL, gradient 5% to 50% iPrOH/hexanes,
18 minutes, 1 mL/min, 285.0 nm.

Supplementary Figure 58a. Racemic Chromatogram of Major Diastereomer of **10**.

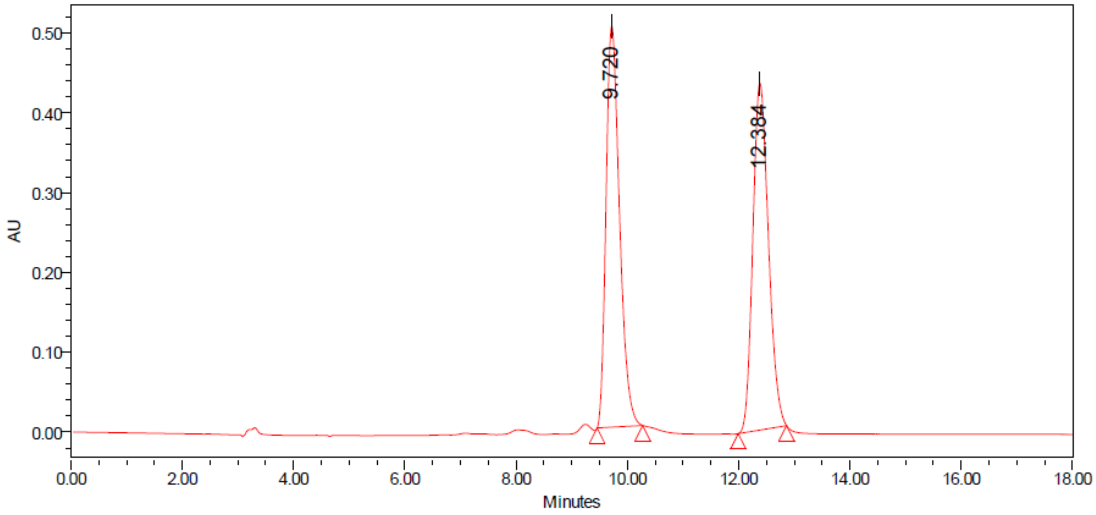

Peak	% Area	Retention Time	Area	Height
1	49.96	9.720	8453746	502796
2	50.04	12.384	8465751	434381
Total	100.00			

Supplementary Figure 58b. Scalemic Chromatogram of Major Diastereomer of **10**.

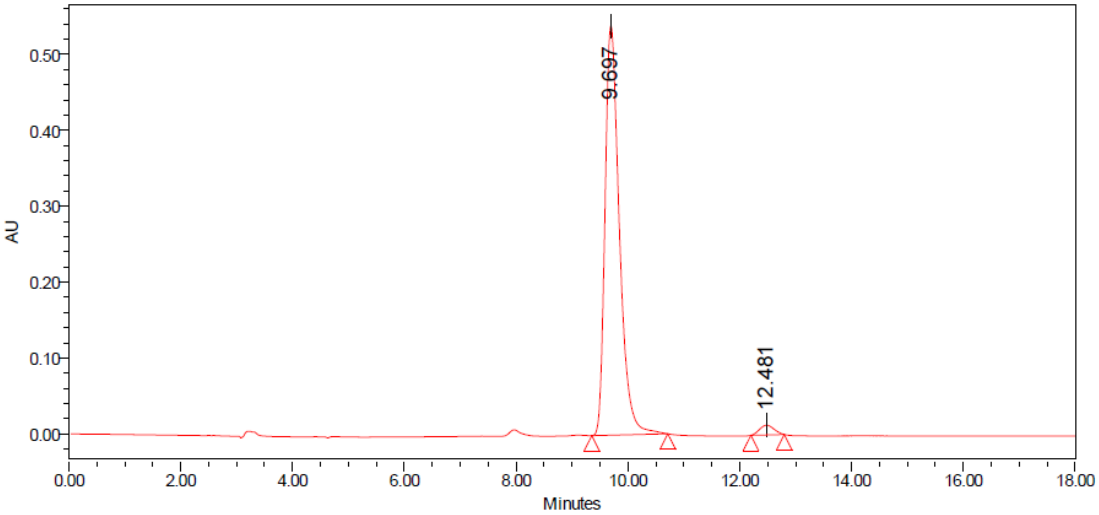

Peak	% Area	Retention Time	Area	Height
1	97.66	9.697	9617702	539301
2	2.34	12.481	229998	12779
Total	100.00			

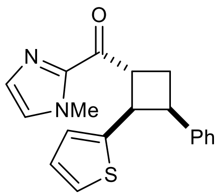

11 – Major Diastereomer
Run Details: HPLC, Daicel CHIRALPAK OD-H,
10.00 µL, gradient 5% to 50% iPrOH/hexanes,
18 minutes, 1 mL/min, 285.0 nm.

Supplementary Figure 59a. Racemic Chromatogram of Major Diastereomer of **11**.

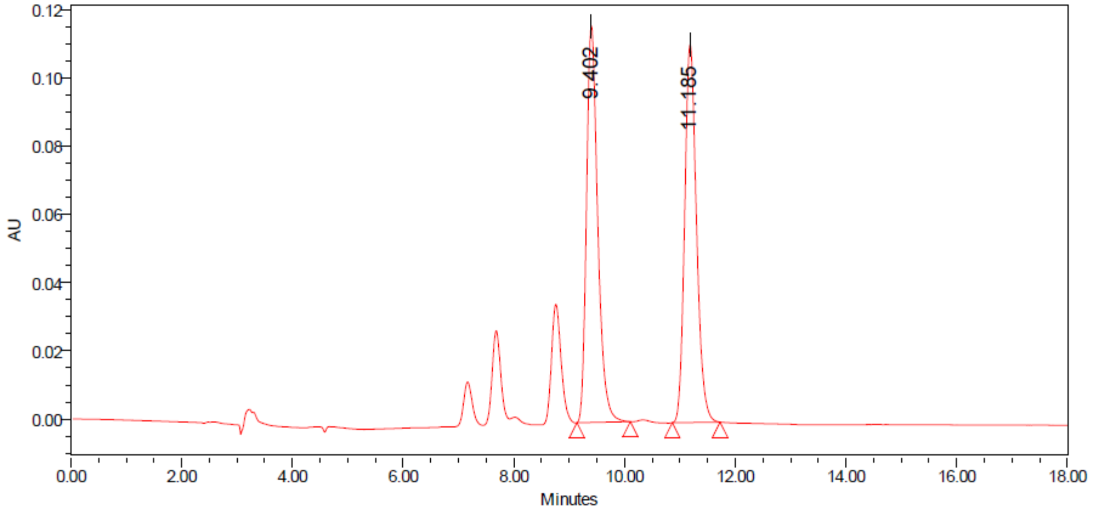

Peak	% Area	Retention Time	Area	Height
1	49.86	9.402	1638697	116327
2	50.14	11.185	1648042	110812
Total	100.00			

Supplementary Figure 59b. Scalemic Chromatogram of Major Diastereomer of **11**.

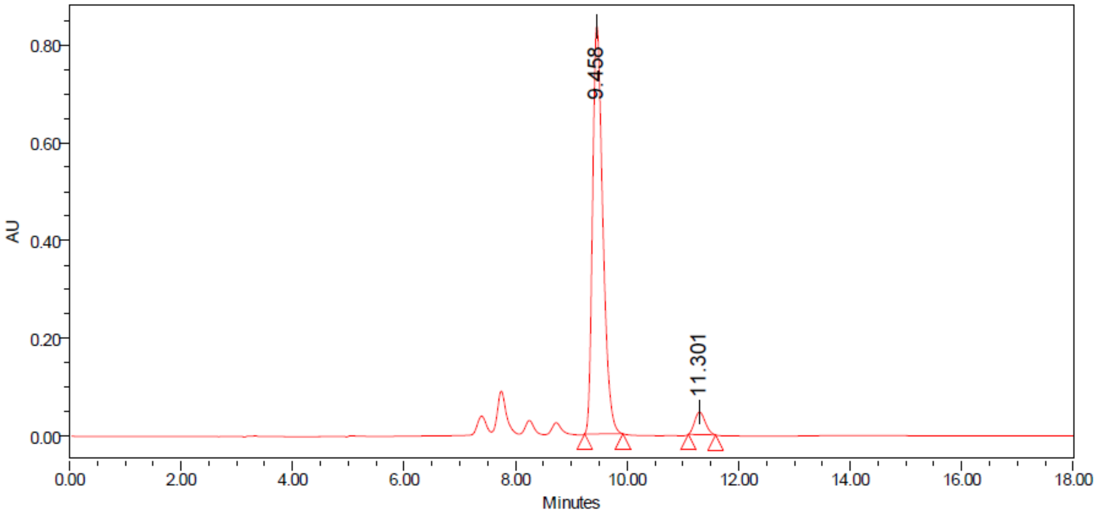

Peak	% Area	Retention Time	Area	Height
1	94.58	9.458	10663442	833712
2	5.42	11.301	610988	45976
Total	100.00			

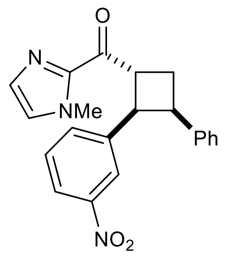

12 – Major Diastereomer
Run Details: HPLC, Daicel CHIRALPAK OD-H,
10.00 µL, gradient 5% to 50% iPrOH/hexanes,
18 minutes, 1 mL/min, 285.0 nm.

Supplementary Figure 60a. Racemic Chromatogram of Major Diastereomer of **12**.

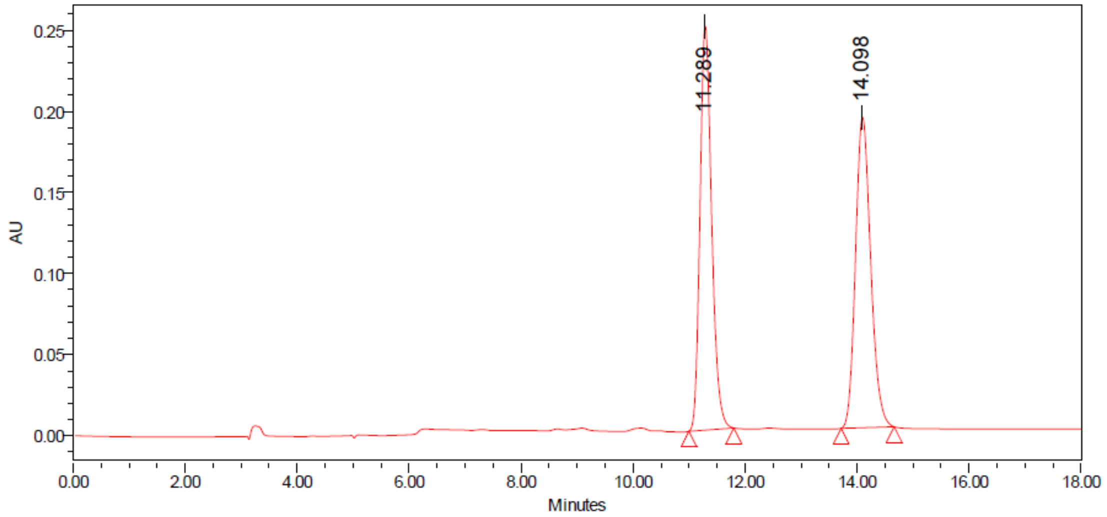

Peak	% Area	Retention Time	Area	Height
1	49.90	11.289	3597092	249200
2	50.10	14.098	3611037	191412
Total	100.00			

Supplementary Figure 60b. Scalemic Chromatogram of Major Diastereomer of **12**.

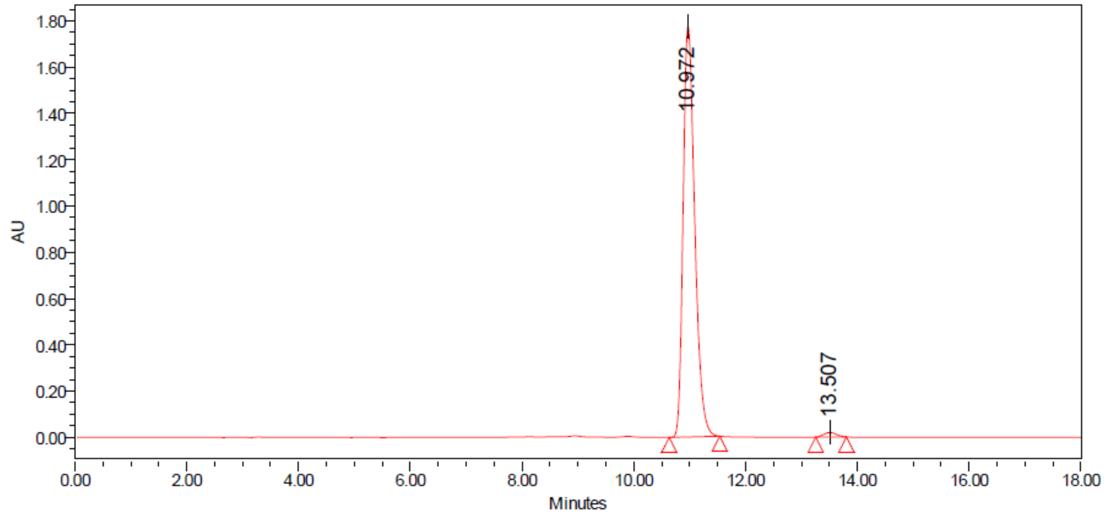

Peak	% Area	Retention Time	Area	Height
1	98.80	10.972	25497032	1775923
2	1.20	13.507	309999	19321
Total	100.00			

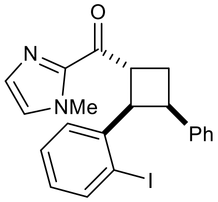

13 – Major Diastereomer
Run Details: HPLC, Daicel CHIRALPAK AD-H,
10.00 µL, gradient 5% to 50% iPrOH/hexanes,
20 minutes, 1 mL/min, 285.0 nm.

Supplementary Figure 61a. Racemic Chromatogram of Major Diastereomer of **13**.

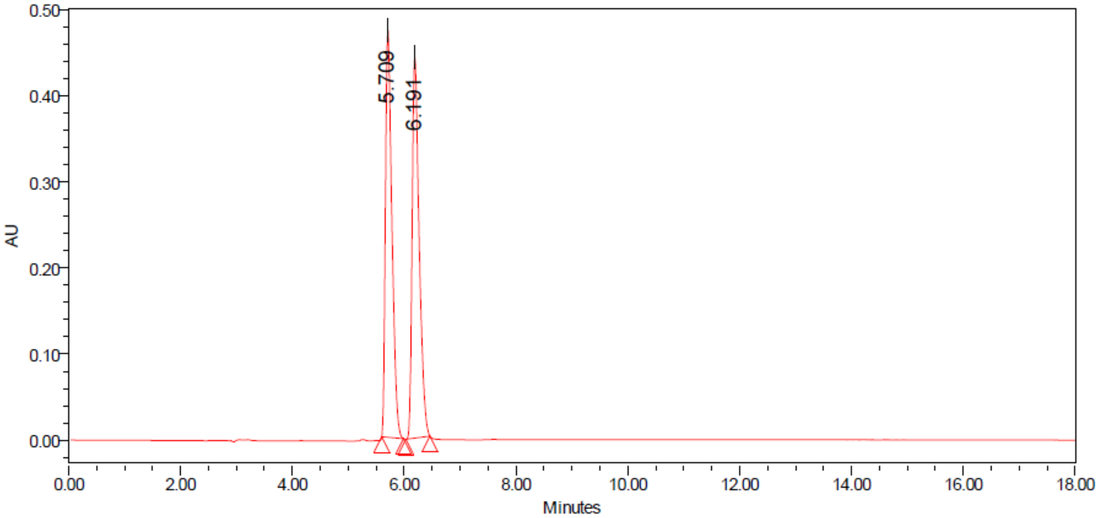

Peak	% Area	Retention Time	Area	Height
1	49.84	5.709	3816214	473791
2	50.16	6.191	3839989	442580
Total	100.00			

Supplementary Figure 61b. Scalemic Chromatogram of Major Diastereomer of **13**.

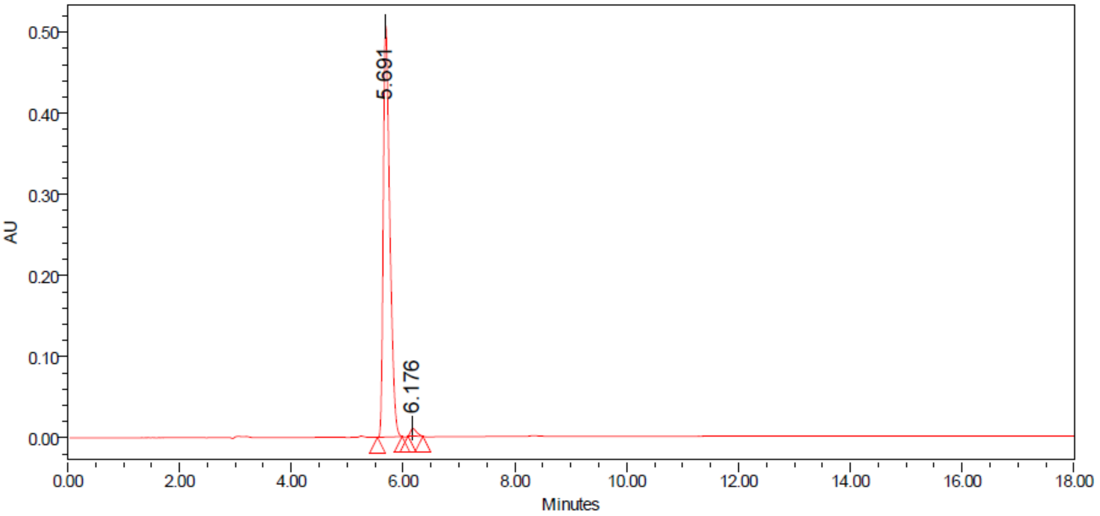

Peak	% Area	Retention Time	Area	Height
1	98.28	5.691	4093630	506805
2	1.72	6.176	71674	9489
Total	100.00			

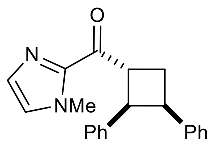

14 – Major Diastereomer
Run Details: HPLC, Daicel CHIRALPAK OD-H,
10.00 μL, gradient 5% to 50% iPrOH/hexanes,
18 minutes, 1 mL/min, 295.0 nm.

Supplementary Figure 62a. Racemic Chromatogram of Major Diastereomer of **14**.

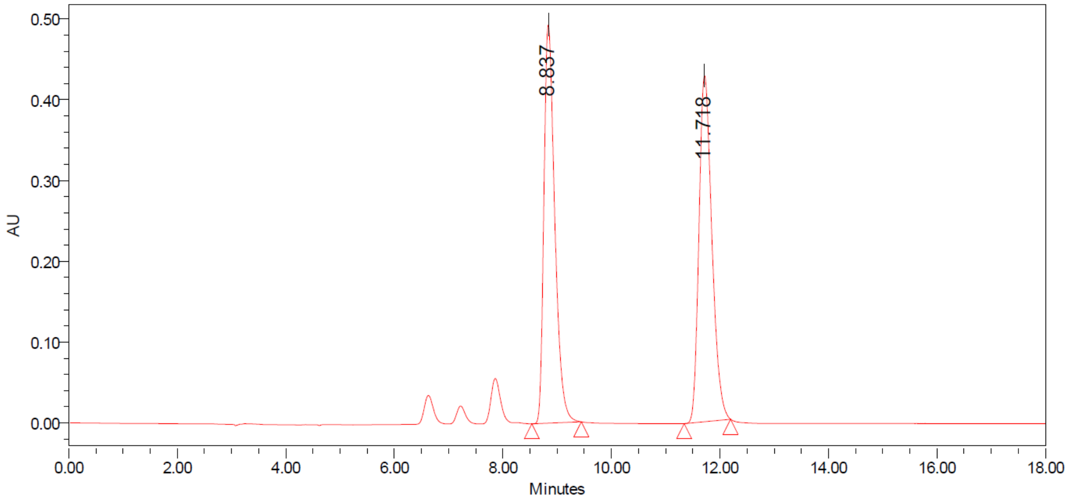

Peak	% Area	Retention Time	Area	Height
1	49.16	8.837	6844274	493024
2	50.84	11.718	7078999	428301
Total	100.00			

Supplementary Figure 62b. Scalemic Chromatogram of Major Diastereomer of **14**.

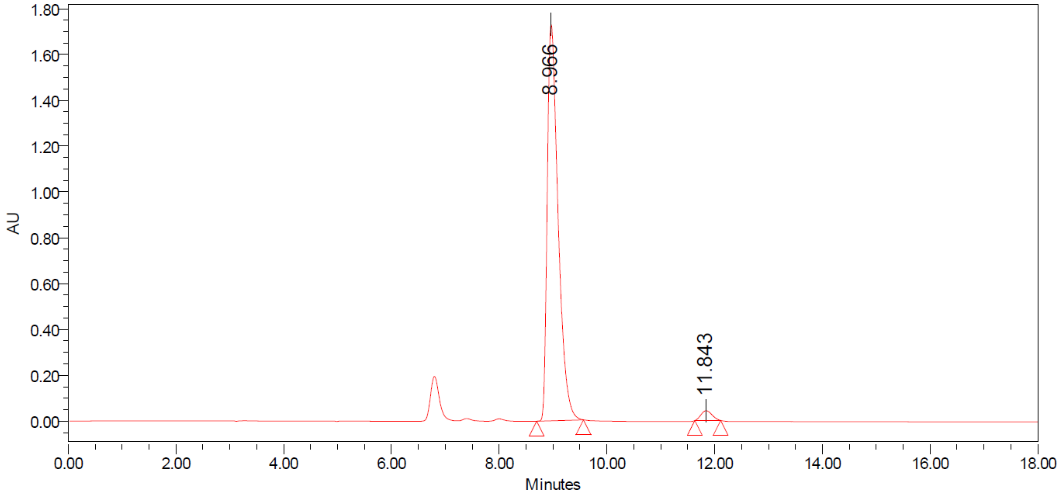

Peak	% Area	Retention Time	Area	Height
1	97.55	8.966	24201086	1729670
2	2.45	11.843	608085	42774
Total	100.00			

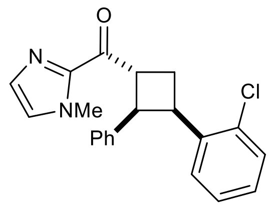

15 – Major Diastereomer
Run Details: HPLC, Daicel CHIRALPAK AD, 10.00 µL, gradient 5% to 50% iPrOH/hexanes, 18 minutes, 1 mL/min, 285.0 nm.

Supplementary Figure 63a. Racemic Chromatogram of Major Diastereomer of **15**.

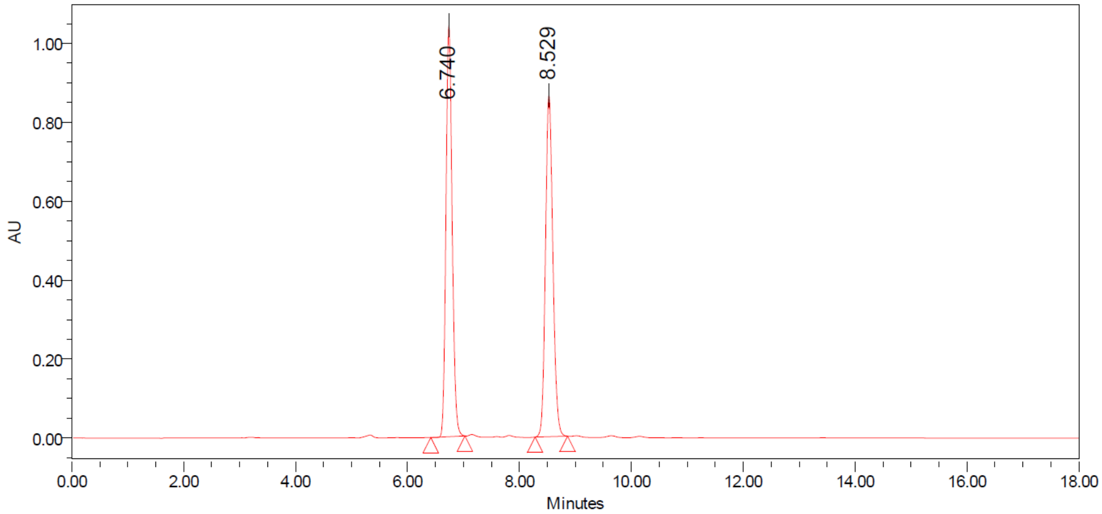

Peak	% Area	Retention Time	Area	Height
1	50.19	6.740	7983942	10419808
2	49.81	8.529	7923782	864460
Total	100.00			

Supplementary Figure 63b. Scalemic Chromatogram of Major Diastereomer of **15**.

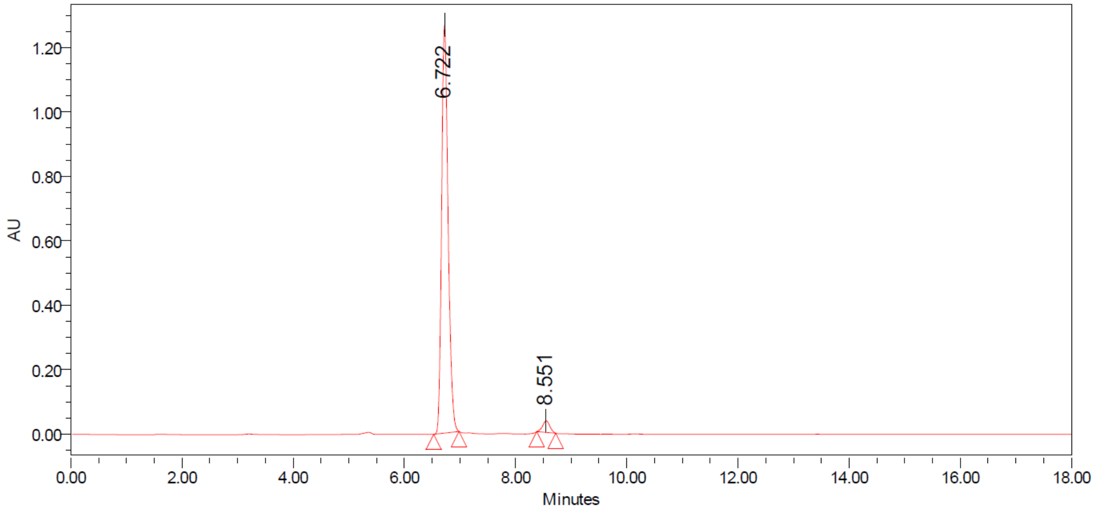

Peak	% Area	Retention Time	Area	Height
1	96.88	6.722	10250099	1265982
2	3.12	8.551	329693	36031
Total	100.00			

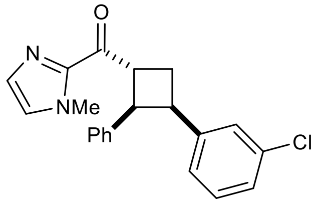

16 – Major Diastereomer
Run Details: HPLC, Daicel CHIRALPAK AD-H,
10.00 μL, gradient 5% to 50% iPrOH/hexanes,
28 minutes, 1 mL/min, 285.0 nm.

Supplementary Figure 64a. Racemic Chromatogram of Major Diastereomer of **16**.

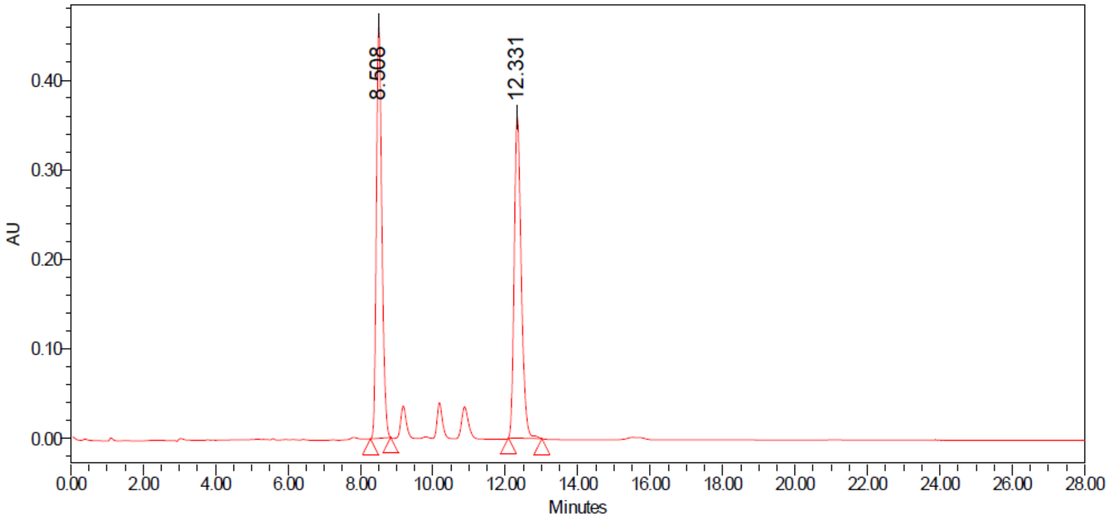

Peak	% Area	Retention Time	Area	Height
1	50.00	8.512	4865982	461766
2	50.00	12.330	4865231	359873
Total	100.00			

Supplementary Figure 64b. Scalemic Chromatogram of Major Diastereomer of **16**.

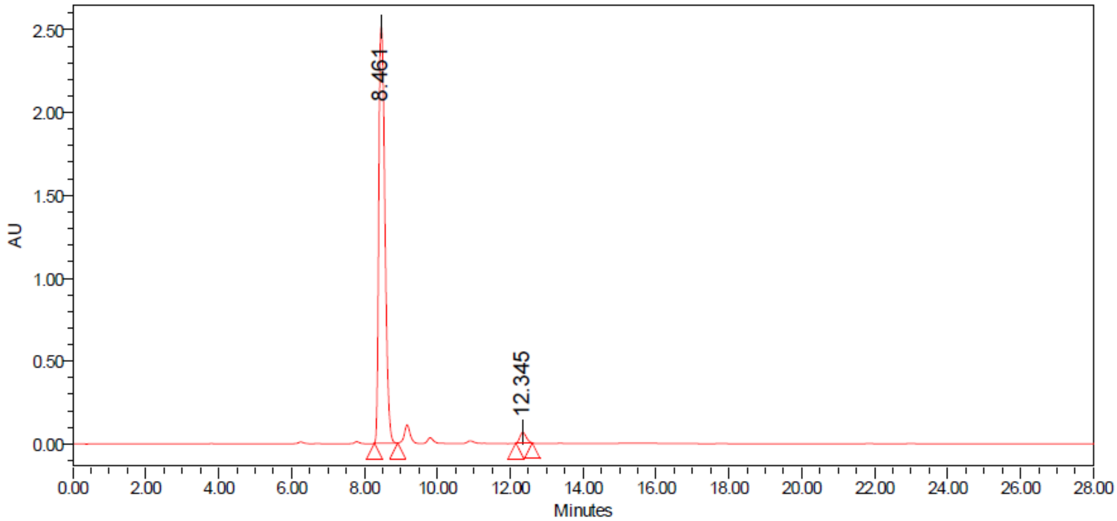

Peak	% Area	Retention Time	Area	Height
1	97.44	8.461	29518349	2513279
2	2.56	12.345	774187	64286
Total	100.00			

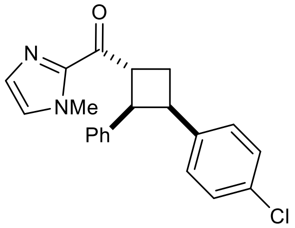

17 – Major Diastereomer
Run Details: HPLC, Daicel CHIRALPAK AD,
10.00 μL, gradient 5% to 50% iPrOH/hexanes,
18 minutes, 1 mL/min, 285.0 nm.

Supplementary Figure 65a. Racemic Chromatogram of Major Diastereomer of **17**.

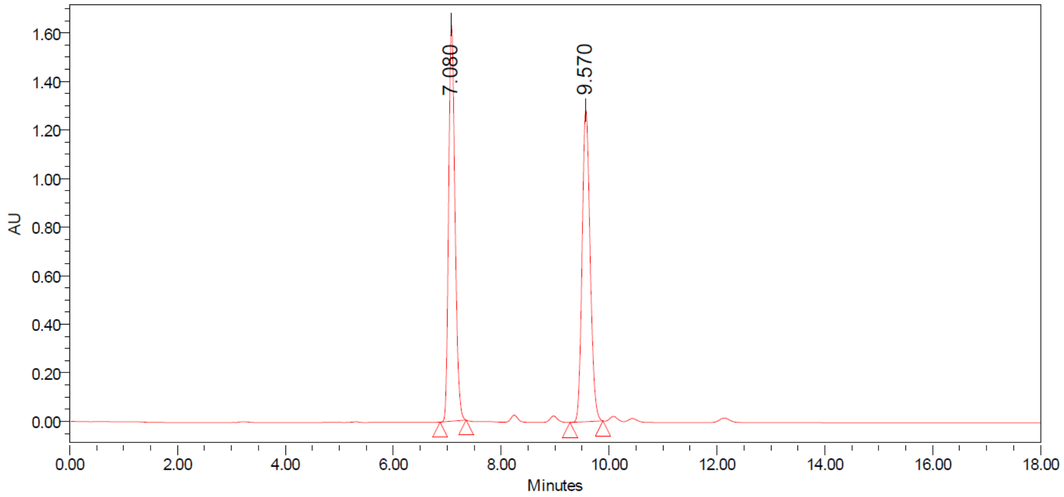

Peak	% Area	Retention Time	Area	Height
1	50.13	7.080	13336366	1633332
2	49.87	9.570	13269671	1283622
Total	100.00			

Supplementary Figure 65b. Scalemic Chromatogram of Major Diastereomer of **17**.

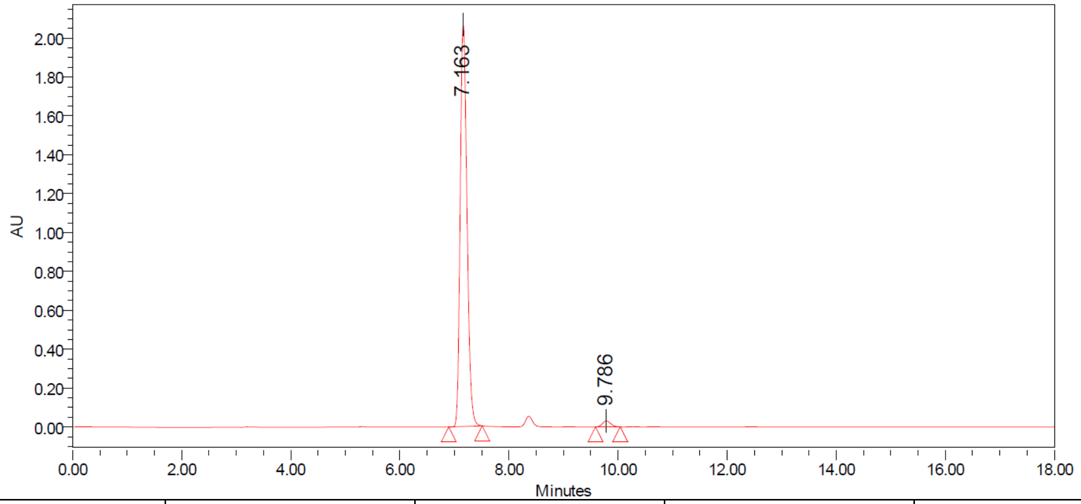

Peak	% Area	Retention Time	Area	Height
1	98.24	7.163	17804766	2064862
2	1.79	9.786	318815	30470
Total	100.00			

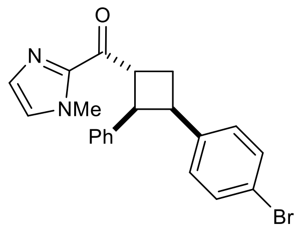

18 – Major Diastereomer
Run Details: HPLC, Daicel CHIRALPAK OD-H,
10.00 µL, gradient 5% to 30% iPrOH/hexanes,
20 minutes, 1 mL/min, 285.0 nm.

Supplementary Figure 66a. Racemic Chromatogram of Major Diastereomer of **18**.

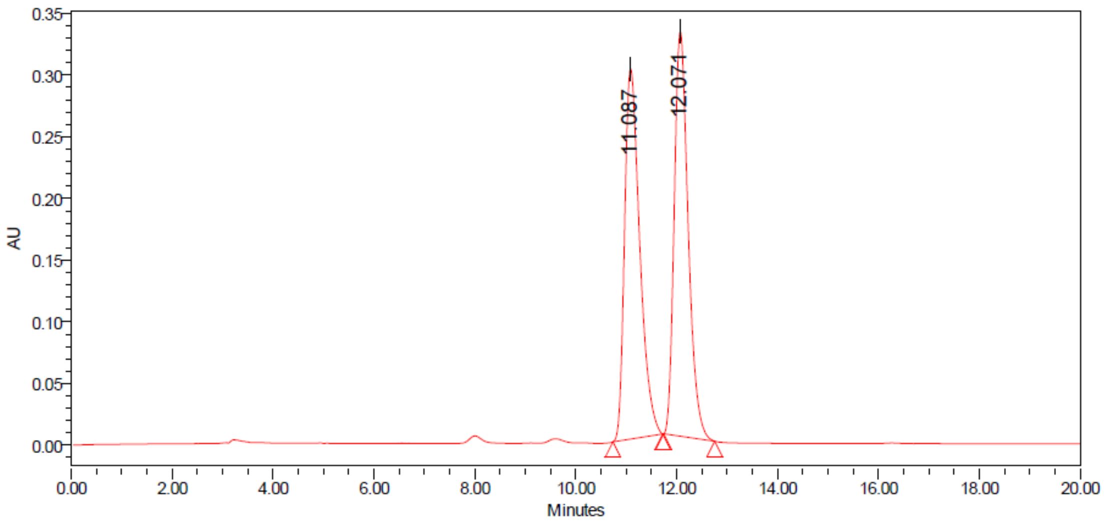

Peak	% Area	Retention Time	Area	Height
1	49.92	11.087	6271700	300296
2	50.08	12.071	6290694	328124
Total	100.00			

Supplementary Figure 66b. Scalemic Chromatogram of Major Diastereomer of **18**.

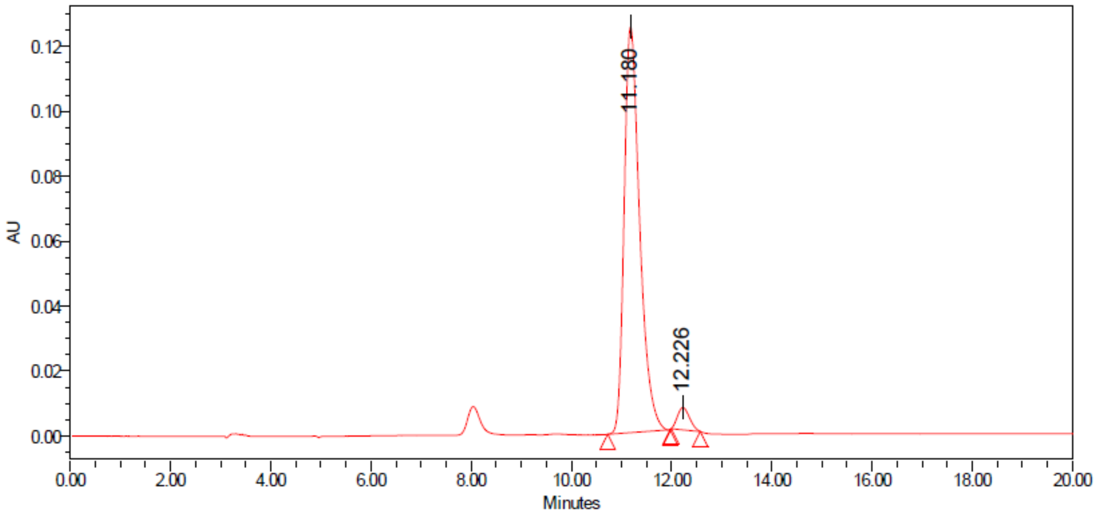

Peak	% Area	Retention Time	Area	Height
1	95.89	11.180	2684924	124948
2	4.11	12.226	115218	6870
Total	100.00			

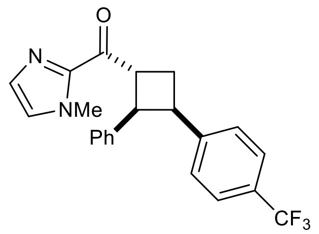

19 – Major Diastereomer
Run Details: HPLC, Daicel CHIRALPAK OD-H,
10.00 µL, gradient 5% to 50% iPrOH/hexanes,
13 minutes, 1 mL/min, 280.0 nm.

Supplementary Figure 67a. Racemic Chromatogram of Major Diastereomer of **19**.

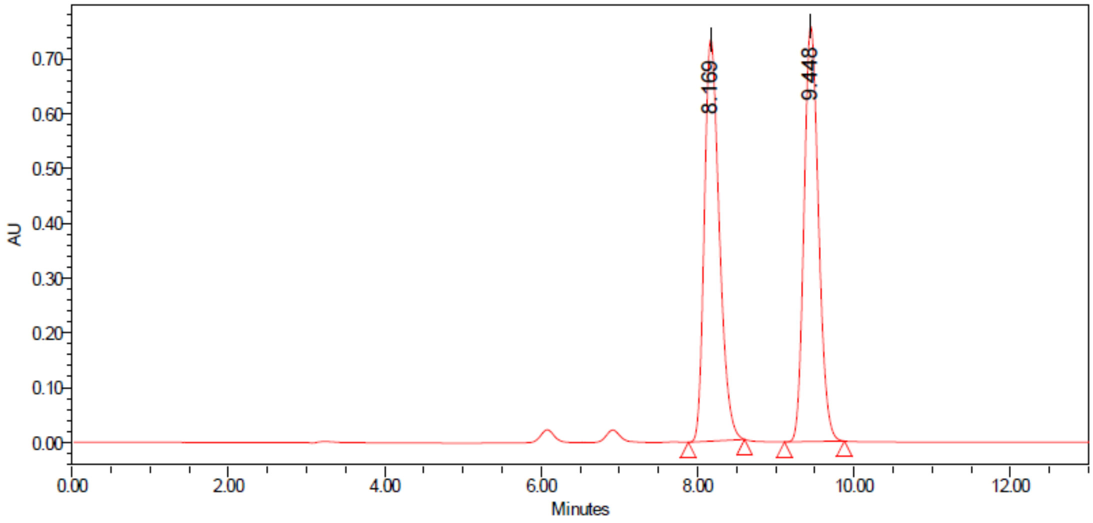

Peak	% Area	Retention Time	Area	Height
1	49.89	8.169	9741011	733077
2	50.11	9.448	9785893	757999
Total	100.00			

Supplementary Figure 67b. Scalemic Chromatogram of Major Diastereomer of **19**.

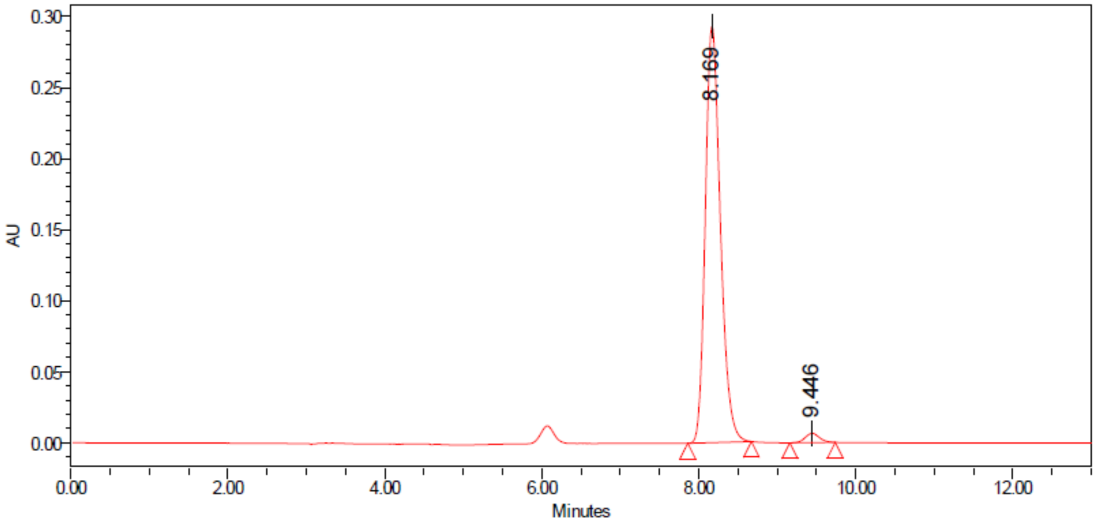

Peak	% Area	Retention Time	Area	Height
1	97.96	8.196	3904779	292916
2	2.04	9.446	81183	6513
Total	100.00			

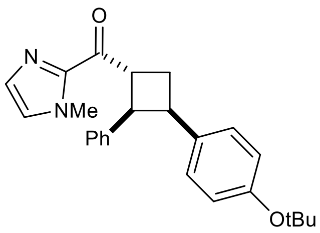

20 – Major Diastereomer
Run Details: HPLC, Daicel CHIRALPAK OD-H,
10.00 µL, gradient 5% to 50% iPrOH/hexanes,
18 minutes, 1 mL/min, 285.0 nm.

Supplementary Figure 68a. Racemic Chromatogram of Major Diastereomer of **20**.

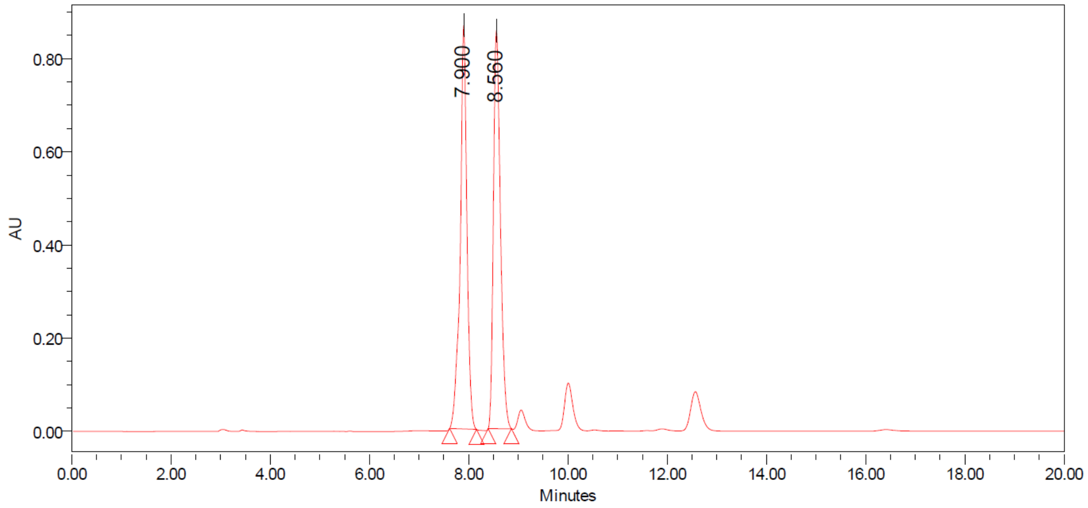

Peak	% Area	Retention Time	Area	Height
1	49.32	7.900	8150154	866166
2	50.68	8.560	8374564	854725
Total	100.00			

Supplementary Figure 68b. Scalemic Chromatogram of Major Diastereomer of **20**.

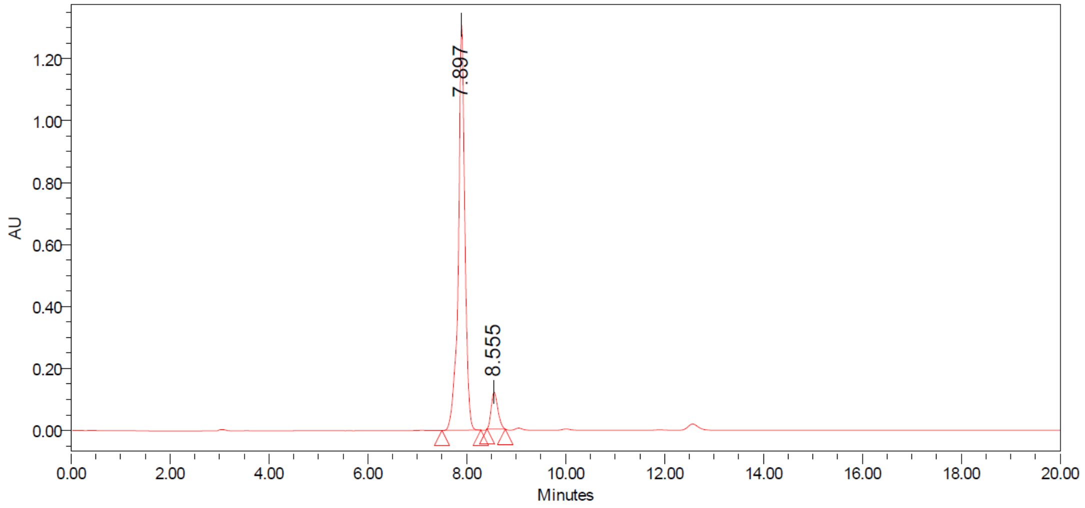

Peak	% Area	Retention Time	Area	Height
1	91.50	7.897	12010713	1308606
2	8.50	8.555	1115558	119100
Total	100.00			

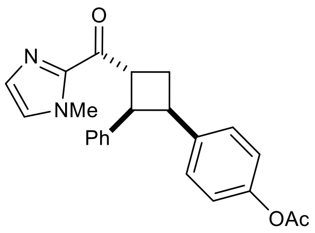

21 – Major Diastereomer
Run Details: HPLC, Daicel CHIRALPAK OD-H,
10.00 µL, gradient 5% to 50% iPrOH/hexanes,
18 minutes, 1 mL/min, 285.0 nm.

Supplementary Figure 69a. Racemic Chromatogram of Major Diastereomer of **21**.

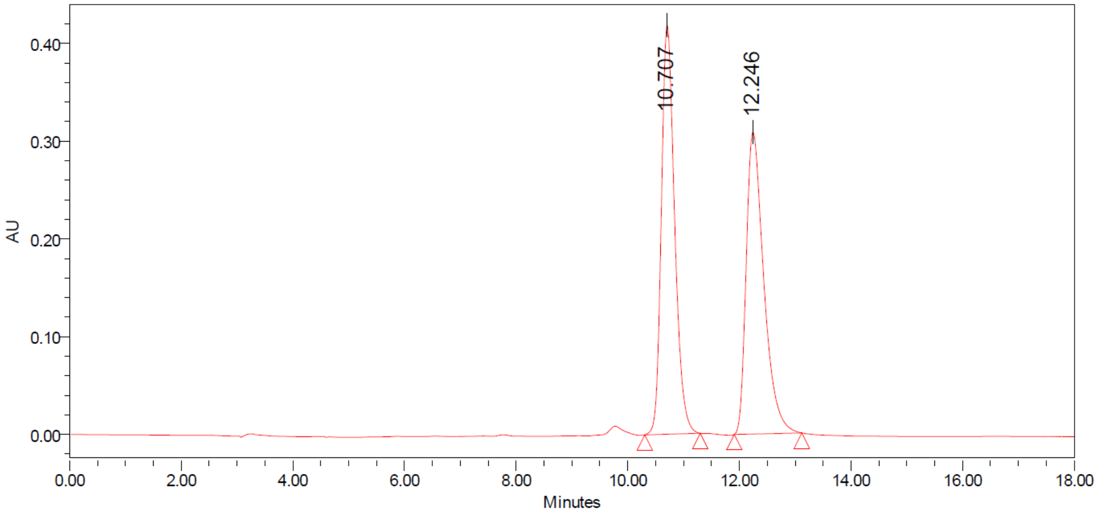

Peak	% Area	Retention Time	Area	Height
1	50.54	10.707	6903222	418287
2	49.46	12.246	6754542	308996
Total	100.00			

Supplementary Figure 69b. Scalemic Chromatogram of Major Diastereomer of **21**.

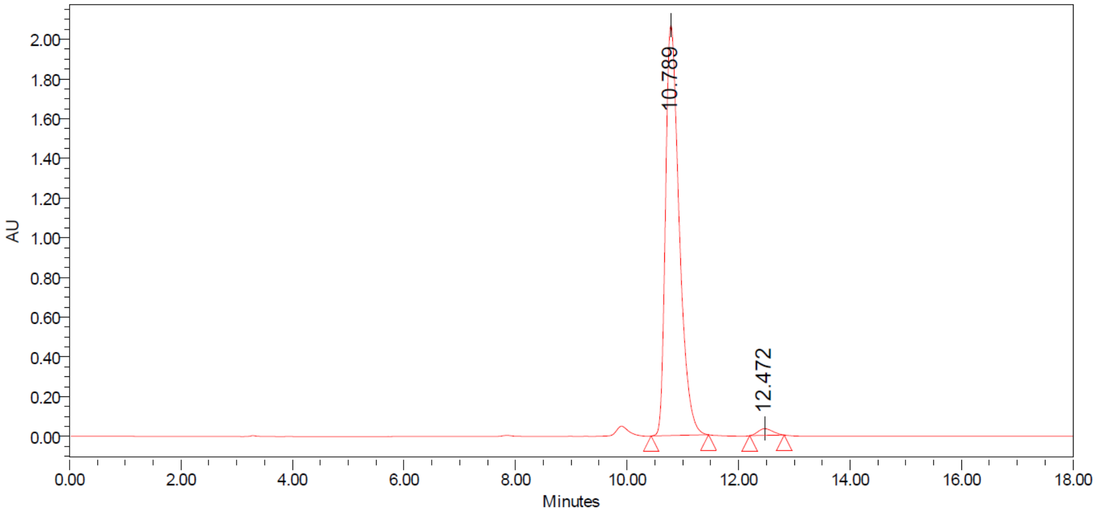

Peak	% Area	Retention Time	Area	Height
1	98.26	10.789	35347991	2066329
2	1.74	12.472	626226	33837
Total	100.00			

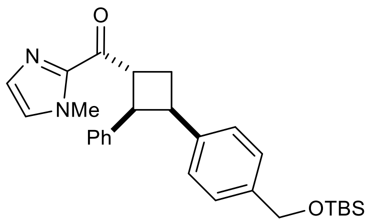

22 – Major Diastereomer
Run Details: HPLC, Daicel CHIRALPAK OD-H,
10.00 µL, gradient 5% to 50% iPrOH/hexanes,
18 minutes, 1 mL/min, 285.0 nm.

Supplementary Figure 70a. Racemic Chromatogram of Major Diastereomer of **22**.

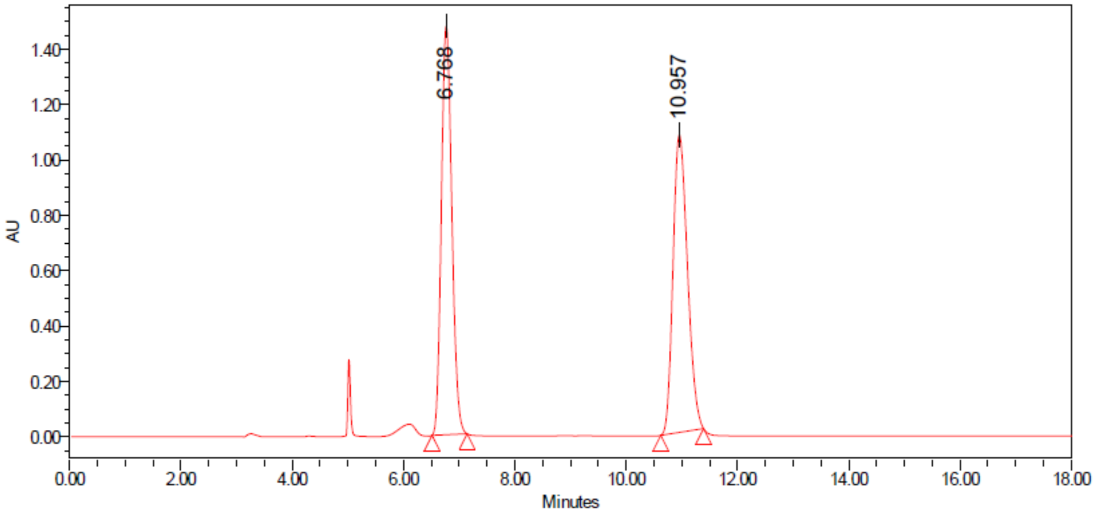

Peak	% Area	Retention Time	Area	Height
1	49.92	6.768	19293809	1476222
2	50.08	10.957	19357563	1074753
Total	100.00			

Supplementary Figure 70b. Scalemic Chromatogram of Major Diastereomer of **22**.

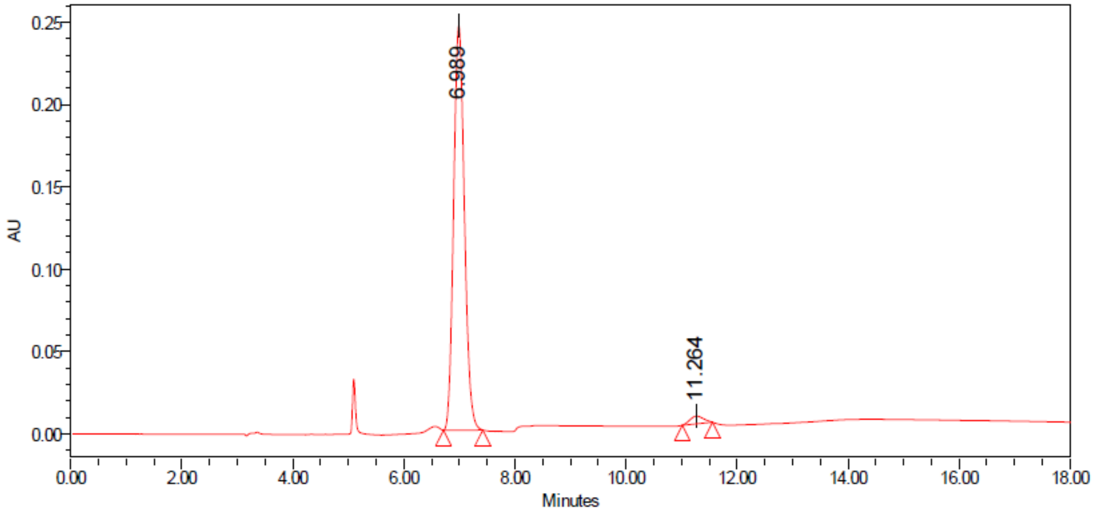

Peak	% Area	Retention Time	Area	Height
1	97.65	6.989	3307673	245547
2	2.35	11.264	79739	4704
Total	100.00			

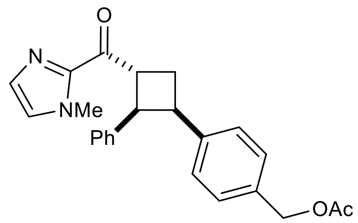

23 – Major Diastereomer
Run Details: HPLC, Daicel CHIRALPAK OD-H, 10.00 µL, gradient 5% to 50% iPrOH/hexanes, 28 minutes, 1 mL/min, 280.0 nm.

Supplementary Figure 71a. Racemic Chromatogram of Major Diastereomer of **23**.

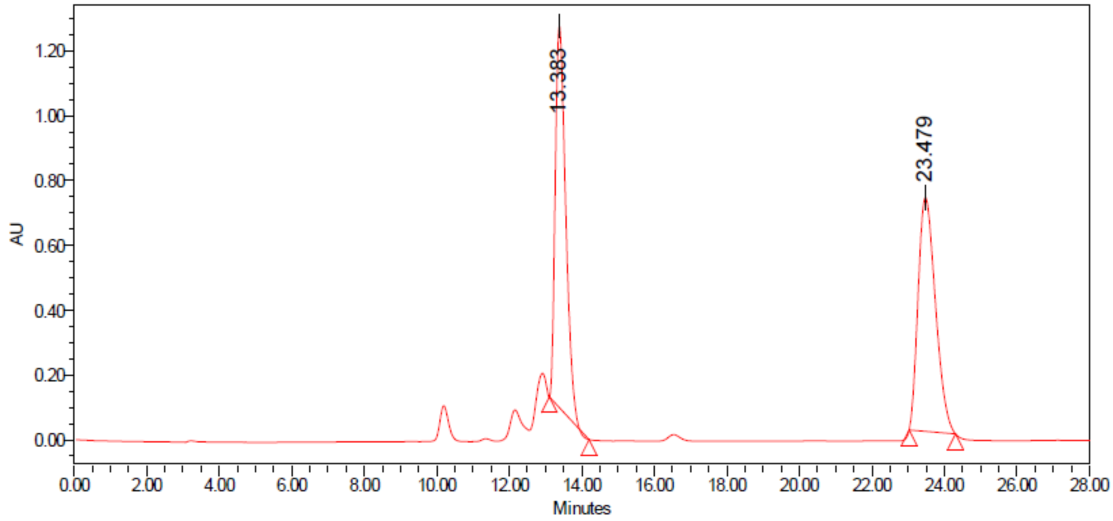

Peak	% Area	Retention Time	Area	Height
1	49.66	13.383	23641117	1176330
2	50.34	23.479	23961968	721251
Total	100.00			

Supplementary Figure 71b. Scalemic Chromatogram of Major Diastereomer of **23**.

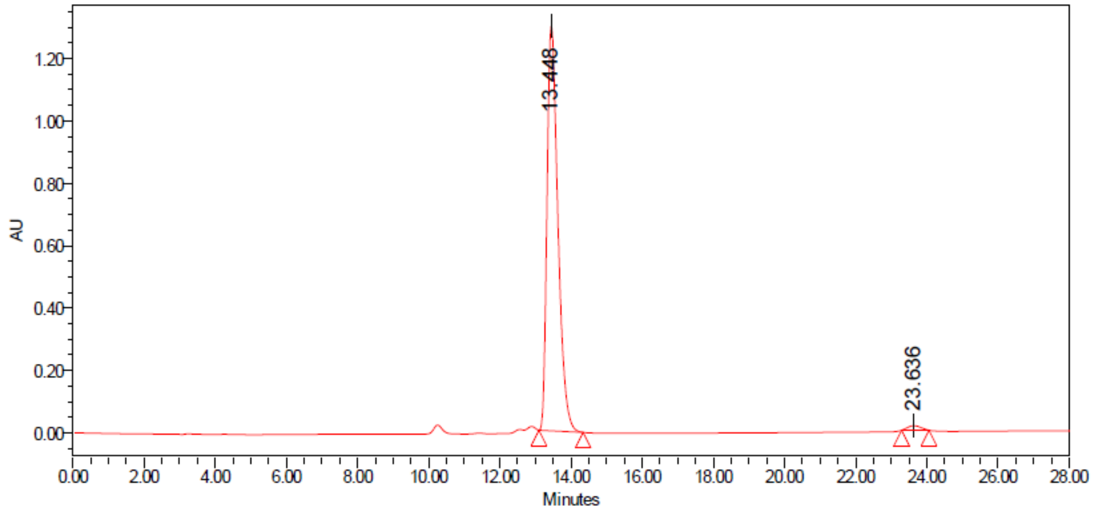

Peak	% Area	Retention Time	Area	Height
1	98.67	13.448	27623051	1298019
2	1.33	23.636	373592	14428
Total	100.00			

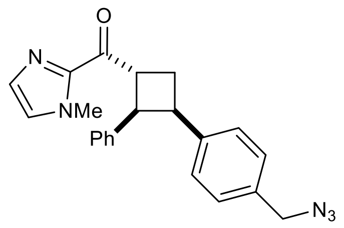

24 – Major Diastereomer
Run Details: HPLC, Daicel CHIRALPAK AD,
10.00 µL, gradient 5% to 50% iPrOH/hexanes,
18 minutes, 1 mL/min, 280.0 nm.

Supplementary Figure 72a. Racemic Chromatogram of Major Diastereomer of **24**.

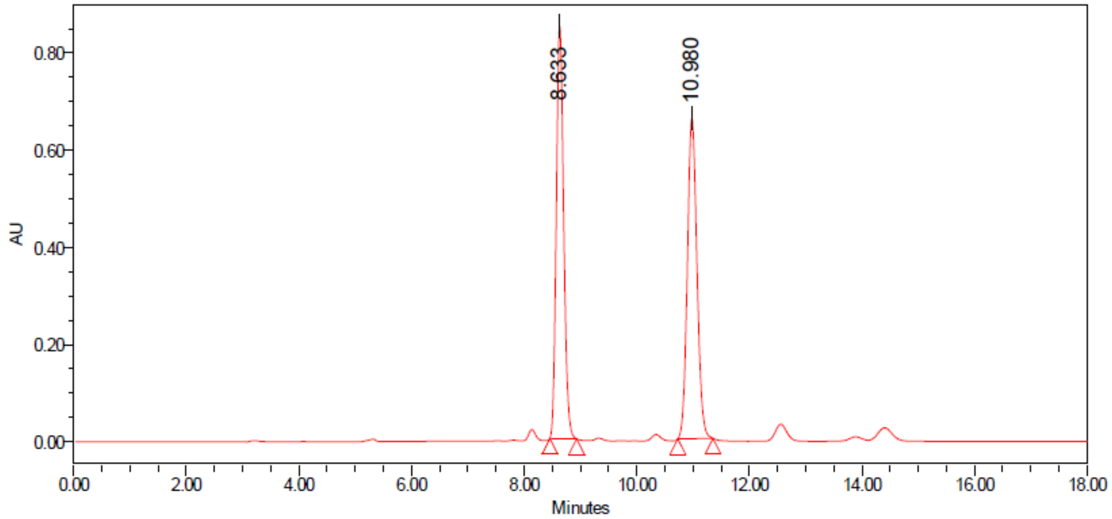

Peak	% Area	Retention Time	Area	Height
1	49.70	8.633	7691352	848642
2	50.30	10.980	7784586	659174
Total	100.00			

Supplementary Figure 72b. Scalemic Chromatogram of Major Diastereomer of **24**.

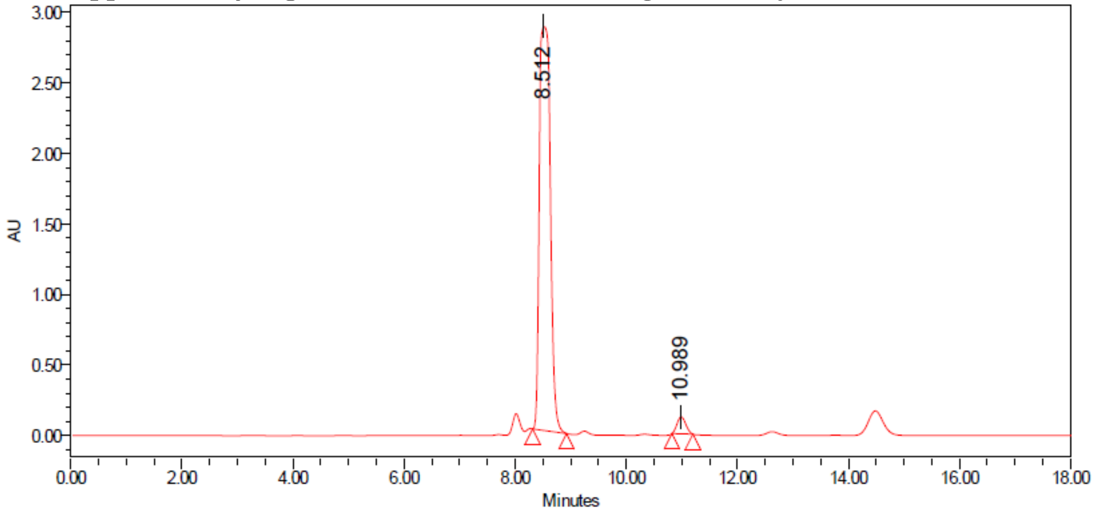

Peak	% Area	Retention Time	Area	Height
1	96.78	8.512	2865539	2865539
2	3.22	10.989	119972	119972
Total	100.00			

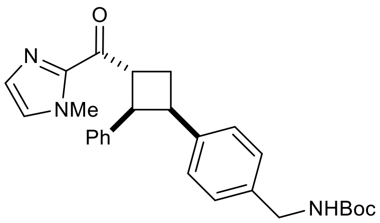

25 – Major Diastereomer
Run Details: HPLC, Daicel CHIRALPAK OD-H,
10.00 µL, gradient 5% to 50% iPrOH/hexanes,
28 minutes, 1 mL/min, 285.0 nm.

Supplementary Figure 73a. Racemic Chromatogram of Major Diastereomer of **25**.

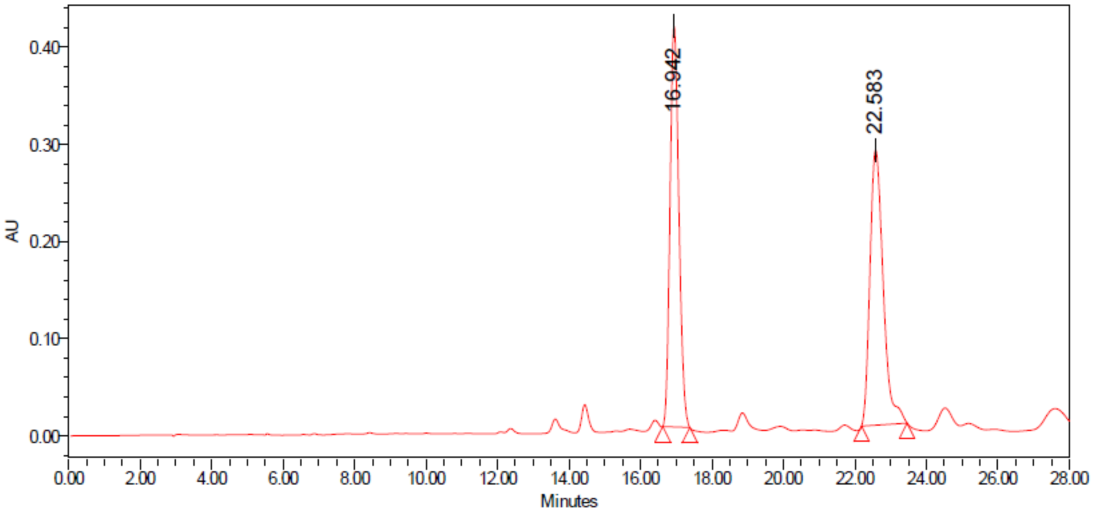

Peak	% Area	Retention Time	Area	Height
1	49.25	16.942	7089598	412447
2	50.75	22.583	7304797	283175
Total	100.00			

Supplementary Figure 73b. Scalemic Chromatogram of Major Diastereomer of **25**.

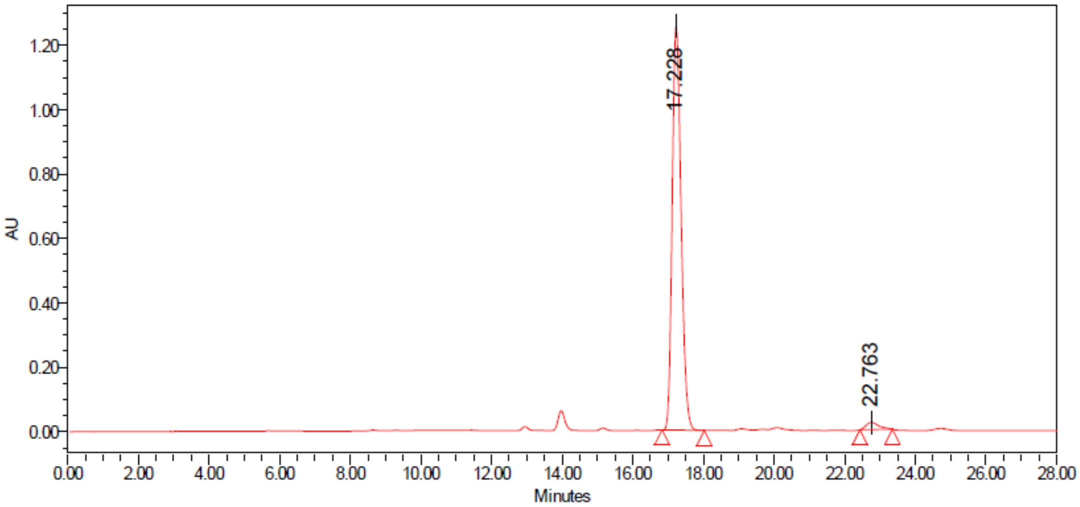

Peak	% Area	Retention Time	Area	Height
1	97.45	17.228	21877872	1254062
2	2.55	22.763	573270	22464
Total	100.00			

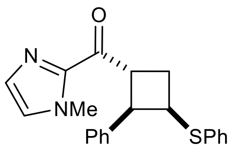

26 – Major Diastereomer
Run Details: HPLC, Daicel CHIRALPAK AD, 10.00 µL, gradient 5% to 50% iPrOH/hexanes, 18 minutes, 1 mL/min, 285.0 nm.

Supplementary Figure 74a. Racemic Chromatogram of Major Diastereomer of **26**.

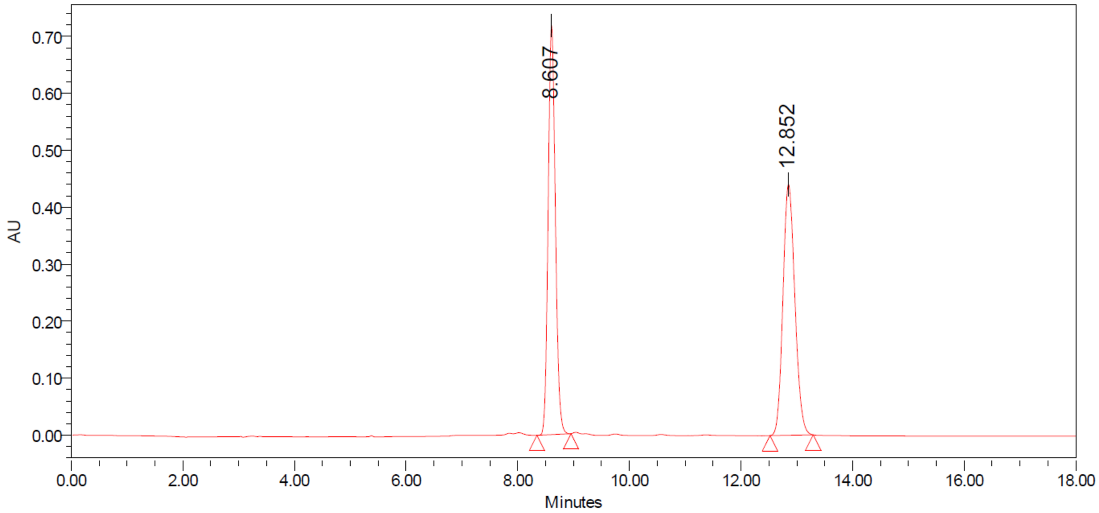

Peak	% Area	Retention Time	Area	Height
1	49.62	8.607	6435841	718229
2	50.38	12.852	6534797	440270
Total	100.00			

Supplementary Figure 74b. Scalemic Chromatogram of Major Diastereomer of **26**.

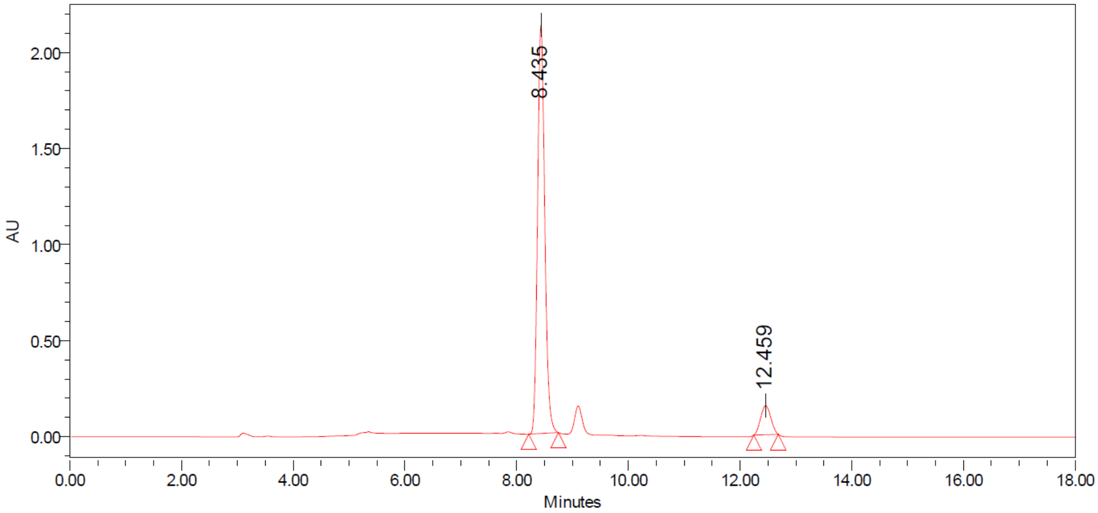

Peak	% Area	Retention Time	Area	Height
1	90.84	8.435	19057924	2126287
2	9.16	12.459	1921213	151508
Total	100.00			

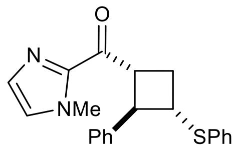

26 – Minor Diastereomer
Run Details: HPLC, Daicel CHIRALPAK OD-H,
10.00 µL, gradient 5% to 50% iPrOH/hexanes,
18 minutes, 1 mL/min, 285.0 nm.

Supplementary Figure 75a. Racemic Chromatogram of Minor Diastereomer of **26**.

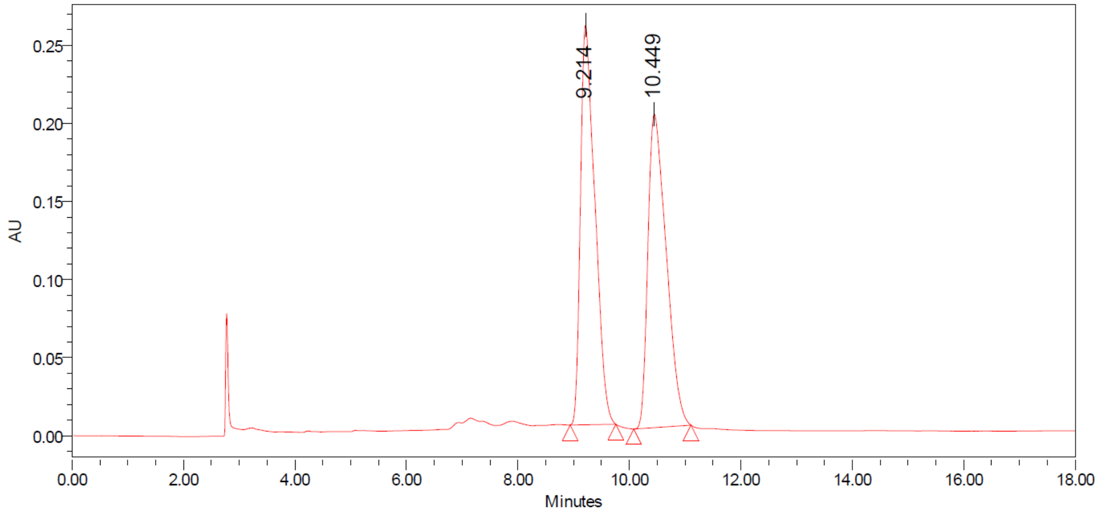

Peak	% Area	Retention Time	Area	Height
1	50.02	9.214	4597067	255909
2	49.98	10.449	4593163	200910
Total	100.00			

Supplementary Figure 75b. Scalemic Chromatogram of Minor Diastereomer of **26**.

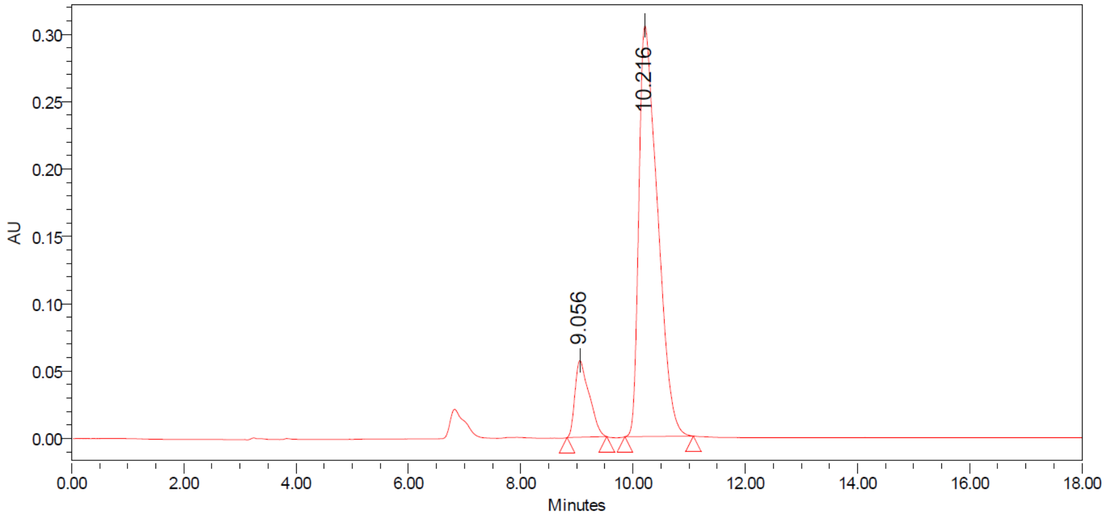

Peak	% Area	Retention Time	Area	Height
1	12.45	9.056	999244	56884
2	87.55	10.216	7025160	305015
Total	100.00			

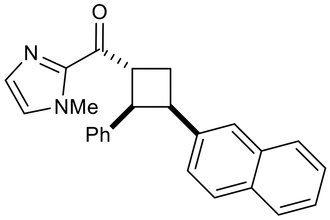

27 – Major Diastereomer
Run Details: HPLC, Daicel CHIRALPAK AD-H,
10.00 µL, gradient 5% to 50% iPrOH/hexanes,
13 minutes, 1 mL/min, 280.0 nm.

Supplementary Figure 76a. Racemic Chromatogram of Major Diastereomer of **27**.

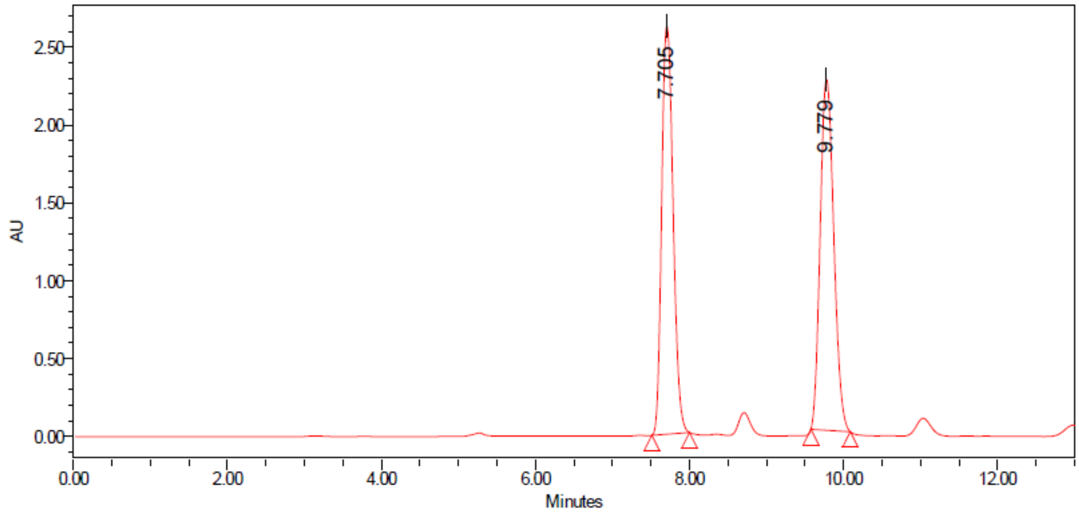

Peak	% Area	Retention Time	Area	Height
1	49.38	7.705	26951400	2618716
2	50.62	9.779	27633264	2251612
Total	100.00			

Supplementary Figure 76b. Scalemic Chromatogram of Major Diastereomer of **27**.

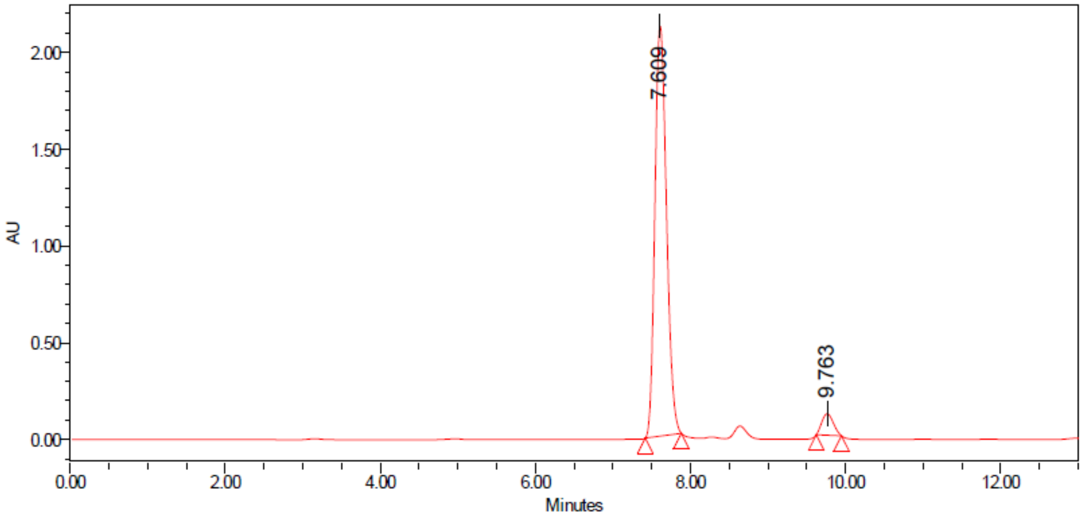

Peak	% Area	Retention Time	Area	Height
1	95.05	7.609	21961022	2117815
2	4.95	9.763	1143222	110920
Total	100.00			

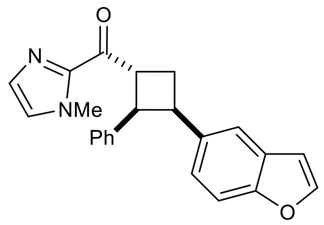

28 – Major Diastereomer
Run Details: HPLC, Daicel CHIRALPAK OD-H,
10.00 µL, gradient 5% to 50% iPrOH/hexanes,
18 minutes, 1 mL/min, 285.0 nm.

Supplementary Figure 77a. Racemic Chromatogram of Major Diastereomer of **28**.

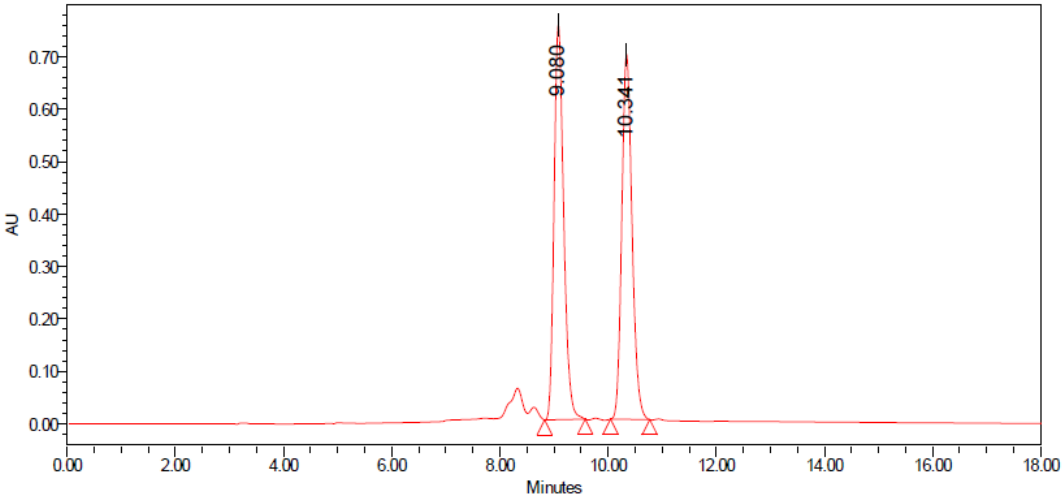

Peak	% Area	Retention Time	Area	Height
1	50.29	9.080	9278212	752544
2	49.71	10.341	9171396	696002
Total	100.00			

Supplementary Figure 77b. Scalemic Chromatogram of Major Diastereomer of **28**.

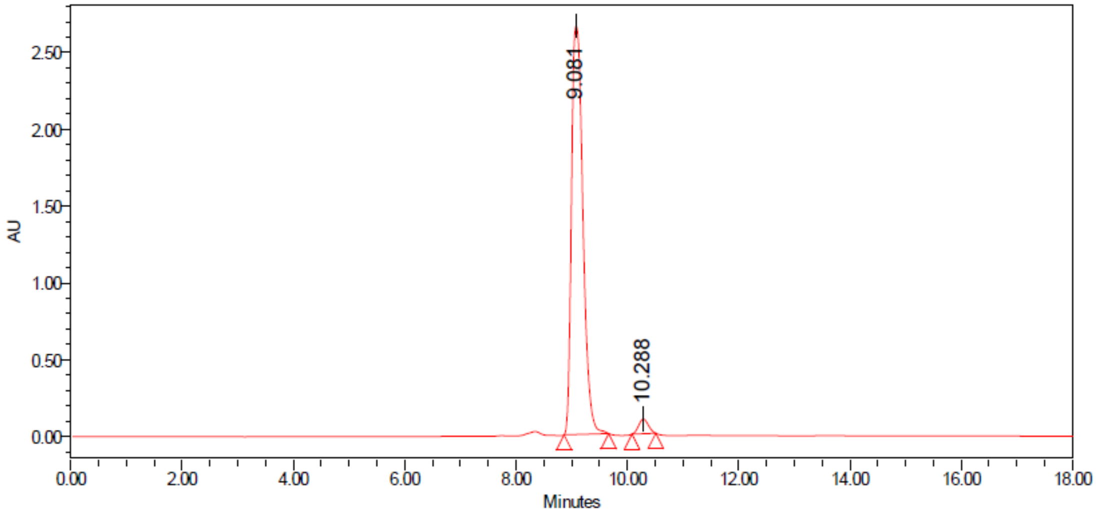

Peak	% Area	Retention Time	Area	Height
1	97.02	9.081	37854855	2658322
2	2.98	10.288	1161872	95801
Total	100.00			

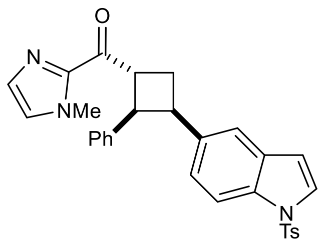

29 – Major Diastereomer
Run Details: HPLC, Daicel CHIRALPAK AS-H,
10.00 µL, gradient 5% to 50% iPrOH/hexanes,
28 minutes, 1 mL/min, 285.0 nm.

Supplementary Figure 78a. Racemic Chromatogram of Major Diastereomer of **29**.

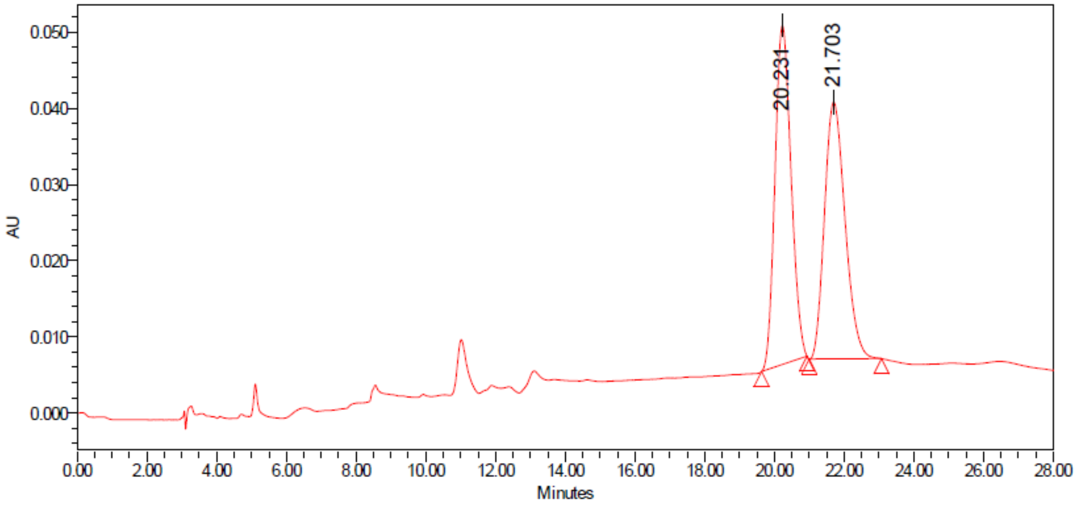

Peak	% Area	Retention Time	Area	Height
1	50.84	20.231	1425708	44483
2	49.16	21.703	1378662	33699
Total	100.00			

Supplementary Figure 78b. Scalemic Chromatogram of Major Diastereomer of **29**.

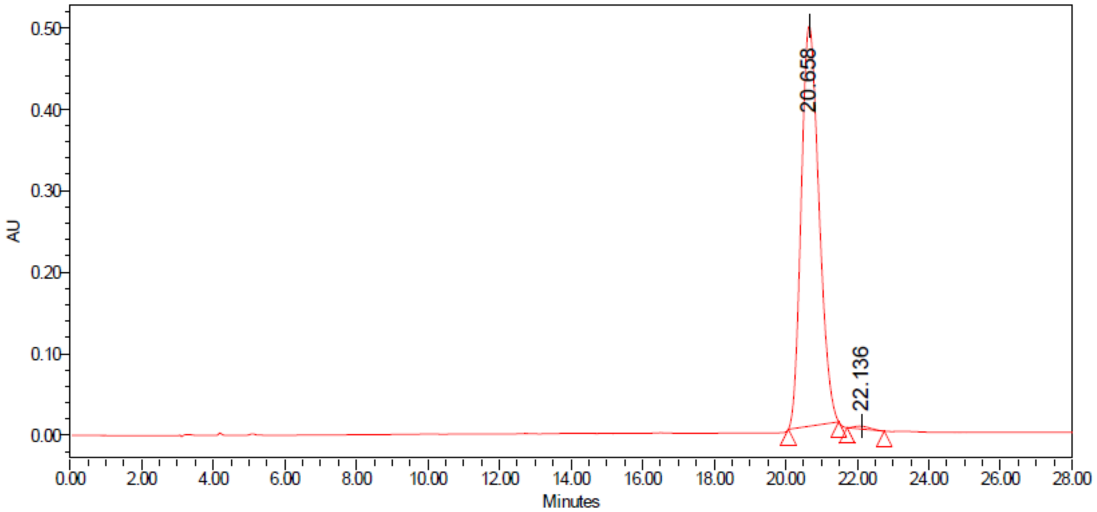

Peak	% Area	Retention Time	Area	Height
1	99.36	20.658	16949841	490473
2	2.45	22.136	108667	3556
Total	100.00			

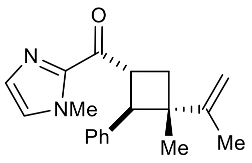

30 – Major Diastereomer
Run Details: HPLC, Daicel CHIRALPAK AD,
10.00 µL, gradient 5% to 50% iPrOH/hexanes,
18 minutes, 1 mL/min, 295.0 nm.

Supplementary Figure 79a. Racemic Chromatogram of Major Diastereomer of **30**.

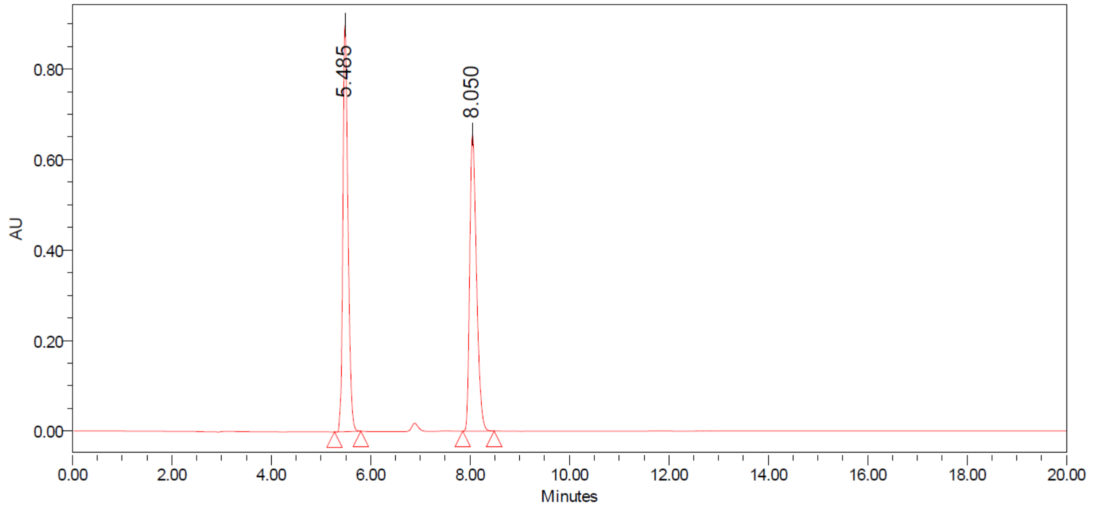

Peak	% Area	Retention Time	Area	Height
1	50.53	5.485	6398300	898523
2	49.47	8.050	6264920	656586
Total	100.00			

Supplementary Figure 79b. Scalemic Chromatogram of Major Diastereomer of **30**.

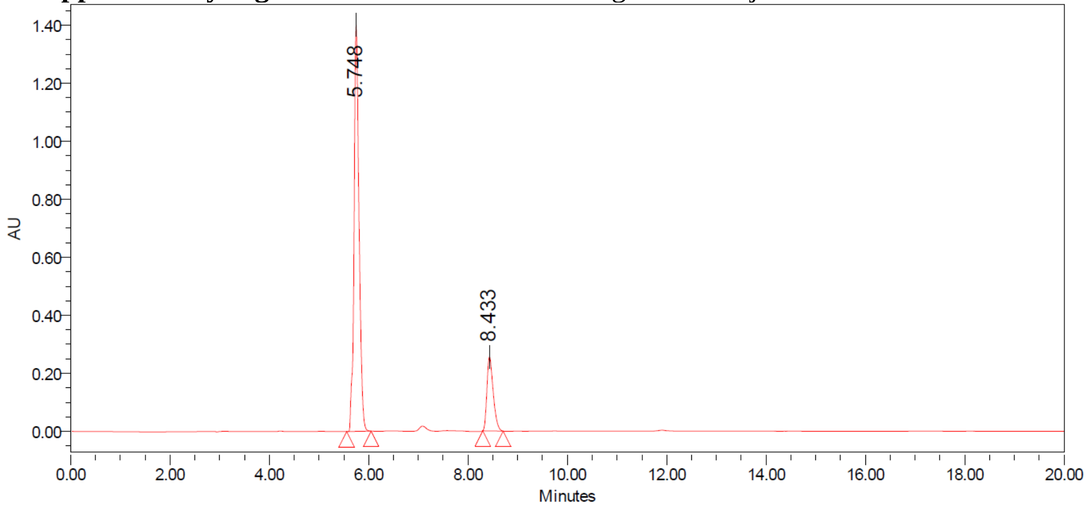

Peak	% Area	Retention Time	Area	Height
1	81.38	5.748	9802518	1401129
2	18.62	8.433	2243334	255768
Total	100.00			

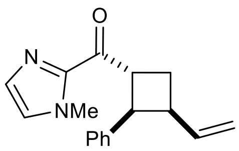

31 – Major Diastereomer
Run Details: HPLC, Daicel CHIRALPAK AD, 10.00 µL, gradient 5% to 50% iPrOH/hexanes, 18 minutes, 1 mL/min, 295.0 nm.

Supplementary Figure 80a. Racemic Chromatogram of Major Diastereomer of **31**.

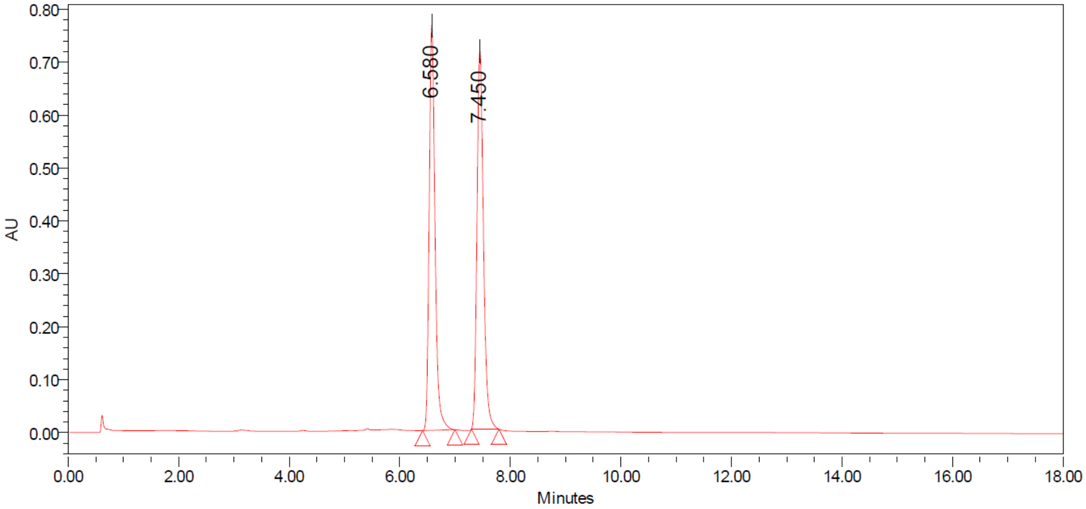

Peak	% Area	Retention Time	Area	Height
1	49.18	6.580	5619294	767670
2	50.82	7.450	5806639	714161
Total	100.00			

Supplementary Figure 80b. Scalemic Chromatogram of Major Diastereomer of **31**.

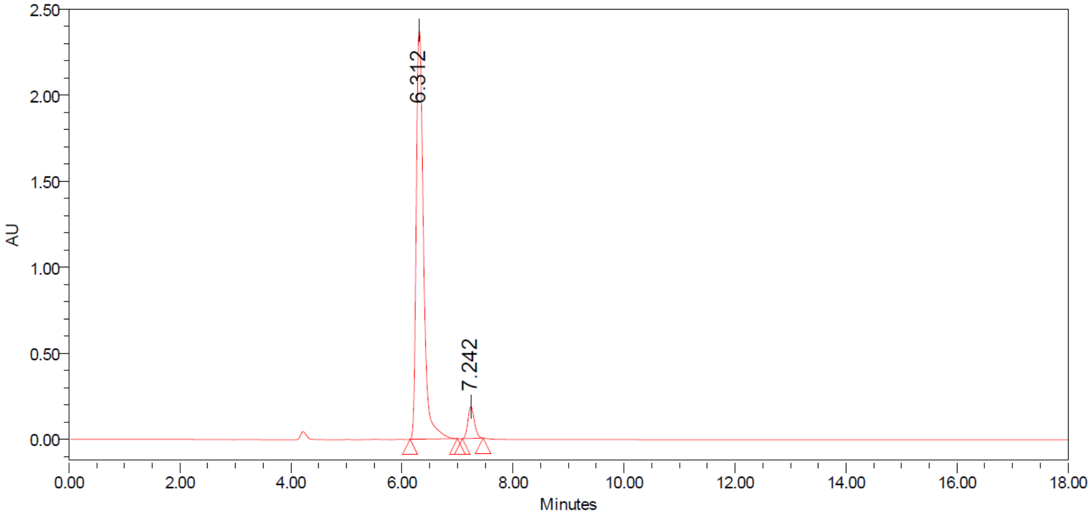

Peak	% Area	Retention Time	Area	Height
1	93.48	6.312	21698128	2379222
2	6.52	7.242	1513611	185695
Total	100.00			

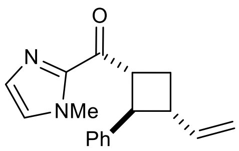

31 – Minor Diastereomer
Run Details: HPLC, Daicel CHIRALPAK AD, 10.00 µL, gradient 5% to 50% iPrOH/hexanes, 18 minutes, 1 mL/min, 295.0 nm.

Supplementary Figure 81a. Racemic Chromatogram of Minor Diastereomer of **31**.

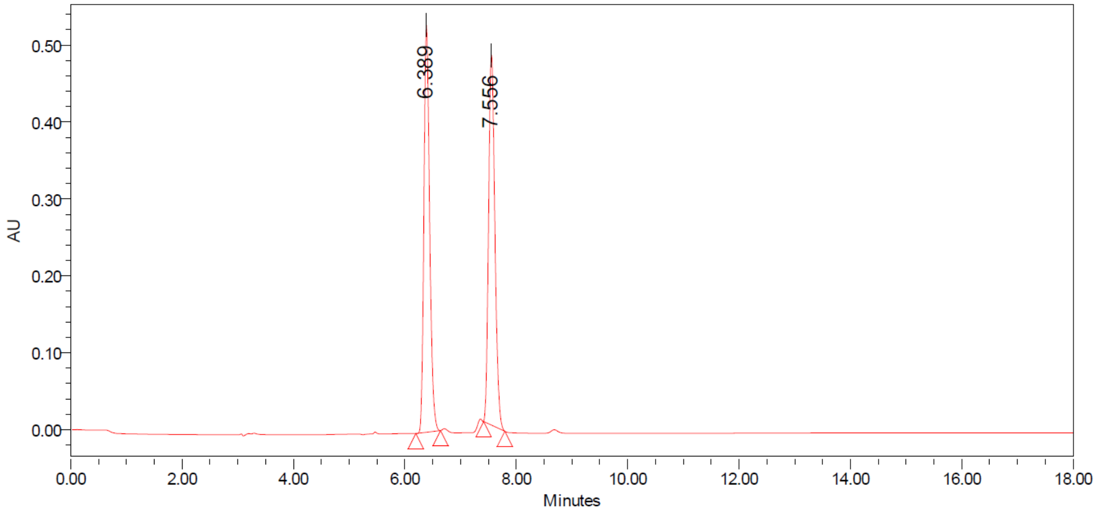

Peak	% Area	Retention Time	Area	Height
1	49.99	6.389	3780701	529097
2	50.01	7.556	3782575	481054
Total	100.00			

Supplementary Figure 81b. Scalemic Chromatogram of Minor Diastereomer of **31**.

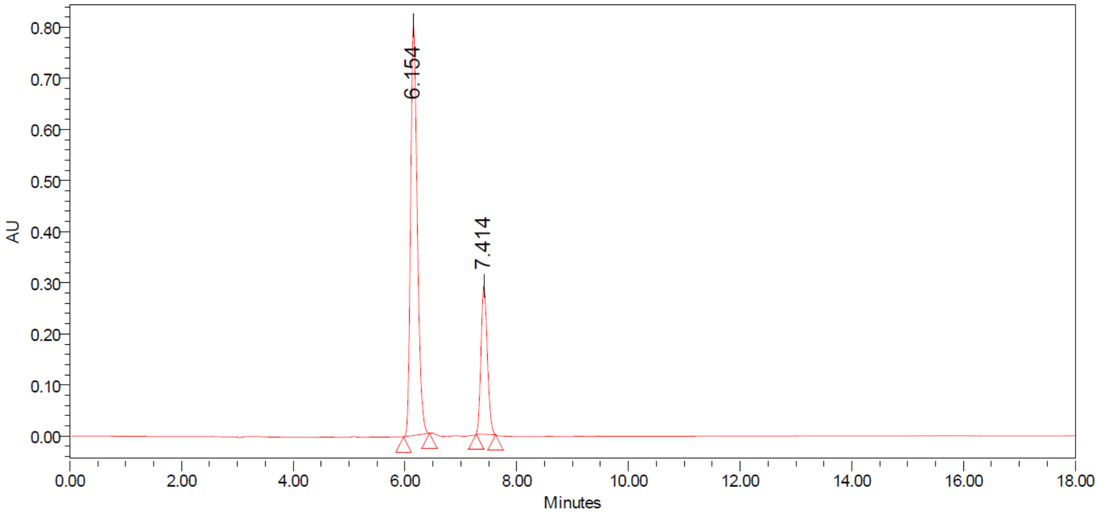

Peak	% Area	Retention Time	Area	Height
1	74.35	6.154	6640710	802202
2	26.65	7.414	2290774	290511
Total	100.00			

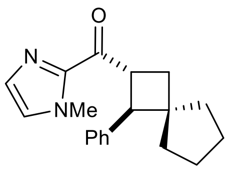

34 – Major Diastereomer
Run Details: HPLC, Diacel CHIRALPAK OD-H,
10.00 µL, gradient 5% to 50% iPrOH/hexanes,
18 minutes, 1 mL/min, 295.0 nm.

Supplementary Figure 82a. Racemic Chromatogram of Major Diastereomer of **34**.

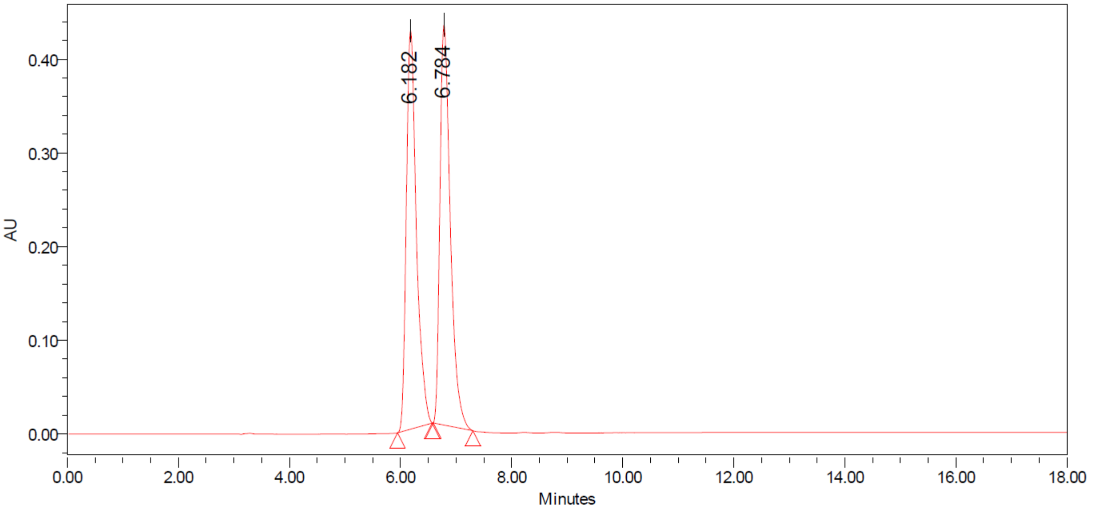

Peak	% Area	Retention Time	Area	Height
1	49.10	6.182	5361808	425302
2	50.90	6.784	5559201	427104
Total	100.00			

Supplementary Figure 82b. Scalemic Chromatogram of Major Diastereomer of **34**.

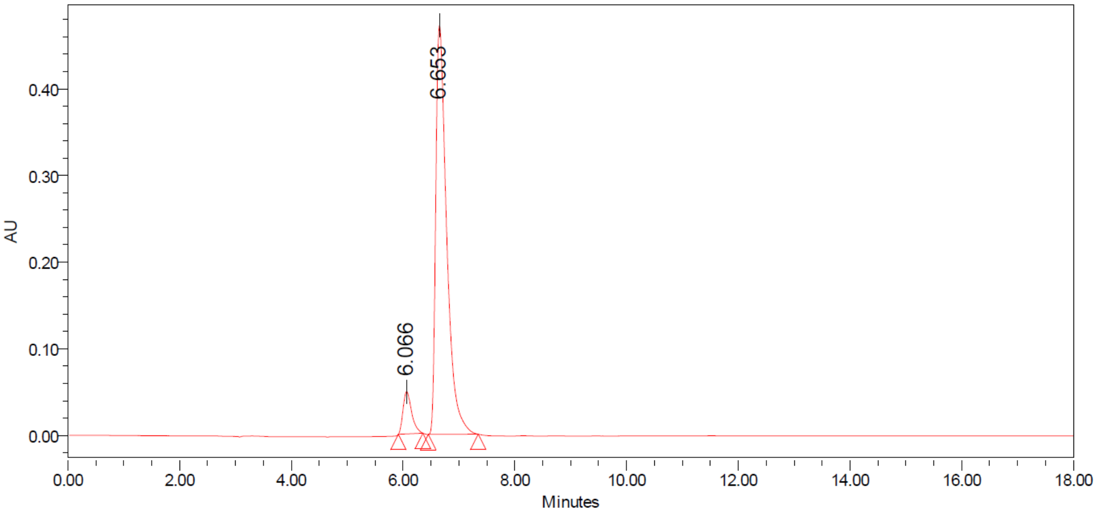

Peak	% Area	Retention Time	Area	Height
1	7.52	6.066	519995	48805
2	92.48	6.653	6395904	471931
Total	100.00			

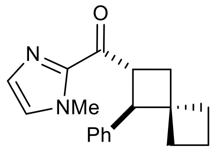

35
Run Details: HPLC, Daicel CHIRALPAK AD, 10.00 μL, gradient 5% to 50% iPrOH/hexanes, 18 minutes, 1 mL/min, 285.0 nm.

Supplementary Figure 83a. Racemic Chromatogram of **35**.

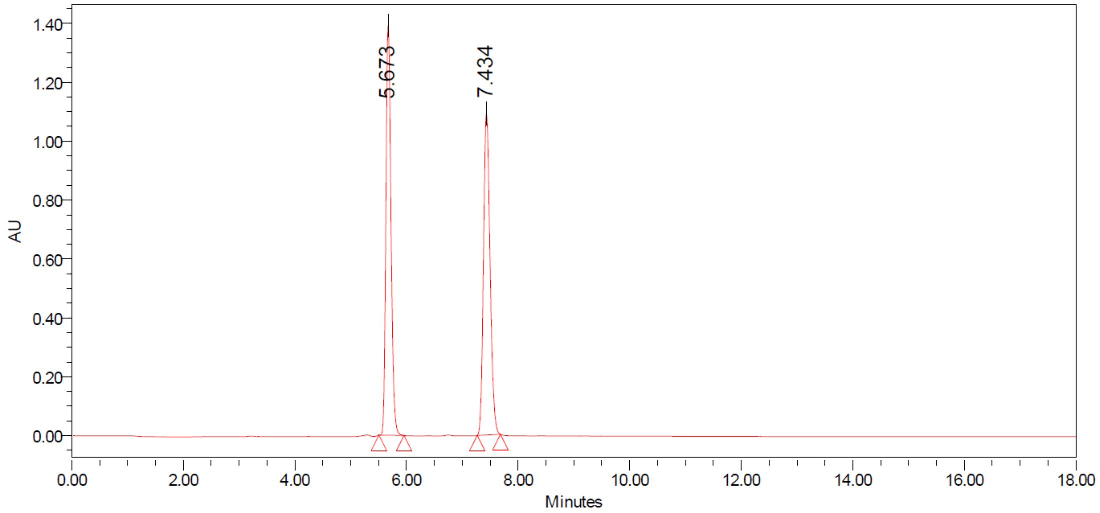

Peak	% Area	Retention Time	Area	Height
1	49.53	5.673	8797392	1391958
2	50.47	7.434	8962578	1091598
Total	100.00			

Supplementary Figure 83b. Scalemic Chromatogram of **35**.

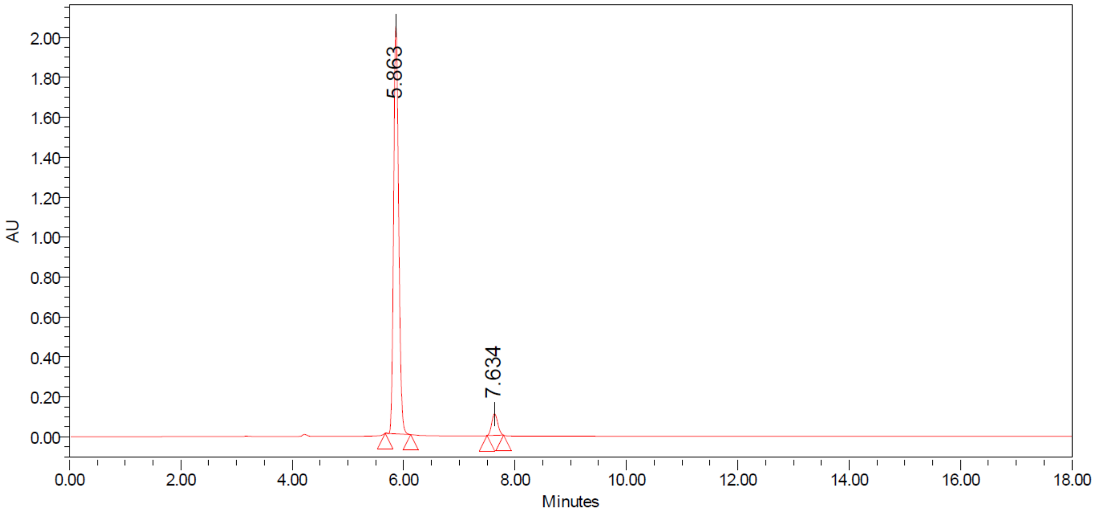

Peak	% Area	Retention Time	Area	Height
1	93.99	5.863	12847379	2044953
2	6.01	7.634	820925	108421
Total	100.00			

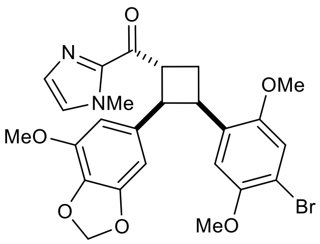

36 – Major Diastereomer
Run Details: HPLC, Daicel CHIRALPAK AD,
10.00 µL, gradient 5% to 50% iPrOH/hexanes,
20 minutes, 1 mL/min, 285.0 nm.

Supplementary Figure 84a. Racemic Chromatogram of Major Diastereomer of **36**.

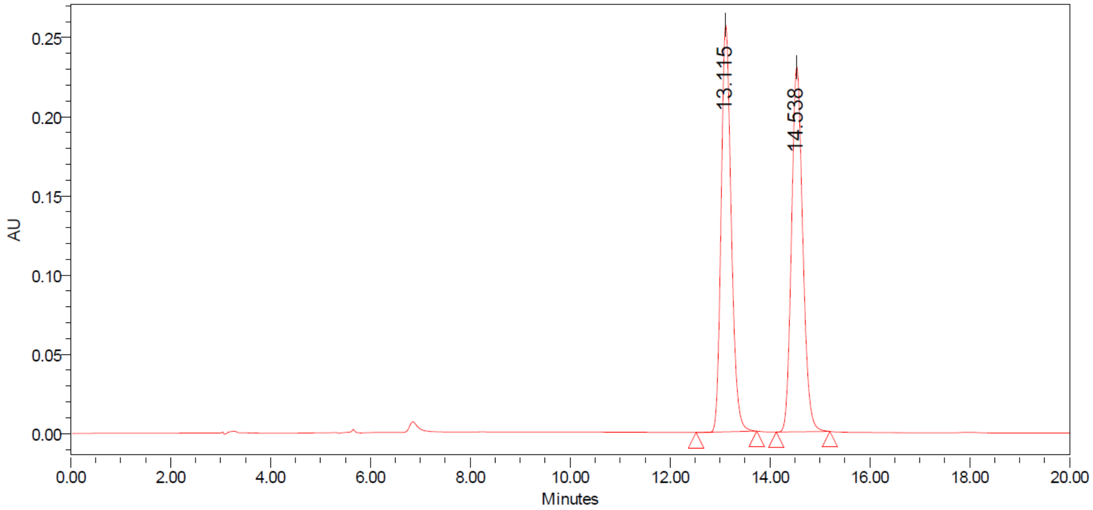

Peak	% Area	Retention Time	Area	Height
1	49.98	13.115	3596281	256807
2	50.02	14.538	3599837	230267
Total	100.00			

Supplementary Figure 84b. Scalemic Chromatogram of Major Diastereomer of **36**.

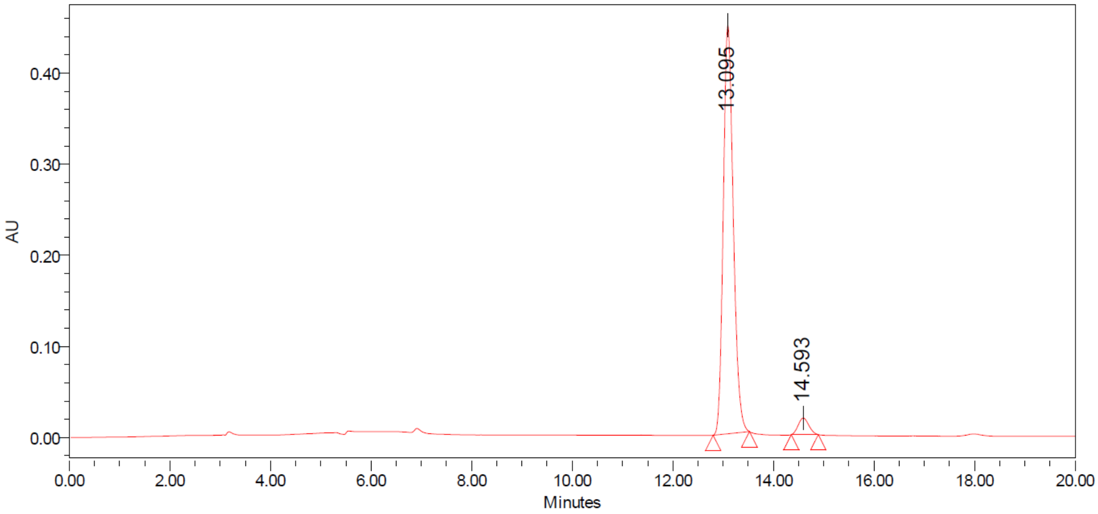

Peak	% Area	Retention Time	Area	Height
1	95.88	13.095	6183232	448302
2	4.12	14.593	265927	18169
Total	100.00			

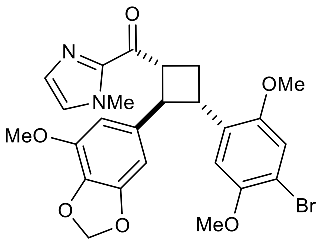

36 – Minor Diastereomer
Run Details: HPLC, Daicel CHIRALPAK AS-H,
10.00 µL, gradient 5% to 30% EtOH/hexanes,
35 minutes, 1 mL/min, 285.0 nm.

Supplementary Figure 85a. Racemic Chromatogram of Minor Diastereomer of **36**.

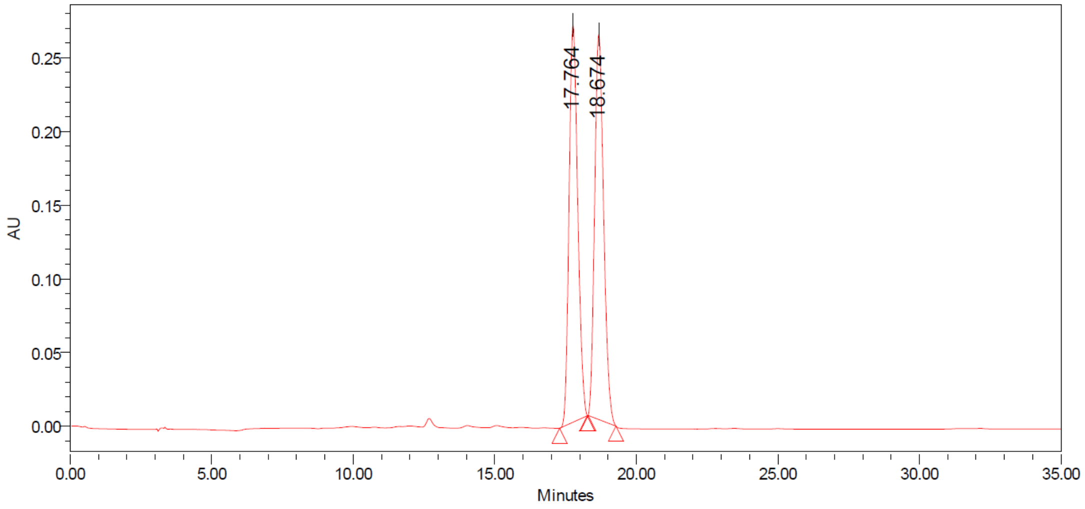

Peak	% Area	Retention Time	Area	Height
1	49.86	17.764	5725848	269376
2	50.14	18.674	5758071	261335
Total	100.00			

Supplementary Figure 85b. Scalemic Chromatogram of Minor Diastereomer of **36**.

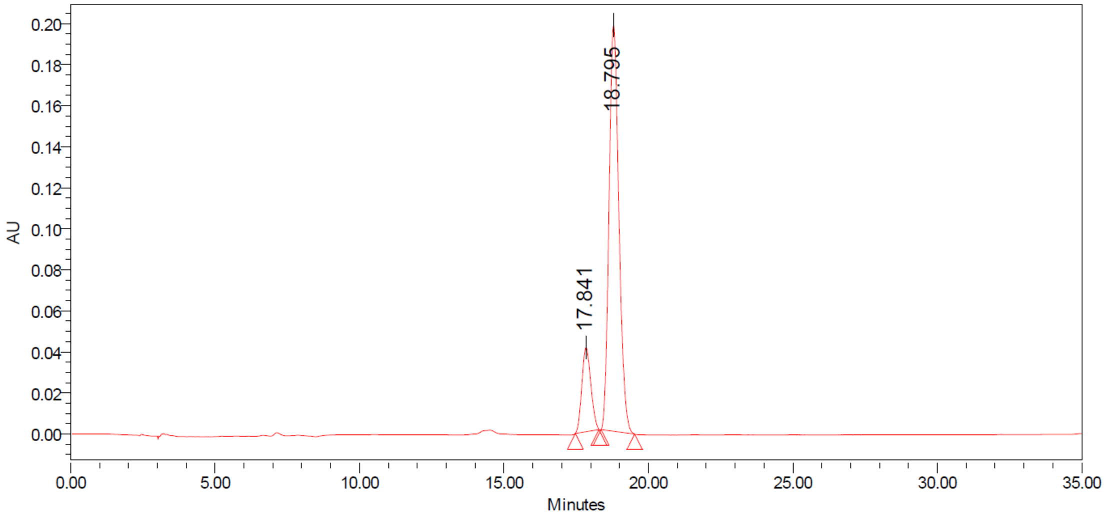

Peak	% Area	Retention Time	Area	Height
1	15.92	17.841	885618	41061
2	84.08	18.795	4678118	197836
Total	100.00			

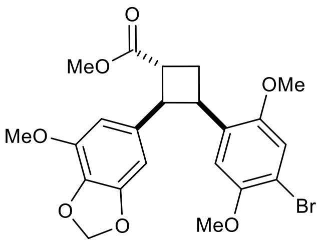

37
Run Details: HPLC, Daicel CHIRALPAK AD, 10.00 µL, gradient 5% to 50% iPrOH/hexanes, 20 minutes, 1 mL/min, 285.0 nm.

Supplementary Figure 86a. Racemic Chromatogram of **37**.

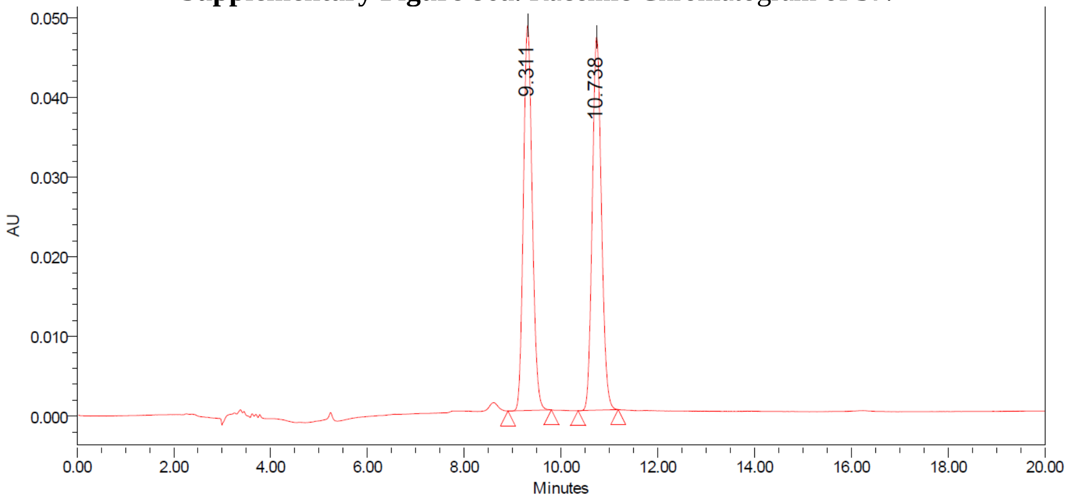

Peak	% Area	Retention Time	Area	Height
1	49.86	9.311	611954	48313
2	50.14	10.738	615302	46848
Total	100.00			

Supplementary Figure 86b. Scalemic Chromatogram of **37**.

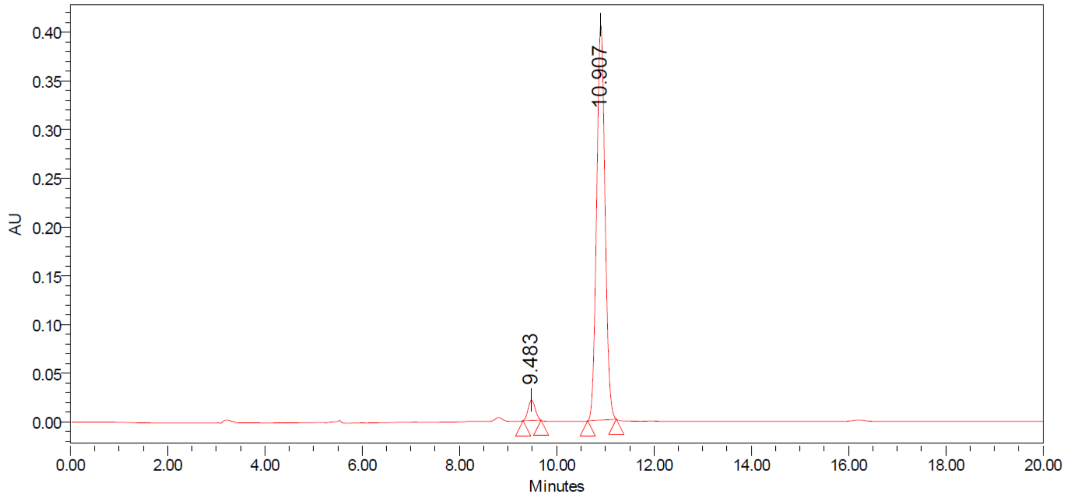

Peak	% Area	Retention Time	Area	Height
1	4.20	9.483	212731	20891
2	95.80	10.907	4848368	405365
Total	100.00			

Supplementary References

1. Pangborn, A. B., Giardello, M. A., Grubbs, R. H., Rosen, R. K. & Timmers, F. J. Safe and convenient procedure for solvent purification. *Organometallics* **15,** 1518–1520 (1996).
2. Uraguchi, D. & Terada, M. Chiral Brønsted acid-catalyzed direct Mannich reactions via electrophilic activation. *J. Am. Chem. Soc.* **126,** 5356–5357 (2004).
3. Nakashima, D. & Yamamoto, H. Design of *N*-Triflyl phosphoramide as a strong chiral Brønsted acid and its application to asymmetric Diels–Alder reaction. *J. Am. Chem. Soc.* **128,** 9626–9627 (2006).
4. Chong, J. M., Shen, L. & Taylor, N. J. Asymmetric conjugate addition of alkynylboronates to enones. *J. Am. Chem. Soc.* **122,** 1822–1823 (2000).
5. Ooi, T., Kameda, M. & Maruoka, K. Design of *N*-spiro C_2-symmetric chiral quaternary ammonium bromides as novel chiral phase-transfer catalysts: synthesis and application to practical asymmetric synthesis of α-amino acids. *J. Am. Chem. Soc.* **125,** 5139–5151 (2003).
6. Ahmed, I. & Clark, D. A. Rapid synthesis of 3,3' bis-arylated BINOL derivatives using a C–H borylation *in situ* Suzuki–Miyaura coupling sequence. *Org. Lett.* **16,** 4332–4335 (2014).
7. Tolstikova, L. L., Bel'skikh, A. V. & Shainyan, B. A. The reaction of *N*-sulfinyltrifluoromethansulfonamide with triethylphosphate and triethylphosphite. *Russ. J. Gen. Chem.* **80,** 1258–1262 (2010).
8. Davies, D. H., Hall, J., Smith, E. H. Non-oxidative converstion of ketone carbonyls into carboxy carbonyls. Comparison of 2-acylthiazoles and 2-acylimidazoles in the aldol condensation and the stereospecific cleavage of an example of the latter to a β-hydroxy ester via the azolium salt. *J. Chem. Soc. Perkin Trans. 1* 2691–2698 (1991).
9. Hiyama, T., Reddy, G. B., Minami, T. & Hanamoto, T. Stereoselective reduction of β,δ-diketo esters. A novel strategy for the synthesis of HMG-CoA reductase inhibitors. *Bull. Chem. Soc. Jpn.* **68,** 350–363 (1995).
10. Evans, D. A., Fandrick, K. R. & Song, H.-J. Enantioselective Friedel–Crafts alkylations of α,β-unsaturated 2-acyl imidazoles catalyzed by bis(oxazolinyl)pyridine–scandium(III) triflate complexes. *J. Am. Chem. Soc.* **127,** 8942–8943 (2005).
11. Myers, M. C., Bharadwaj, A. R., Migram, B. C. & Scheidt, K. A. Catalytic conjugate additions of carbonyl anions under neutral aqueous conditions. *J. Am. Chem. Soc.* **127,** 14675–14680 (2005).
12. Sherbrook, E. M., Jung. H., Cho, D., Baik, M.-H. & Yoon, T. P. Brønsted acid catalysis of photosensitized cycloadditions. *Chem. Sci.* **11,** 856–861 (2020).
13. Note that MeOH is used in this case to avoid transesterification to generate mixed ester products.
14. Rout, S., Das, A. & Singh, V. K. Metal-controlled switching of enantioselectivity in the Mukaiyama–Michael reaction of α,β-unsaturated 2-acyl imidazoles catalyzed by chiral metal–pybox complexes. *J. Org. Chem.* **83,** 5058–5071 (2018).
15. Zhao, L. J., Kwong, C. K. W., Shi, M. & Toy, P. H. Optimization of polystyrene-supported triphenylphosphine catalysts for aza-Morita–Baylis–Hillman reactions. *Tetrahedron* **61,** 12026–12032 (2005).
16. Stals, P. J. M., Phan, T. N. T., Gigmes, D., Paffen, T. F. E., Meijer, E. W. & Palmans, A. R. A. Nitroxide-mediated controlled radical polymerizations of styrene derivatives. *J. Polym. Sci. Part A Polym. Chem.* **50,** 780–791 (2012).
17. Williamson, K. S. & Yoon, T. P. Iron-catalyzed aminohydroxylation of olefins. *J. Am. Chem. Soc.* **132,** 4570–4571 (2010).
18. Gaali, S., Kozany, C., Hoogeland, B., Klein, M., Hausch, F. Facile synthesis of a fluorescent cyclosporin A analogue to study cyclophilin 40 and cyclophilin 18 ligands. *ACS Med. Chem. Lett.* **1,** 536–539 (2010).
19. Zhou, Y., Bandar, J. S. & Buchwald, S. L. Enantioselective CuH-catalyzed hydroacylation employing unsaturated carboxylic acids as aldehyde surrogates. *J. Am. Chem. Soc.* **139,** 8126–8129 (2017).
20. Brown, A. R. & Molander G. A. Suzuki–Miyaura cross-coupling reactions of potassium vinyltrifluoroborate with aryl and heteroaryl electrophiles. *J. Org. Chem.* **71,** 9681–9686 (2016).
21. Texter, K. B., Waymach, R., Kavanagh, P. V., O'Brien, J. E., Talbot, B., Brandt, S. D. & Gardner, E. A. Identification of pyrolysis products of the new psychoactive substance 2-amino-1(4-bromo-2,5-dimethoxyphenyl)ethenone hydrochloride (bk-2C-B) and its iodo analogue bk-2C-I. *Drug Test. Analysis*, **10,** 229–236 (2018).
22. Ball, L. T., Lloyd-Jones, G. C., Russell, C. A. Gold-catalysed oxyarylation of styrenes and mono- and *gem*-disubstituted olefins facilitated by an iodine (III) oxidant. *Chem. Eur. J.* **18,** 2931–2937 (2012).
23. Thordarson, P. Determining association constants from titration experiments in supramolecular chemistry. *Chem. Soc. Rev.* **40,** 1305–1323 (2011).
24. Bruker-AXS (2018). APEX3. *Version 2018.1-0.* Madison, Wisconsin, USA.
25. Krause, L., Herbst-Irmer, R., Sheldrick, G. M. & Stalke, D. Comparison of silver and molybdenum microfocus x-ray sources for single crystal structure determination. *J. Appl. Cryst.* **48,** 3–10 (2015).

26. Sheldrick, G. M. (2013b). XPREP. *Version 2013/1.* Georg-August-Universität Göttingen, Göttingen, Germany
27. Sheldrick, G. M. (2013a). The SHELX homepage, http://shelx.uni-ac.gwdg.de/SHELX/.
28. Sheldrick, G. M. SHELXT – Integrated space-group and crystal-structure determination. *Acta Cryst. A* **71,** 3–8 (2015).
29. Sheldrick, G. M. Crystal structure refinement with SHELXT. *Acta Cryst. C* **71,** 3–8 (2015).
30. Dolomanov, O. V., Bourhis, L. J., Gildea, R. J., Howard, J. A. K. & Puschmann, H. OLEX2: a complete structure solution, refinement, and analysis program. *J. Appl. Cryst.* **42,** 339–341 (2009).
31. Guzei, I. A. (2007–2013). Programs Gn. University of Wisconsin-Madison, Madison, Wisconsin, USA.
32. Parr, R.G. & Yang, W. *Density Functional Theory of Atoms and Molecules* (Oxford University Press, New York, NY, 1989).
33. Bochevarov, A.D., Harder, E., Hughes, T. F., Greenwood, J. R., Braden, D. A., Philipp, D. M., Rinaldo, D., Halls, M. D., Zhang, J. & Friesner, R. Jaguar: a high-performance quantum chemistry software program with strengths in life and materials sciences. *Int. J. Quantum Chem.* **113,** 2110–2142 (2013).
34. Slater, J. C. *Quantum theory of molecules and solids, vol. 4: The Self-Consistent Field for Molecules and Solids.* (McGraw-Hill, New York, NY, 1974).
35. Vosko, S. H., Wilk, L. & Nusair, M. Accurate spin-dependent electron liquid correlation energies for local spin density calculations: a critical analysis. *Can. J. Phys.* **58,** 1200–1211 (1980).
36. Becke, A. D. Density-functional exchange-energy approximation with correct asymptotic behavior. *Phys. Rev. A* **38,** 3098–3100 (1988).
37. Lee, C., Yang, W. & Parr, R. G. Development of the Colle-Salvetti correlation-energy formula into a functional of the electron density. *Phys. Rev. B* **37,** 785–789 (1988)
38. Becke, A. D. Density-functional thermochemistry. III. The role of exact exchange. *J. Chem. Phys.* **98,** 5648–5652 (1993).
39. Grimme, S., Antony, J., Ehrlich, S. & Krieg, S. A consistent and accurate ab initio parametrization of density functional dispersion correction (DFT-D) for the 94 elements H-Pu. *J. Chem. Phys.* **132,** 154104 (210)
40. Ditchfield, R., Hehre, W. J. & Pople, J. A. Self-consistent molecular-orbital methods. IX. An extended Gaussian-type basis for molecular-orbital studies of organic molecules. *J. Chem. Phys.* **54,** 724–728 (1971).
41. Hehre, W. J. & Pople, J. A. Self-consistent molecular orbital methods. XIII. An extended Gaussian-type basis for boron. *J. Chem. Phys*. **56,** 4233–4234 (1972).
42. Binkley, J. S. & Pople, J. A. Self-consistent molecular orbital methods. XIX. Split-valence Gaussian-type basis sets for beryllium. *J. Chem. Phys.* **66,** 879–880 (1977).
43. Hariharan, P. C. & Pople, J. A. The influence of polarization functions on molecular orbital hydrogenation energies. *Theor. Chim. Acta* **28,** 213–222 (1973).
44. Hehre, W. J., Ditchfield, R. & Pople, J. A. Self-consistent molecular orbital methods. XII. Further extensions of Gaussian-type basis sets for use in molecular orbital studies of organic molecules. *J. Chem. Phys*. **56,** 2257–2261 (1972).
45. Francl, M. M., Pietro, W. J., Hehre, W. J., Binkley, J. S., Gordon, M. S., DeFrees, D. J. & Pople, J. A. Self-consistent molecular orbital methods. XXIII. A polarization-type basis set for second-row elements. *J. Chem. Phys.* **77,** 3654–3665 (1982).
46. Shao Y. et al. Advances in molecular quantum chemistry contained in the Q-Chem 4 program package. *Mol. Phys*. **113,** 184–215 (2015).
47. Yanai, T., Tew, D. & Handy, N. A new hybrid exchange–correlation functional using the coulomb-attenuating method (CAM-B3LYP). *Chem. Phys. Lett.* **393,** 51–57 (2004).
48. Hirata, S. & Head-Gordon, M. Time-dependent density functional theory within the Tamm–Dancoff approximation. *Chem. Phys. Lett.* **314,** 291–299 (1999).
