# Supplementary material for: Chiral Brønsted acid-controlled intermolecular asymmetric [2 + 2] photocycloadditions
Source: Nat Commun. 2021 Sep 30;12:5735. doi: 10.1038/s41467-021-25878-9 (PMC8484615; doi:10.1038/s41467-021-25878-9)
Supplement: Supplementary file 2 — Description of Additional Supplementary Files [file 41467_2021_25878_MOESM2_ESM.pdf]

Description of Additional Supplementary Files

File name: Supplementary Data 1
Description: UV-vis spectroscopic studies
